# Substrate-Selective Catalysis Enabled Synthesis of Azaphilone Natural Products

**DOI:** 10.1021/acscentsci.3c01405

**Published:** 2024-02-29

**Authors:** Ye Wang, Katherine J. Torma, Joshua B. Pyser, Paul M. Zimmerman, Alison R. H. Narayan

**Affiliations:** ^†^Life Sciences Institute, ^‡^Department of Chemistry, University of Michigan, Ann Arbor, Michigan 48109, United States

## Abstract

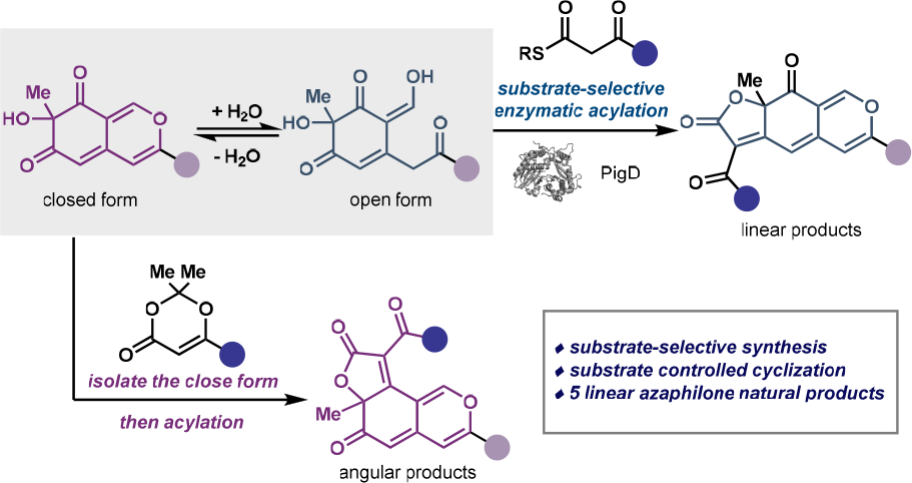

Achieving substrate-selectivity is a central element of nature’s approach to synthesis. By relying on the ability of a catalyst to discriminate between components in a mixture, control can be exerted over which molecules will move forward in a synthesis. This approach can be powerful when realized but can be challenging to duplicate in the laboratory. In this work, substrate-selective catalysis is leveraged to discriminate between two intermediates that exist in equilibrium, subsequently directing the final cyclization to arrive at either the linear or angular tricyclic core common to subsets of azaphilone natural products. By using a flavin-dependent monooxygenase (FDMO) in sequence with an acyl transferase (AT), the conversion of several orcinaldehyde substrates directly to the corresponding linear tricyclic azaphilones in a single reaction vessel was achieved. Further, mechanistic studies support that a substrate equilibrium together with enzyme substrate selectivity play an import role in the selectivity of the final cyclization step. Using this strategy, five azaphilone natural products were synthesized for the first time as well as a number of unnatural derivatives thereof.

## Abstract 2

A substrate-selective biocatalytic strategy is used to discriminate between two intermediates in equilibrium, subsequently directing a cyclization step to arrive at either linear or angular tricyclic azaphilone natural products.

## Introduction

**Figure 1:**
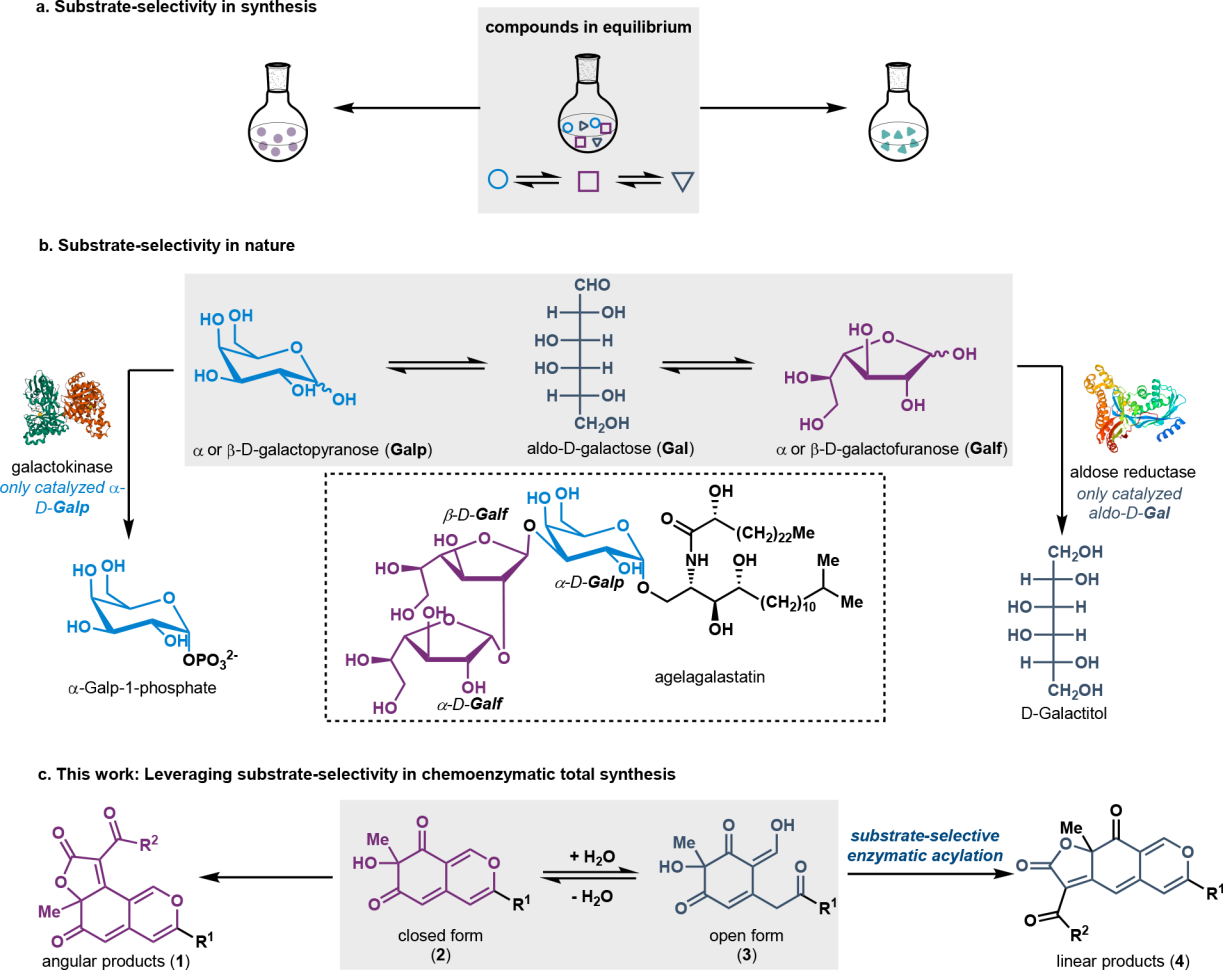
Substrate selectivity can be used to effectively and selectively make desired products. (a) Substrate selectivity allows for selective transformations for a mixture of intermediates in equilibrium. (b) Example of substrate selective transformations in nature with the sugar galactose. (c) Utilizing a substrate selective transformation to achieve different azaphilone natural products.

Selectivity is a central consideration in planning a synthetic strategy toward a target molecule.^1^ Therefore, highly selective transformations are of great value, and emerging methods often seek to achieve high levels of chemo-, site-, and enantioselectivity. One additional form of selectivity that is often less developed is substrate selectivity as methods in traditional organic chemistry commonly strive to achieve broad substrate compatibility. In contrast, enzymes in nature often rely on substrate selectivity to transform specific metabolites in complex mixtures.^2^ Despite the dearth of substrate-selective methods, when substrate selectivity can be accomplished, it can enable one-pot reactions and also discriminate between substrates in equilibrium (Figure 1a).^3^

Biosynthetic pathways provide a rich source of substrate-selective catalysts. Based on this selectivity, it is possible to access divergent pathways toward structurally distinct natural products from common intermediates that exist in equilibrium. Carbohydrates provide a classic example of this phenomenon, with an equilibrium between the cyclic and linear forms (Figure 1b).^4^ With complementary substrate-selective enzymes, different forms of a sugar can be transformed into related natural products. Specifically, d-galactose can exist in at least five forms, which are all potential substrates that can be advanced toward different natural products. For example, d-galactitol, formed from aldo-d-galactose through a substrate-selective enzymatic reduction.^5^ From the same equilibrium, d-Galp can undergo a substrate-selective reaction to form α-Glap-1-phosphate.^6^ Aside from the linear form Gal, all four other isomers bear similar structural features (Figure 1b). By leveraging this substrate-selective approach, these different forms of d-galactose can be elaborated to an array of complex natural products. For instance, agelagalastatin is derived from galactose by incorporating three distinct forms of galactose.^7^ When equilibrating compounds possess functional groups with distinct reactivity (e.g., the formyl group of the linear carbohydrate), it is plausible to adopt this strategy in the laboratory using small molecule reagents or catalysts; however, if the reactivity of the equilibrating compounds is not distinct, this strategy becomes challenging to implement in the lab. Biocatalysis provides an opportunity to exploit substrate-selective strategies and access divergent end points from common equilibrating intermediates. To demonstrate the potential of this substrate-selective strategy, we envisioned a divergent chemoenzymatic approach toward tricyclic azaphilone natural products with two distinct core structures (Figure 1c).

**Figure 2:**
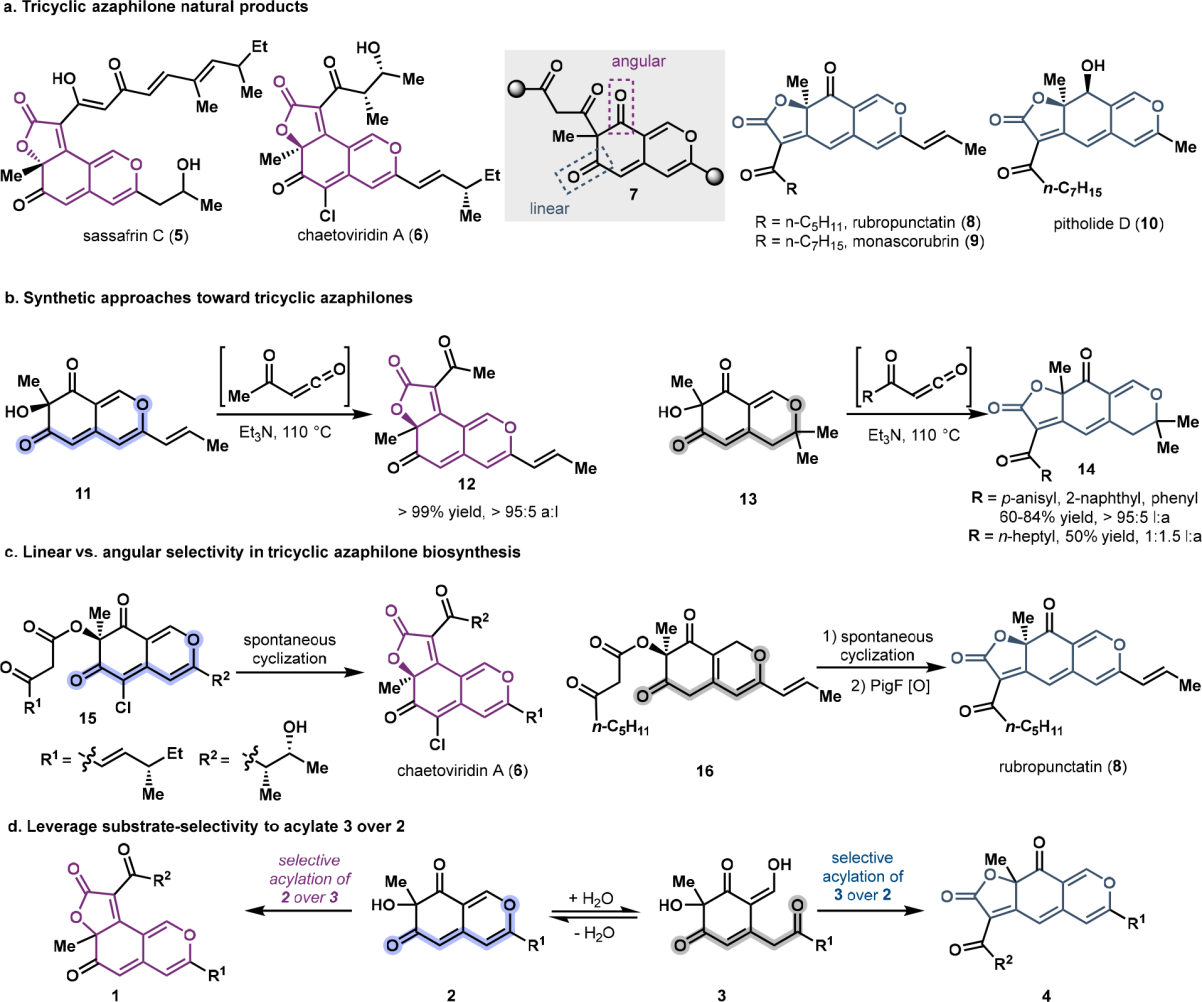
Tricyclic azaphilone natural products and methods to synthesize them. (a) Examples of linear and angular tricyclic azaphilone natural products. (b) Previous synthetic approaches toward tricyclic azaphilones. (c) Biosynthesis examples of tricyclic azaphilones. (d) Substrate selective approach to selectively form the linear and angular tricyclic azaphilones. Purple highlights the linear ketone conjugation with an enol functional group; gray highlights that the conjugation was broken.

Tricyclic azaphilones are a subset of this expansive family of fungal natural products that can be classified into two categories: angular and linear (see **5** and **8**, respectively, Figure 2a).^8^ These tricyclic azaphilones are known fungal pigments that have garnered growing interest for their antimicrobial, cytotoxic, antioxidative, and anti-inflammatory activities.^8^ Recently, more general azaphilone cores were discovered as primary amine selective reagents for bioconjugation which can selectively modify the lipid components of Gram-positive bacteria. In this context, azaphilones exhibited high specificity for forming lysine conjugates over other amino acids.^9^ These properties make tricyclic azaphilone natural products attractive synthetic targets, yet these molecules present significant challenge due to the difficulty in accessing either the linear or angular scaffolds in a selective manner.^11−13^

To date, reported examples for selective tricyclic azaphilone cyclization rely on the reactivity of the substrate to dictate formation of either angular or linear tricyclic products, typically as mixtures of the two. In our investigation of the natural product, trichoflectin, we demonstrated the preference for formation of the angular product under a given set of conditions reported by Franck and co-workers.^10−12^ In contrast, the original report by Franck et al. demonstrated that selectivity for the linear product could be achieved when the substrate contained one fewer degree of unsaturation.^12^ Comparison of these substrates demonstrates that the angular product was favored when the linear ketone was in conjugation with an enol functional group (see **11**, Figure 2b). In contrast, the linear product was formed from substrate **13**, in which this conjugation was broken. Nature navigates this substrate-controlled selectivity by adjusting the oxidation state of the azaphilone core post-tricycle formation, as demonstrated in the biosynthesis of rubropunctatin.^13^ Interestingly, the analogous angular tricyclic azaphilone can form the substrate when conjugation exists between the linear ketone and the enol of the substrate (Figure 2c).^14^ In both cases, the cyclization selectivity is substrate-controlled.

We envisioned leveraging substrate-selective catalysis and substrate-controlled cyclization to enable either angular or linear azaphilones from a common intermediate. This strategy would provide rapid access to this natural product class and the opportunity to investigate the impact of the ring structure and other elements contributing to a given compound’s bioactivity. Herein, we report our progress toward these goals.

## Results and Discussion

### Reaction Development

Based on the established access to angular azaphilone natural products from **11**,^10^ we questioned if it could be possible to access linear tricyclic azaphilones from the same substrate to arrive at natural products such as rubropunctatin (**8**). We postulated that access to the linear tricycle from a common intermediate would require some alteration of the bicyclic core prior to installation of the lactone through an acylation followed by the Knoevenagel condensation/cyclization strategy to access linear natural products, which to date have only been isolated from natural sources with no reported syntheses.

**Figure 3:**
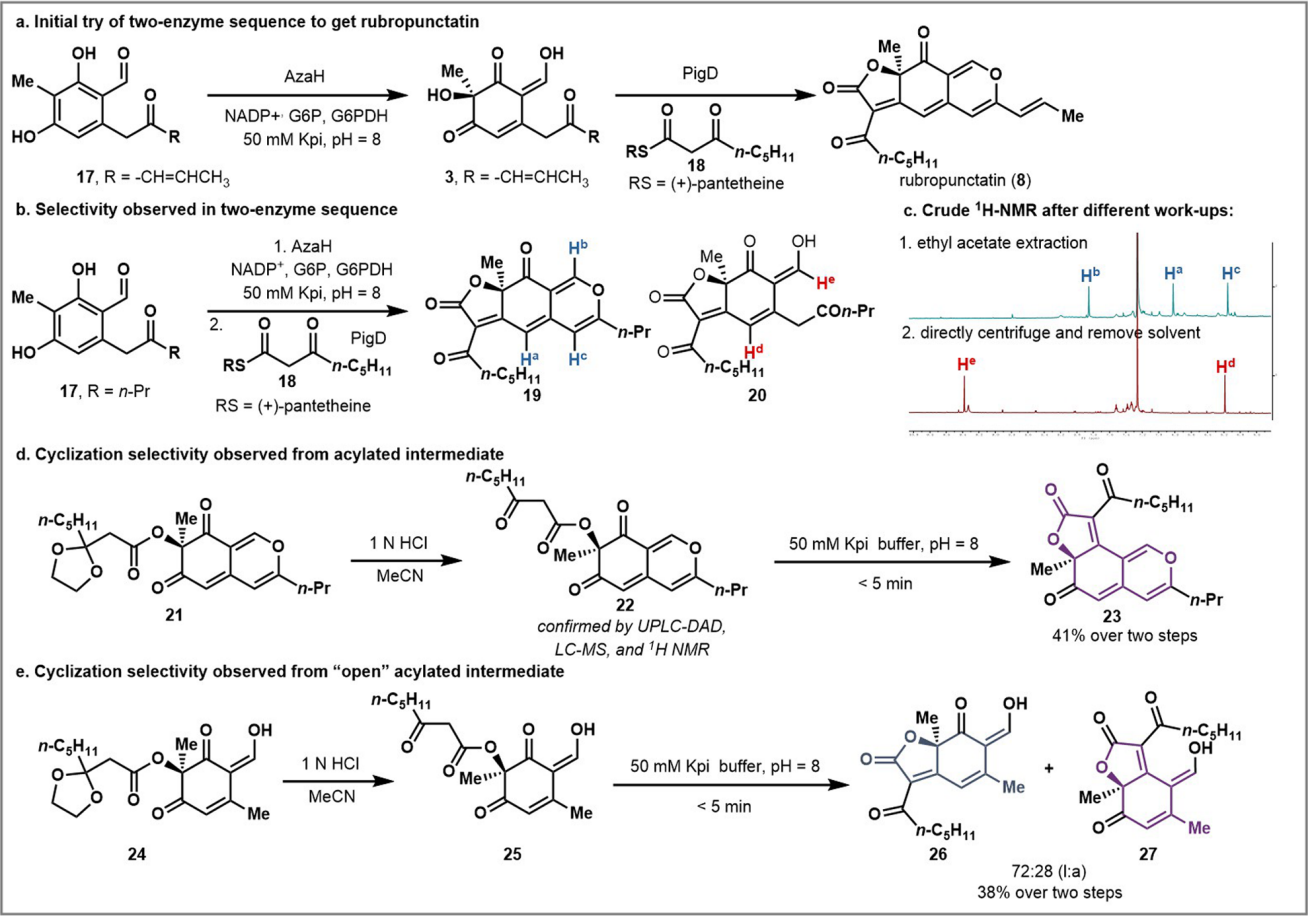
Assessing the cyclization selectivity of various acylated intermediates. (a) Initial try of the two-enzyme sequence to get rubropunctatin. (b) The two-enzyme sequence of AzaH followed by PigD affords the linear tricycle in its closed and open forms. (c) ^1^H NMR of the different extraction and quenching conditions. (d) The angular cyclization selectivity of the closed form substrate. (e) The linear cyclization selectivity of the open form substrate.

Through our work on a flavin-dependent monooxygenase (FDMO) AzaH, which mediates oxidative dearomatization, we came to appreciate that under aqueous reaction conditions the direct product of dearomatization (**3**) is in equilibrium with bicycle **2** (Figure 2d). Further experimentation with substrates which vary at the R^1^ group revealed significant differences in the equilibrium ratio. For example, when R^1^ is an *n*-propyl (*n*-Pr) group, the major product is the open-ring form **3**. However, when R^1^ is CH=CHCH_3_, the presence of additional conjugation leads to a nearly equal distribution between the open and closed forms **2** and **3** (see Supporting Information IX for details). Extraction of the product into organic solvent and removal of water affords solely bicycle **2**; however, if the open form **3** could be acylated, we anticipated that the subsequent cyclization would afford linear-type azaphilone products, due to the open form not having an electron donating group conjugated with the linear ketone. To realize linear product **4**, suitable acylation conditions that display both (a) high substrate-selectivity for the open *o*-quinol **3** over the closed form **2** would be necessary and (b) high chemoselectivity for the tertiary hydroxyl group over the enolic hydroxyl group in an aqueous phase. To this end, a small library of acyl transferases (ATs) was built based on their sequence similarity to MrPigD (PigD),^14^ an AT involved in the biosynthesis of rubropunctatin. Incubating each AT with *o*-quinol **3** and β-ketothioester **18** revealed that PigD was the best catalyst for the desired acylation.^13^ With PigD in hand, following after the AzaH step, the tricyclic azaphilone product was directly afforded from orcinaldehyde substrate **3** (R_1_ = −CH=CHCH_3_) without the need to isolate the dearomatized intermediate (Figure 3a).^13,15,16^ Upon isolation of the product, we confirmed that the linear azaphilone, rubropunctatin (**8**), was exclusively formed, achieving the first total synthesis of a linear tricyclic azaphilone natural product.

### Mechanism Exploration

We propose that the formation of the linear tricycle arises from the selectivity of PigD for acylation of the open form of *o*-quinol **3**. This is based on observations made during the optimization of the preparative-scale reaction of substrate **17**. While optimizing the isolation of **19**, we observed that the solvent used for the workup afforded two different products (Figure 3b). When using ethyl acetate to extract, three characteristic protons of rubropunctatin were detected in the ^1^H NMR spectrum. However, if acetonitrile was used instead to quench the reaction, the crude NMR only showed two proton signals in the aromatic region, which can be assigned to the open form of the linear product **20** (Figure 3c). The UPLC traces of the reaction mixture support **20** as the major product of this reaction, which slowly undergoes ring closure in the NMR tube to tricycle **19**. These data support that **3** is the preferred substrate of PigD.

To understand the factors that govern formation of the linear tricycle (**4**) over the angular product (**1**), we first sought to understand the reactivity of the acylated intermediates. Toward this end, **21** was synthesized and converted to **22** under acidic conditions. **22** possess both the closed form of the bicycle and the β-ketoester group which is primed for cyclization. Interestingly, under the same buffer conditions as used for the PigD acylation reaction, the angular product was exclusively formed (Figure 3d). This provides evidence against the linear product being formed selectively by kinetic control at room temperature. To investigate the cyclization selectivity of the open form, the dearomatization product **25**, which cannot adopt the closed form, was elaborated through the same synthetic route. Under the same conditions, the Knoevenagel condensation of **25** proceeded to afford a 72:28 ratio of products **26** and **27** favoring the product that corresponds to linear selectivity (Figure 3e).

**Figure 4:**
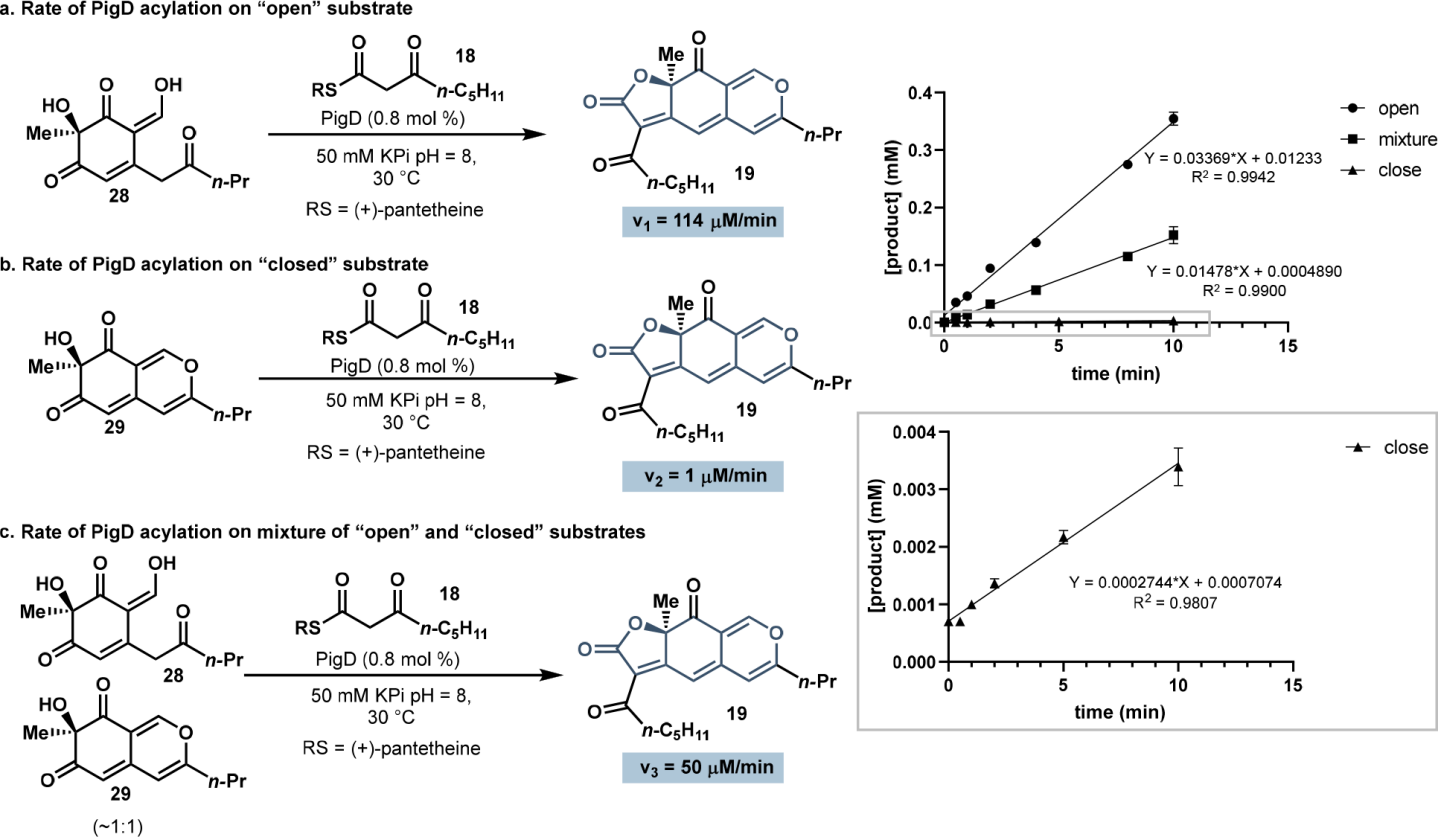
Comparison of the initial velocity of PigD reactions with open and closed forms of substrate. (a) The kinetics of the open form substrate. (b) The kinetics of the closed form substrate. (c) The kinetics of equal amounts of open and closed substrates.

To further support the divergent cyclization selectivity from the open and closed forms of the acylated intermediate, a kinetic study was designed to assess the substrate selectivity of PigD (Figure 4). Under the standard telescoped reaction conditions, we can measure a rate of product formation forward from **28** that is 114 μM/min (Figure 4a). To access the closed intermediate **29**, the product of the dearomatization reaction was extracted into ethyl acetate and purified by prep HPLC, which induced the formation of the bicycle **29**. From **29**, a much slower rate, 1 μM/min, was measured in the PigD acylation reaction (Figure 4b). We hypothesize that the difference in rates of acylation of the open and closed forms (see **28** and **29**, respectively) is based on the substrate selectivity of PigD. When an equivalent of closed form **29** was added to the standard telescoped reaction conditions, the rate of the second step decreased to 50 μM/min (Figure 4c).^17^ Together these data support that PigD’s preferred substate is the open form, and that PigD’s substrate selectivity as the origin of the linear selectivity can be uniquely achieved using this chemoenzymatic strategy.

### Reaction Scope

With experimental support for the substrate selectivity of PigD enabling the synthesis of linear tricycles, the next question rests in the substrate scope of this selective two-enzyme sequence. To answer this question, a range of substrates and thioester acyl donors were tested in this two-enzyme sequence. The incorporation of different groups, specifically at R^1^ and R^2^, was designed to probe the diversity of groups at these positions that map onto the chemical diversity present among natural azaphilones or to provide functional handles for diversification at these positions (see **4**, Table 1). Overall, this strategy proved useful for accessing a range of tricyclic azaphilones with good selectivity for the linear tricyclic core over the angular core (83:17 to >95:5 l/a). Further, the two-enzyme sequence displayed functional group tolerance in the resorcinol substrate (see R^1^, Table 1) with the dearomatization step proceeding in 91–99% conversion and the PigD acylation affording conversions of 74–91%. It is worth noting that, as mentioned earlier, when the R^1^ group is CH=CHCH_3_, the open form **3** and the closed form **2** of the dearomatization intermediate exist in nearly a 1:1 ratio (see Supporting Information**IX** for detail). However, in the second step, the conversion remains almost complete (99% conversion), and UPLC and NMR detect the linear tricycle (**8**) as the major product (>95:5 l:a). This further indicates that PigD selectively acylates the open intermediate **3**. In addition, various chain lengths on the thioester substrate were also tolerated for this one-pot sequence.^18−21^ Generally, the PigD acylation proceeded in low yield with a methyl ketone, with tricycle **35** detected in trace amounts. However, when the length of the R^2^ chain was increased to three carbons, an increase in conversion to 17% was observed, whereas five to seven carbon chains afforded yields ranging from 73% to 91% to deliver tricycles with good selectivity for the linear products 91:9–94:6 rr. When the chain length was increased further, solubility became an issue, leading to decreased conversion (see **39**).

**Table 1:**
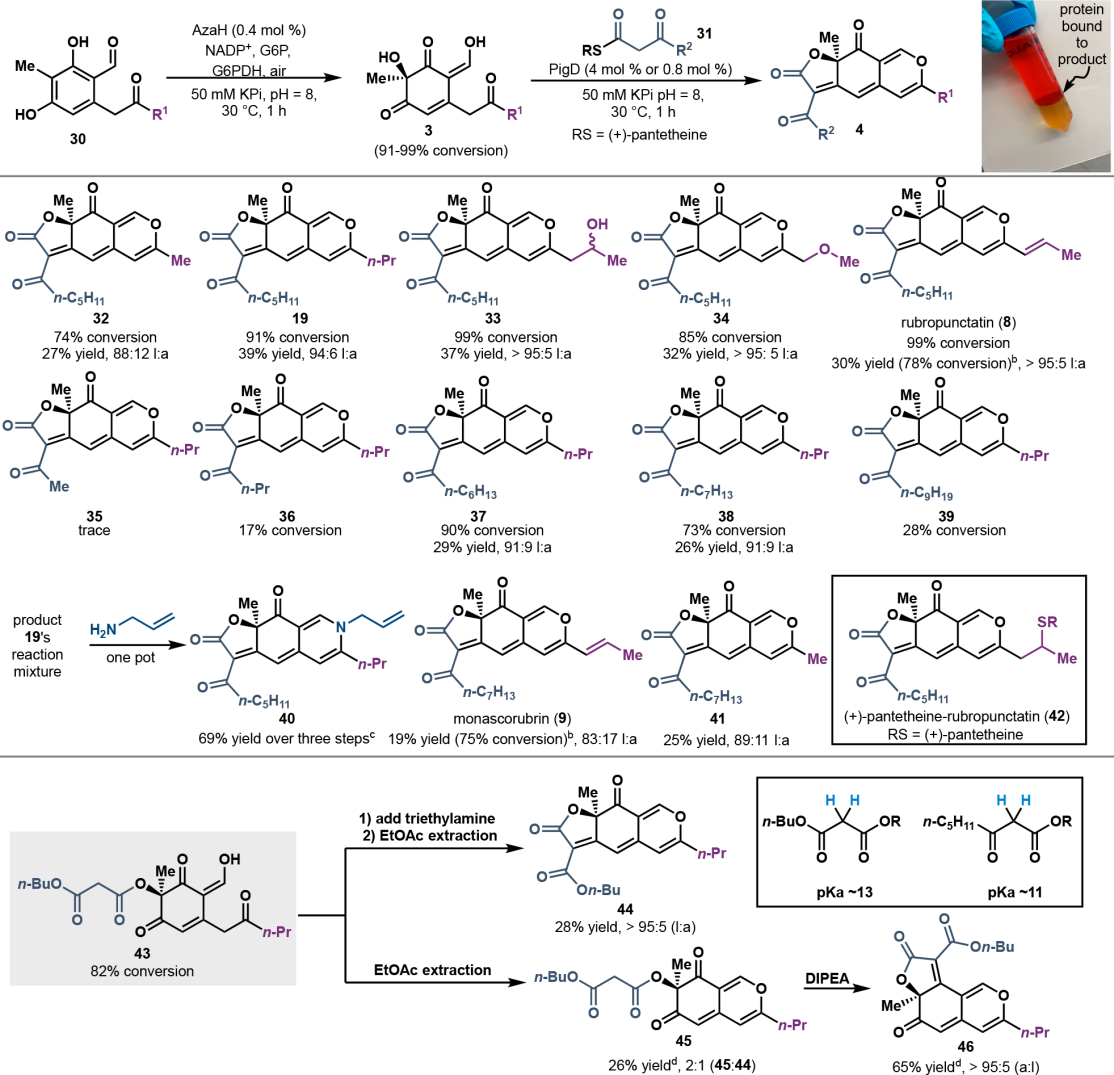
Substrate Scope of Two-Enzyme Sequence to Access Linear Tricyclic Azaphilones^a^

a Reaction conditions of step 1:2.5 mM substrate, 10 μM AzaH, 1 mM NADP^+^, 5 mM glucose-6-phosphate (G6P), 1 U mL^–1^ glucose-6-phosphate dehydrogenase (G6PDH), 50 mM potassium phosphate buffer, 30 °C, 1 h. Reaction conditions of step 2, anylatical scale: dilute the step 1 mixture five times with 50 mM potassium phosphate buffer, then add 1.1 equiv, 0.55 mM thiolester **31**, 20 μM PigD, 30 °C, 1 h. Reaction conditions of step 2, preparative-scale: the mixture of step 1, add 1.1 equiv, 2.75 mM thiolester **31**, 20 μM PigD, 30 °C, 1 h. Conversion depends on substrate consumption; yield refers to the isolated yield on a preparative scale.

b Preparative-scale, 2 equiv, 5 mM maleimide added in the second step as thiol scavenger.

c Reaction conditions of step 3: the mixture of preparative-scale step 2, 10% by volume of allylamine added, 30 °C, 5 min.

d NMR yield.

Based on this analytical data, preparative-scale reactions were also tested on substrates with good conversions. Although the conversions for the biocatalytic sequence were high (73–99%), the isolated yields were low (26–39%, Table 1). This observation prompted an optimization of the workup and isolation procedure from the two-enzyme sequence. Upon precipitation of protein and cellular debris during the reaction workup, the intense red color of the pellet suggested that the characteristically red azaphilone product was also precipitating out of solution, potentially covalently linked to protein through condensation of free amino groups onto the azaphilone pyran ring.^8,22,23^ To solve this problem, a small molecule amine was added in an attempt to outcompete the condensation with amino groups present in the protein or other biomolecules. After stirring for 5 min with an amine, the product could be isolated with an improved yield of 69%. For alkenyl substrate **3** (R_1_ = −CH=CHCH_3_), the unexpected thiol Michael addition side reaction also complicated scale-up, leading to variable isolated yields, dependent on reaction time. Maleimide was used as a thiol scavenger to capture free pantetheine liberated over the course of the acylation reaction.^24,25^ After the addition of maleimide (2 equiv), this byproduct was not detected, allowing for a 30% isolated yield of rubropunctatin with the second step conversions of 78%. Using an analogous approach, monascorubrin (**9**) was isolated in 19% yield over two steps with a conversion of 75% in the second step.

As many azaphilone natural products exist with variation at R^2^ that exceed the substrate scope of PigD, we sought a thioester acyl donor that could allow for downstream functionalization.^8^ When ester **31** (R^2^ = O*n*Bu) was tested as the acyl group donor, 82% conversion of the *o*-quinol intermediate was observed on an analytical scale. The resulting acylated intermediate displayed unique behavior. With a p*K*_a_ of the 1,3-diester not low enough for the Knoevenagel condensation to spontaneously proceed under the enzymatic acylation conditions, the direct acylation product (**43**) was observed as the major product. From **43**, either the linear or angular tricycle could be selectively accessed. If **43** was treated with triethylamine, the cyclization from **43** was induced to afford the linear product following extraction into ethyl acetate. In contrast, direct extraction of **43** into ethyl acetate afforded the acylated bicycle **45** as a major product, which upon treatment with Hünig’s base cyclized to give the angular tricycle **46**. This further points to the substrate control as the origin of linear selectivity rather than a cyclization that is dictated by the AT.

### Application

**Figure 5:**
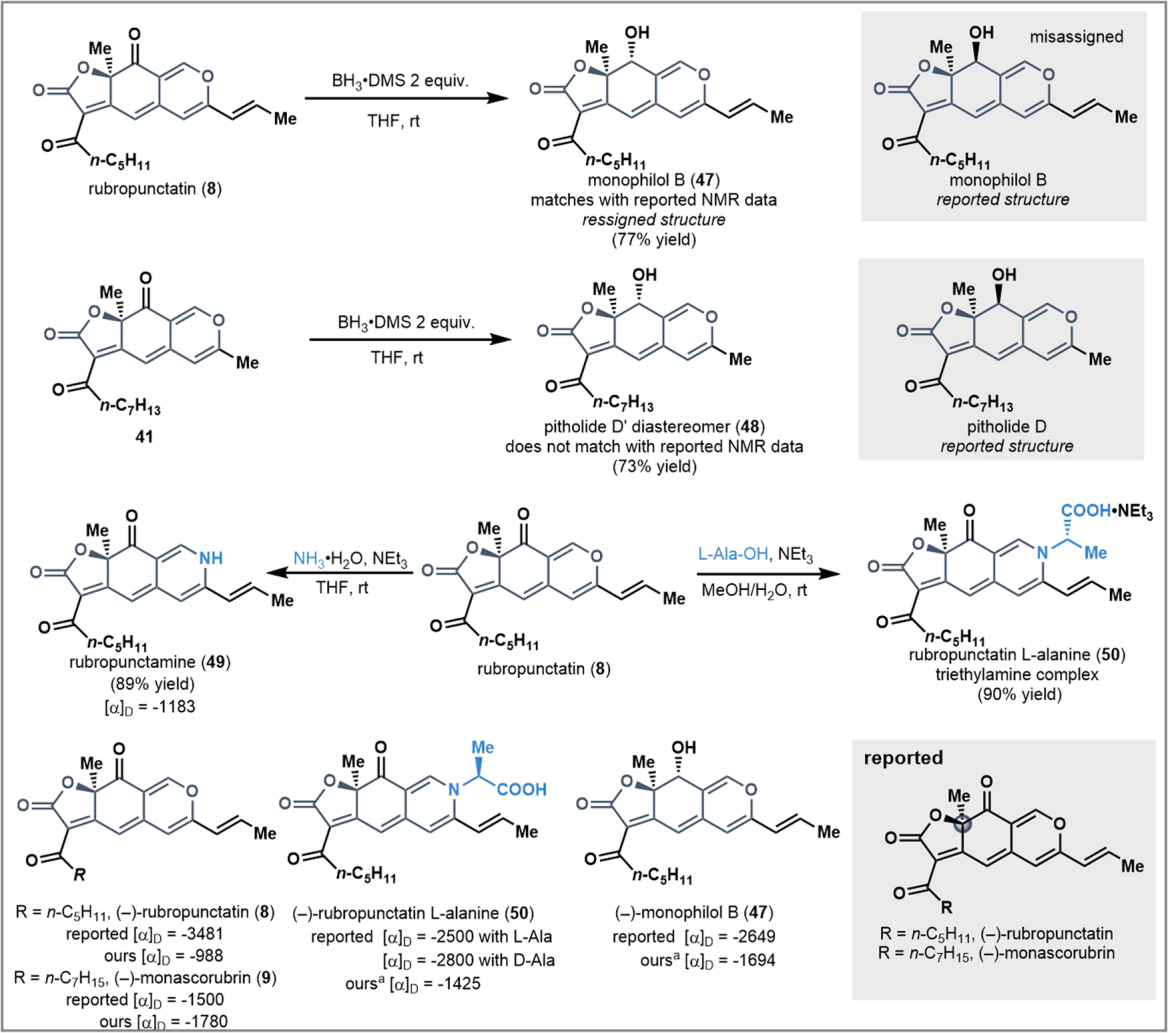
Transformation of a common tricyclic azaphilone core into a variety of natural products and related structural reassignments.

With an established strategy for the synthesis of tricyclic linear azaphilones, we sought to access additional natural products in this family beyond rubropunctatin (**8**) and monascorubrin (**9**). First, we investigated the reduction of the azaphilone core to access natural products such as monophilol B (**47**) and pitholide D (**10**; Figure 5).^26,27^ After exploring several reduction conditions, BH_3_**·**DMS was identified as a broadly applicable reducing agent. For example, rubropunctatin (**8**) could be directly reduced to afford monophilol B (**47**) as a single diastereomer in 77% yield. However, the same reaction with **41** gave an unexpected result, delivering a product that did not match the reported ^1^H NMR spectrum of the natural product pitholide D (**10**). Nearly all the ^1^H NMR peaks matched with the isolation paper, except for the methine at the newly set stereocenter, suggesting that a diastereomer of the pitholide D was synthesized in 73% yield. Since the ^1^H NMR did not match the reported data generated using the same reduction method for pitholide D (**10**) as monophilol B (**47**), which have the same reported relative configuration, it became clear that at least one of these structures was misassigned (Figure 5).^26,27^ To further characterize our synthetic material, monophilol B was acylated with a 4-nitrobenzoyl group. An NOE signal between the *ortho*-proton on the 4-nitrobenzoyl group and the relevant methyl group was detected (see SI, compound **S20**), which supports that the methyl group and the hydroxyl group are arranged in a *syn* fashion (see Figure 5). In addition, a number of natural products were accessed through amination of rubropuncatin. For example, rubropunctamine (**49**) and rubropunctatin l-alanine (**50**) were synthesized from the corresponding amine in high yields (89% and 90%, respectively).^28−30^ With synthetic natural products in hand, the absolute configuration of each compound was characterized by comparing the experimental optical rotations to those reported for the natural compounds. Because the enzymatic dearomatization with AzaH exclusively affords the *R* product and the optical rotation measurements for each synthetic compound were the same sign as those reported in the isolation papers, the absolute configuration of (−)-rubropunctatin and (−)-monascorubrin are *R* as originally reported.^30−32^

## Conclusion

In summary, a substrate selective strategy was developed to access linear tricyclic azaphilone natural products. Through a two-enzyme, one-pot sequence, linear tricyclic azaphilone scaffolds were built from readily available resorcinol starting materials. Specially, five linear azaphilone natural products were synthesized for the first time. In addition to this synthetic achievement, the origins of the observed selectivity were investigated to support that an enzyme, PigD, selectively acylates the open form of the substrate, which controls the selectivity of the subsequent cyclization step to afford the linear azaphilone tricyclic core. This demonstrates the utility of a substrate-selective synthetic approach and the opportunity to use biocatalysis to achieve this type of selectivity.

## Data Availability

The data underlying this study are available in the published article and its Supporting Information.

## Supporting Information Available

Experimental procedures, characterization of products, NMR spectra (PDF)
