# Supplementary material for: Substrate-Selective Catalysis Enabled Synthesis of Azaphilone Natural Products
Source: ACS Cent Sci. 2024 Feb 29;10(3):708–16. doi: 10.1021/acscentsci.3c01405 (PMC10979483; doi:10.1021/acscentsci.3c01405)
Supplement: Supplementary file 1 — oc3c01405_si_001.pdf [file oc3c01405_si_001.pdf]

Supplementary Information for

Substrate-selective catalysis enabled synthesis of azaphilone natural products

Ye Wang, Katherine J. Torma, Joshua B. Pyser, Paul Zimmerman, and Alison R. H. Narayan*

*Corresponding author
Email: arhardin@umich.edu

Table of contents

I. Chemical synthesis 3
II. Plasmids and Proteins 6
III. Protein overexpression and purification 8
i. Protein overexpression: 8
ii. Protein purification procedure: 8
IV. Biocatalytic reactions 9
V. Synthesis to support mechanistic insights. 14
VI. Transformation. 17
VII. Standard curves and quantification of biocatalytic reactions 20
VIII. Kinetic experiment. 21
IX. Different ratio of close (2) and open form (3) depend on R_1 group. 23
X. UPLC traces for analytical-scale biocatlytic reactions 24
XI. Preparative-scale biocatalytic reactions of Substrate S4 with C5(18) and C7 (S11) thioester. 51
XII. Natural product NMR comparison and assignment of absolute configurations 53
i. Rubropunctatin NMR comparison table 53
ii. Monascorubrin NMR comparison table: 55
iii. Monaphilol B NMR comparison table: 57
iv. Rubropunctamine NMR comparison table: 59
v. Rubropunctain alanine NMR compariosn table: 61
vi. Natural product optical rotation and our assignment of configurations:[10-12] 64
XIII. ^{1}H NMR and ^{13}C NMR spectra of compounds 65
XIV. References 130

I. Chemical synthesis

General considerations. All reagents were used as received unless otherwise noted. Reactions were carried out under a nitrogen atmosphere using standard Schlenck techniques unless otherwise noted. Solvents were degassed and dried over aluminum columns on an MBraun solvent system (Innovative Technology, Inc., Model PS-00-3). Reactions were monitored by thin layer chromatography using Machery-Nagel 60 F_{254} precoated silica TLC plates (0.25 mm) or Merck Silica Gel 60 F_{254} precoated silica TLC plates (0.25 mm) which were visualized using UV, *p*-anisaldehyde, CAM, DNP, or bromocresol green stain. Flash column chromatography was performed using Machery-Nagel 60 µm (230-400 mesh) silica gel. All compounds purified by column chromatography were sufficiently pure for use in further experiments unless otherwise indicated. ^{1}H and ^{13}C NMR spectra were obtained in $CDCl_3$ at rt (25 °C), unless otherwise noted, on Varian 400 MHz or Bruker 600 MHz spectrometers. Chemical shifts of ^{1}H NMR spectra were recorded in parts per million (ppm) on the δ scale. High resolution electrospray mass spectra were obtained on an Agilent UPLC-QTOF at the University of Michigan Life Sciences Institute or Agilent UPLC-TOF at the University of Michigan Life Sciences Institute.

Synthesis of substrates

All aromatic substrates (except **S4**) were prepared as reported previously by Baker Dockrey, S. A. et al.[1] **S4** was prepared as reported previously by Pyser, J. B. et al.[2]

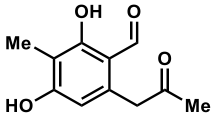

2,4-dihydroxy-3-methyl-6-(2-oxopropyl)benzaldehyde (S1). **^{1}H NMR** (600 MHz, $CDCl_3$) δ 12.66 (s, 1H), 9.87 (s, 1H), 6.21 (s, 1H), 5.62 (s, 1H), 3.92 (s, 2H), 2.25 (s, 3H), 2.09 (s, 3H). All spectra obtained were constant with literature values.[1]

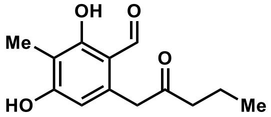

2,4-dihydroxy-3-methyl-6-(2-oxopentyl)benzaldehyde (17). **^{1}H NMR** (600 MHz, $CDCl_3$) δ 12.63 (s, 1H), 9.86 (s, 1H), 6.18 (s, 1H), 3.90 (s, 2H), 2.52 (t, J = 7.3 Hz, 2H), 2.07 (s, 3H), 1.62 (q, J = 7.4 Hz, 2H), 0.91 (t, J = 7.4 Hz, 3H). All spectra obtained were constant with literature values.[1]

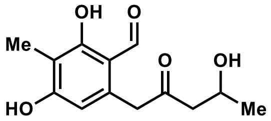

2,4-dihydroxy-6-(4-hydroxy-2-oxopentyl)-3-methylbenzaldehyde (S2). **^{1}H NMR** (600 MHz, CD_3OD) δ 9.77 (s, 1H), 6.23 (s, 1H), 4.26 – 4.18 (m, 1H), 4.04 (s, 2H), 2.71 (dd, *J* = 15.8, 8.2 Hz, 1H), 2.62 (dd, *J* = 15.8, 4.5 Hz, 1H), 2.01 (s, 3H), 1.18 (d, *J* = 6.3 Hz, 3H). All spectra obtained were constant with literature values.[1]

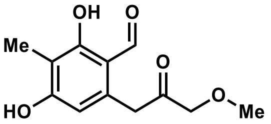

2,4-dihydroxy-6-(3-methoxy-2-oxopropyl)-3-methylbenzaldehyde (S3). **^{1}H NMR** (600 MHz, CD_3OD) δ 9.81 (s, 1H), 6.24 (s, 1H), 4.19 (s, 2H), 4.01 (s, 2H), 3.39 (s, 3H), 2.01 (s, 3H). All spectra obtained were constant with literature values.[1]

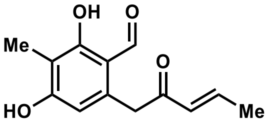

(*E*)-2,4-dihydroxy-3-methyl-6-(2-oxopent-3-en-1-yl)benzaldehyde (S4). **^{1}H NMR** (400 MHz, CD_3OD) δ 9.78 (s, 1H), 7.15 – 7.00 (m, 1H), 6.29 – 6.22 (m, 2H), 4.13 (s, 2H), 2.01 (s, 3H), 1.93 (dd, *J* = 6.8, 1.5 Hz, 3H). All spectra obtained were constant with literature values.[2]

Preparation of ketoacyl derivatives 3a-3f (fellowed a general procudue reported by Vinayak Agarwal et al.[3])

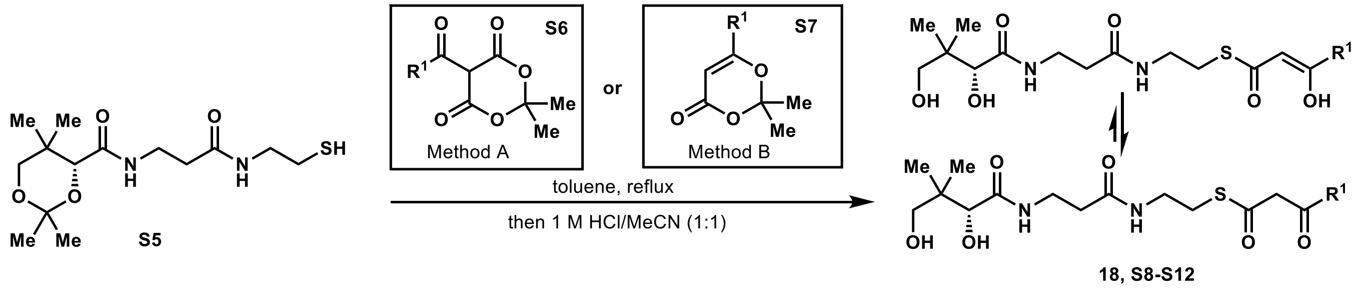

General procedure:

Method A: To thiol **S5** (1 equiv) in toluene (0.1 M), acyl meldrum's acid **S6** (1.5 equiv) was added. This reaction mixture was refluxed for 12 h. After, the solvent was removed and the resulting oil was purified by flash column chromatography (hexanes/EtOAc 1:1 → EtOAc). The thioester was then dissolved in 1 M HCl/MeCN (1:1, 0.1 M) and the reaction mixture was stirred for 5 h. The solvent was evaporated, and the residue was subjected to flash column chromatography (CH_2Cl_2/MeOH 20:1) to give the final product.

Method B: To thiol **S5** (1 equiv) in toluene (0.1 M), ester **S7** (1.5 equiv) was added. The mixture was refluxed for 12 h. After, the solvent was removed, and the resulting oil was purified by flash column chromatography (hexanes/EtOAc 1:1 → EtOAc). The resulting thioester was dissolved in 1 M HCl/MeCN (1:1, 0.1 M) and the mixture was stirred for 5 h. The solvent was evaporated, and the residue was subjected to flash column chromatography (CH_2Cl_2/MeOH 20:1) to give the final product.
Note: all of these ketoacyl derivatives existed in an equilibrium of ketone and enol.

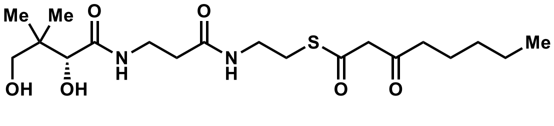

(*R*)-S-(2-(3-(2,4-dihydroxy-3,3-dimethylbutanamido)propanamido)ethyl) 3-oxooctanethioate (18). **Method B**, 426.6 mg, 39% yield, ketone form: enol form = 3:1. Pale yellow oil. **^{1}H NMR** (600 MHz, $CDCl_3$) δ 7.55 – 7.44 (m, 1H), 7.02 – 6.90 (m, 1H), 4.71 (s, 1H), 4.14 (s, 1H), 3.95 (s, 1H), 3.67 (s, 2H),

3.55 – 3.33 (m, 6H), 3.09 – 2.96 (m, 2H), $2.49_{\text{ketone form}}$ and $2.12_{\text{enol form}}$ (t, J = 7.4 Hz, $2H_{\text{total}}$), 2.40 (t, J = 6.1 Hz, 2H), 1.54 (p, J = 7.4 Hz, 2H), 1.35 – 1.18 (m, 4H), 0.94 (s, 3H), 0.87 (s, 3H), 0.85 (t, J = 7.1 Hz, 3H). All spectra obtained were constant with literature values.[4]

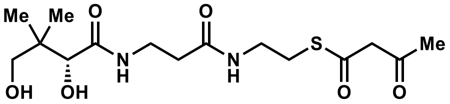

(*R*)-S-(2-(3-(2,4-dihydroxy-3,3-dimethylbutanamido)propanamido)ethyl) 3-oxobutanethioate (S8). Method A, 160.0 mg, 40% yield, ketone form: enol form = 1.3:1. Pale yellow oil. **^{1}H NMR** (600 MHz, CD_3OD) δ 8.28 – 8.15 (m, 1H), 8.01 – 7.89 (m, 1H), 3.89 (s, 1H), 3.55 – 3.32 (m, 8H), 3.06 (t, J = 6.6 Hz, 2H), 2.41 (t, J = 6.6 Hz, 2H), $2.22_{\text{ketone form}}$ and $1.93_{\text{enol form}}$ (s, $3H_{\text{total}}$), 0.92 (s, 6H). All spectra obtained were constant with literature values.[3]

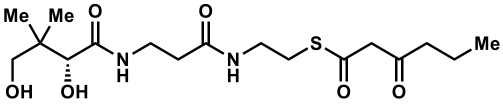

(*R*)-S-(2-(3-(2,4-dihydroxy-3,3-dimethylbutanamido)propanamido)ethyl) 3-oxohexanethioate (S9). Method A, 77.4 mg, 43% yield, ketone form: enol form = 2.8:1. Pale yellow oil. **^{1}H NMR** (600 MHz, $CDCl_3$) δ 7.43 (s, 1H), 6.77 – 6.52 (m, 1H), 4.30 (s, 1H), 3.99 (s, 1H), 3.70 (s, 2H), 3.62 – 3.37 (m, 6H), 3.08 (dq, J = 18.9, 7.1, 6.7 Hz, 2H), $2.51_{\text{ketone form}}$ and $2.14_{\text{enol form}}$ (t, J = 7.2 Hz, $2H_{\text{total}}$), 1.61 (q, J = 7.3 Hz, 2H), 0.99 (s, 3H), 0.96 – 0.88 (m, 6H). **^{13}C NMR** (150 MHz, $CDCl_3$) δ 203.0, 194.2, 192.7, 177.6, 173.9, 173.8, 172.1, 171.9, 99.4, 77.6, 70.9, 57.2, 45.5, 39.9, 39.5, 39.2, 36.9, 35.80, 35.75, 35.4, 35.3, 29.8, 29.2, 27.8, 22.8, 21.7, 21.6, 20.6, 19.7, 17.0, 13.7, 13.6. **HRMS** (ESI) *m/z* calculated for $C_{17}H_{31}N_2O_6S^+$ $[M+H]^+$ 391.1897, found 391.1907.

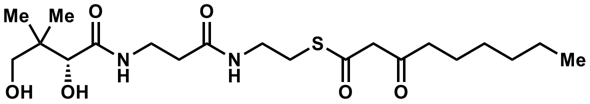

(*R*)-S-(2-(3-(2,4-dihydroxy-3,3-dimethylbutanamido)propanamido)ethyl) 3-oxononanethioate (S10). Method A, 44.2 mg, 41.2% yield, ketone form: enol form = 2.7:1. Pale yellow oil. **^{1}H NMR** (600 MHz, $CDCl_3$) δ 7.39 (s, 1H), 6.64 – 6.38 (m, 1H), 3.99 (s, 1H), $3.70_{\text{ketone form}}$ and $5.45_{\text{enol form}}$ (s, $2H_{\text{total}}$), 3.62 – 3.36 (m, 6H), 3.06 (s, 2H), $2.52_{\text{ketone form}}$ and $2.16_{\text{enol form}}$ (t, J = 7.4 Hz, $2H_{\text{total}}$), 2.43 (t, J = 5.9 Hz, 2H), 1.57 (h, J = 7.3 Hz, 2H), 1.34 – 1.23 (m, 6H), 1.01 (s, 3H), 0.92 (s, 3H), 0.87 (t, J = 6.9 Hz, 3H).**^{13}C NMR** (150 MHz, $CDCl_3$) δ 203.1, 194.2, 192.8, 178.0, 173.8, 173.7, 172.1, 171.9, 99.2, 77.74, 77.73, 71.0, 57.2, 43.8, 40.0, 39.51, 39.48, 39.3, 35.8, 35.7, 35.3, 35.2, 35.1, 31.62, 31.59, 29.3, 28.9, 28.8, 27.8, 26.3, 23.5, 22.60, 22.57, 21.8, 21.7, 20.6, 20.5, 14.2, 14.1. **HRMS** (ESI) *m/z* calculated for $C_{20}H_{37}N_2O_6S^+$ $[M+H]^+$ 433.2367, found 433.2376.

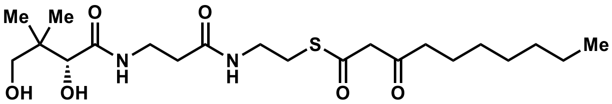

(*R*)-S-(2-(3-(2,4-dihydroxy-3,3-dimethylbutanamido)propanamido)ethyl) 3-oxodecanethioate (S11). Method A, 48.3 mg, 46% yield, ketone form: enol form = 3.4:1. Pale yellow oil. **^{1}H NMR** (600 MHz, $CDCl_3$) δ 7.47 (t, J = 6.2 Hz, 1H), 6.86 – 6.77 (m, 1H), 4.62 – 4.52 (m, 1H), 4.06 – 3.93 (m, 2H), $3.69_{\text{ketone form}}$ and $5.42_{\text{enol form}}$ (s, $2H_{\text{total}}$), 3.57 – 3.31 (m, 6H), 3.09 – 3.01 (m, 2H), $2.50_{\text{ketone form}}$ and $2.14_{\text{enol form}}$ (t, J = 7.4 Hz, $2H_{\text{total}}$), 2.42 (t, J = 6.0 Hz, 2H), 1.62 – 1.50 (m, 2H), 1.25 (s, 8H), 0.97 (s, 3H), 0.89 (s, 3H), 0.85 (t, J = 6.8 Hz, 3H). **^{13}C NMR** (150 MHz, $CDCl_3$) δ 203.0, 194.1, 192.6, 177.8, 174.02, 173.97, 172.1, 171.9, 99.2, 77.48, 77.46, 70.90, 70.86, 57.1, 43.7, 39.9, 39.43, 39.41, 39.3, 39.2, 35.8,

35.7, 35.34, 35.27, 35.0, 31.73, 31.70, 29.16, 29.14, 29.07, 29.0, 27.7, 26.3, 23.5, 22.68, 22.66, 21.54, 21.45, 20.6, 20.5, 14.2. **HRMS** (ESI) *m/z* calculated for $C_{21}H_{39}N_2O_6S^+$ $[M+H]^+$ 447.2523, found 447.2531.

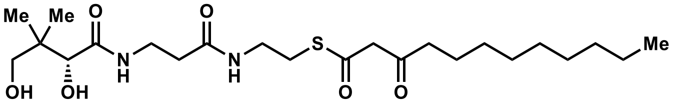

(*R*)-S-(2-(3-(2,4-dihydroxy-3,3-dimethylbutanamido)propanamido)ethyl) 3-oxododecanethioate (S12). Method A, 80.6 mg, 44% yield, ketone form: enol form = 3.2:1. Pale yellow oil. **^{1}H NMR** (600 MHz, $CDCl_3$) δ 7.53 – 7.40 (m, 1H), 6.86 – 6.69 (m, 1H), 4.47 (s, 1H), 3.98 (s, 1H), $3.69_{\text{ketone form}}$ and $5.43_{\text{enol form}}$ (s, $2H_{\text{total}}$), 3.57 – 3.34 (m, 6H), 3.11 – 3.01 (m, 2H), $2.50_{\text{ketone form}}$ and $2.14_{\text{enol form}}$ (t, *J* = 7.4 Hz, 2H), 2.42 (t, *J* = 6.0 Hz, 2H), 1.62 – 1.49 (m, 2H), 1.30 – 1.19 (m, 12H), 0.97 (s, 3H), 0.90 (s, 3H), 0.86 (t, *J* = 7.0 Hz, 3H). **^{13}C NMR** (150 MHz, $CDCl_3$) δ 203.0, 194.1, 192.6, 177.9, 174.0, 173.9, 172.1, 171.9, 99.2, 77.6, 70.93, 70.90, 57.1, 43.7, 39.9, 39.5, 39.2, 35.8, 35.7, 35.33, 35.26, 35.0, 31.9, 29.50, 29.48, 29.44, 29.38, 29.35, 29.34, 29.22, 29.15, 29.1, 27.7, 26.4, 23.5, 22.8, 21.6, 21.5, 20.6, 20.5, 14.2. **HRMS** (ESI) *m/z* calculated for C23H43N2O6S$^+$ $[M+H]^+$ 475.2836, found 475.2841.

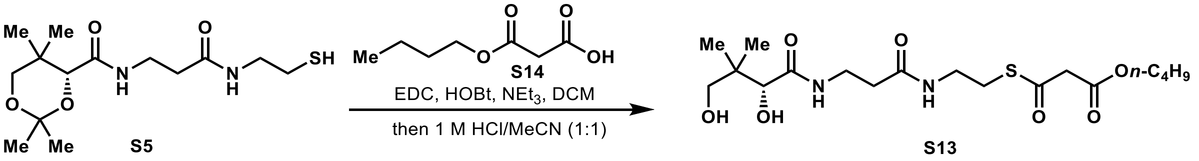

Butyl (*R*)-3-((2-(3-(2,4-dihydroxy-3,3-dimethylbutanamido)propanamido)ethyl)thio)-3-oxopropanoate (S13). To a mixture of **S1** (200 mg, 628 µmol, 1.0 equiv), HOBt (132 mg, 691 µmol, 1.1 equiv) and **S4** (111 mg, 691 µmol, 1.1 equiv) dissolved in dry DCM (3 mL), triethylamine (127 µL, 1.26 mmol, 2 equiv) was added under a nitrogen atmosphere. The solution was cooled to 0 °C and EDC (132 mg, 691 µmol, 1.1 equiv) was added portionwise over 5 min. The reaction mixture was stirred for 15 min at 0 °C and then at room temperature overnight. The solvent was directly evaporated under reduced pressure to yield the crude product. The crude mixture was then purified by column chromatography using DCM:MeOH (20:1). After removal of all the volatiles at reduced pressure, the product was directly dissolved in 5 mL MeCN, and then 5 mL 1 M HCl was added. The mixture was stirred at room temperature for 4 h. After the starting material was consumed (detected by TLC), all the solvent was directly removed under reduced pressure. The crude mixure was then purified by column chromatography using DCM: MeOH (20:1) to give the product **S13** (150 mg, 57% yield). **^{1}H NMR** (600 MHz, $CDCl_3$) δ 7.41 (s, 1H), 6.46 (s, 1H), 4.15 (t, *J* = 6.7 Hz, 2H), 4.01 (s, 1H), 3.62 – 3.48 (m, 12H), 3.45 – 3.37 (m, 1H), 3.20 – 3.03 (m, 2H), 2.43 (t, *J* = 6.0 Hz, 2H), 1.63 (p, 2H), 1.38 (h, *J* = 7.4 Hz, 2H), 1.02 (s, 3H), 0.97 – 0.88 (m, 6H). **^{13}C NMR** (150 MHz, $CDCl_3$) δ 192.1, 173.8, 172.1, 166.6, 77.8, 71.0, 66.1, 49.4, 39.5, 39.3, 35.7, 35.3, 30.6, 29.3, 21.8, 20.6, 19.2, 13.8. **HRMS** (ESI) *m/z* calculated for $C_{18}H_{33}N_2O_7S^+$ $[M+H]^+$ 421.2003, found 421.2012.

II. Plasmids and Proteins

Plasmids: The gene encoding *azaH* (G3XMC2.1) was contained within a modified pET28 vector. To afford the protein with both C- and N-terminal 6xHis-tags. This plasmid is hereby referred to as pAZ83. This was a generous gift from Professor Yi Tang's lab at the University of California, Los Angeles.[5] The plasmid encoding *mrpigD* (AGI63864.1)[4] was synthesized by Twist Biosciences and cloned into a pET-28a(+) vector.

<u>*azaH* Sequence</u>

ATGAGTACAGACTCGATCGAAGTTGCCATTATAGGCGCCGGGATCACGGGAATCACCCTGGCC
CTGGGCCTCCTGTCTCGCGGCATTCCCGTCCGCGTCTACGAGCGAGCCCGCGACTTTCACGAA
ATTGGAGCCGGTATCGGTTTCACCCCCAACGCCGAATGGGCGATGAAAGTCGTCGACCCGCGC
ATTCAAGCTGCTTTCAAACGCGTCGCTACCCCCAATGCCTCCGACTGGTTCCAGTGGGTGGACG
GATTCAACGAGTCCGGTACCGACCCGCGCGAGACCGAGGAACAGCTACTCTTCAAGATCTACCT
CGGCGAGCGTGGATTTGAGGGCTGCCACCGTGCCGACTTCCTAGGTGAGCTGGCACGTCTACT
ACCGGAAGGTGTGGTGACATTCCAGAAGGCGCTGGATACCGTGGAGCCTGCAGCAGATAATAG
CCTCGGCCAGCTTCTTCGATTCCAAGATGGCACGACAGCTACCGCCCACGCGGTGATCGGCTG
CGATGGCATTCGGTCGCGCGTTCGTCAGATCCTCCTAGGTGAAGACCATCCGACAGCATCAGC
CCATTACAGTCATAAATATGCAGCACGCGGCCTTATTCCCATGGACCGCGCCCGGGAGGCGCT
GGGCGAAGATAAAGTGGCGACACGCTTCATGCATCTCGGTCCGGATGCCCATGCCCTGACCTT
CCCCGTTAGCCATGGGTCCTTGTTGAACGTCGTCGCCTTCGTCACGGACCCTAACCCTTGGCCA
TATGCTGATCGCTGGACGGCGCAGGGGCCCAAGAAAGACGTGACGGCTGCCTTTTCCCGCTTT
GGTCCGACCATGCGCACCATAATTGACCTCTTGCCTGATCCTATTGATCAATGGGCCGTTTTTGA
TACATACGACCATCCCCCAAATACGTATTCCCGGGGAGCTGTCTGTATAGCAGGGGATGCTGCT
CATGCCGCGGCTCCGCATCACGGTGCAGGTGCAGGTTGTGGTGTGGAAGACGCGGCTGTGCT
GTGCGCTGTGCTTCATATGGCTGCGAAAAAAGTTAACACCGCAAAAACTGGTTCTGAGGGGAAA
GCCGCTCTTATCACGGCCGCATTCGAAACCTATGATTCGGTTTGTCGCGAGCGTGCGCAGTGGC
TGGTGGAAAGTAGTCGCGTTATCGGTAATCTGTATGAGTGGCAGGATAAGGAGGTAGGGTCGG
ATGCTTCCAGGTGCCACGATGAGGTGTATTGGCGCTCTCATCGCATTTGGGACTATGATATTGAT
GCGATGATGAGAGAGACAGCTGAGGTGTTTGAGGCGCAGGTAGCTGGGGTGGCGAGAAAT

AzaH Protein Sequence
MSTDSIEVAIIGAGITGITLALGLLSRGIPVRVYERARDFHEIGAGIGFTPNAEWAMKVVDPRIQAAFKR
VATPNASDWFQWVDGFNESGTDPRETEEQLLFKIYLGERGFEGCHRADFLGELARLLPEGVVTFQK
ALDTVEPAADNSLGQLLRFQDGTTATAHAVIGCDGIRSRVRQILLGEDHPTASAHYSHKYAARGLIPM
DRAREALGEDKVATRFMHLGPDAHALTFPVSHGSLLNVVAFVTDPNPWPYADRWTAQGPKKDVTA
AFSRFGPTMRTIIDLLPDPIDQWAVFDTYDHPPNTYSRGAVCIAGDAAHAAAPHHGAGAGCGVEDAA
VLCAVLHMAAKKVNTAKTGSEGKAALITAAFETYDSVCRERAQWLVESSRVIGNLCHDEVYWRSHRI
WDYDIDAMMRETAEVFEAQVAGVARN

Non-optimized *mrpigD* Sequence
ATGGAGGATCCTGCTCGGACGCAAGACATCCTTGGACAGCTGCCAATCCTCAAGGCATACAACC
ATATTCTCCTGGGGTTTGCATTGTCCGAGGATATCTCCCGGGAATCCGTCGTCCAAGCGCTCAA
CGCAGCAGCCCTGCAGCTAGCAACGTCCGTCCCATGGATCGGAGGCAAGGTCGTGAACGTGG
GAAGCGGACCCGGCAACACTGGGCTATTCAGGAGCGTGCCATGCGAGCTGTTCGCGCCTCCGA
ATTCGATCCTTCGAGTCAAGGATGTGACGGCGGACTATCCCTCTTACGAGGAGATCGTGAGCGC
CAAAGGCCCCATCTCGATGCTGGACGGCAGCATCATTGCACCCAAGCCTGCATTTCCTGTGAGC
TATGTAGACTGTGAATCCGATCCAGCCCCGGCCCTCCTCATCCAGGCCACTTTCCTCAAGGGTG
GTGTGCTGTTGGACTTCGCCGCGCAGCACAATCTGTCCGACGGCGGCGGCGTCATCCAGATGA
TAAACCTGGTCGCTACCACCCTGCGCGGCGAGAAGATCCCCGAAAAGGCCATAGTACAGGCCA
ATCGAGACCGCAGAGATGTCATCCGGCTTCTGGATCCCGCAGAACCCATGCTGGACCACAGCC
ATCTCGTCCGTCCGCCGCCGTCCGCGATCCCCGCCAACCCCGTCGTCTCGCCCGATAACTTCAT
CTGGCAGTATTTTCGCTTTTCCGCGTCGACGCTGGGTGCGTTGAAGAACATCGCTTCGAATCCG
GCCGACTTCGACCCGTCTGTCAAGTTTATCTCGACGGATGACGCCCTGTGCGCGTTCTTATGGC
AGCGCATCGCGACCGTCCGTCTACGCCGCCGCCAGACGCCGGATGACCTGTGCAAGATGACAC
GGGCCGTCGACATCCGCCGTACTCTCCAGGTGCCATCGGAGTACATGGGCGTGATGGTATACA
ATGTGAGCGGTCGACTGCCTCTCGGCCAACTGGCGACGGCTTCGCTGGCCCGCGCGGCGTCC
GAGTTGCGCAAAGCCCTAAACAGCATCGACGAGTATGCAGTGCGCAGCTTCGCCACATTTGTAG
CGCGCCAGCCGGATAAGTCCACCCTGGCGTACGCGGGGAAGTTCAATCCTGATGTTGACATGG
GGGTATCCTCTATGGCGTCGGTGCCGCTGTATCGCGCCGATTTTGGCCCACTGGGCGCTCCGG
GCCTCGTGCGACGGCCCAATTTTGCACCGGTCCTGAGCACCATCTATGTGATGCCGCAGACTGT

GGAAGGGGACGTTGATGTCTTGATCTGTCTGACGAGGGAGGATATTGGGGCCTTGCGGGCGGA
CCCCGAATGGACTGCGTATGCGGAGTATATAGGGTGATTTACACCATCGAAGAGAGGCAG

MrPigD Protein Sequence
MEDPARTQDILGQLPILKAYNHILLGFALSEDISRESVVQALNAAALQLATSVPWIGGKVVNVGSGPG
NTGLFRSVPCELFAPPNSILRVKDVTADYPSYEEIVSAKGPISMLDGSIIAPKPAFPVSYVDCESDPAP
ALLIQATFLKGGVLLDFAAQHNLSDGGGVIQMINLVATTLRGEKIPEKAIVQANRDRRDVIRLLDPAEP
MLDHSHLVRPPPSAIPANPVVSPDNFIWQYFRFSASTLGALKNIASNPADFDPSVKFISTDDALCAFL
WQRIATVRLRRRQTPDDLCKMTRAVDIRRTLQVPSEYMGVMVYNVSGRLPLGQLATASLARAASEL
RKALNSIDEYAVRSFATFVARQPDKSTLAYAGKFNPDVDMGVSSMASVPLYRADFGPLGAPGLVRR
PNFAPVLSTIYVMPQTVEGDVDVLICLTREDIGALRADPEWTAYAEYIG

III. Protein overexpression and purification

i. Protein overexpression:

Chemically competent *E. coli* into BL21(DE3) cells were transformed with plasmids containing either *azaH* or *mrpigD* using standard heat-shock protocols into chemically competent *E. coli* into BL21(DE3) cells. Overexpression of *azaH* or *mrpigD* was achieved in 1 L of Terrific Broth (TB) media in 2.8 L flasks. 1 L portions of media were inoculated with 10 mL of overnight culture prepared from a single colony in Luria Broth (LB) media supplemented with 50 μg/mL kanamycion (Gold Biotechnology). Cultures were grown at 37 °C and 200 rpm until the optical density at 600 nm reached 0.8 (approx. 3.5 h). The cultures were then cooled to 16 °C for 30 min and protein expression was induced with 0.1 mM isopropyl-β-D-1-thiogalactopyranoside (IPTG, Gold Biotechnology). Expression continued at 16 °C overnight (approx. 18 h) at 200 rpm. The typical yield for 1 L culture was ~25 g cell pellet.

ii. Protein purification procedure:

AzaH purification followed the procedure reported previously by Baker Dockrey et al.[1]
Harvested cell pellets from 1 L overexpression were resuspended in 40 mL of lysis buffer (50 mM Tris-HCl pH 7.4, 300 mM NaCl, 10 mM imidazole, and 10% (v/v) glycerol) containing 1 mg/mL lysozyme, 0.1 mM flavin adenine dinucleotide (FAD), and 1 mM phenylmethylsulfonyl fluoride (PMSF), incubated on a rocker at 4 °C for 45 min, and lysed by sonication for 3 min total in cycles of 10 s on and 30 s off. Insoluble material was removed by centrifugation (40000 x g for 30 min). The clarified lysate was combined with equilibrated Ni-NTA resin (3 mL bed volume) and incubated on a rocker at 4 °C for 1 h. The resin was collected in a gravity-flow column and washed with 50 mL of lysis buffer containing 20 mM imidazole. Enriched His-tagged proteins were eluted from the resin with up to 10 mL lysis buffer containing 400 mM imidazole. The eluted proteins were concentrated and exchanged into storage buffer (SB, 50 mM Tricine pH 7.4, 300 mM NaCl, 10% (v/v) glycerol) using a Cytiva PD-10 desalting column, flash frozen with liquid nitrogen, and stored at −80 °C for future use. Average yields: 40 mg from 1 L AzaH culture. Molecular weights including 6xHis-tags for each protein were estimated by the ProtParam tool on the Expasy server to be 47.6 kDa for AzaH. These molecular weights are consistent with the proteins observed by SDS-PAGE analysis (**Figure S1**).

MrPigD purification:
Harvested cell pellets from 1 L overexpression were resuspended in 40 mL of lysis buffer (50 mM Tris-HCl, 1mM phenylmethylsulfonyl fluoride (PMSF), pH= 7.5) 1 mg/mL. The resuspended cells were homogenized using a handheld Dounce homogenizer. The cells were lysed by sonicating the mixture on ice for 5 min total in cycles of 5 s on and 10 s off. Insoluble material was removed by centrifugation (40000 x g for 30 min). The clarified lysate was combined with equilibrated Ni-NTA resin (3 mL bed volume) and incubated on a rocker at 4 °C for 1 h. The resin was collected in a gravity-flow column and washed with 30 mL of lysis buffer containing 10 mM imidazole. Enriched His-tagged proteins were

eluted from the resin with up to 10 mL lysis buffer containing 250 mM imidazole. The eluted proteins were concentrated and exchanged into storage buffer (SB, 25 mM Tricine pH 8.0, 100 mM NaCl, 10% (v/v) glycerol) using a Cytiva PD-10 desalting column. The eluted protein was then concentrated to 1.7 mM (determined by Pierce assay) using a 30,000 NMWL Amicon Ultra-15 centrifugal filter. Average yields: 80 mg from 1 L MrPigD culture. Molecular weights including 6xHis-tags for each protein were estimated by the ProtParam tool on the Expasy server to be 50.2 kDa for MrPigD. These molecular weight was consistent with the protein observed by SDS-PAGE analysis (**Figure S1**).

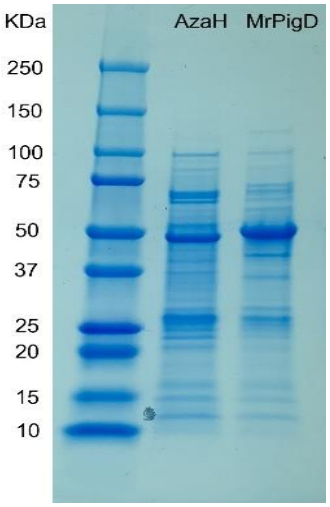

Supplemental Figure 1. Purified AzaH and MrPigD. Approximately 2 µL of 20 µM each protein was loaded onto a 12.5% SDS-PAGE gel. The gel was stained with Quick Coomassie stain (Anatrace) and visualized with the Azure Gel Imaging System. The relative apparent masses are consistent with the predicted estimates. Quick Coomassie stain (Anatrace) and visualized with the Azure Gel Imaging System. The relative apparent masses are consistent with the predicted estimates.

IV. Biocatalytic reactions

Stock solutions: Stock solutions of each orcinaldehyde substrate (50 mM), each pantetheine thioester substrate (100 mM) and maleimide (1 M) were prepared by dissolving the substrate in DMSO (analytical grade) and stored at -20 °C. Stock solutions of $NADP^+$ (100 mM) and glucose-6-phosphate (G6P, 500mM) were stored at -20 °C. Aliquots of each enzyme and glucose-6-phosphate dehydrogenase (G6PDH, 100 U/mL) were stored at -80 °C.

Analytical-scale reactions: Each reaction contained 2.5 mM of the orcinaldehyde substrate (5 uL of a 50 mM stock solution in DMSO), 5 mM G6P (1 µL, 500 mM), 1 mM $NADP^+$ (1 µL, 100 mM), 1 U/mL G6P-DH (1 µL, 100 U/mL), 10 µM AzaH (6.6 µL of a 150 µM stock solution), and 76.6 µL of a 50 mM potassium phosphate buffer, pH 8.0. The dearomatization reaction was carried out at 30 °C for 1 h. Following this, 400 µL of 50 mM potassium phosphate buffer added (5 times dilute), 0.55 mM thioester **18** (2.8 µL of a 100 mM stock solution in DMSO) and 20 µM MrPigD (6 µL of a 1.7 mM stock solution) were then added, and the second reaction was carried out at 30 °C for 1 h. The reactions were quenched with 1 mL of MeOH and the precipitated biomolecules were pelleted by centrifugation (16,000 x g, 12 min). The supernatant was analyzed by UPLC-DAD and conversion obtained by comparison to calibration curves of each substrate unless full conversion was observed.

General procedure for preparative scale *in vitro* reactions (8 mL scale):
2.5 mM orcinaldehyde substrate (400 µL of a 50 mM stock solution in DMSO), 5 mM G6P (80 µL, 500 mM), 1 mM NADP+ (80 µL, 100 mM), 1 U/mL G6P-DH (80 µL, 100 U/mL), 10 µM AzaH (535 µL of a 150 µM stock solution) was added to 6.51 mL of 50 mM potassium phosphate buffer, pH 8.0. The reaction was placed in an incubator at 30 °C with 100 rpm shaking. After 1 h, 2.75 mM thioester **18** (220 µL of a 100 mM stock solution in DMSO), 5 mM maleimide (thiol scavenger for substrate **1e** only, 40 µL 1 M stock solution in DMSO) and 20 µM MrPigD (95 µL of a 1.7 mM stock solution) were then added, and the reaction was incubated at 30 °C for 25 min with 100 rpm shaking. The reaction was then diluted with brine (~3x reaction volume) and extracted 3 times with ~10 mL EtOAc (centrifuge if necessary). The combined organic layers were washed with ~30 mL of brine, then dried with sodium

sulfate. The sodium sulfate and precipitated biomolecules were removed through gravity filtration and the flow-through was passed through a 0.22 μm filter before the solvent was removed using a rotary evaporator. The resulting crude oil was dissolved in 1 mL of HPLC grade MeCN and subjected to purification by preparative HPLC using a Phenomenex Kinetex 5 μm C18, 150 x 21.2 mm column under the following conditions: mobile phase A = deionized water and B = acetonitrile; method = 5% B hold 1 min,5% to 20% B over 2 min, 20% to 50% B over 5 min, 50% to 90% B over 9 min, 90% to 100% B over 5 min, 100% B for 3 min; flow rate, 10 mL/min. Fractions containing the purified compound were pooled and the solvent was removed using a rotary evaporator.

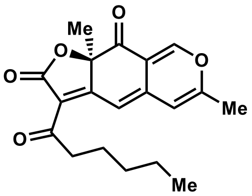

(*R*)-3-hexanoyl-6,9a-dimethyl-2H-furo[3,2-g]isochromene-2,9(9aH)-dione (32). Following preparation by the general method described above, the title compound was isolated as an orange, amorphous solid (1.9 mg, 27% yield over two steps). **^{1}H NMR** (600 MHz, $CDCl_3$) δ 7.84 (s, 1H), 6.81 (s, 1H), 6.16 (s, 1H), 2.99 – 2.88 (m, 2H), 2.21 (s, 3H), 1.70 (s, 3H), 1.63 – 1.60 (m, 2H), 1.33 – 1.30 (m, 4H), 0.88 (t, *J* = 7.1 Hz, 3H). **^{13}C NMR** (150 MHz, $CDCl_3$) δ 197.6, 191.1, 172.8, 169.4, 160.0, 153.3, 141.6, 116.7, 113.6, 110.5, 103.2, 86.0, 41.8, 31.5, 28.4, 23.5, 22.7, 19.6, 14.1. **HRMS** (ESI) *m/z* calculated for C19H20NaO5^{+} $[M+Na]^{+}$ 351.1203, found 351.1213.

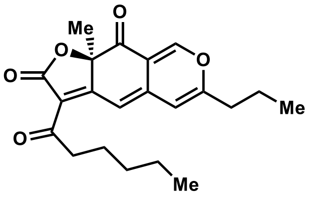

(*R*)-3-hexanoyl-9a-methyl-6-propyl-2H-furo[3,2-g]isochromene-2,9(9aH)-dione (19). Following preparation by the general method described above, the title compound was isolated as an orange, amorphous solid (3.0 mg, 39% yield over two steps). **^{1}H NMR** (600 MHz, $CDCl_3$) δ 7.85 (d, *J* = 0.6 Hz, 1H), 6.82 (d, *J* = 1.2 Hz, 1H), 6.15 (s, 1H), 2.99 – 2.88 (m, 2H), 2.41 (t, *J* = 7.5 Hz, 2H), 1.71 (s, 3H), 1.70 – 1.64 (m, 4H), 1.34 – 1.30 (m, 4H), 1.00 (t, *J* = 7.4 Hz, 3H), 0.88 (t, *J* = 6.9 Hz, 3H). **^{13}C NMR** (150 MHz, $CDCl_3$) δ 197.6, 191.2, 172.3, 169.4, 163.6, 153.5, 141.7, 116.8, 113.4, 109.9, 103.24, 86.0, 41.8, 35.3, 31.5, 28.4, 23.6, 22.7, 20.1, 14.1, 13.6. **HRMS** (ESI) *m/z* calculated for C21H24NaO5^{+} $[M+Na]^{+}$ 379.1516, found 379.1526.

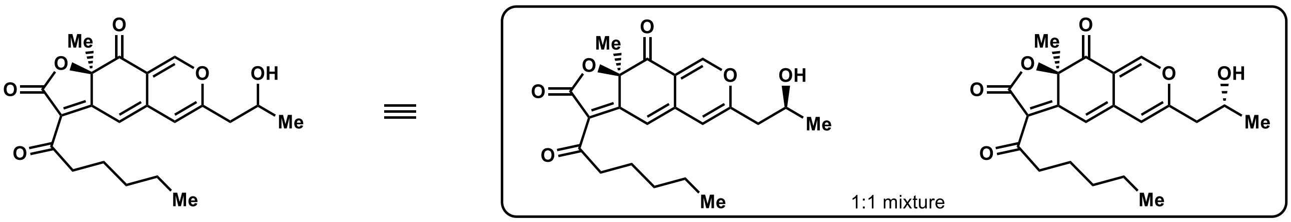

(*9aR*)-3-hexanoyl-6-(2-hydroxypropyl)-9a-methyl-2H-furo[3,2-g]isochromene-2,9(9aH)-dione (33). Following preparation by the general method described above, the title compound was isolated as an orange, amorphous solid, mixture of a 1:1 ratio two diastereoisomers (3.0 mg, 37% yield over two steps). **^{1}H NMR** (600 MHz, ^{1}H NMR (599 MHz, Chloroform-*d*) δ 7.85 (s, 1H), 6.84 (s, 1H), 6.26 and 6.25 (s, $0.5H_{each}$, 1H $_{total}$), 4.23 – 4.15 (m, 1H), 2.98 – 2.88 (m, 2H), 2.60 – 2.52 (m, 2H), 1.712 and

1.170 (s,1.5H$_{each}$, 3H$_{total}$), 1.64 – 1.59 (m, 2H), 1.34 – 1.30 (m, 7H), 0.88 (t, *J* = 7.1 Hz, 3H). **^{13}C NMR** (150 MHz, $CDCl_3$) δ 197.6, 191.0, 172.0, 169.3, 160.5, 160.4, 153.3, 141.10, 141.08, 116.83, 116.81, 113.8, 111.8, 111.7, 103.72, 103.69, 86.1, 65.7, 65.6, 42.8, 42.7, 41.8, 31.5, 28.3, 23.90, 23.87, 23.53, 23.51, 22.7, 14.1. **HRMS** (ESI) *m/z* calculated for $C21H24NaO6^+$ $[M+Na]^+$ 395.1465, found 395.1467.

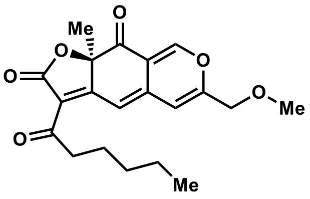

(*R*)-3-hexanoyl-6-(methoxymethyl)-9a-methyl-2H-furo[3,2-g]isochromene-2,9(9aH)-dione (34). Following preparation by the general method described above, the title compound was isolated as an orange, amorphous solid (2.3 mg, 32% yield over two steps). **^{1}H NMR** (600 MHz, $CDCl_3$) δ 7.83 (s, 1H), 6.89 (s, 1H), 6.40 (s, 1H), 4.15 (s, 2H), 3.47 (s, 3H), 3.00 – 2.89 (m, 2H), 1.71 (s, 3H), 1.62 (d, *J* = 7.2 Hz, 3H), 1.35 – 1.31 (m, 4H), 0.89 (t, *J* = 6.3 Hz, 3H). **^{13}C NMR** (150 MHz, $CDCl_3$) δ 197.5, 190.8, 171.7, 169.2, 158.5, 153.0, 140.2, 117.0, 114.4, 110.4, 104.6, 86.1, 70.0, 59.5, 41.8, 31.5, 28.2, 23.5, 22.7, 14.1. **HRMS** (ESI) *m/z* calculated for $C20H22NaO6^+$ $[M+Na]^+$ 381.1309, found 381.1301.

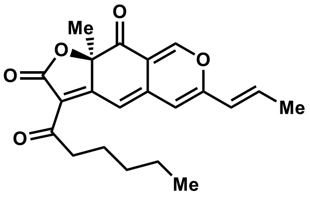

Rubropunctatin (8). Following preparation by the general method described above, the title compound was isolated as an orange, amorphous solid (2.3 mg, 30% yield over two steps). **^{1}H NMR** (600 MHz, $CDCl_3$) ^{1}H NMR (599 MHz, Chloroform-*d*) δ 7.86 (s, 1H), 6.89 (s, 1H), 6.59 (dd, *J* = 15.5, 7.0 Hz, 1H), 6.14 (s, 1H), 6.07 – 6.01 (m, 1H), 2.99 – 2.89 (m, 2H), 1.97 – 1.94 (m, 3H), 1.71 (s, 3H), 1.64 – 1.59 (m, 2H), 1.32 (dt, *J* = 7.6, 3.7 Hz, 4H), 0.88 (t, *J* = 6.9 Hz, 3H). **^{13}C NMR** (150 MHz, $CDCl_3$) δ 197.6, 191.0, 171.8, 169.4, 156.6, 152.9, 141.7, 136.5, 122.5, 116.5, 113.4, 109.7, 104.3, 85.9, 41.8, 31.6, 28.5, 23.6, 22.7, 18.9, 14.1. **HRMS** (ESI) *m/z* calculated for $C21H22NaO5^+$ $[M+Na]^+$ 377.1359, found 377.1364.

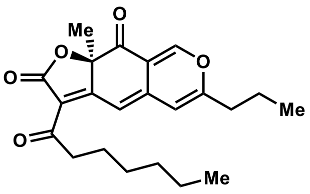

(*R*)-3-heptanoyl-9a-methyl-6-propyl-2H-furo[3,2-g]isochromene-2,9(9aH)-dione (37). Following preparation by the general method described above, the title compound was isolated as an orange, amorphous solid (2.2 mg, 29% yield over two steps). **^{1}H NMR** (600 MHz, $CDCl_3$) δ 7.85 (s, 1H), 6.82 (s, 1H), 6.15 (s, 1H), 2.99 – 2.88 (m, 2H), 2.41 (t, *J* = 7.5 Hz, 2H), 1.71 (s, 3H), 1.70 – 1.60 (m, 4H), 1.35 – 1.28 (m, 6H), 1.00 (t, *J* = 7.4 Hz, 3H), 0.87 (t, *J* = 6.7 Hz, 3H). **^{13}C NMR** (150 MHz, $CDCl_3$) δ 197.6, 191.2, 172.2, 169.4, 163.6, 153.5, 141.7, 116.8, 113.4, 109.9, 103.2, 86.0, 41.8, 35.3, 31.9, 29.1, 28.4, 23.8, 22.7, 20.1, 14.2, 13.6. **HRMS** (ESI) *m/z* calculated for $C22H27O5^+$ $[M+Na]^+$ 371.1853, found 371.1868.

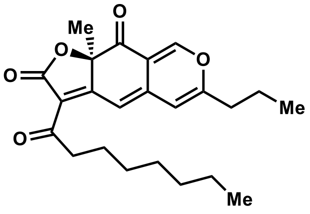

(*R*)-9a-methyl-3-octanoyl-6-propyl-2H-furo[3,2-g]isochromene-2,9(9aH)-dione (38). Following preparation by the general method described above, the title compound was isolated as an orange, amorphous solid (2.1 mg, 26% yield over two steps). **^{1}H NMR** (600 MHz, $CDCl_3$) δ 7.85 (s, 1H), 6.82 (s, 1H), 6.15 (s, 1H), 2.99 – 2.88 (m, 2H), 2.42 (t, *J* = 7.5 Hz, 2H), 1.71 (s, 3H), 1.69 – 1.62 (m, 4H), 1.33 – 1.27 (m, 8H), 1.00 (t, *J* = 7.4 Hz, 3H), 0.87 (t, *J* = 6.9 Hz, 3H). **^{13}C NMR** (150 MHz, $CDCl_3$) δ 197.6, 191.2, 172.2, 169.4, 163.6, 153.5, 141.7, 116.8, 113.4, 109.9, 103.2, 86.0, 41.8, 35.3, 31.9, 29.34, 29.33, 28.4, 23.9, 22.8, 20.1, 14.3, 13.6. **HRMS** (ESI) *m/z* calculated for $C23H28NaO5^+$ $[M+Na]^+$ 407.1829, found 407.1837.

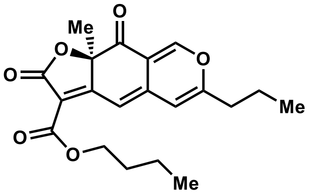

Butyl (*R*)-9a-methyl-2,9-dioxo-6-propyl-9,9a-dihydro-2H-furo[3,2-g]isochromene-3-carboxylate (44). Following preparation by the general method described above, however before the EtOAc extraction, 0.8 mL triethylamine was added and stirred for 30 min. The title compound was isolated as an orange, amorphous solid (2.0 mg, 28% yield over two steps). **^{1}H NMR** (600 MHz, $CDCl_3$) δ 7.84 (s, 1H), 6.55 (s, 1H), 6.13 (s, 1H), 4.27 (td, *J* = 6.8, 3.1 Hz, 2H), 2.41 (t, *J* = 7.5 Hz, 2H), 1.75 – 1.72 (m, 2H), 1.71 (s, 3H), 1.68 –1.65 (m, 2H), 1.47 – 1.41 (m, 2H), 1.00 (t, *J* = 7.4 Hz, 3H), 0.95 (t, *J* = 7.4 Hz, 3H). **^{13}C NMR** (150 MHz, $CDCl_3$) δ 190.9, 172.7, 167.1, 163.3, 162.3, 153.4, 140.3, 116.5, 109.6, 107.1, 102.2, 85.9, 64.9, 35.2, 30.8, 28.2, 20.1, 19.3, 13.9, 13.6. **HRMS** (ESI) *m/z* calculated for $C20H22NaO6^+$ $[M+Na]^+$ 381.1309, found 381.1304.

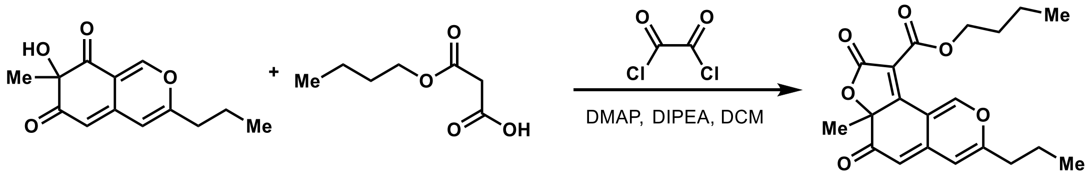

Butyl (±)-6a-methyl-6,8-dioxo-3-propyl-6a,8-dihydro-6H-furo[2,3-h]isochromene-9-carboxylate (46). The acid in 3.0 mL of anhydrous dichloromethane was cooled to 0 ^oC under argon, and oxalyl chloride (85 µL, 0.97 mmol) as well as one drop of anhydrous DMF was added. The reaction mixture was stirred for 15 min at 0 ^oC then warmed to rt for 1.5 h. Then the solvent and extra oxalyl chloride was removed by vacuum. After that, the mixture was dissolved in DCM (2 mL) and cooled to 0 ^oC. To this mixture, another solution of Diisopropylethylamine (260 µL, 1.5 mmol), azaphilone (±)-**29** (35 mg, 0.15 mmol), and 4-dimethylaminopyridine in 1 mL dichloromethane was added. The reaction mixture was slowly warmed to rt and stirred until full disappearance of azaphilone (±)-**29** by TLC analysis. The reaction mixture was quenched with water and extracted with ethyl acetate. The organic layer was

separated, sequentially washed with brine, dried over anhydrous Na_2SO_4, filtered, and concentrated *in vacuo*. Purification on silica gel (hexane : EtOAc = 4:1 to 1:1) provided 20 mg (37%) of product (±)-**46**.

Following preparation by the method described above, the title compound was isolated as an orange, solid, M.P. 107.5-108.2 °C. 20 mg, 37% yield. **^{1}H NMR** (600 MHz, $CDCl_3$) δ 8.56 (s, 1H), 6.08 (s, 1H), 5.29 (s, 1H), 4.31 (dt, *J* = 10.8, 6.7 Hz, 1H), 4.24 (dt, *J* = 10.8, 6.7 Hz, 1H), 2.42 (t, *J* = 7.5 Hz, 2H), 1.74 – 1.65 (m, 7H), 1.44 (h, *J* = 7.3 Hz, 2H), 1.01 (t, *J* = 7.4 Hz, 3H), 0.94 (t, *J* = 7.4 Hz, 3H). **^{13}C NMR** (150 MHz, $CDCl_3$) δ 1 ^{13}C NMR (151 MHz, $CDCl_3$) δ 190.3, 166.1, 166.0, 162.3, 161.5, 153.5, 143.9, 116.8, 111.1, 108.0, 105.4, 87.3, 66.2, 35.3, 30.6, 26.3, 20.0, 19.2, 13.8, 13.6. **HRMS** (ESI) *m/z* calculated for C20H22NaO6$^+$ [M+Na]$^+$ 381.1309, found 381.1311.

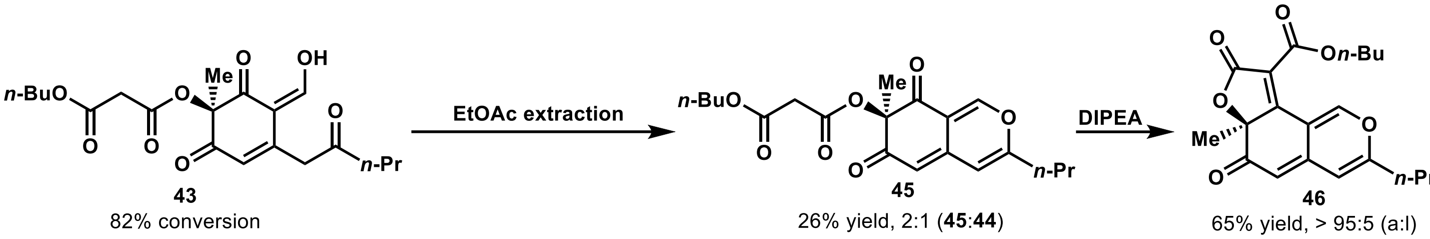

Following preparation by the general method described above before the Prep HPLC purification, a mixture of 45 and 44 was obtained in 2:1 ratio determined by NMR with CH_2Br_2 as internal standard. To the mixture, 1mL of 10% DIPEA in DCM was added and stirred for 30 min under room temperature. After the reaction was finished (determined by LCMS), all the solvent was removed by rotary evaporation. Then the product 46 yield was determined by NMR with CH_2Br_2 as internal standard.

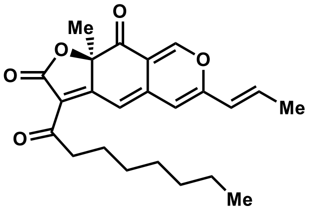

Monascorubrin (9). Following preparation by the general method described above, the title compound was isolated as an orange, amorphous solid (1.5 mg, 19% yield over two steps). **^{1}H NMR** (600 MHz, $CDCl_3$) δ 7.86 (s, 1H), 6.89 (s, 1H), 6.59 (dd, *J* = 15.5, 7.0 Hz, 1H), 6.14 (s, 1H), 6.08 – 6.01 (m, 1H), 3.02 – 2.87 (m, 2H), 1.95 (dd, *J* = 7.0, 1.2 Hz, 4H), 1.71 (s, 3H), 1.63 – 1.59 (m, 2H), 1.31 – 1.25 (m, 8H), 0.87 (d, *J* = 6.8 Hz, 3H). **^{13}C NMR** (150 MHz, $CDCl_3$) δ 197.6, 191.0, 171.8, 169.4, 156.5, 152.9, 141.7, 136.5, 122.5, 116.5, 113.4, 109.7, 104.3, 85.9, 41.8, 31.9, 29.4, 29.3, 28.46, 23.9, 22.8, 18.9, 14.3. **HRMS** (ESI) *m/z* calculated for C23H26NaO5$^+$ [M+Na]$^+$ 405.1672, found 405.1677.

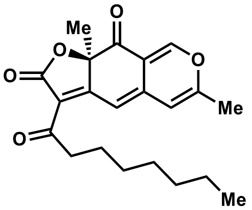

(*R*)-6,9a-dimethyl-3-octanoyl-2H-furo[3,2-g]isochromene-2,9(9aH)-dione (41). Following preparation by the general method described above, the title compound was isolated as an orange, amorphous solid (1.8 mg, 25% yield over two steps). **^{1}H NMR** (600 MHz, $CDCl_3$) δ 7.84 (s, 1H), 6.81 (s, 1H), 6.16 (s, 1H), 3.00 – 2.88 (m, 2H), 2.21 (s, 3H), 1.70 (s, 3H), 1.64 – 1.58 (m, 2H), 1.34 – 1.24 (m, 8H), 0.87 (t, *J* = 7.0 Hz, 3H). **^{13}C NMR** (150 MHz, $CDCl_3$) δ 197.6, 191.1, 172.7, 169.4, 160.0, 153.3,

141.6, 116.7, 113.6, 110.5, 103.2, 86.0, 41.8, 31.9, 29.33, 29.32, 28.4, 23.8, 22.8, 19.6, 14.2. **HRMS** (ESI) *m/z* calculated for C21H24NaO5$^+$ [M+Na]$^+$ 379.1516, found 379.1524.

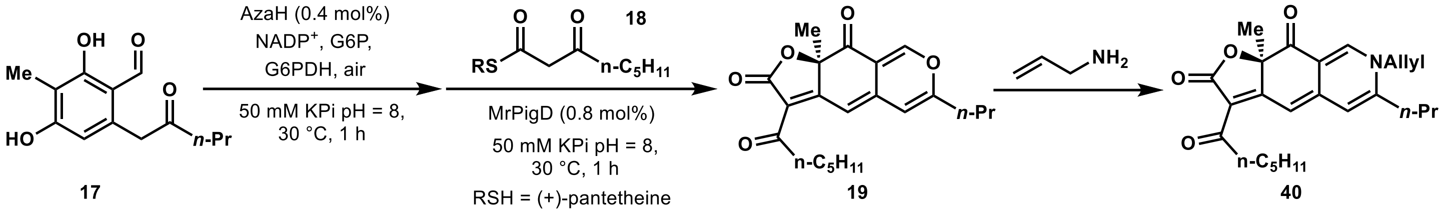

2.5 mM orcinaldehyde substrate (250 uL of a 50 mM stock solution in DMSO), 5 mM G6P (50 µL, 500 mM), 1 mM NADP+ (50 µL, 100 mM), 1 U/mL G6P-DH (50 µL, 100 U/mL), 10 µM AzaH (333 µL of a 150 µM stock solution) was added to 4.13 mL of 50 mM potassium phosphate buffer, pH 8.0. The reaction was placed in an incubator at 30 °C with 85 rpm shaking. After 1 h, the 2.5 mM thioester **18** (125 µL of a 100 mM stock solution in DMSO) and 20 µM MrPigD (12 µL of a 1.7 mM stock solution) were then added, and the reaction was returned to the 30 °C for 1 h with shaking. Allylic amine was added to the mixture, and the reaction was then diluted with brine (~3x reaction volume) and extracted 3 times with ~10 mL EtOAc. The combined organic layers were washed with ~30 mL of brine, then dried with sodium sulfate. The sodium sulfate and precipitated biomolecules were removed through gravity filtration and the flow-through was passed through a 0.22 µm filter before the solvent was removed using a rotary evaporator. The resulting crude oil was dissolved in ~500 µL of HPLC grade MeCN and subjected to purification by preparative HPLC using a Phenomenex Kinetex 5 µm C18, 150 x 21.2 mm column under the following conditions: mobile phase A = deionized water and B = acetonitrile; method = 5% B hold 1 min,5% to 20% B over 2 min, 20% to 50% B over 5 min, 50% to 90% B over 9 min, 90% to 100% B over 5 min, 100% B for 3 min; flow rate, 10 mL/min. Fractions containing the purified compound were pooled and the solvent was removed using a rotary evaporator give product 3.4 mg (69% yield) **40**.

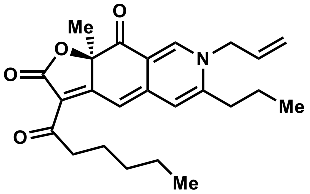

(*R*)-7-allyl-3-hexanoyl-9a-methyl-6-propylfuro[3,2-g]isoquinoline-2,9(7H,9aH)-dione (40). Following preparation by the general method described above, the title compound was isolated as a red, amorphous solid (3.0 mg, 69% yield over three steps). **^{1}H NMR** (600 MHz, CDCl$_3$) δ 7.75 (s, 1H), 6.71 (s, 1H), 6.46 (s, 1H), 5.93 (ddt, *J* = 17.0, 10.2, 5.0 Hz, 1H), 5.43 (d, *J* = 10.4 Hz, 1H), 5.18 (dt, *J* = 17.1, 1.6 Hz, 1H), 4.45 (d, *J* = 4.9 Hz, 2H), 2.95 – 2.89 (m, 2H), 2.53 – 2.48 (m, 2H), 1.70 – 1.67 (m, 5H), 1.64 – 1.61 (m, 2H), 1.35 – 1.31 (m, 4H), 1.06 (t, *J* = 7.3 Hz, 3H), 0.88 (t, *J* = 7.0 Hz, 3H). **^{13}C NMR** (150 MHz, CDCl$_3$) δ 197.7, 194.4, 173.8, 170.8, 150.7, 149.1, 141.7, 130.9, 120.2, 118.3, 117.6, 106.39, 97.7, 85.7, 55.5, 41.0, 33.7, 31.8, 30.1, 24.2, 22.8, 21.6, 14.2, 13.8. **HRMS** (ESI) *m/z* calculated for C24H30NO4$^+$ [M+H]$^+$ 396.2169, found 396.2172.

V. Synthesis to support mechanistic insights.

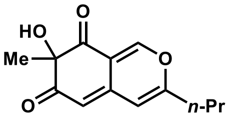

(±)-7-hydroxy-7-methyl-3-propyl-6H-isochromene-6,8(7H)-dione, (±)-29.

Prepared as previously reported by Baker Dockrey et al.[1] ^{1}H NMR (600 MHz, $CDCl_3$) δ 7.88 (s, 1H), 6.11 (s, 1H), 5.51 (s, 1H), 3.89 (s, 1H), 2.40 (t, J = 7.5 Hz, 2H), 1.67 (h, J = 7.4 Hz, 2H), 1.54 (s, 3H), 0.99 (t, J = 7.4 Hz, 3H). spectra obtained were consistent with literature values.[1]

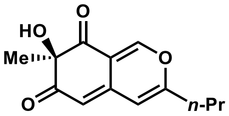

(*R*)-7-hydroxy-7-methyl-3-propyl-6H-isochromene-6,8(7H)-dione (29).
Prepared as previously reported by Baker Dockrey et al.[1]

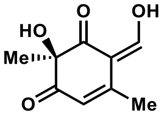

(*R*,*Z*)-2-hydroxy-6-(hydroxymethylene)-2,5-dimethylcyclohex-4-ene-1,3-dione (S16).
Prepared as previously reported by Baker Dockrey et al.[1]

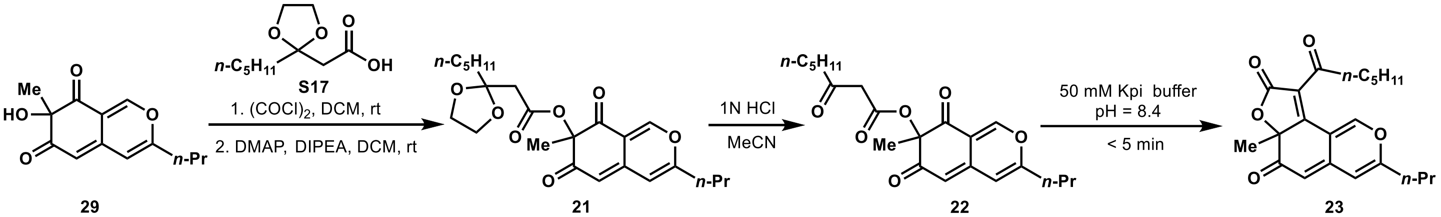

7-methyl-6,8-dioxo-3-propyl-7,8-dihydro-6H-isochromen-7-yl 2-(2-pentyl-1,3-dioxolan-2-yl)acetate, (±)-21. Acid **S17** in 5.0 mL of anhydrous dichloromethane was cooled to 0 ^{o}C under argon and oxalyl chloride (100 µL, 1.2 mmol) as well as one drop of anhydrous DMF was added. The reaction mixture was stirred for 15 min at 0 ^{o}C and then warmed to rt for 1.5 h. Then the solvent and extra oxalyl chloride was removed by vacuum. After that, the mixture was dissolved in DCM (5 mL) and cooled to 0 ^{o}C. To this mixture, another solution of diisopropylethylamine (480 µL, 2.7 mmol), azaphilone (±)-**29** (80 mg, 0.34 mmol) and 4-dimethylaminopyridine in 1 mL dichloromethane was added. The reaction mixture was slowly warmed to rt and stirred until full disappearance of azaphilone (±)-**29** by TLC analysis. The reaction mixture was quenched with water and extracted with ethyl acetate. The organic layer was separated, sequentially washed with brine, dried over anhydrous Na_2SO_4, filtered, and concentrated in vacuo. Purification on silica gel (hexane : EtOAc = 4:1 to 1:1) provided 84 mg (59% yield) of product **21**. **^{1}H NMR** (600 MHz, $CDCl_3$) δ 7.85 (s, 1H), 6.06 (s, 1H), 5.49 (s, 1H), 4.05 – 3.99 (m, 2H), 3.97 – 3.92 (m, 2H), 2.78 (d, J_{AB} = 14.3 Hz, 1H), 2.73 (d, J_{BA} = 14.3 Hz, 1H), 2.36 (t, J = 7.5 Hz, 2H), 1.85 – 1.78 (m, 2H), 1.64 (h, J = 7.4 Hz, 2H), 1.52 (s, 3H), 1.42 – 1.36 (m, 2H), 1.31 – 1.25 (m, 4H), 0.97 (t, J = 7.4 Hz, 3H), 0.86 (t, J = 6.8 Hz, 3H). **^{13}C NMR** (150 MHz, $CDCl_3$) δ 193.2, 192.6, 168.8, 162.2, 154.1, 142.8, 115.3, 109.5, 108.8, 107.0, 84.4, 65.3, 65.3, 41.4, 37.8, 35.1, 32.0, 23.3, 22.7, 22.3, 20.0, 14.1, 13.5. **HRMS** (ESI) *m/z* calculated for C23H31O7$^+$ [M+H]$^+$ 419.2064, found 419.2067.

To a solution of **5** (72.5 mg, 173 µmol) in MeCN (5 mL), 5 mL of 1 N HCl was added. Then, the mixture was heated to 50 °C for 12 h. Monitored by LC-MS, the starting material was consumed to afford **22** which was isolated by preparative HPLC using a Phenomenex Kinetex 5 µm C18, 150 x 21.2 mm column under the following conditions: mobile phase A = deionized water and B = acetonitrile; method = 5% to 20% B over 4 min, 20% to 50% B over 9 min, 50% to 100% B over 2 min, 100% B for 3 min; flow rate, 10 mL/min. Fractions containing the purified compound were pooled and the solvent was removed using a rotary evaporator. **22** was formed as the major product and a small amount of

product **23** was also detected. Then, all solvent was removed and a mixture of acetonitrile/water (1:1, 15 mL) was added. After 1 M KPi pH 8.0 buffer (0.75 mL) was added which afforded the product within 5 min. The product was extracted with ethyl acetate and the organic layer was separated. The combined organic layers sequentially washed with brine, dried over anhydrous Na_2SO_4, filtered, and concentrated *in vacuo*. Purification on silica gel (hexane : EtOAc = 4:1 to 1:1) provided 25.6 mg (41% yield over two steps) of product **23**.

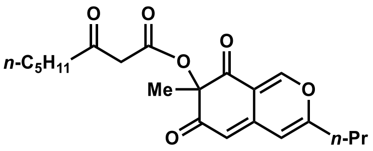

7-methyl-6,8-dioxo-3-propyl-7,8-dihydro-6H-isochromen-7-yl 3-oxooctanoate, (±)-22. ^{1}H NMR (600 MHz, $CDCl_3$) δ 7.88 (s, 1H), 6.10 (s, 1H), 5.51 (d, *J* = 0.6 Hz, 1H), 3.51 (d, *J* = 1.6 Hz, 2H), 2.63 (t, *J* = 7.2 Hz, 2H), 2.39 (t, *J* = 7.5 Hz, 2H), 1.68 – 1.64 (m, 2H), 1.62 – 1.59 (m, 2H), 1.55 (s, 3H), 1.33 – 1.26 (m, 4H), 1.00 (t, *J* = 7.4 Hz, 3H), 0.88 (t, *J* = 7.0 Hz, 3H). **^{13}C NMR** (150 MHz, $CDCl_3$) δ 202.1, 192.8, 192.1, 166.5, 162.5, 154.3, 143.0, 115.3, 108.9, 106.8, 85.5, 48.6, 42.6, 35.2, 31.3, 23.2, 22.6, 22.5, 20.1, 14.1, 13.6. **HRMS** (ESI) *m/z* calculated for $C21H27O6^+$ $[M+H]^+$ 375.1802, found 375.1803.

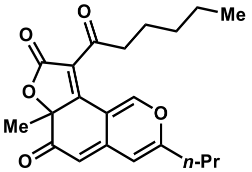

9-hexanoyl-6a-methyl-3-propyl-6H-furo[2,3-h]isochromene-6,8(6aH)-dione, (±)-23. ^{1}H NMR (600 MHz, $CDCl_3$) δ 8.78 (s, 1H), 6.07 (s, 1H), 5.28 (d, *J* = 1.2 Hz, 1H), 3.16 (ddd, *J* = 18.1_{AB}, 8.8, 6.1 Hz, 1H), 2.81 (ddd, *J* = 18.1_{BA}, 8.8, 5.7 Hz, 1H), 2.41 (td, *J* = 7.4, 2.4 Hz, 2H), 1.72 – 1.65 (m, 5H), 1.65 – 1.52 (m, 2H), 1.33 – 1.25 (m, 4H), 1.00 (t, *J* = 7.4 Hz, 3H), 0.86 (t, *J* = 7.0 Hz, 3H). **^{13}C NMR** (150 MHz, $CDCl_3$) δ 197.3, 190.4, 168.2, 165.5, 162.4, 153.6, 144.3, 123.6, 111.3, 107.9, 105.1, 87.8, 42.2, 35.2, 31.3, 26.3, 23.2, 22.6, 20.0, 14.0, 13.6. **HRMS** (ESI) *m/z* calculated for $C21H25O5^+$ $[M+H]^+$ 357.1697, found 357.1704.

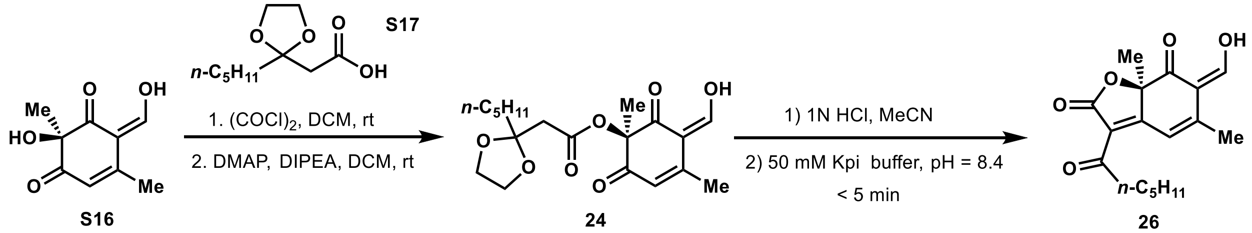

(*Z*)-5-(hydroxymethylene)-1,4-dimethyl-2,6-dioxocyclohex-3-en-1-yl 2-(2-pentyl-1,3-dioxolan-2-yl)acetate (24). The acid in 5.0 mL of anhydrous dichloromethane was cooled to 0 °C under argon, and oxalyl chloride (100 µL, 1.2 mmol) as well as one drop of anhydrous DMF was added. The reaction mixture was stirred for 15 min at 0 °C and warmed to rt for 1.5 h. Then the solvent and extra oxalyl chloride was removed by vacuum. After that, the mixture was dissolved in DCM (5 mL) and cooled to 0 °C. To this mixture, another solution of diisopropylethylamine (356 µL, 2.0 mmol), azaphilone **16** (46 mg, 0.25 mmol) and 4-dimethylaminopyridine in 1 mL dichloromethane was added. The reaction mixture was slowly warmed to rt and stirred until full disappearance of azaphilone **16** by TLC analysis. The reaction mixture was quenched with water and extracted with ethyl acetate. The organic layer was separated, sequentially washed with brine, dried over anhydrous Na_2SO_4, filtered, and concentrated in vacuo. Purification on silica gel (hexane : EtOAc = 1:1 to 1:2) provided 39.2 mg (42% yield) of product **24**. **^{1}H NMR** (600 MHz, $CDCl_3$) δ 14.53 (s, 1H), 7.93 (s, 1H), 5.87 (s, 1H), 4.04 – 4.00 (m, 2H), 3.98 –

3.94 (m, 2H), 2.80 (d, J_{AB} = 14.3 Hz, 1H), 2.76 (d, J_{BA} = 14.3 Hz, 1H), 2.14 (s, 3H), 1.82 – 1.77 (m, 2H), 1.57 (s, 3H), 1.42 – 1.38 (m, 2H), 1.32 – 1.28 (m, 4H), 0.88 (t, *J* = 6.8 Hz, 3H). **^{13}C NMR** (150 MHz, $CDCl_3$) δ 200.8, 193.0, 169.7, 168.8, 150.2, 120.0, 110.7, 109.5, 82.5, 65.3, 65.3, 41.3, 37.9, 32.0, 23.4, 22.9, 22.7, 19.4, 14.2. **HRMS** (ESI) *m/z* calculated for C19H27O7$^+$ [M+H]$^+$ 367.1751, found 367.1752.

(*R*,*Z*)-3-hexanoyl-6-(hydroxymethylene)-5,7a-dimethylbenzofuran-2,7(6H,7aH)-dione (26). To the solution of **24** (9.8 mg, 27 μmol) in MeCN (2 mL), 2 mL of 1 N HCl was added. Then, the mixture was heated to 50 °C overnight and monitored by LC-MS until the starting material was consumed. After, the solvent was removed and 5 ml 50 M KPi pH 8.0 buffer was added which produced the product within 5 min. Then, the solvent was removed and the linear:angular (l:a) ratio was determined by crude NMR. The mixture was purified by preparative HPLC using a Phenomenex Kinetex 5 µm C18, 150 x 21.2 mm column under the following conditions: mobile phase A = deionized water and B = acetonitrile; method = 5% B hold 1 min, 5% to 20% B over 2 min, 20% to 50% B over 5 min, 50% to 90% B over 9 min, 90% to 100% B over 5 min, 100% B for 3 min; flow rate, 10 mL/min. Fractions containing the purified compound were pooled and the solvent was removed using a rotary evaporator provided 3.1 mg (38% yield over two steps) of product **26** as a single isomer. **^{1}H NMR** (600 MHz, CD_3OD) δ 9.60 (s, 1H), 6.48 (s, 1H), 1.64 (s, 3H), 1.58 (t, *J* = 7.0 Hz, 2H), 1.37 – 1.30 (m, 4H), 0.91 (t, *J* = 7.0 Hz, 3H). **^{1}H NMR** (600 MHz, CD_3CN) δ 9.68 (s, 1H), 6.33 (s, 1H), 3.65 (s, 1H), 2.83 – 2.67 (m, 2H), 2.40 (s, 3H), 1.59 – 1.48 (m, 5H), 1.35 – 1.28 (m, 4H), 0.89 (t, *J* = 7.0 Hz, 3H). **^{13}C NMR** (150 MHz, CD_3OD) δ 199.7, 198.8, 187.5, 180.2, 174.0, 165.4, 161.5, 112.8, 107.0, 106.9, 87.8, 32.8, 32.6, 25.5, 23.6, 14.3. **HRMS** (ESI) *m/z* calculated for C17H21O5$^+$ [M+H]$^+$ 305.1384, found 305.1386.

VI. Transformation.

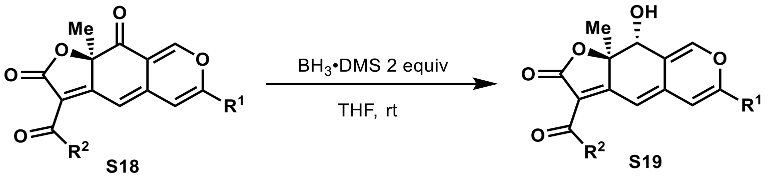

To the solution of corresponding linear azaophlione product (1 equiv) in THF (2.5 mM), BH_3·DMS (2 equiv,1 M stock) was added under 0 °C. After 5 min, UPLC was used to check the reaction and the starting material was consumed. MeOH was added to quench the reaction, and then the solvent was removed and the product was isolated via preparative HPLC using a Phenomenex Kinetex 5 µm C18, 150 x 21.2 mm column under the following conditions: mobile phase A = deionized water and B = acetonitrile; method = 5% B hold 1 min,5% to 20% B over 2 min, 20% to 50% B over 5 min, 50% to 90% B over 9 min, 90% to 100% B over 5 min, 100% B for 3 min; flow rate, 10 mL/min. Fractions containing the purified compound were pooled and the solvent was removed using a rotary evaporator provided the correspond product **10** or **47** as a single isomer.

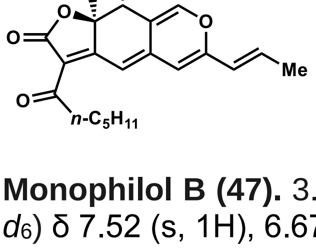

Monophilol B (47). 3.5 mg, 77% yield, bright orange, amorphous solid. **^{1}H NMR** (600 MHz, Acetone-*d*$_6$) δ 7.52 (s, 1H), 6.67 (s, 1H), 6.59 (dq, *J* = 15.6, 7.0 Hz, 1H), 6.42 (s, 1H), 6.23 (d, *J* = 15.6 Hz, 1H), 5.50 (d, *J* = 5.0 Hz, 1H), 4.72 (dd, *J* = 4.9, 2.2 Hz, 1H), 2.83 (m, 2H, overlap with water peak), 1.91 (d, *J* = 6.9 Hz, 3H), 1.60 – 1.54 (m, 2H),1.32 (s, 3H), 1.31 – 1.27 (m, 4H), 0.88 (t, *J* = 6.7 Hz, 3H). **^{13}C NMR** (150 MHz, Acetone-*d*$_6$) δ 197.1, 174.0, 171.3, 158.2, 147.1, 145.9, 135.4, 124.2, 122.7, 111.7, 109.1,

103.5, 83.7, 72.0, 41.7, 32.3, 24.4, 23.2, 19.7, 18.5, 14.3. **HRMS** (ESI) *m/z* calculated for C21H24NaO5$^+$ [M+Na]$^+$ 379.1516, found 379.1518.

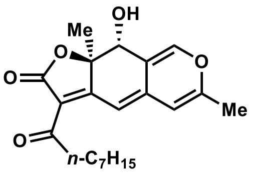

(*9R*,*9aR*)-9-hydroxy-6,9a-dimethyl-3-octanoyl-9,9a-dihydro-2H-furo[3,2-g]isochromen-2-one (pitholide D's distereoisomer, 10). 2.0 mg, 73% yield, luminous yellow, amorphous solid**.** **^{1}H NMR** (600 MHz, CD_3OD) δ 7.58 (s, 1H), 6.64 (s, 1H), 6.37 (s, 1H), 4.71 (d, *J* = 1.7 Hz, 1H), 2.94 – 2.81 (m, *J* = 7.3 Hz, 2H), 2.24 (s, 3H), 1.64 – 1.55 (m, 2H), 1.34 (s, 3H), 1.34 – 1.28 (m, 8H), 0.90 (t, *J* = 7.0 Hz, 3H). **^{13}C NMR** (150 MHz, CD_3OD) δ 199.4, 176.4, 173.5, 164.0, 149.1, 148.7, 123.8, 110.5, 109.9, 102.5, 85.0, 72.2, 42.1, 32.9, 30.4, 30.3, 25.5, 23.7, 19.7, 19.4, 14.4. **HRMS** (ESI) *m/z* calculated for C21H26NaO5$^+$ [M+Na]$^+$ 381.1672, found 381.1676.

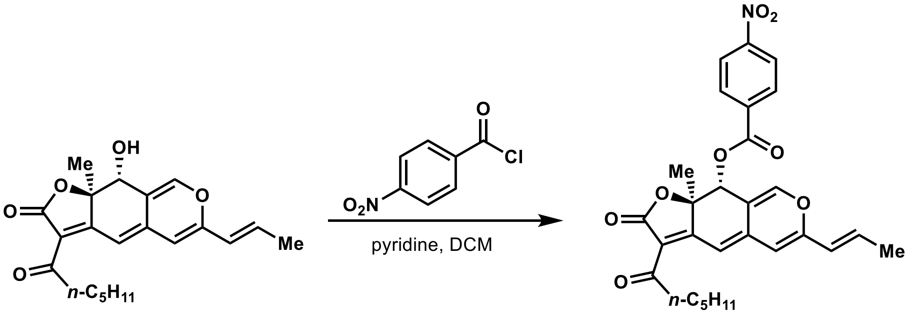

47 S20

(*9R*,*9aR*)-3-hexanoyl-9a-methyl-2-oxo-6-((E)-prop-1-en-1-yl)-9,9a-dihydro-2H-furo[3,2-g]isochromen-9-yl 4-nitrobenzoate (S20). To a solution of **47** (2.3 mg, 6.5 µmol) in CH_2Cl_2 (0.5 mL) and pyridine (0.1 mL), 4-nitrobenzoyl chloride (12 mg, 65 µmol) was added. The mixture was stirred overnight at room temperature. The mixture was evaporated to dryness and purified by preparative TLC, eluting with EtOAc-hexane to yield orange amorphous solid **S20** (2.2 mg, 67% yield). Orange amorphous solid. **^{1}H NMR** (600 MHz, $CDCl_3$) δ 8.37 (d, *J* = 8.6 Hz, 2H), 8.30 (d, *J* = 8.6 Hz, 2H), 7.02 (s, 1H), 6.82 (s, 1H), 6.56 – 6.46 (m, 1H), 6.24 (s, 1H), 6.10 (s, 1H), 6.04 (d, *J* = 16.9 Hz, 1H), 3.00 – 2.93 (m, 2H), 1.92 (d, *J* = 7.7 Hz, 3H), 1.67 – 1.62 (m, 2H), 1.60 (s, 3H), 1.36 – 1.31 (m, 4H), 0.90 (t, *J* = 6.8 Hz, 3H). **^{13}C NMR** (150 MHz, $CDCl_3$) δ 198.0, 171.4, 170.4, 163.6, 157.29, 151.2, 144.7, 144.1, 135.8, 134.3, 131.3, 124.0, 123.0, 117.8, 111.9, 108.5, 103.9, 80.6, 73.6, 41.7, 31.6, 23.6, 22.7, 21.0, 18.8, 14.1. **HRMS** (ESI) *m/z* calculated for C28H27NNaO8$^+$ [M+H]$^+$ 528.1629, found 528.1634.

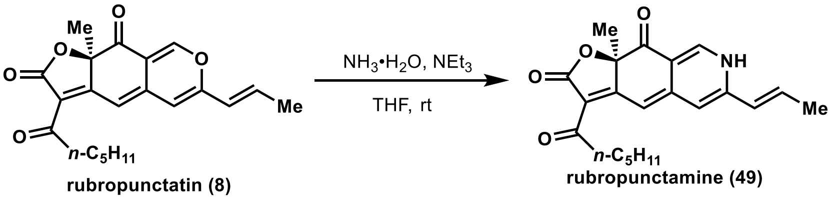

rubropunctatin (8) rubropunctamine (49)

Rubropunctatamine (49). To the solution of rubropunctatin **8** (3.5 mg) in 1 mL THF, 0.1 ml triethylamine and 0.2 ml ammonium hydroxide (29.3%) was added to the mixture. After 5 min, the starting material was consumed (detected by UPLC), and the solvent was removed under vacuum. The mixture was dissolved in 0.2 mL ethyl acetate. Then 1.5 mL hexane was added to the solution give red precipitate. After centrifuge, the precipitate was collected give product 3.1 mg (89% yield) **49**. Red amorphous solid. **^{1}H NMR** (600 MHz, $CDCl_3$) δ 12.62 (s, 1H), 9.27 (s, 1H), 7.05 – 6.98 (m, 1H), 6.78 (s,

1H), 6.73 (s, 1H), 6.35 (d, *J* = 15.9 Hz, 1H), 2.91 – 2.83 (m, 2H), 2.05 (d, *J* = 6.5 Hz, 3H), 1.81 (s, 3H), 1.69 – 1.64 (m, 2H), 1.38 – 1.34 (m, 4H), 0.90 (t, *J* = 6.0 Hz, 3H). **^{13}C NMR** (150 MHz, $CDCl_3$) δ 196.6, 195.4, 174.4, 173.0, 153.7, 147.3, 140.9, 138.1, 123.34, 117.5, 116.1, 101.1, 98.7, 86.8, 40.6, 31.9, 29.6, 24.5, 22.5, 19.2, 14.0. **HRMS** (ESI) *m/z* calculated for $C21H24NO4^+$ $[M+H]^+$ 354.1700, found 354.1706.

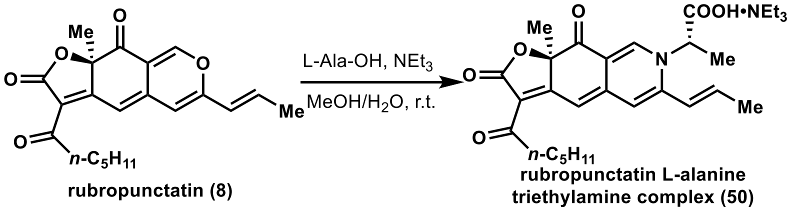

Triethylamine rubropunctatin *L*-alanine (50). To a solution of rubropunctain **8** (2 mg) in 1 mL H_2O/MeOH (1:4), *L*-alanine (2 equiv) and triethylamine (10% by volume) was added. The reaction was stirred at 30 °C for 30 min. The reaction was monitored by UPLC, and after 30 min the starting material was consumed. The solvent was removed, and the resulting mixture was diluted to 1 mL with acetonitrile and subjected to purification by preparative HPLC using a Phenomenex Kinetex 5 µm C18, 150 x 21.2 mm column under the following conditions: mobile phase A = deionized water and B = acetonitrile; method = 5% B hold 1 min, 5% to 20% B over 2 min, 20% to 50% B over 5 min, 50% to 90% B over 9 min, 90% to 100% B over 5 min, 100% B for 3 min; flow rate, 10 mL/min. Fractions containing the purified compound were pooled and the solvent was removed using a rotary evaporator give product **50** (90% yield). **^{1}H NMR** (600 MHz, CD_3OD) δ 8.28 (s, 1H), 7.09 (s, 1H), 6.73 (s, 1H), 6.66 – 6.57 (m, 1H), 6.51 (dd, *J* = 15.6, 1.2 Hz, 1H), 4.97 (q, *J* = 7.2 Hz, 1H), 3.21 (q, *J* = 7.3 Hz, 6H), 2.82 (td, *J* = 7.2, 1.7 Hz, 2H), 1.99 (dd, *J* = 6.6, 1.5 Hz, 3H), 1.74 (d, *J* = 7.2 Hz, 3H), 1.65 (s, 3H), 1.62 – 1.59 (m, 2H), 1.35 (dd, *J* = 7.1, 3.7 Hz, 4H), 1.31 (t, *J* = 7.2 Hz, 9H), 0.91 (t, *J* = 7.0 Hz, 3H). **^{13}C NMR** (150 MHz, CD_3OD) δ 198.7, 196.2, 173.9, 173.7, 153.5, 152.9, 141.5, 141.3, 123.6, 119.7, 119.4, 102.0, 98.9, 86.9, 65.4, 47.9 (NEt_3 C_α), 41.2, 32.9, 30.7, 26.2, 23.6, 19.2, 18.6, 14.3, 9.2 (NEt_3 C_β). **HRMS** (ESI) *m/z* calculated for $C24H28NO6^+$ $[M+H]^+$ 426.1911, found 426.1919.

VII. Standard curves and quantification of biocatalytic reactions

Supplemental Figure 2. substrate standard curves for quantification of percent conversion of analytical reactions.

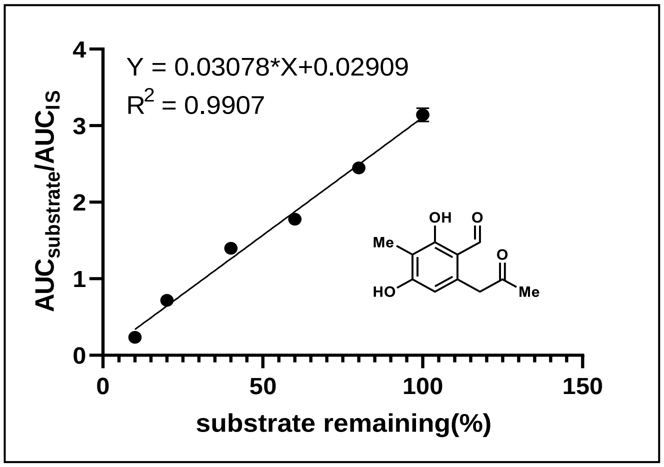

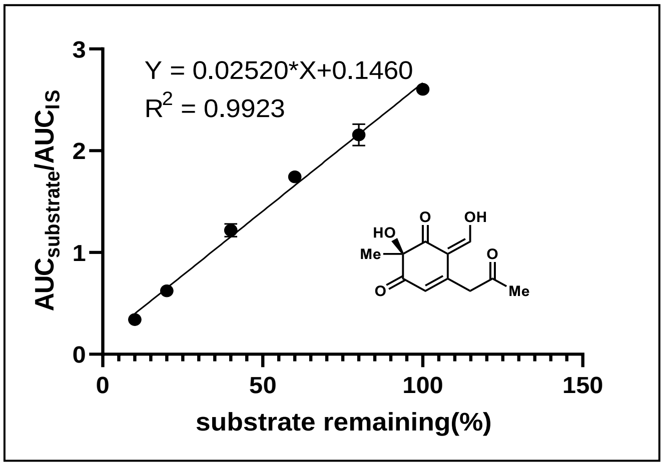

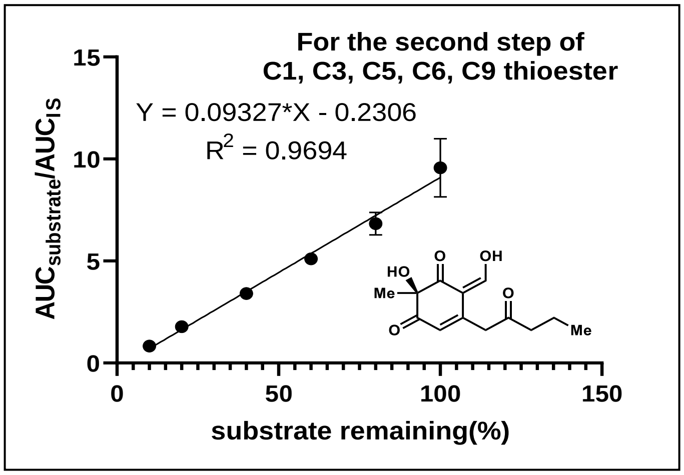

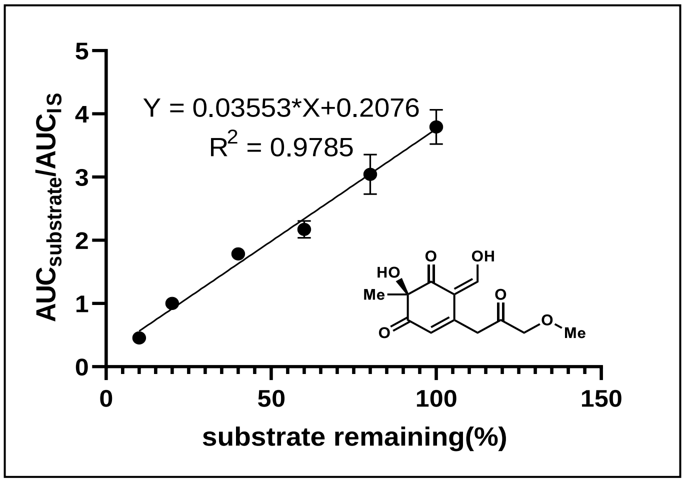

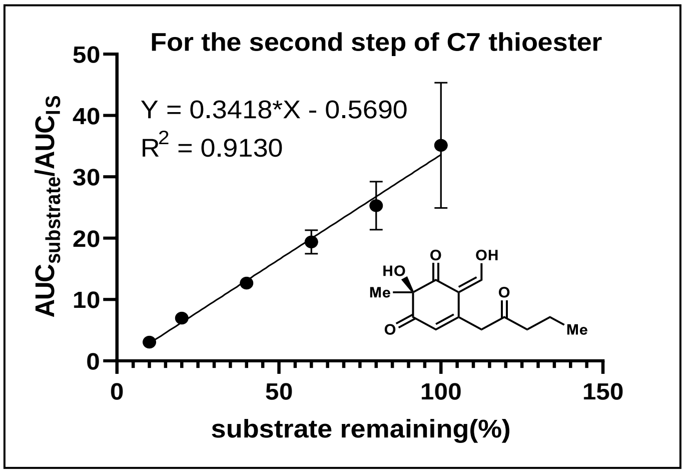

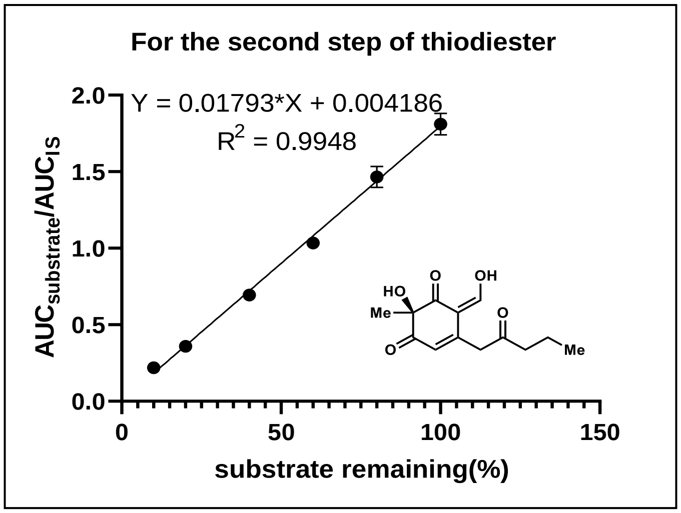

VIII. Kinetic experiment.

Supplemental Figure 3. product standard curves for quantification of product formation concentration and the related kinetic data.
Product standard curves:

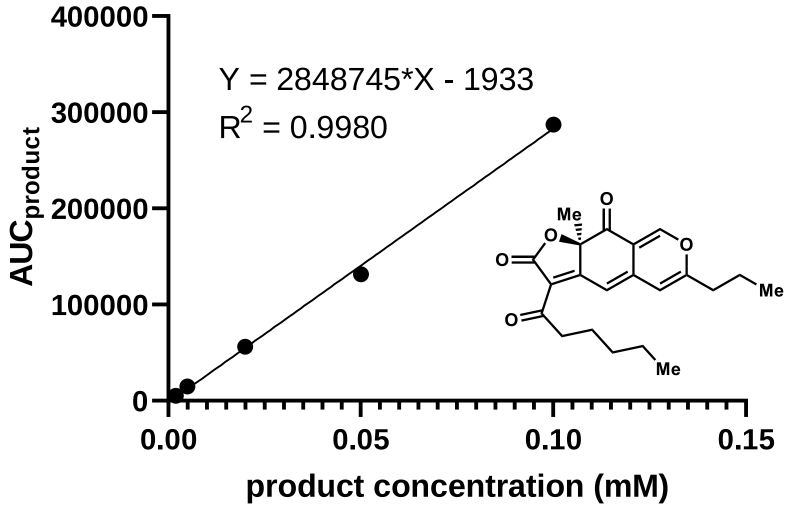

A: Open form of substrate

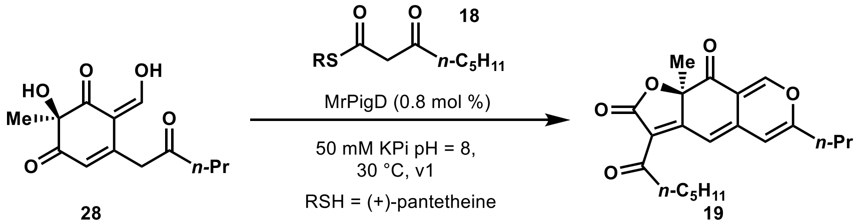

The reaction contained 2.5 mM of the orcinaldehyde substrate (20 uL of a 50 mM stock solution in DMSO), 5 mM G6P (4 μL, 500 mM), 1 mM NADP+ (4 μL, 100 mM), 1 U/mL G6P-DH (4 μL, 100 U/mL), 10 μM AzaH (13.2 μL of a 300 μM stock solution), 310 μL water, and 20 μL of a 1 M potassium phosphate buffer, pH 8.0. The dearomatization reaction was carried out at 30 °C for 1 h. Following this, 10 μL water, 2.5 mM thioester **18** (10 μL of a 100 mM stock solution in DMSO) and 20 μM MrPigD (4.8 μL of a 1.7 mM stock solution) were then added, and the second reaction was carried out at 30 °C. At different reaction timepoints, 20 μL of the reaction mixture was taken and quenched with 40 μL of MeCN, then the precipitated biomolecules were pelleted by centrifugation (17,000 x g, 10 min). 40 μL of the supernatant was added 5 μL 1 N HCl, and then analyzed by UPLC-DAD.

B: Closed form of substrate

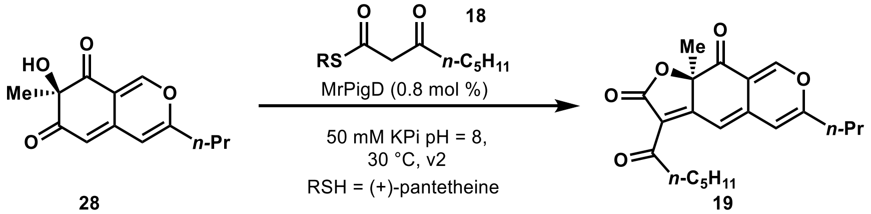

The reaction contained 2.5 mM of the close form dearomatization substrate (10 uL of a 100 mM stock solution in DMSO), 2.5 mM thioester **18** (10 μL of a 100 mM stock solution in DMSO) and 20 μM MrPigD (4.8 μL of a 1.7 mM stock solution), water 355 μL and 20 μL of a 1 M potassium phosphate buffer, pH 8.0. The reaction was carried out at 30 °C. At different reaction time, 20 μL of the reaction mixture was taken and quenched with 40 μL of MeCN, then the precipitated biomolecules were pelleted by centrifugation (17,000 x g, 10 min). 40 μL of the supernatant was added 5 μL 1 N HCl, and then analyzed by UPLC-DAD.

C: Mixture of substrates

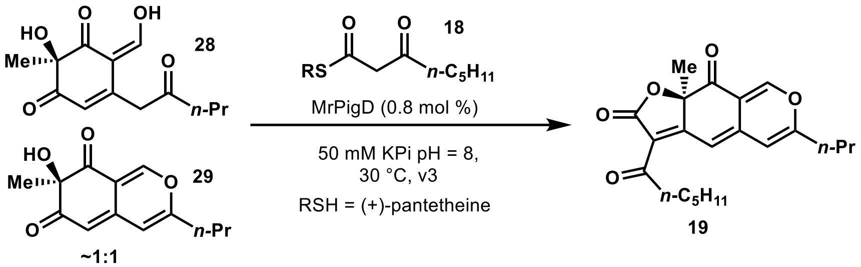

Reaction contained 2.5 mM of the orcinaldehyde substrate (20 uL of a 50 mM stock solution in DMSO), 5 mM G6P (4 µL, 500 mM), 1 mM NADP+ (4 µL, 100 mM), 1 U/mL G6P-DH (4 µL, 100 U/mL), 10 µM AzaH (13.2 µL of a 300 µM stock solution), 310 µL water and 20 µL of a 1 M potassium phosphate buffer, pH 8.0. The dearomatization reaction was carried out at 30 °C for 1 h. Following this, 2.5 mM of the closed form dearomatization substrate (10 µL of a 100 mM stock solution), 2.5 mM thioester **18** (10 µL of a 100 mM stock solution in DMSO) and 20 µM MrPigD (4.8 µL of a 1.7 mM stock solution) were then added, the second reaction was carried out at 30 °C. At different reaction time, 20 µL of the reaction mixture was taken and quenched with 40 µL of MeCN, then the precipitated biomolecules were pelleted by centrifugation (17,000 x g, 10 min). 40 µL of the supernatant was added 5 µL 1 N HCl, and then analyzed by UPLC-DAD.

Kinetic data: A(open), B(closed), C (mixture)

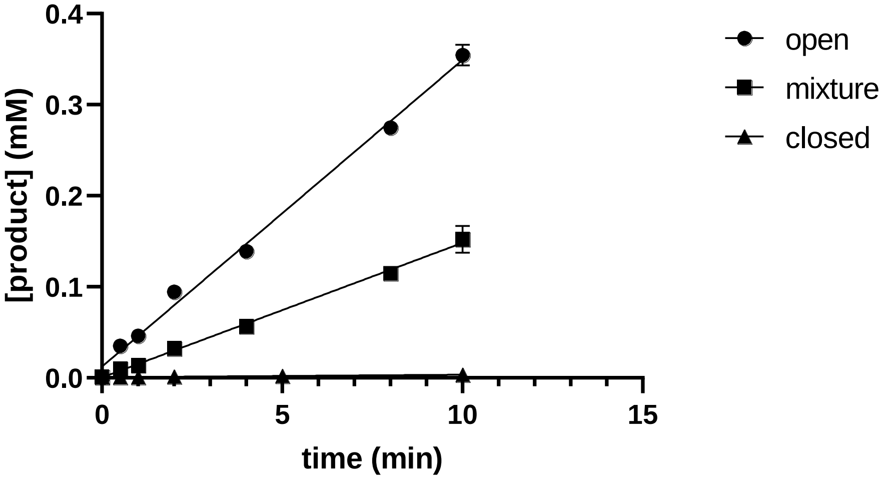

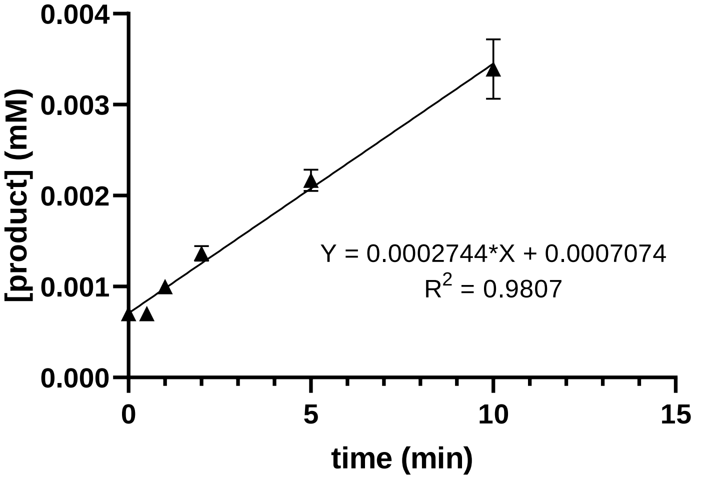

A(open): **v_1** = 0.0337*3*45/40 = 0.114 mM/min

B(closed): $\mathbf{v_2} = 0.0003*3*45/40 = 0.001$ mM/min
C(mixture): $\mathbf{v_3} = 0.0148*3*45/40 = 0.050$ mM/min
Conclusion: $\mathbf{v_1} = 2.28*\mathbf{v_3} = 114*\mathbf{v_2}$

IX. Different ratio of close (2) and open form (3) depend on R_1 group.

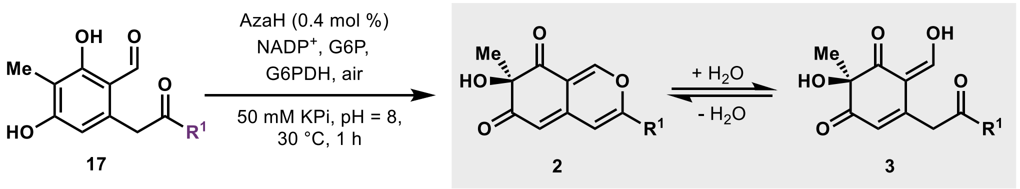

R_1 = *n*-Pr:

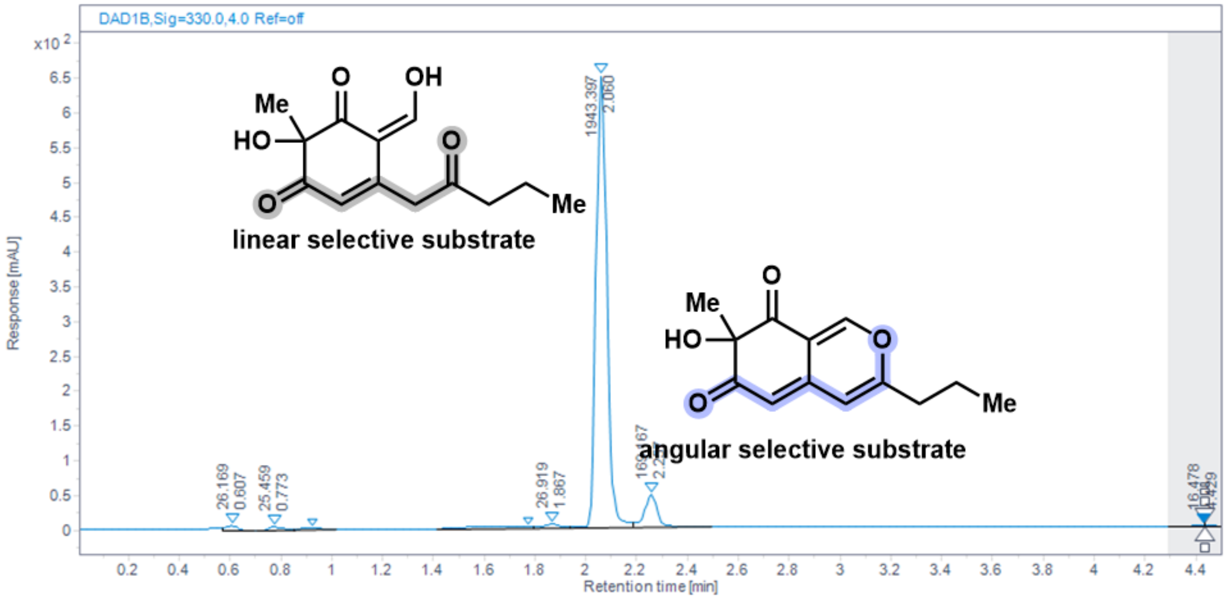

R_1 = -CH=CHCH$_3$:

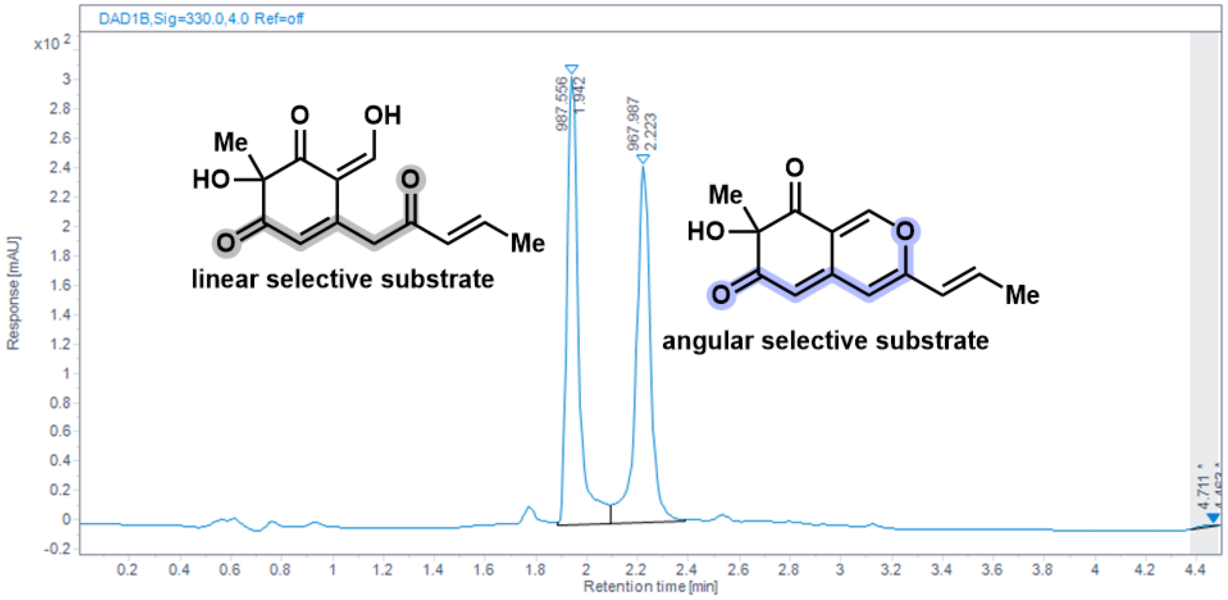

X. UPLC traces for analytical-scale biocatlytic reactions

Substrate **S1** - step1 with AzaH

270 nm:

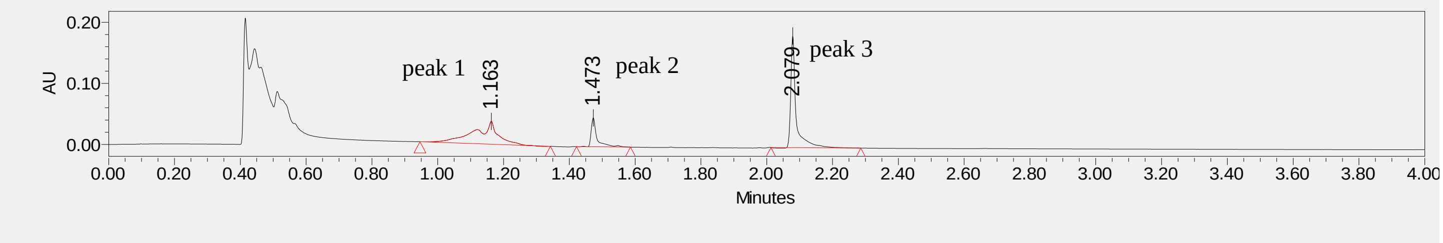

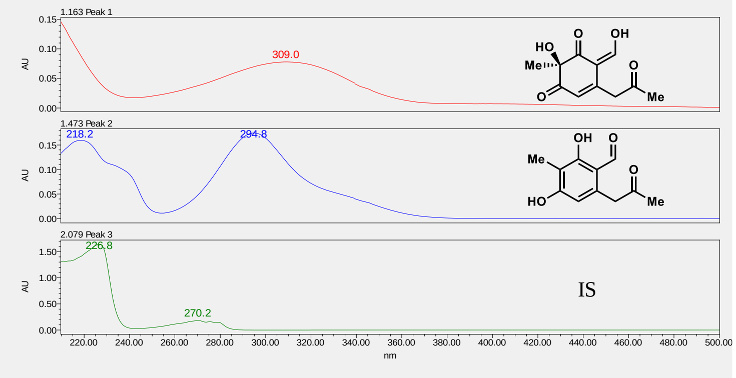

	Retention Time	Area	% Area
1	1.163	189423	41.42
2	1.473	59180	12.94
3	2.079	208672	45.63

Substrate **S1** – step 1 no enzyme control

270 nm:

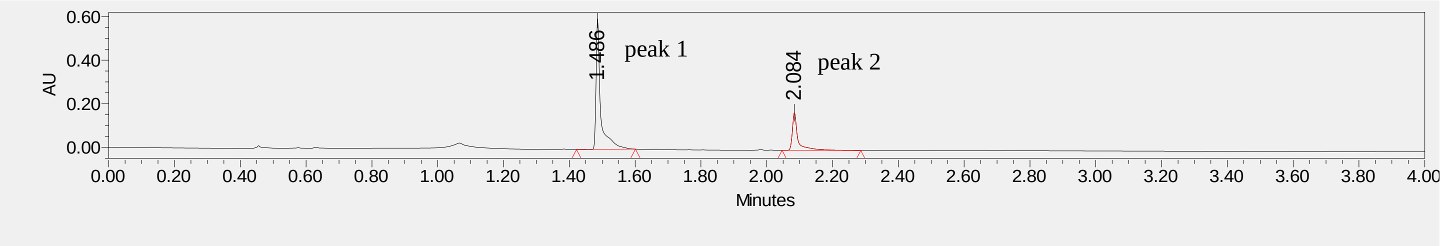

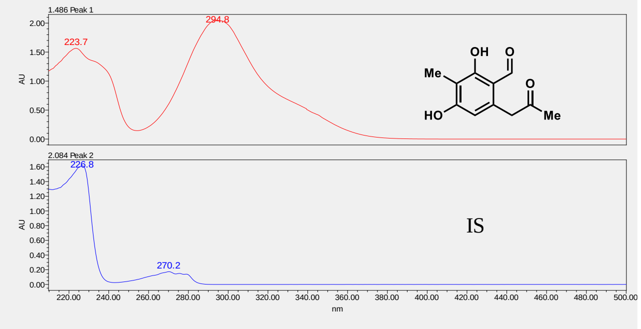

	Retention Time	Area	% Area
1	1.486	593433	74.07
2	2.084	207749	25.93

Substrate **S1** – step 2 with MrPigD

270 nm

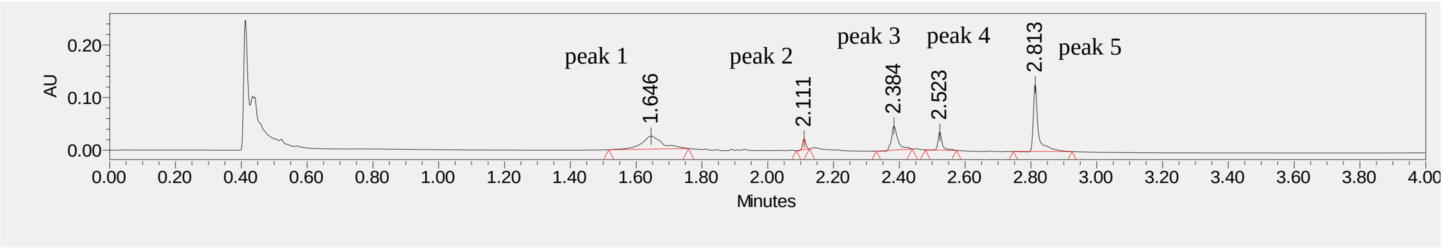

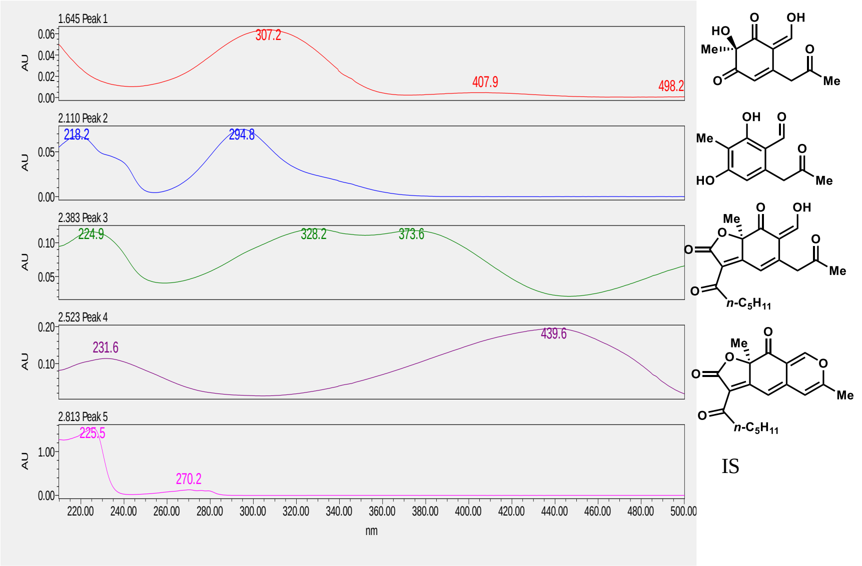

	Retention Time	Area	% Area
1	1.646	108808	30.9
2	2.111	14028	3.98
3	2.384	62672	17.8
4	2.523	30118	8.55
5	2.813	136477	38.76

440 nm

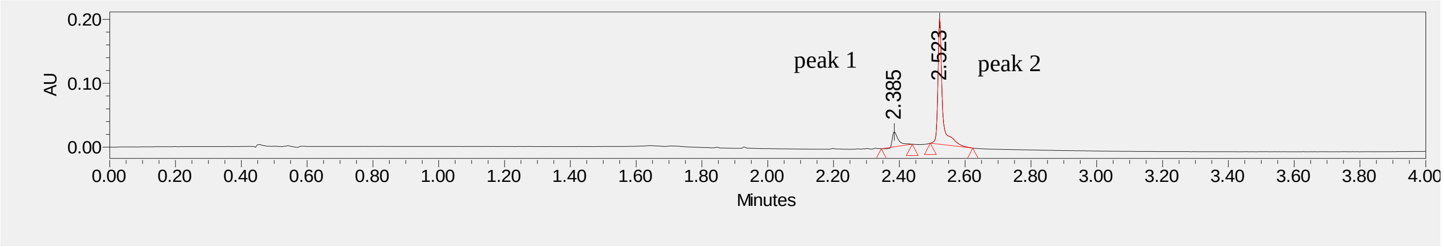

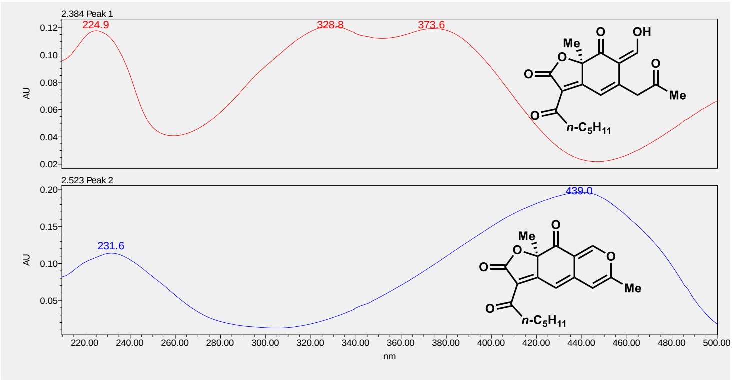

	Retention Time	Area	% Area
1	2.385	31476	14.28
2	2.523	188886	85.72

Substrate **S1** – step 2 no enzyme control

270 nm

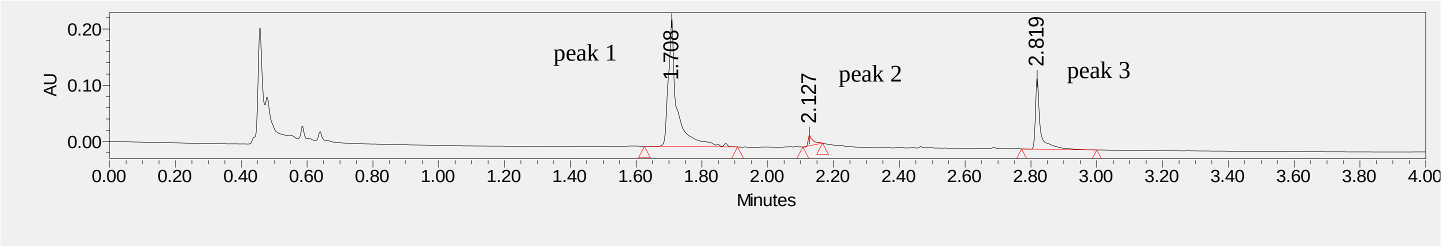

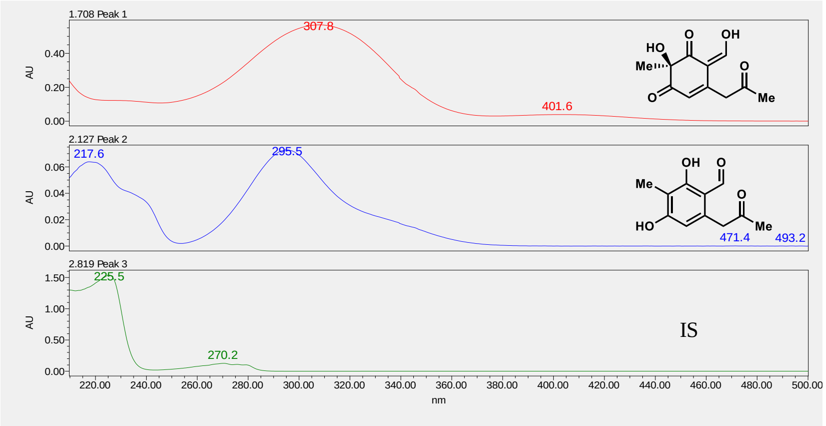

	Retention Time	Area	% Area
1	1.708	412580	73
2	2.127	18991	3.36
3	2.819	133599	23.64

440 nm

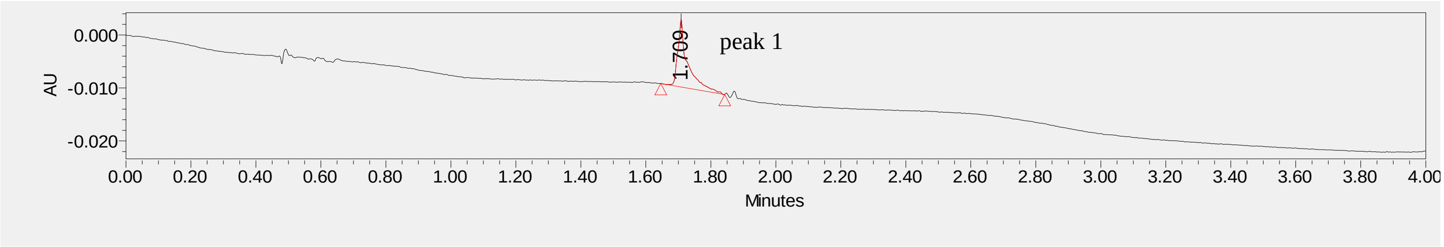

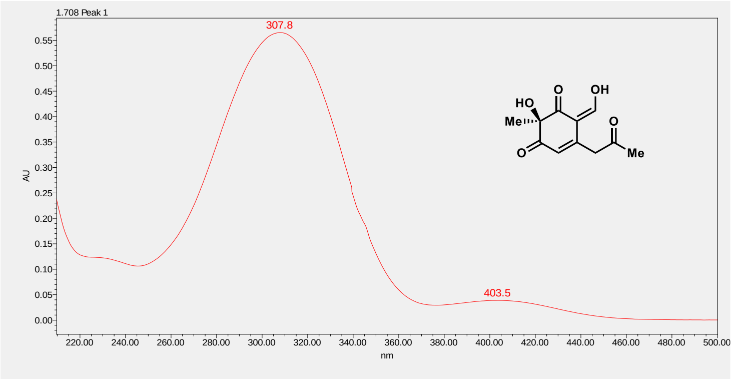

	Retention Time	Area	% Area
1	1.709	26849	100

Substrate **17** – step 1 with AzaH

270 nm:

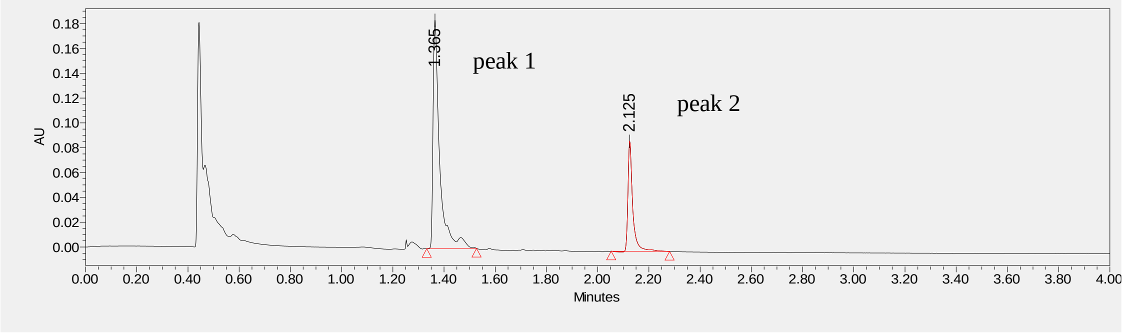

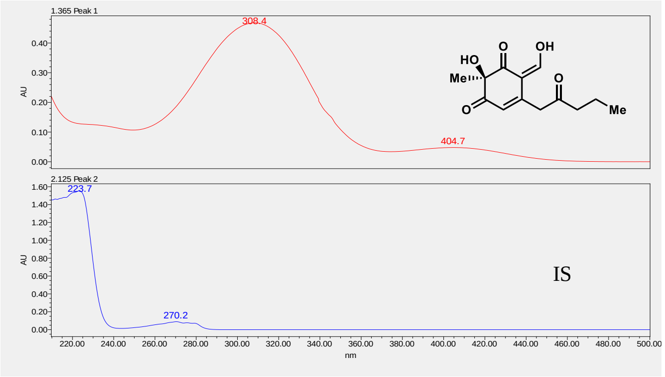

	Retention Time	Area	% Area
1	1.365	333914	74.06
2	2.125	116983	25.94

Substrate **17** – step 1 no enzyme control

270 nm:

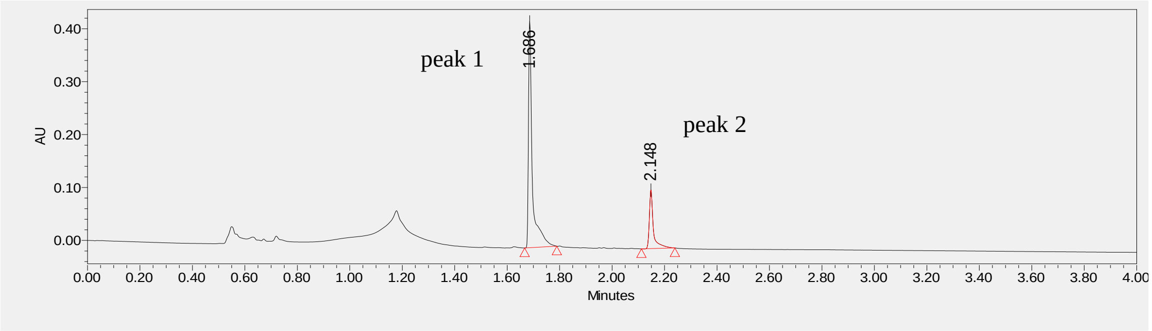

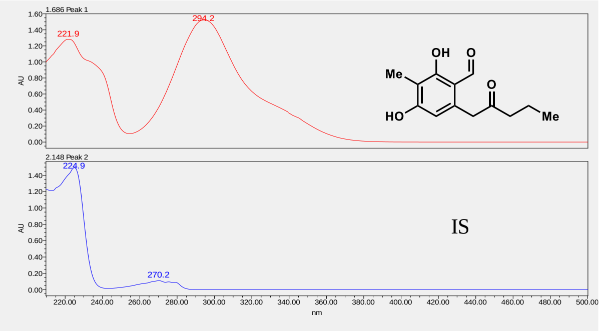

	Retention Time	Area	% Area
1	1.686	445555	78.98
2	2.148	118612	21.02

Substrate **17** – step 2 with C5 thioester and MrPigD

270 nm

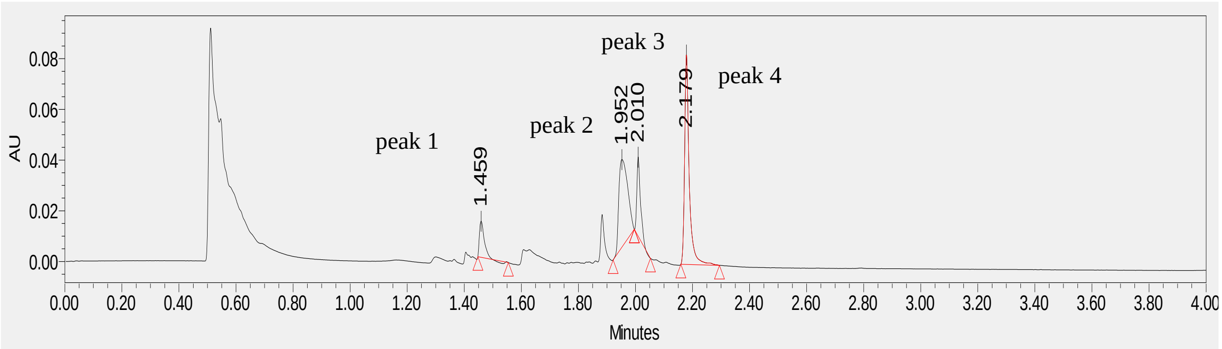

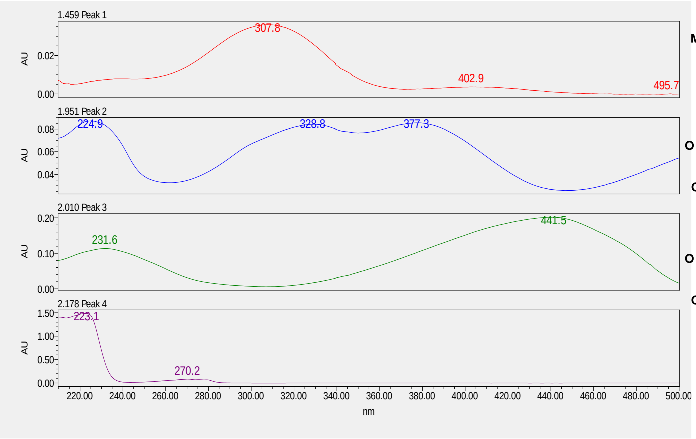

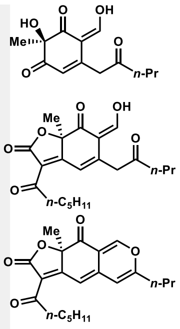

IS

	Retention Time	Area	% Area
1	1.459	18707	8.73
2	1.952	73793	34.45
3	2.01	28585	13.35
4	2.179	93116	43.47

440 nm

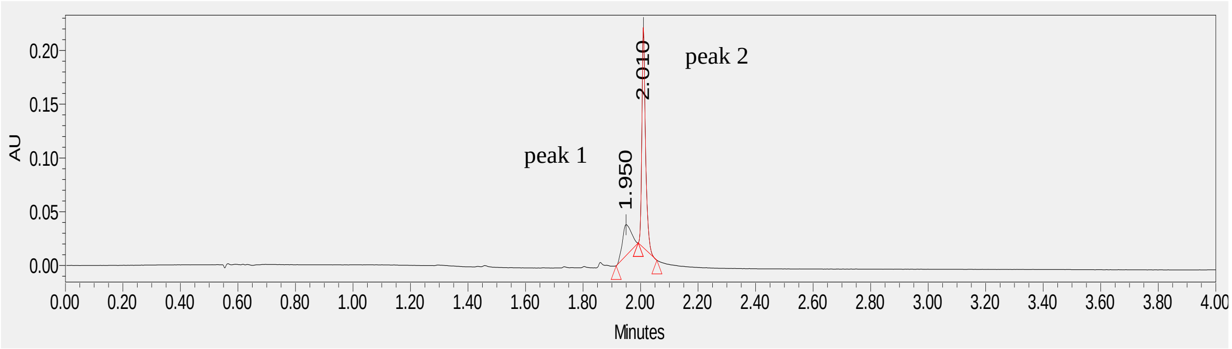

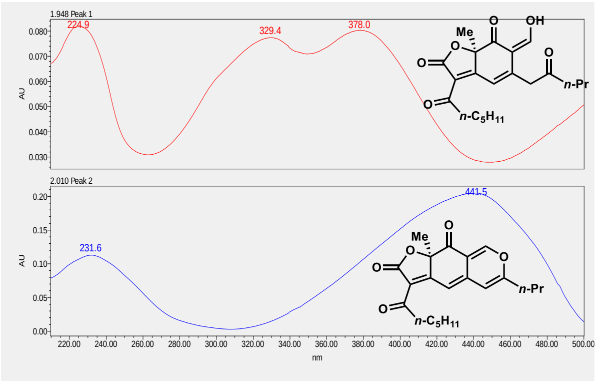

	Retention Time	Area	% Area
1	1.950	61498	25.26
2	2.010	181917	74.74

Substrate **17** – step 2 with C5 thioester and no enzyme control

270 nm

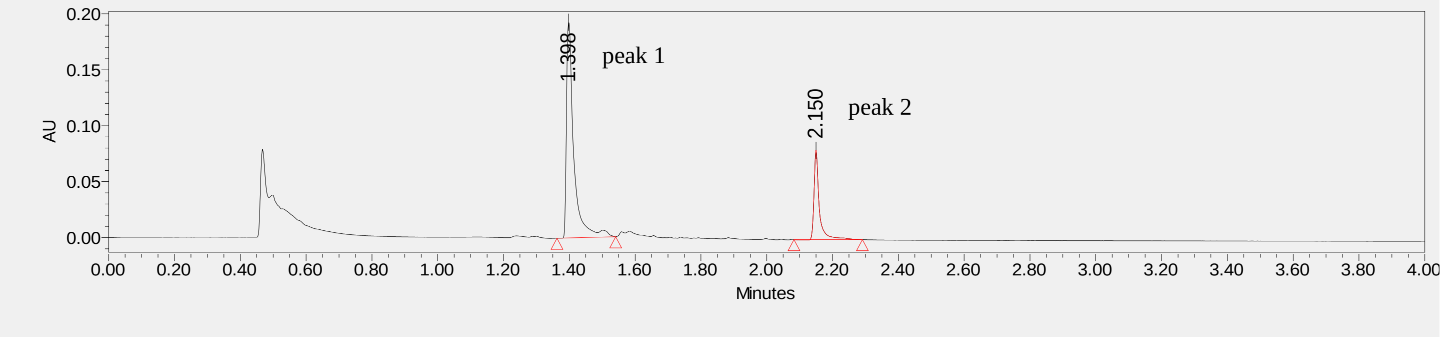

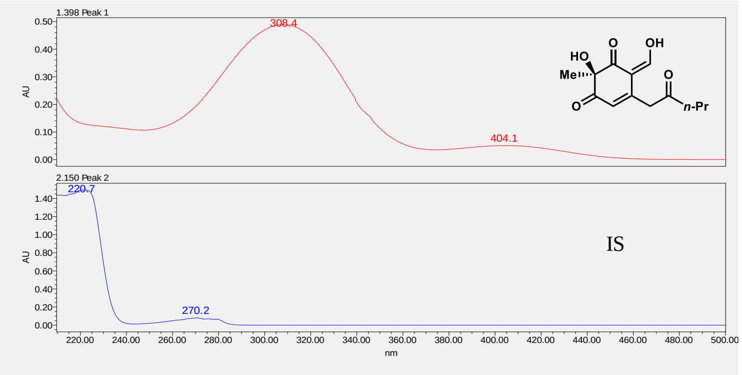

	Retention Time	Area	% Area
1	1.398	299771	76.96
2	2.15	89758	23.04

440 nm

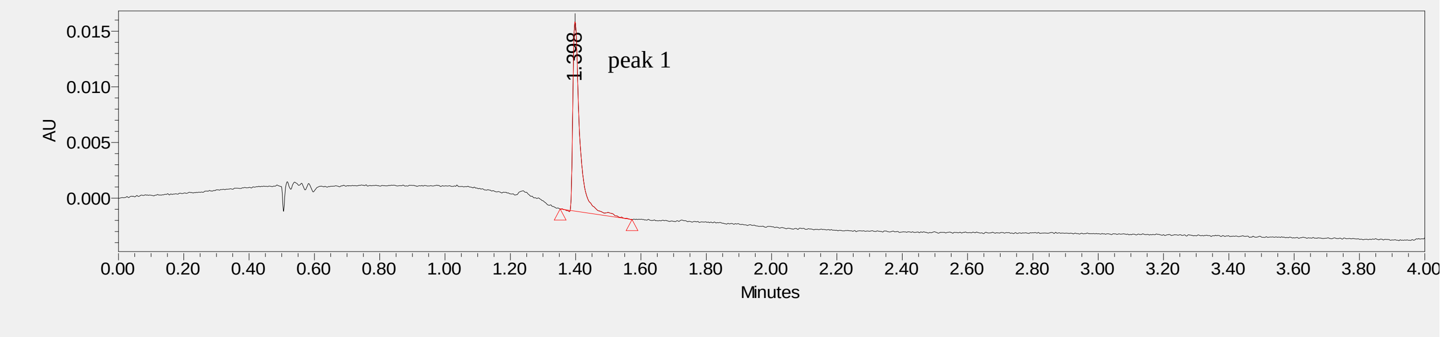

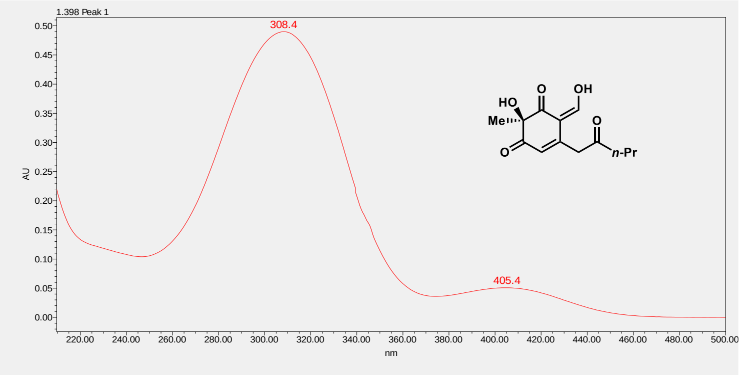

	Retention Time	Area	% Area
1	1.398	25479	100

Substrate **17** – step 2 with C1 thioester and MrPigD

270 nm:

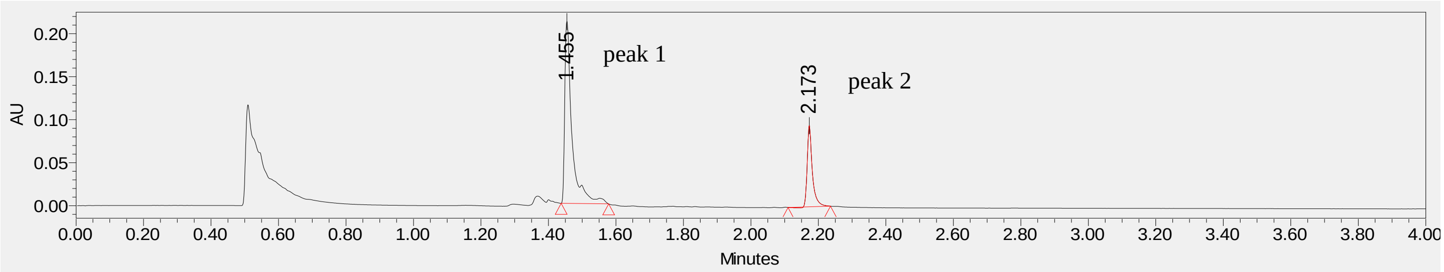

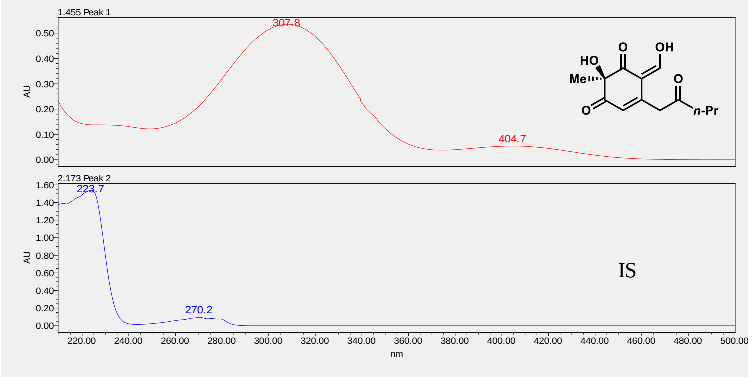

	Retention Time	Area	% Area
1	1.455	318093	75.78
2	2.173	101669	24.22

440 nm:

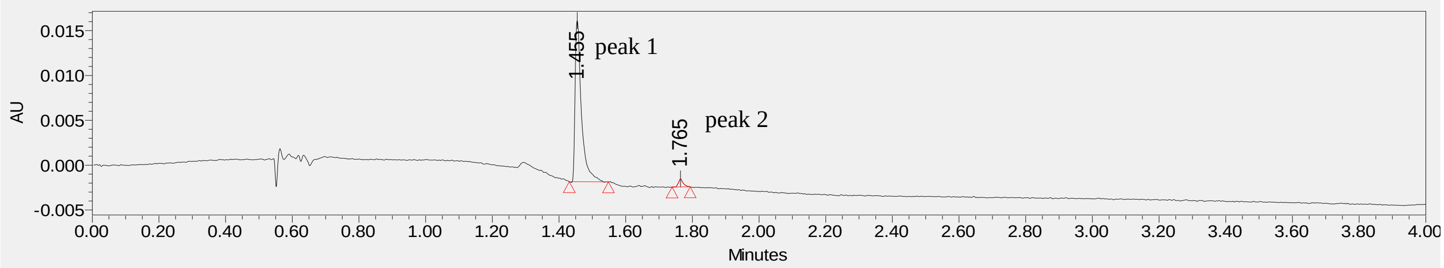

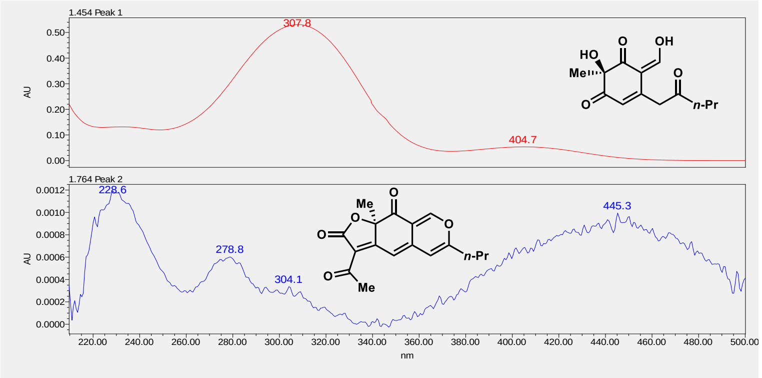

	Retention Time	Area	% Area
1	1.455	23930	96.46
2	1.765	879	3.54

Substrate **17** – step 2 with C1 thioester and no enzyme control

270 nm

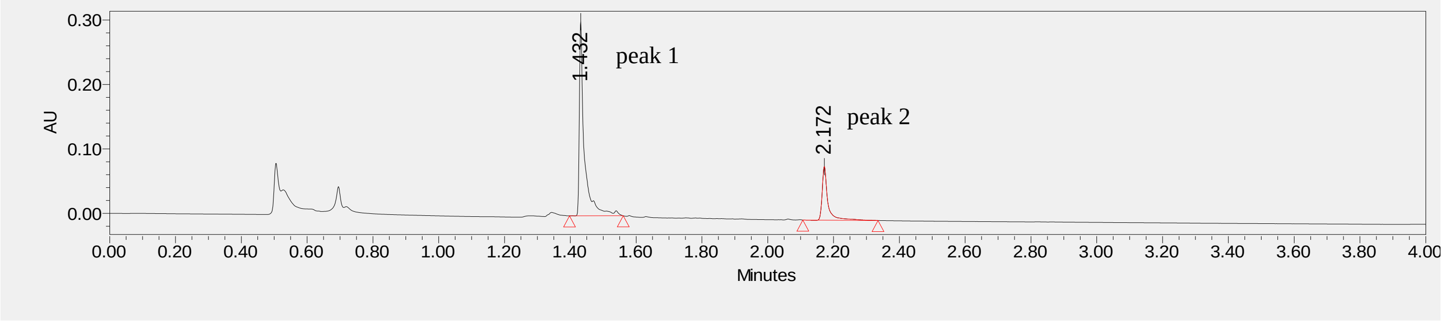

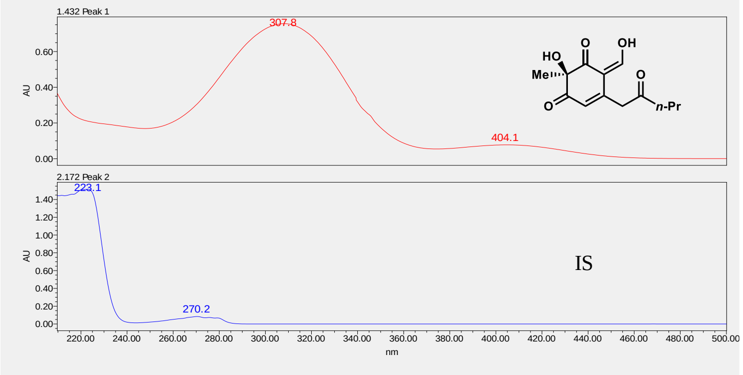

	Retention Time	Area	% Area
1	1.432	318817	76.06
2	2.172	100338	23.94

440 nm

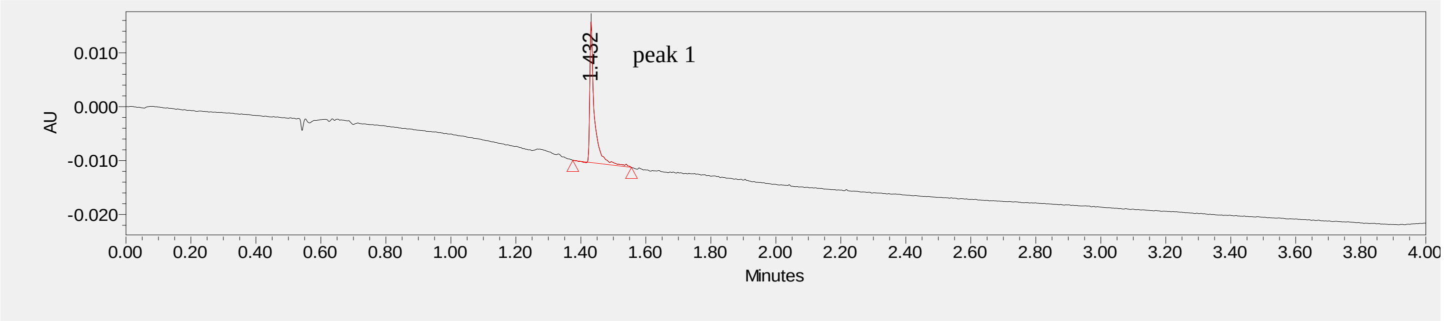

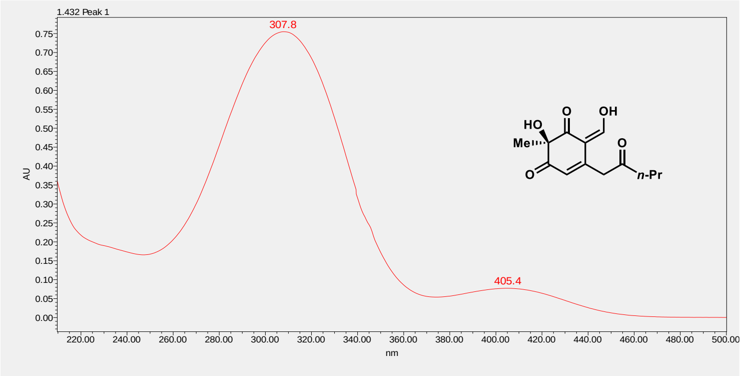

	Retention Time	Area	% Area
1	1.432	25781	100

Substrate **17** – step 2 with C3 thioester and MrPigD

270 nm:

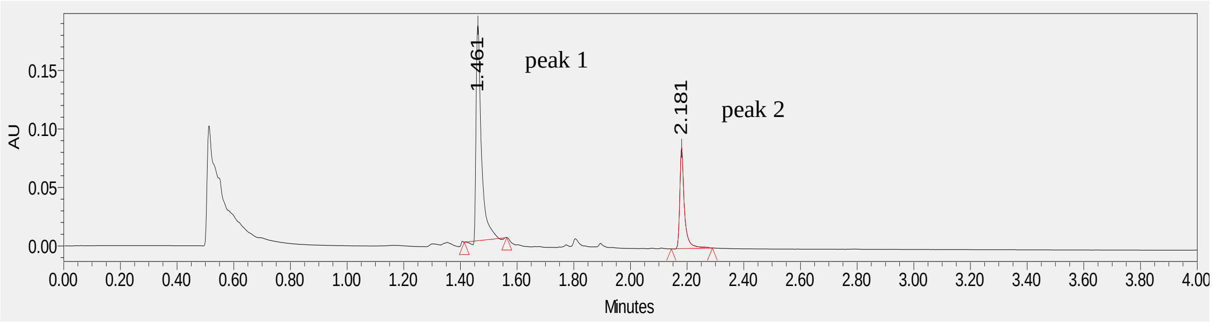

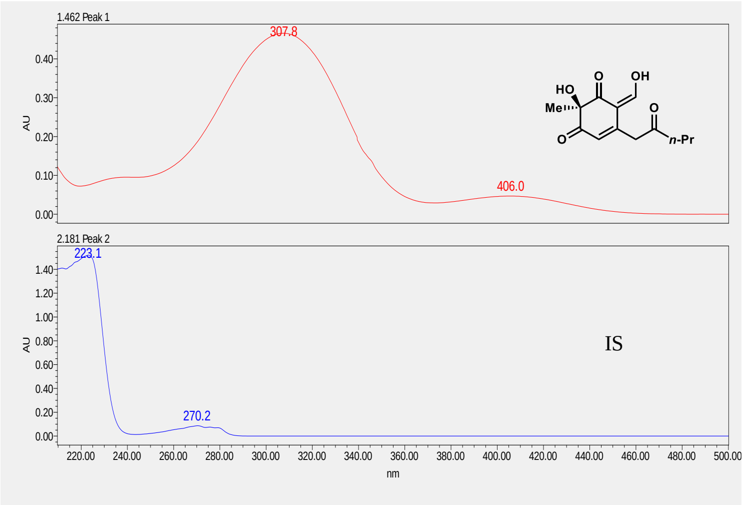

	Retention Time	Area	% Area
1	1.461	245262	71.46
2	2.181	97937	28.54

440 nm:

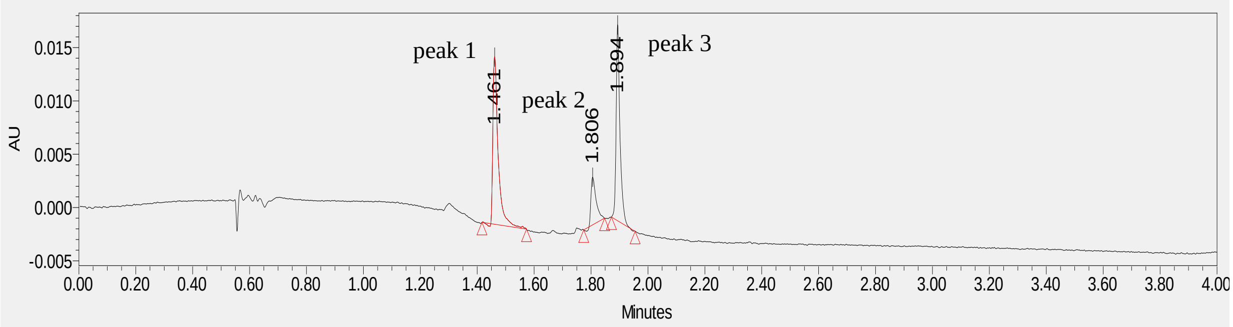

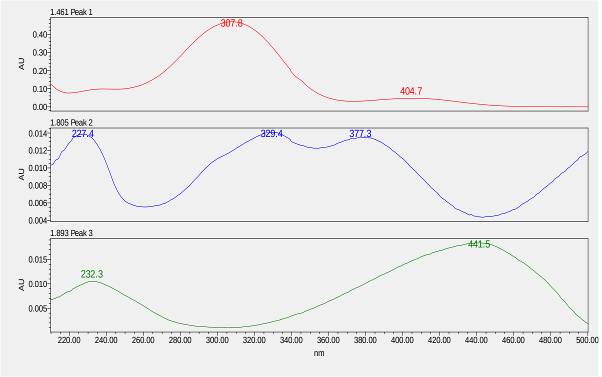

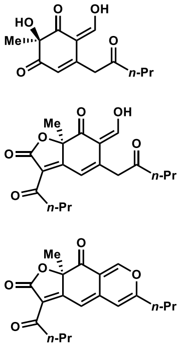

	Retention Time	Area	% Area
1	1.461	20494	47
2	1.806	5908	13.55
3	1.894	17199	39.45

Substrate **17** – step 2 with C3 thioester and no enzyme control

270 nm

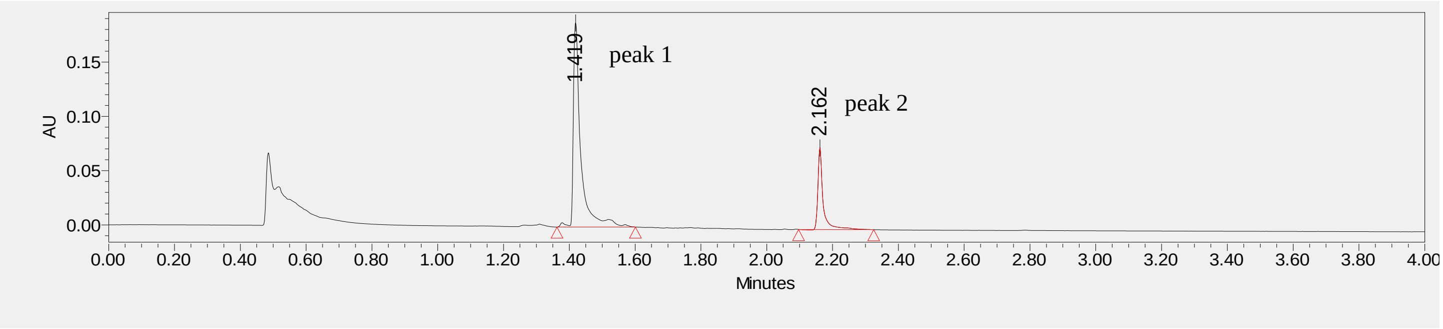

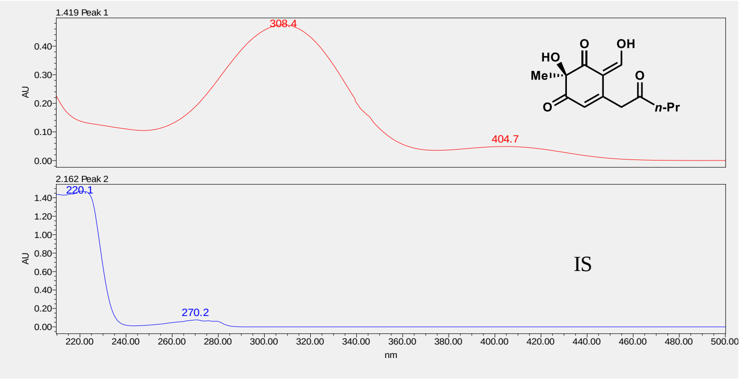

	Retention Time	Area	% Area
1	1.419	300155	78.09
2	2.162	84227	21.91

440 nm

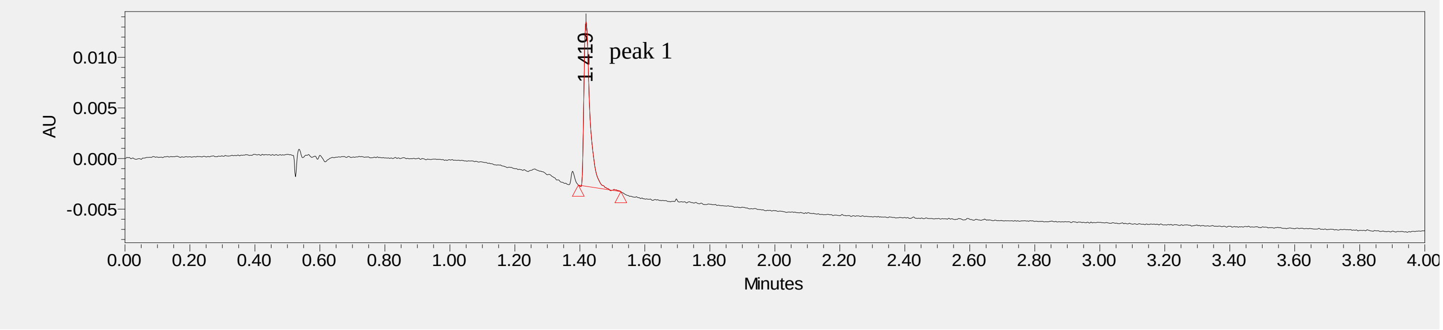

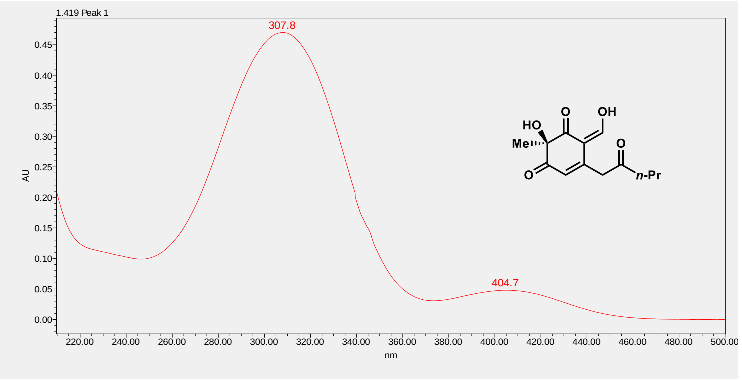

	Retention Time	Area	% Area
1	1.419	21341	100

Substrate **17** – step 2 with C6 thioester and MrPigD

270 nm:

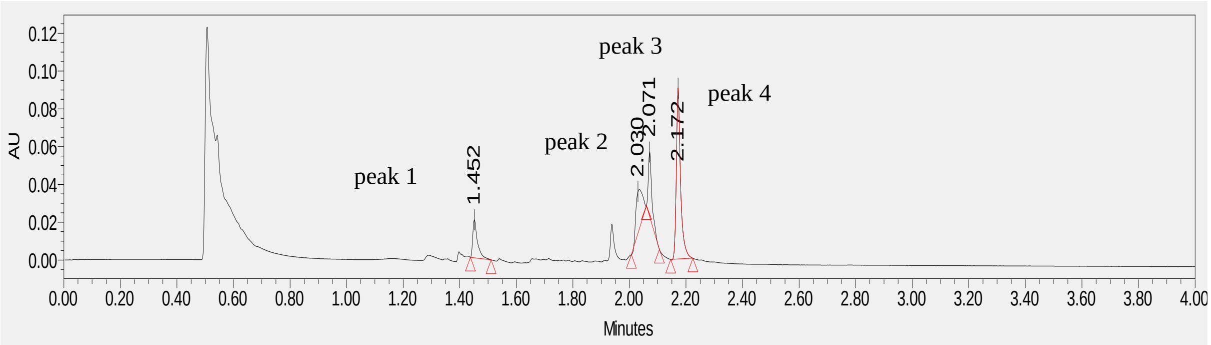

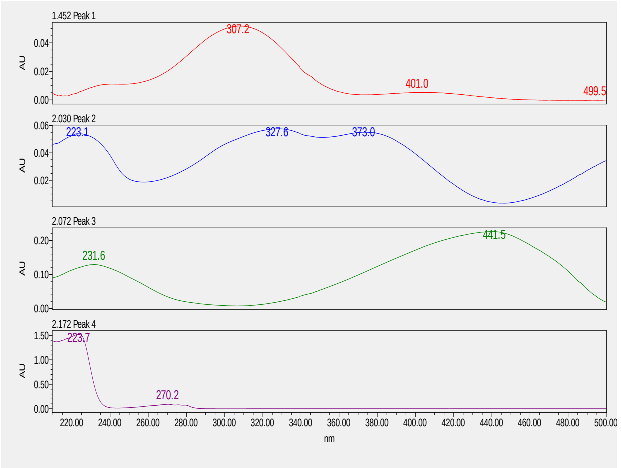

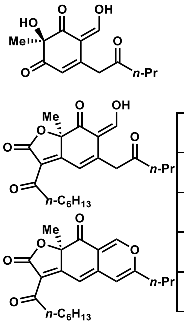

IS

	Retention Time	Area	% Area
1	1.452	23388	12.88
2	2.03	34560	19.04
3	2.071	29068	16.01
4	2.172	94542	52.07

440 nm:

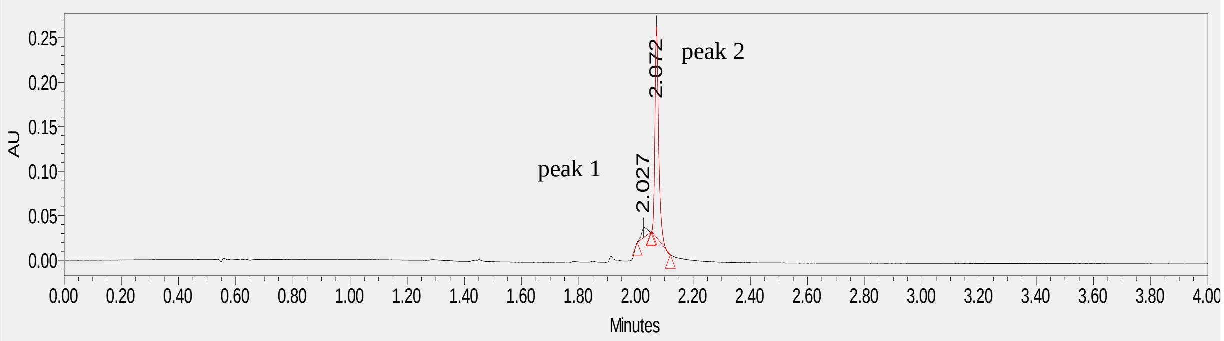

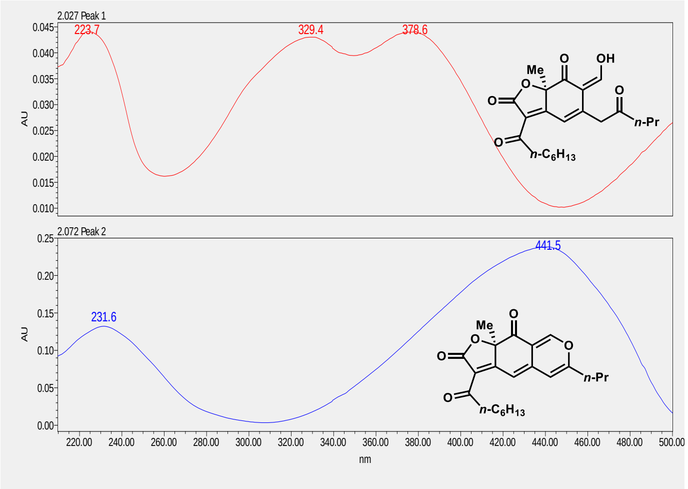

	Retention Time	Area	% Area
1	2.027	15066	6.64
2	2.072	211907	93.36

Substrate **17** – step 2 with C6 thioester and no enzyme control

270 nm

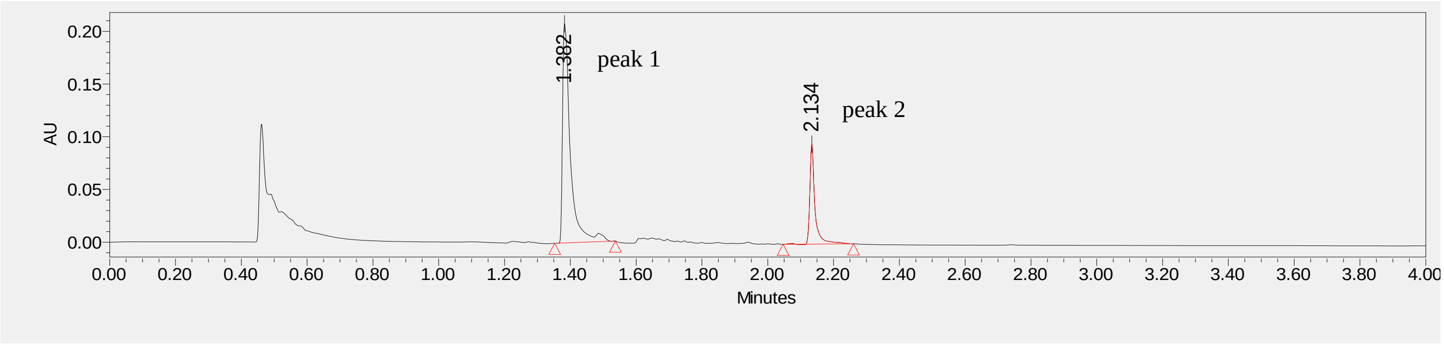

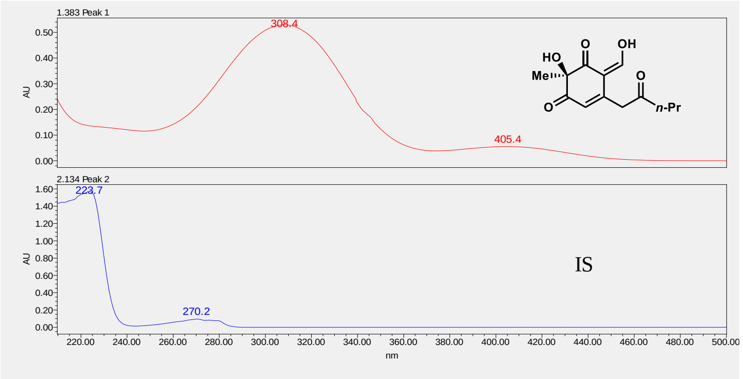

	Retention Time	Area	% Area
1	1.382	347905	76.79
2	2.134	105154	23.21

440 nm

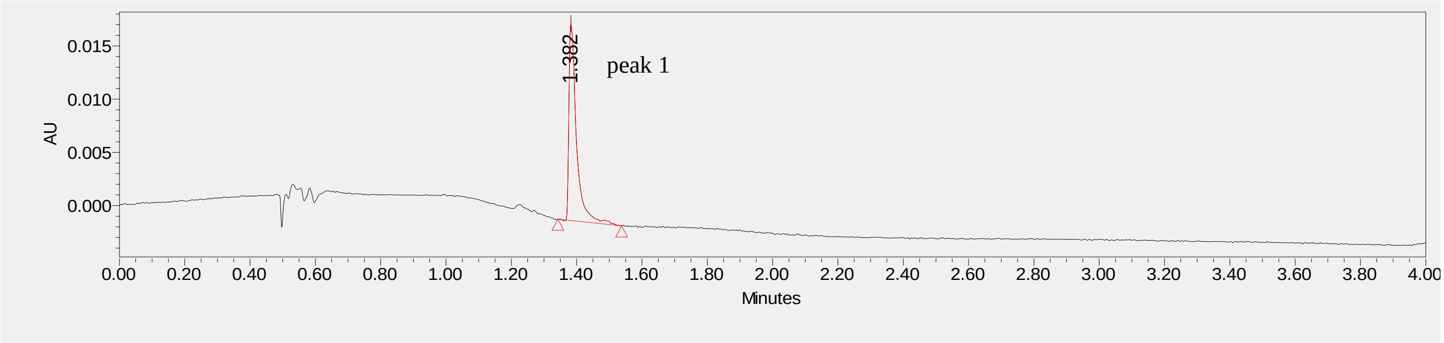

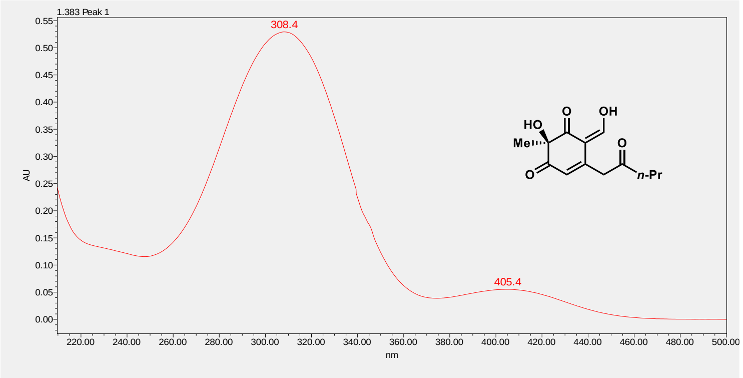

	Retention Time	Area	% Area
1	1.382	29723	100

Substrate **17** – step 2 with C7 thioester and MrPigD

270 nm:

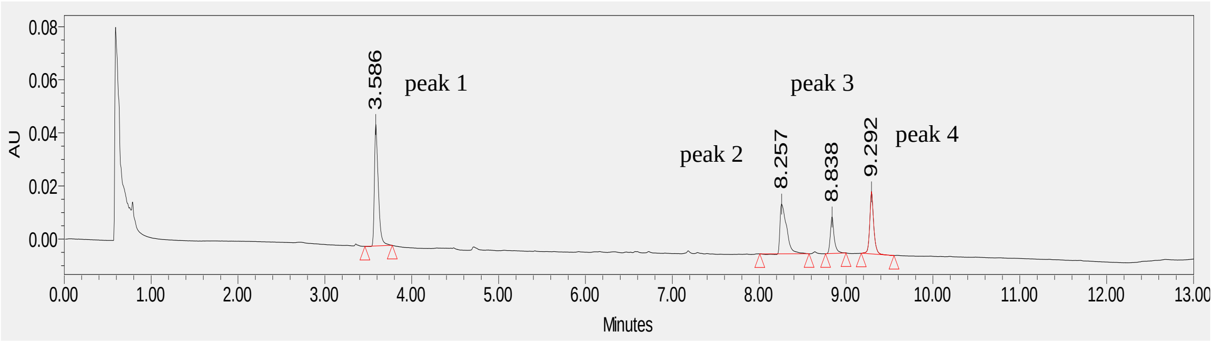

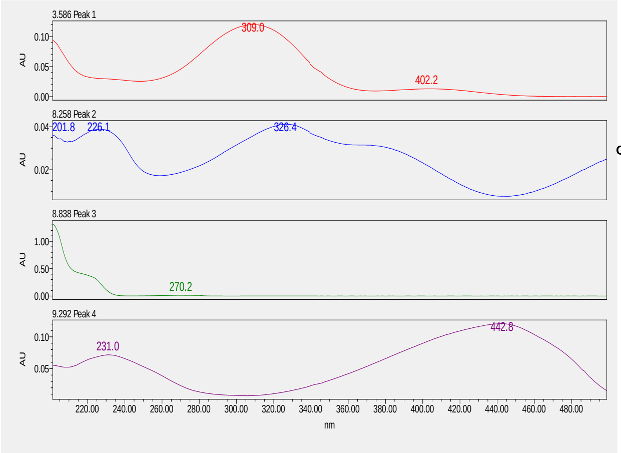

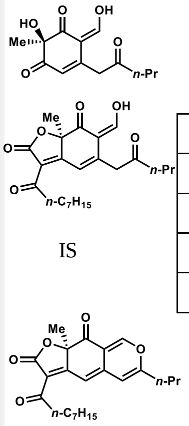

	Retention Time	Area	% Area
1	3.586	143209	39.64
2	8.257	101499	28.09
3	8.838	42147	11.66
4	9.292	74462	20.61

440 nm:

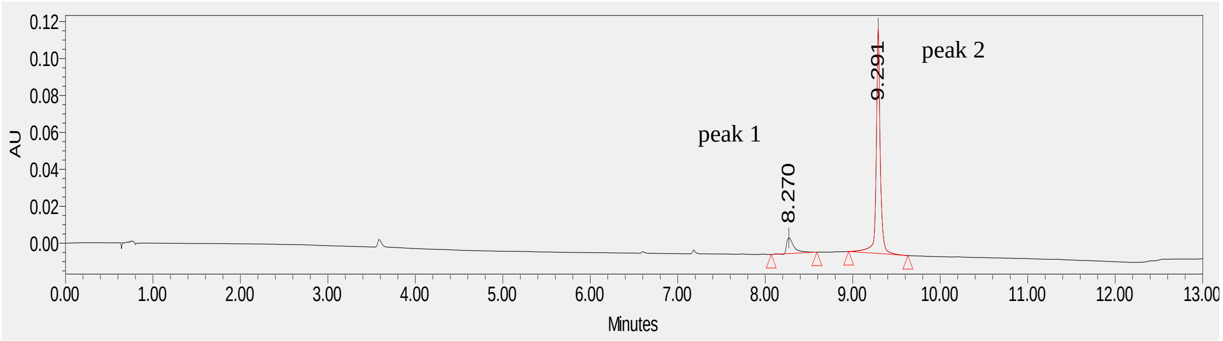

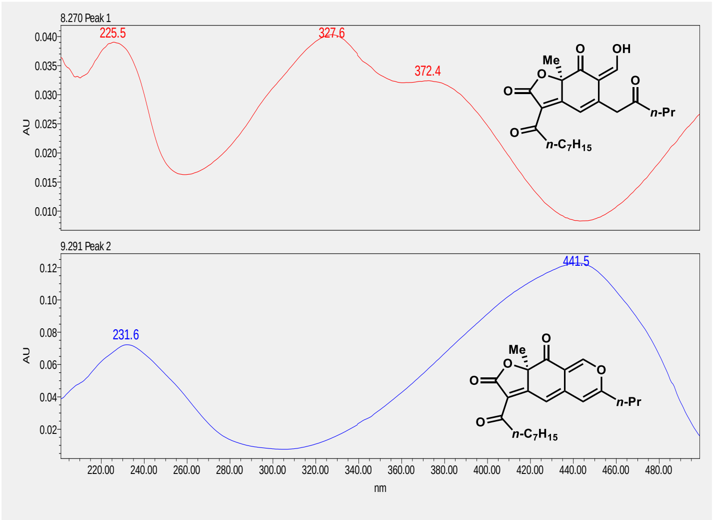

	Retention Time	Area	% Area
1	8.27	50952	11.53
2	9.291	391124	88.47

Substrate **17** – step 2 with C7 thioester and no enzyme control

270 nm

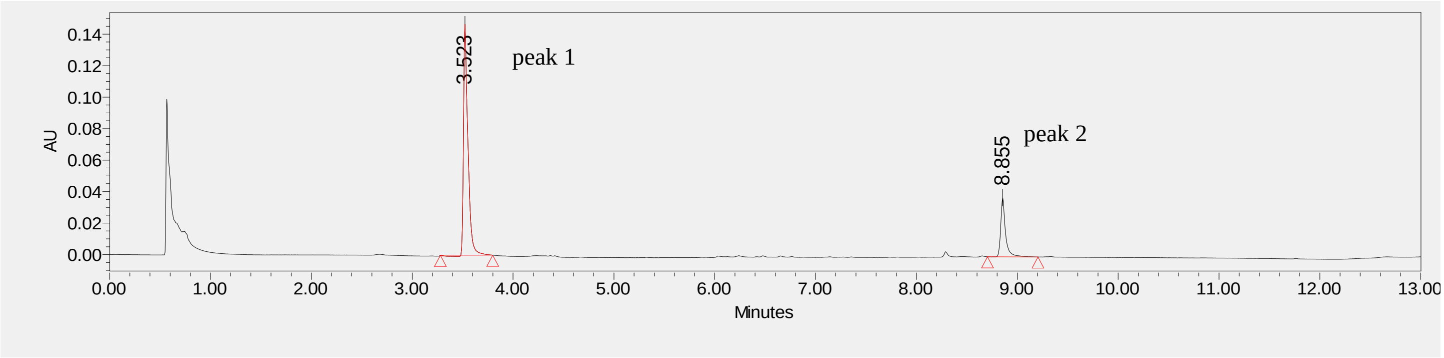

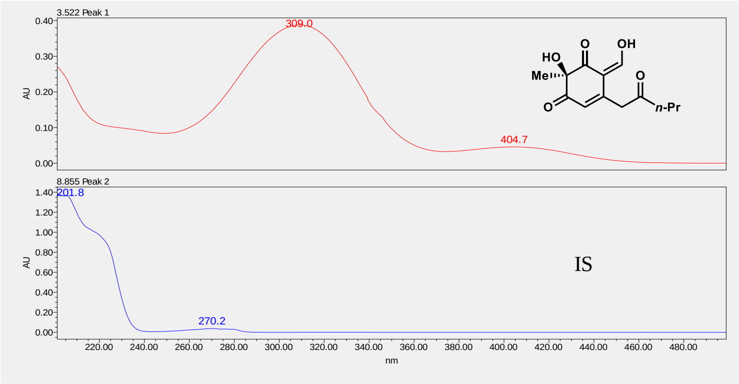

	Retention Time	Area	% Area
1	3.523	455001	78.64
2	8.855	123559	21.36

440 nm

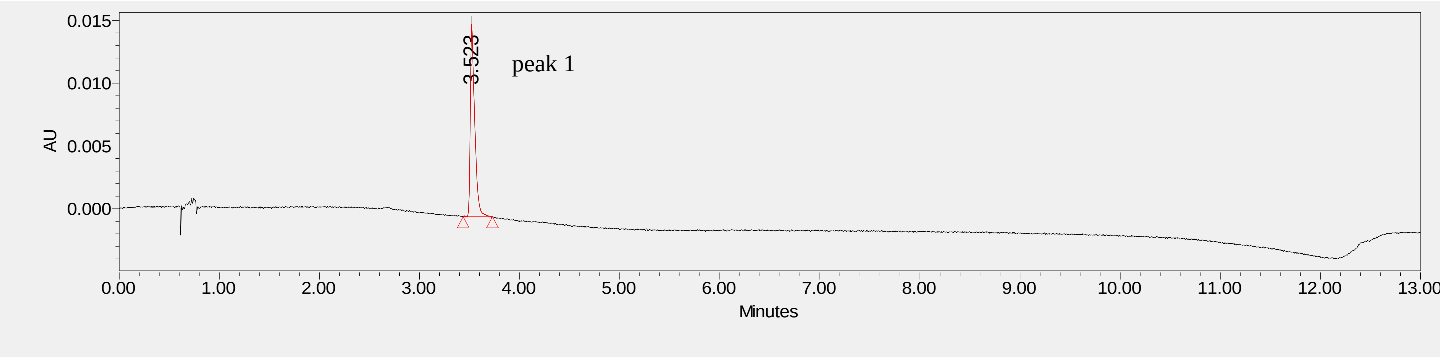

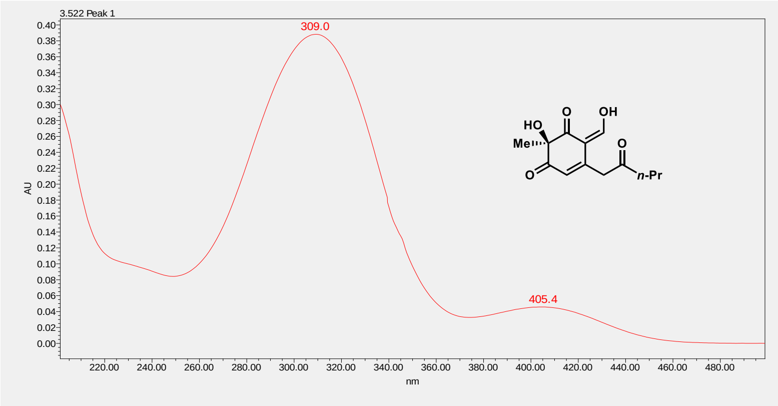

	Retention Time	Area	% Area
1	3.523	46394	100

Substrate **17** – step 2 with C9 thioester and MrPigD

270 nm:

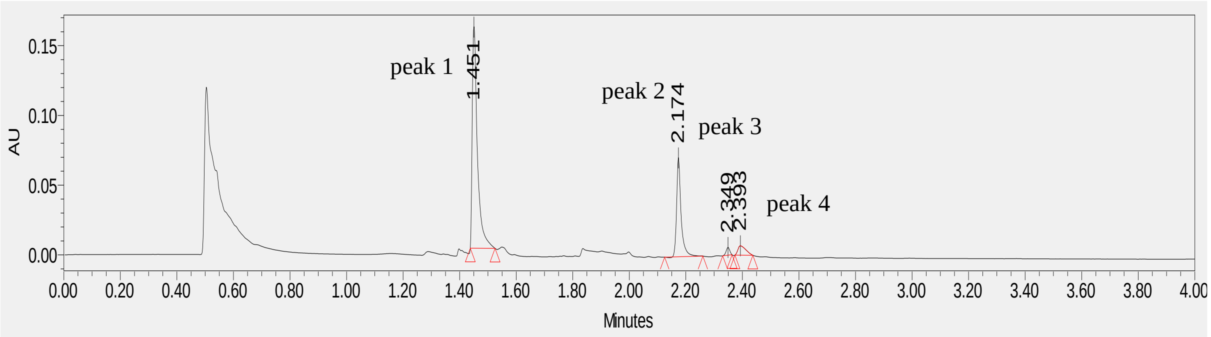

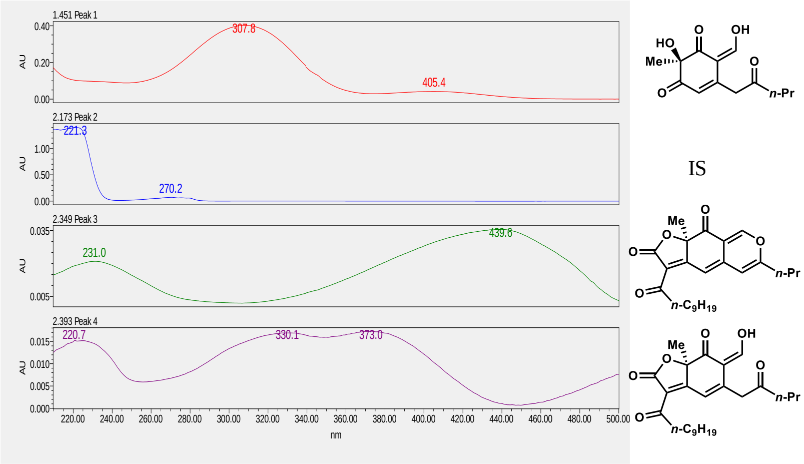

	Retention Time	Area	% Area
1	1.451	203130	67.74
2	2.174	78736	26.26
3	2.349	4947	1.65
4	2.393	13043	4.35

440 nm:

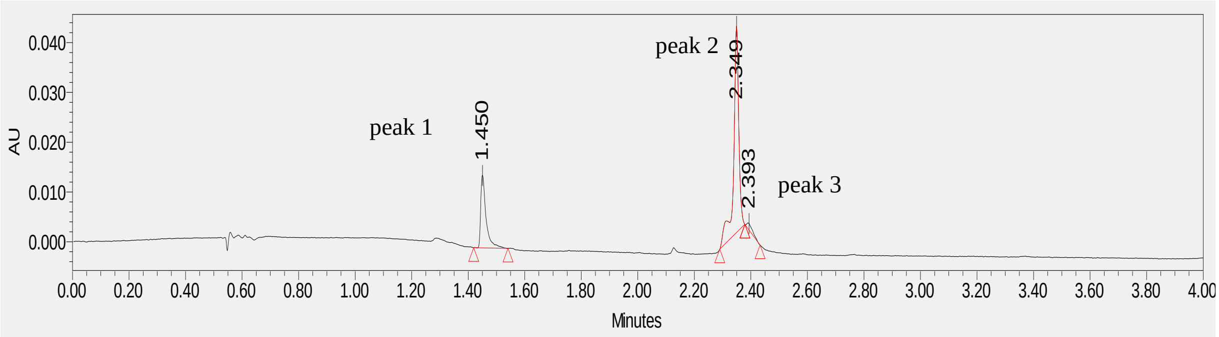

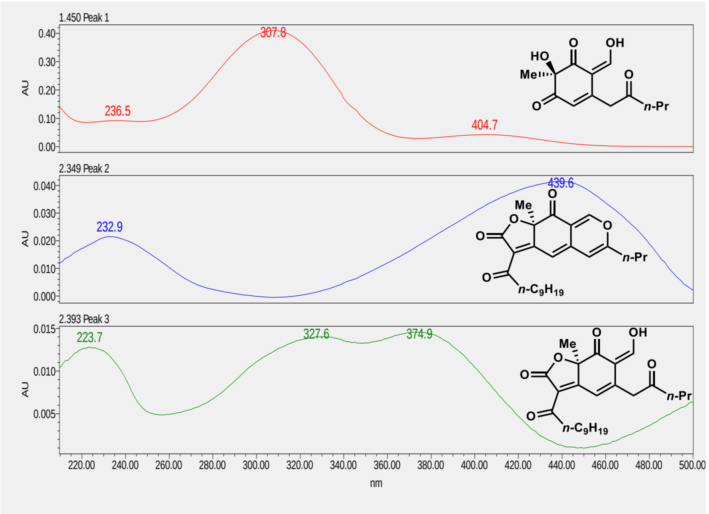

	Retention Time	Area	% Area
1	1.45	19205	26.18
2	2.349	52294	71.29
3	2.393	1858	2.53

Substrate **17** – step 2 with C9 thioester and no enzyme control

270 nm

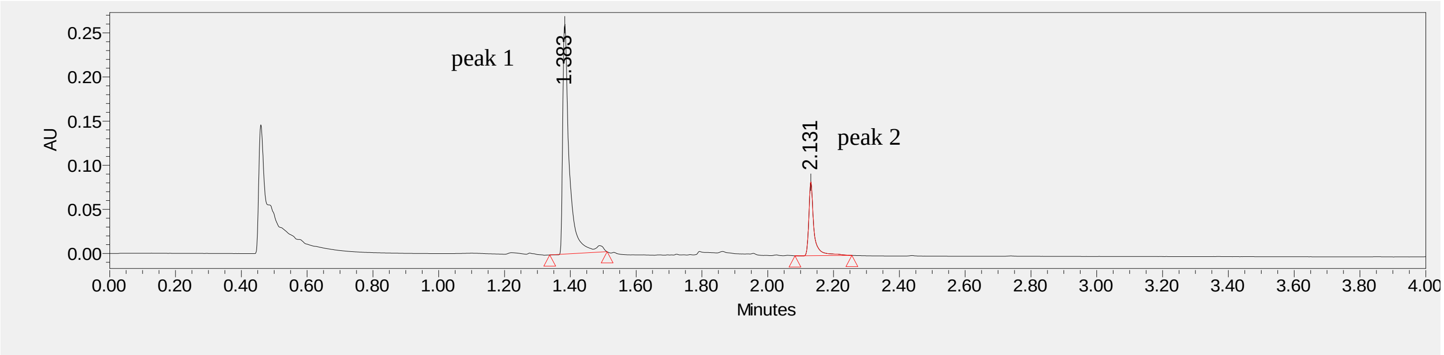

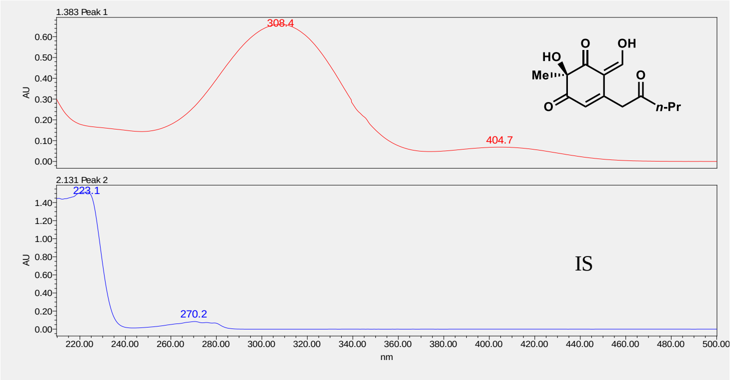

	Retention Time	Area	% Area
1	1.383	368872	79.96
2	2.131	92445	20.04

440 nm

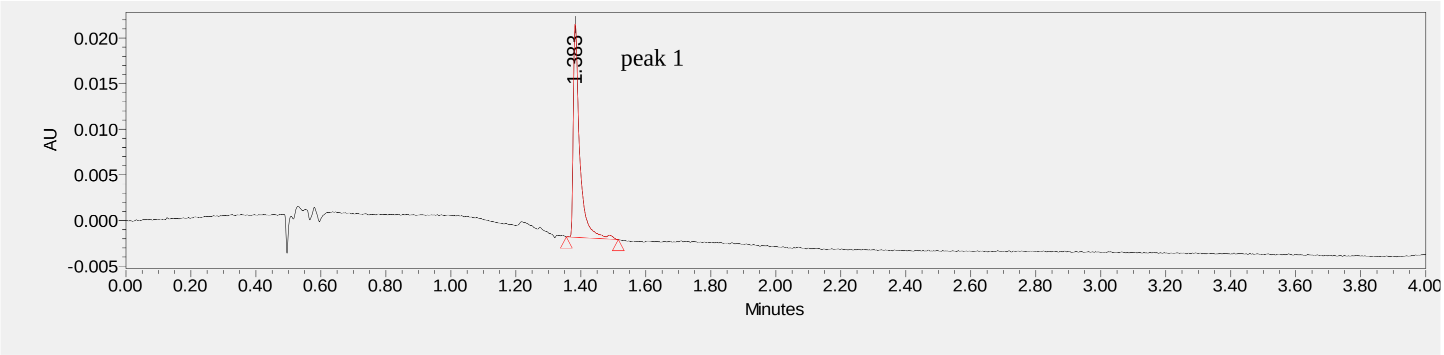

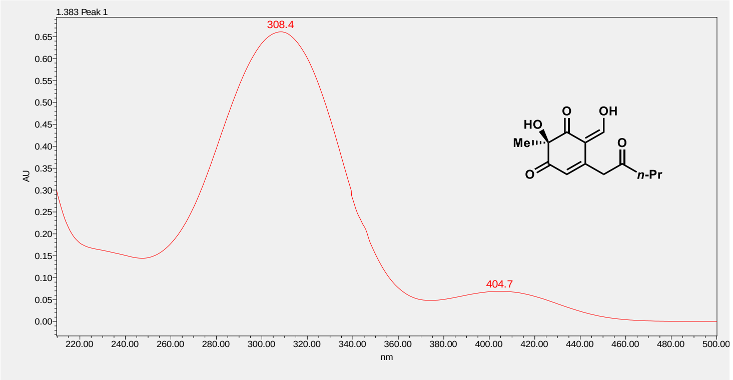

	Retention Time	Area	% Area
1	1.383	32153	100

Substrate **17** – step 2 with thiodiester and MrPigD

270 nm

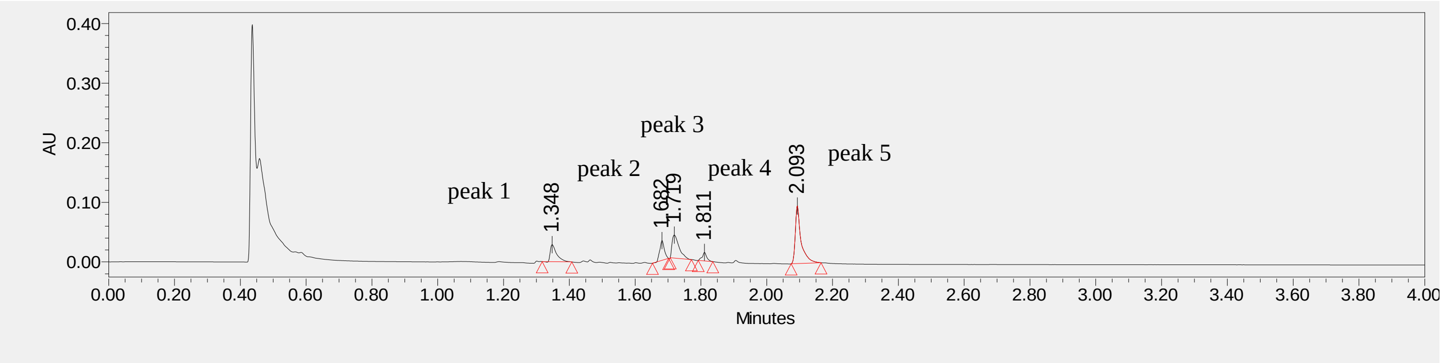

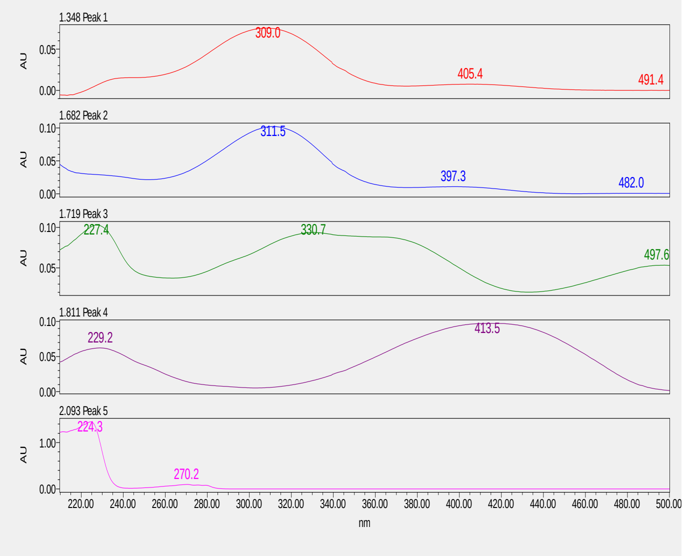

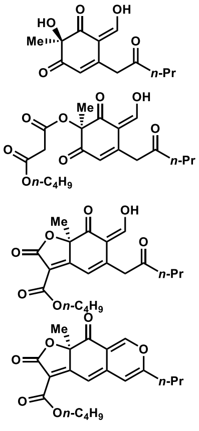

IS

	Retention Time	Area	% Area
1	1.348	39271	15.12
2	1.682	32068	12.35
3	1.719	57047	21.97
4	1.811	13405	5.16
5	2.093	117900	45.4

440 nm

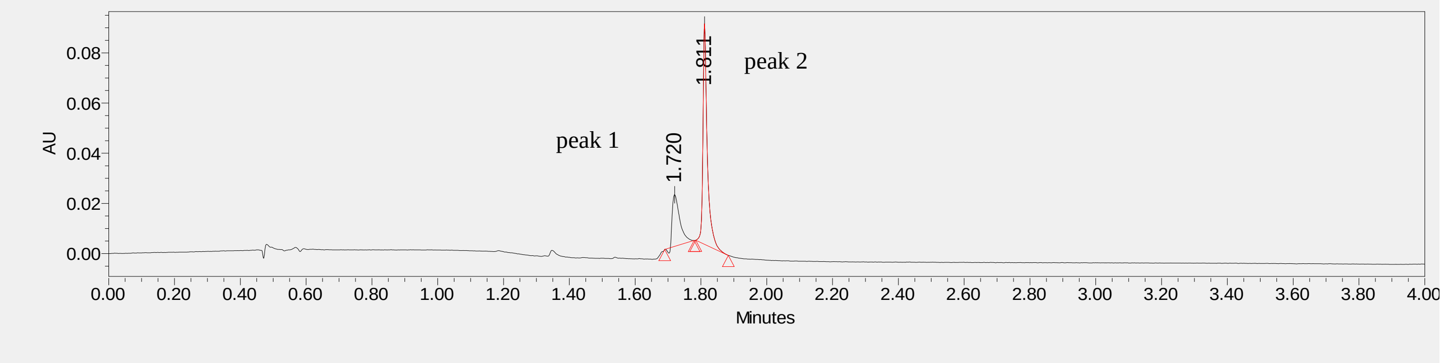

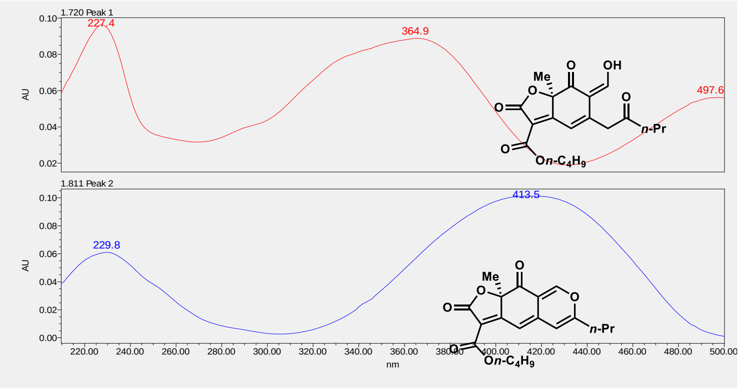

	Retention Time	Area	% Area
1	1.72	34104	29.51
2	1.811	81469	70.49

Substrate **17** – step 2 with with thiodiester and no enzyme control

270 nm

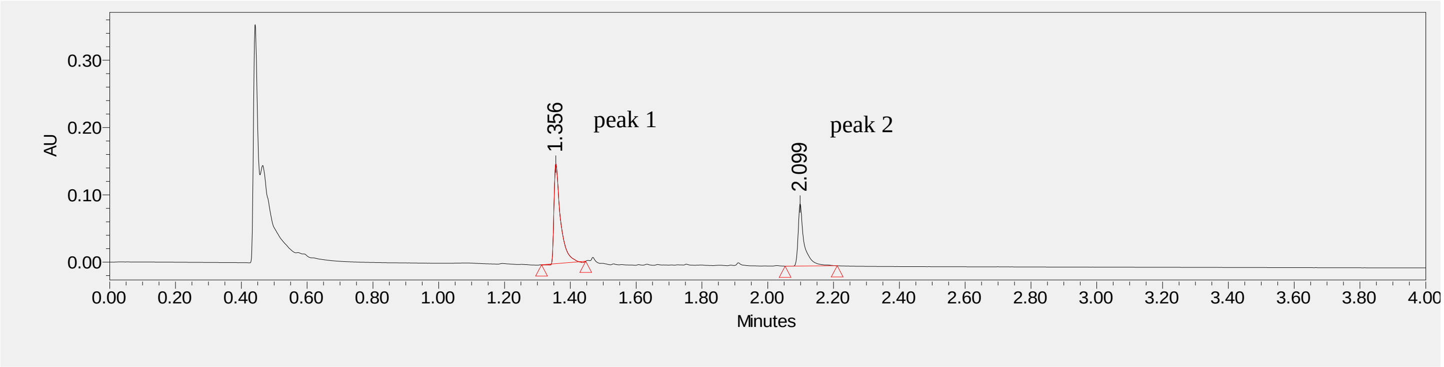

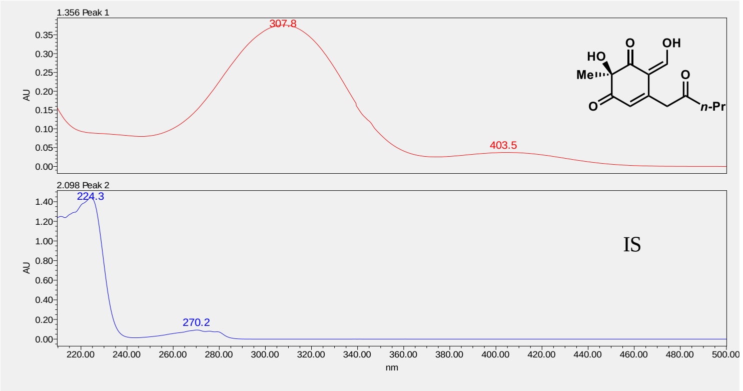

	Retention Time	Area	% Area
1	1.356	207874	63.75
2	2.099	118213	36.25

440 nm

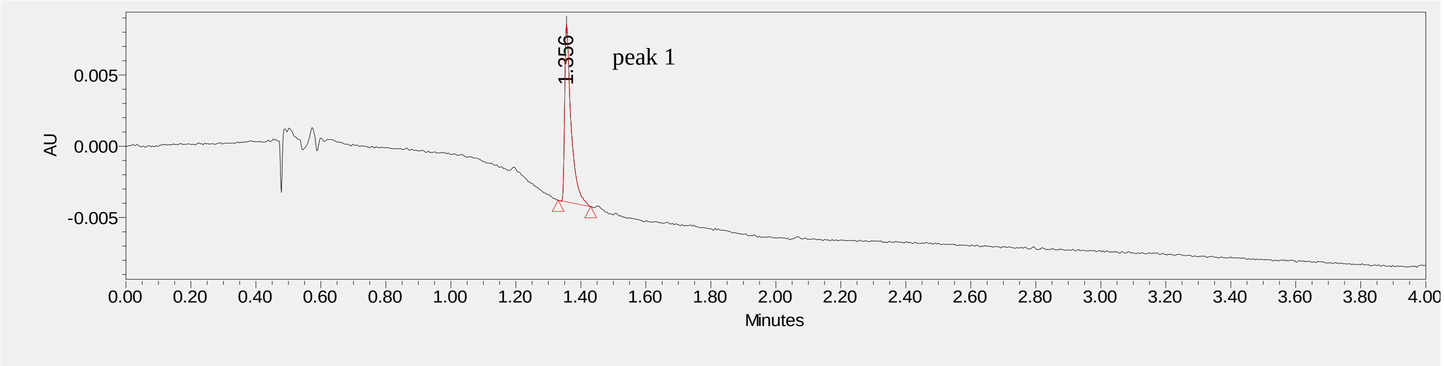

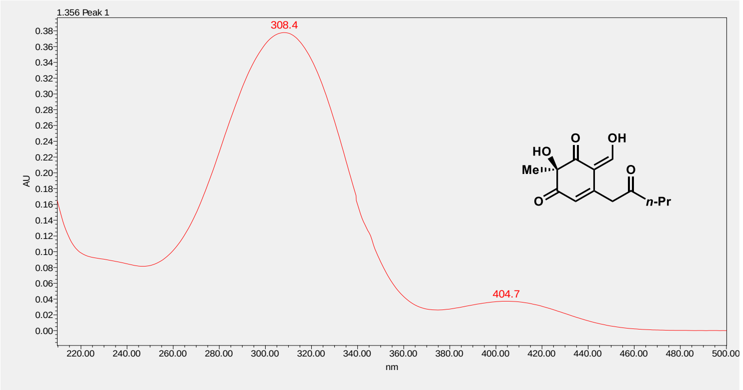

	Retention Time	Area	% Area
1	1.356	17097	100

Substrate **S2** – step 1 with AzaH

270 nm:

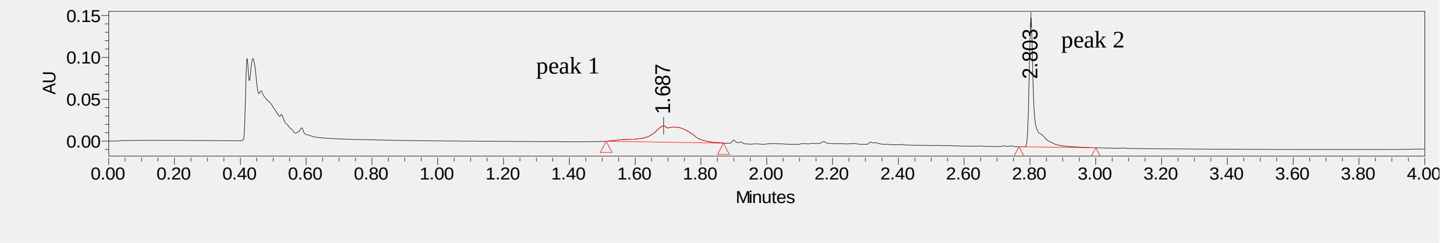

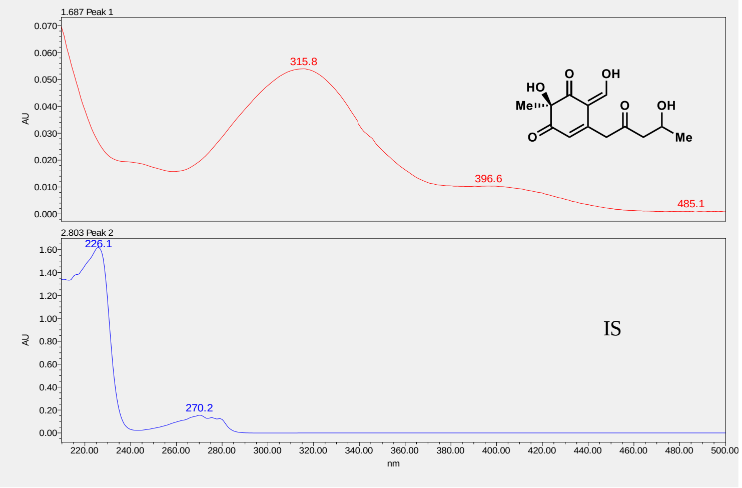

	Retention Time	Area	% Area
1	1.687	157851	47.82
2	2.803	172246	52.18

Substrate **S2** – step 1 no enzyme control

270 nm:

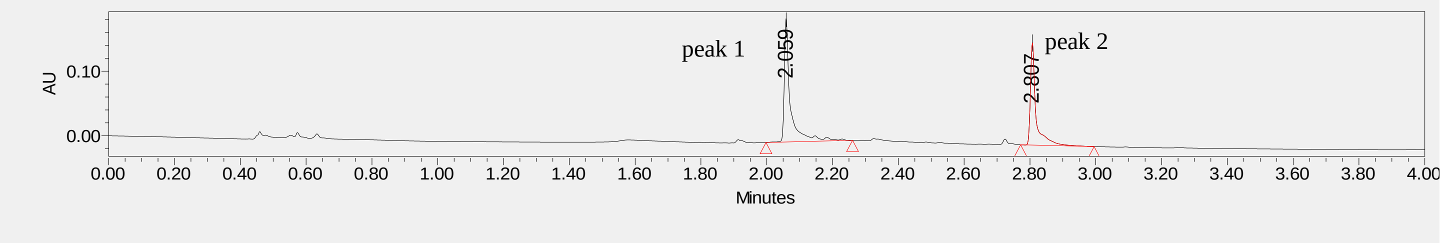

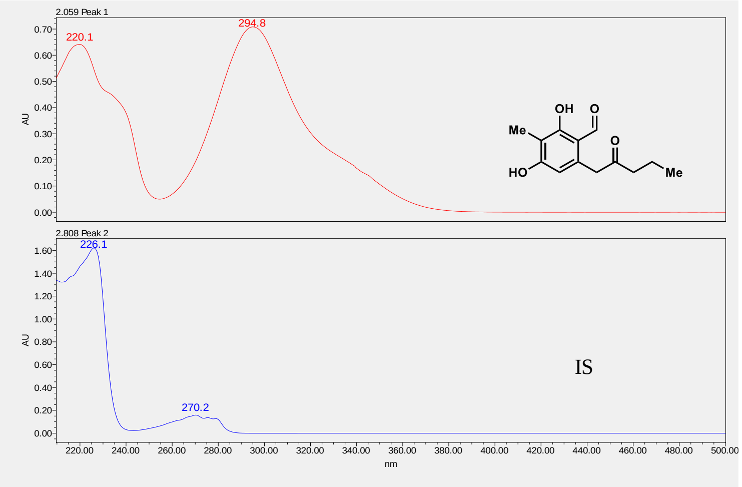

	Retention Time	Area	% Area
1	2.059	251345	57.34
2	2.807	187022	42.66

Substrate **S2** – step 2 with C5 thioester and MrPigD

270 nm

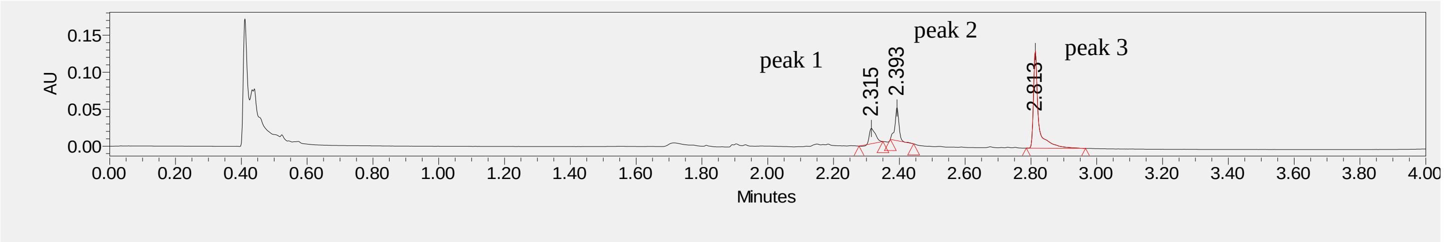

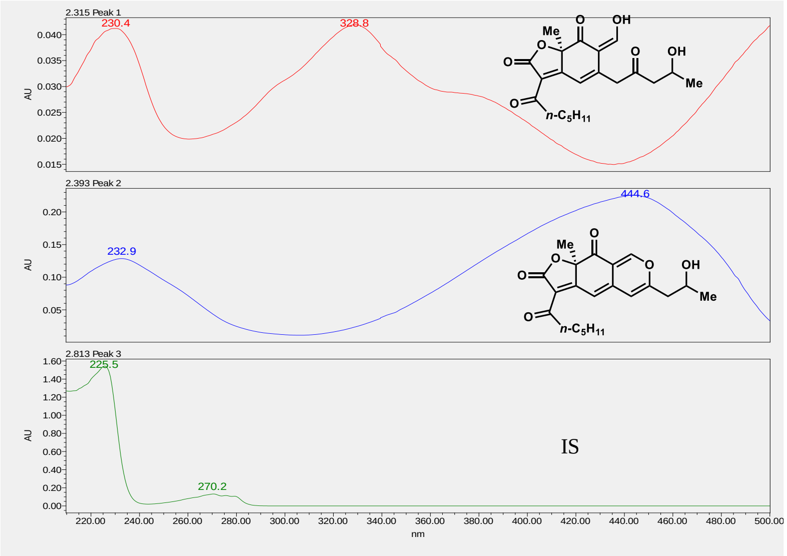

	Retention Time	Area	% Area
1	2.315	27819	13.35
2	2.393	38517	18.48
3	2.813	142051	68.17

440 nm

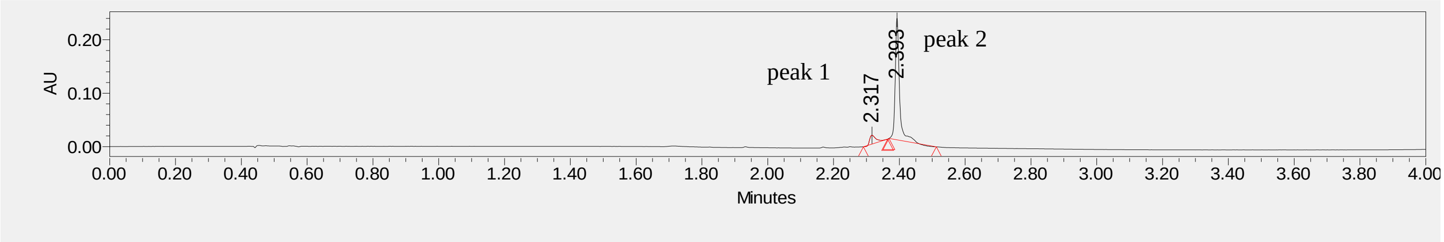

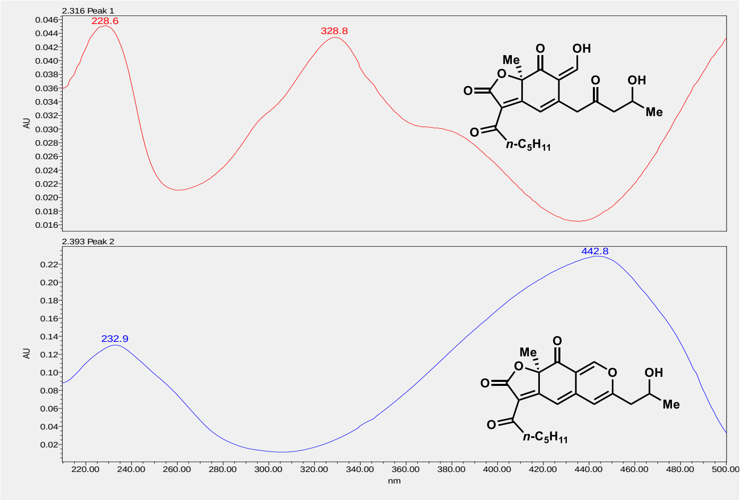

	Retention Time	Area	% Area
1	2.317	21643	9.24
2	2.393	212564	90.76

Substrate **S2** – step 2 with C5 thioester and no enzyme control

270 nm

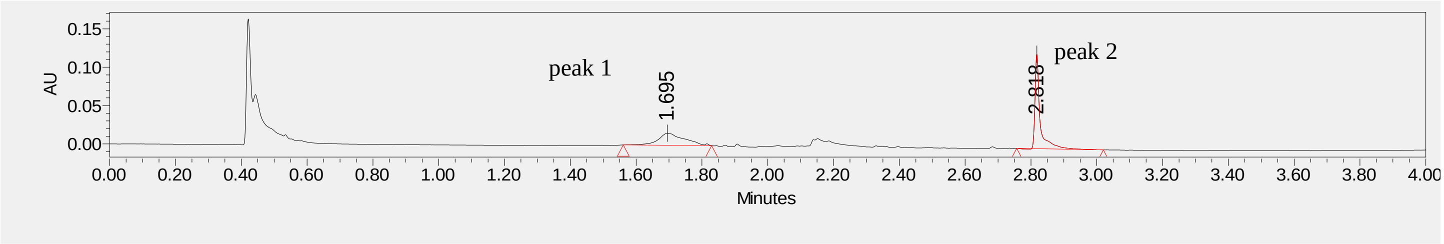

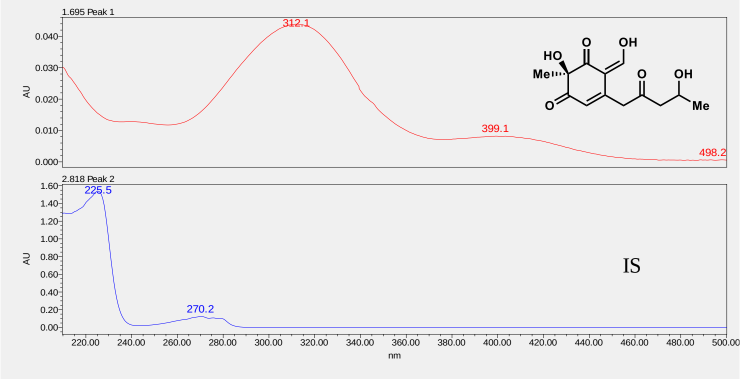

	Retention Time	Area	% Area
1	1.695	87022	39.23
2	2.818	134776	60.77

440 nm

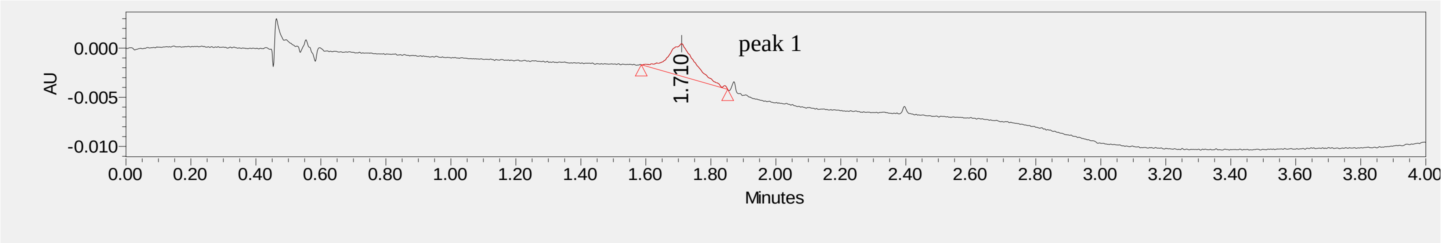

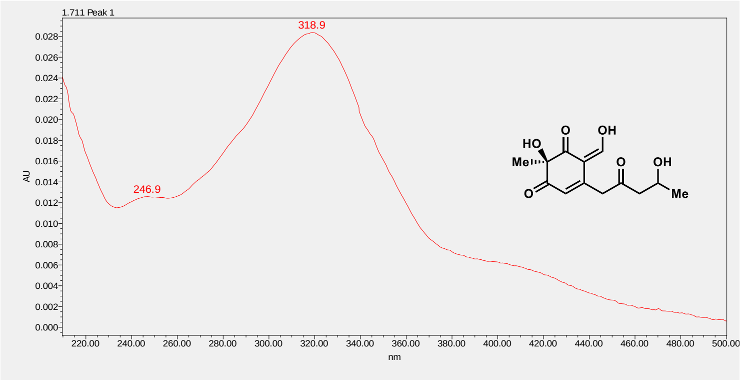

	Retention Time	Area	% Area
1	1.71	19520	100

Substrate **S3** – step 1 with AzaH

270 nm:

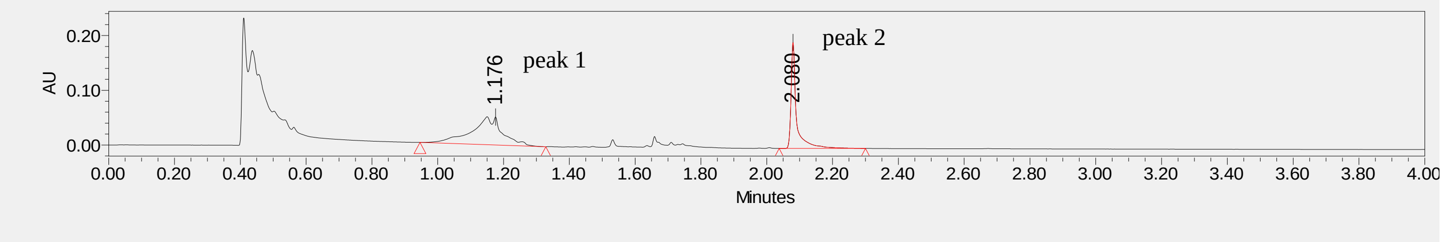

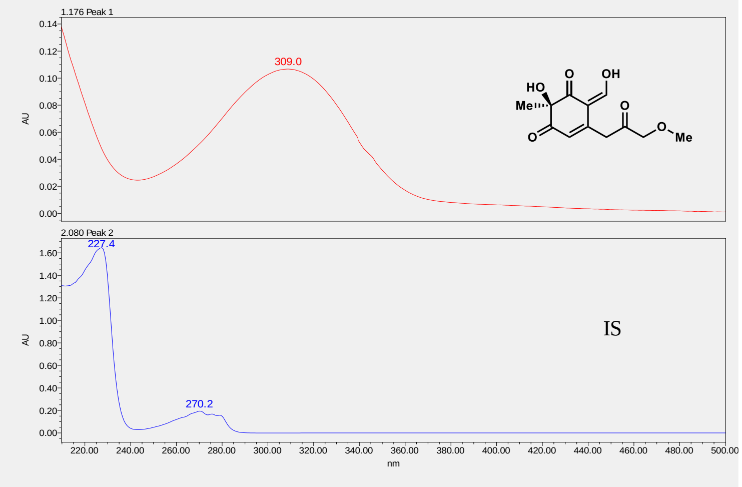

	Retention Time	Area	% Area
1	1.176	334944	60.03
2	2.08	223020	39.97

Substrate **S3** – step 1 no enzyme control

270 nm:

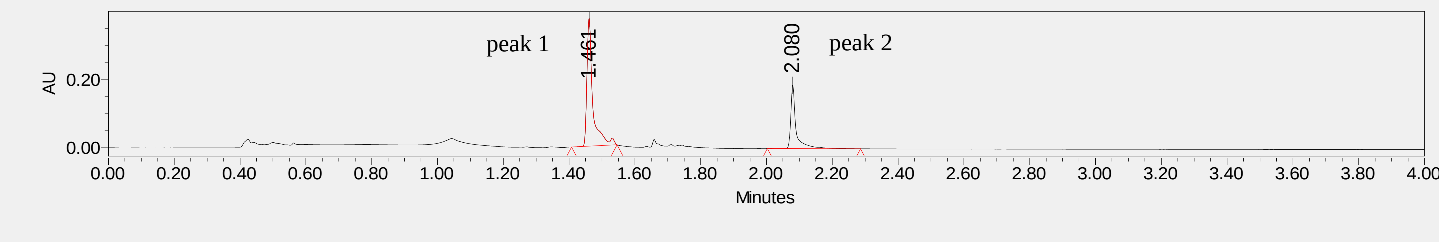

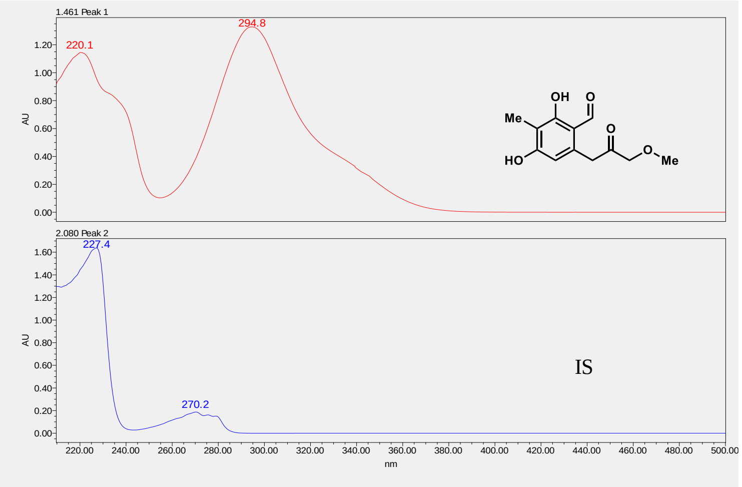

	Retention Time	Area	% Area
1	1.461	471745	68.9
2	2.08	212918	31.1

Substrate **S3** – step 2 with C5 thioester and MrPigD

270 nm

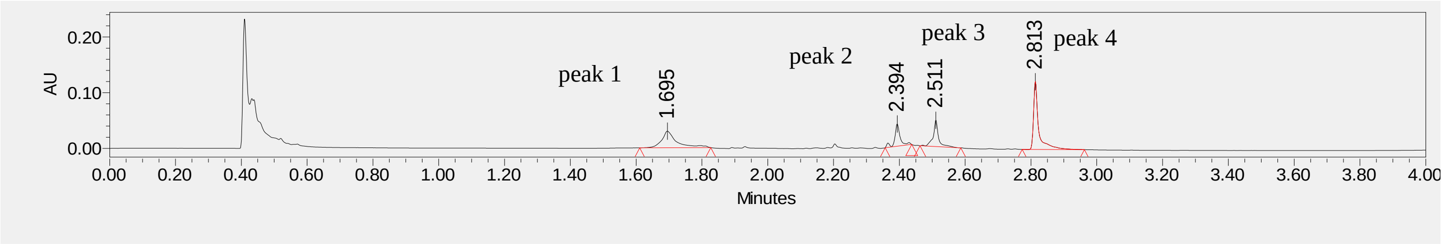

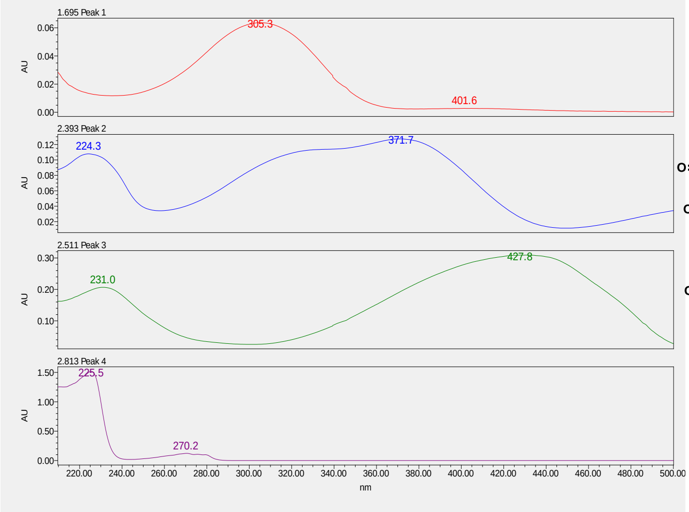

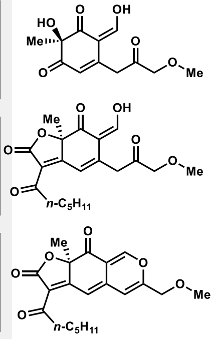

	Retention Time	Area	% Area
1	1.695	95493	29.11
2	2.394	45490	13.87
3	2.511	53964	16.45
4	2.813	133083	40.57

440 nm

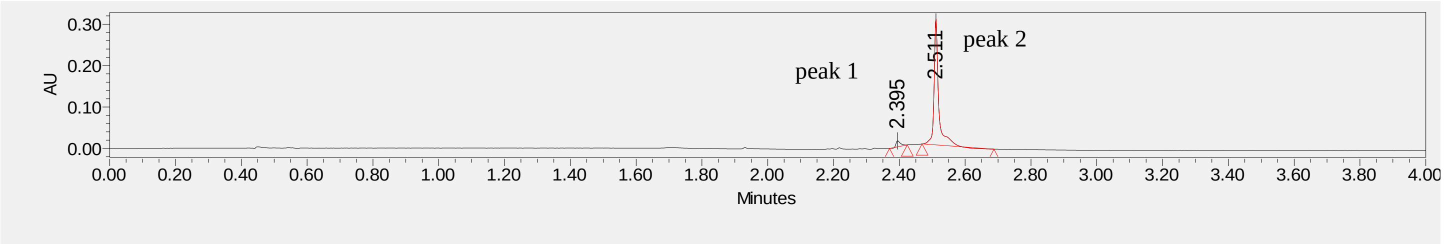

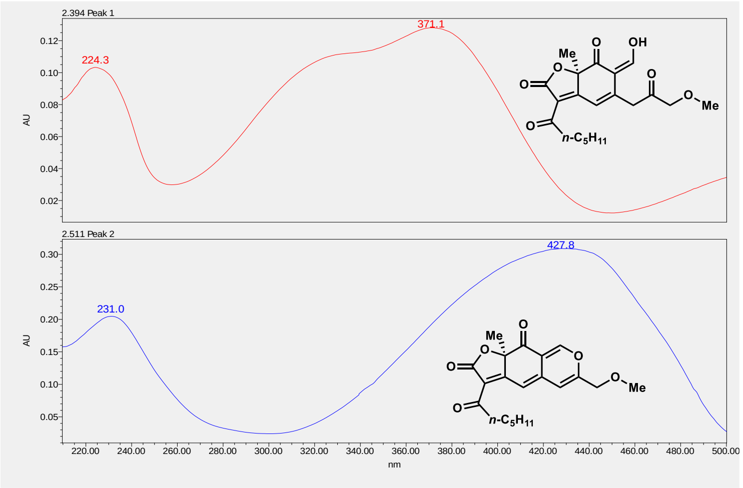

	Retention Time	Area	% Area
1	2.395	13890	4.38
2	2.511	303218	95.62

Substrate **S3**– step 2 with C5 thioester and no enzyme control

270 nm

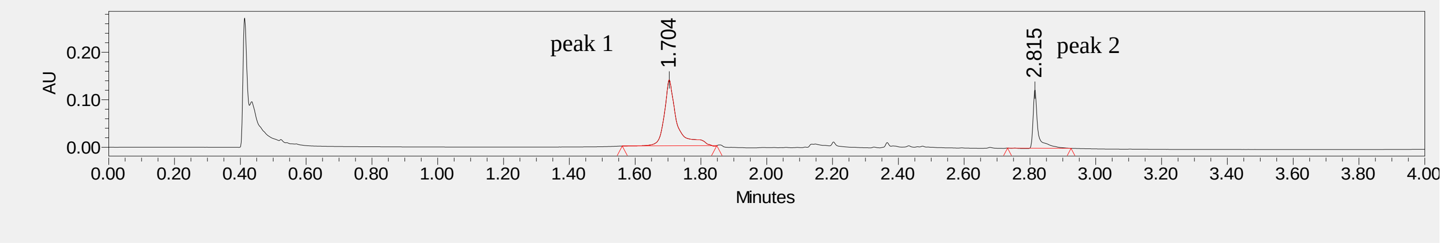

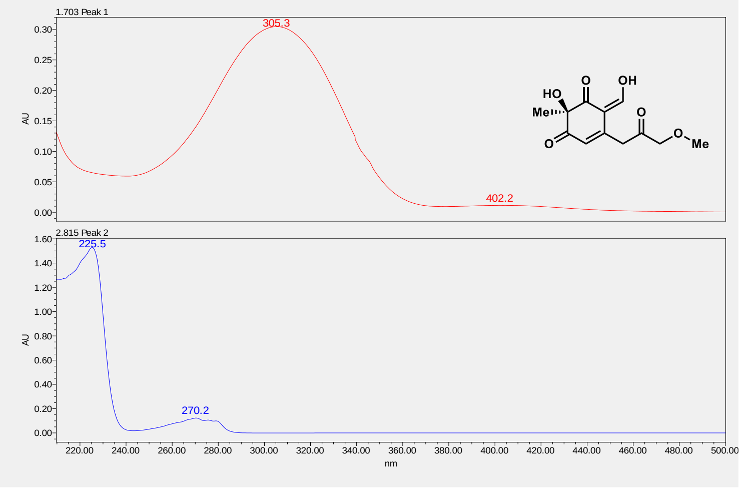

	Retention Time	Area	% Area
1	1.704	370495	73.95
2	2.815	130487	26.05

440 nm

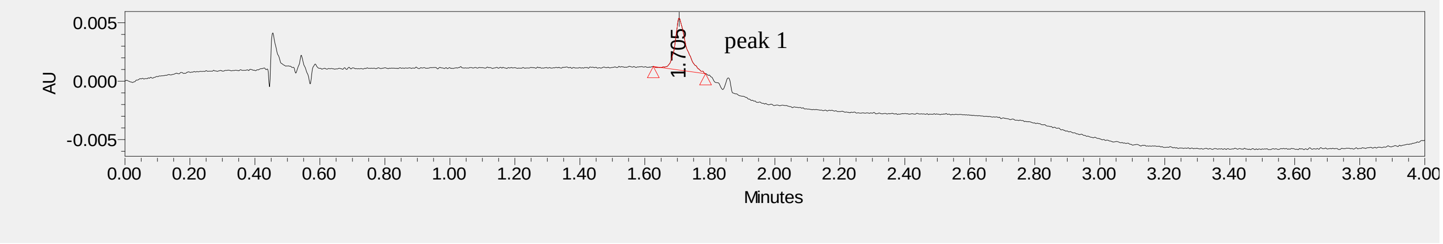

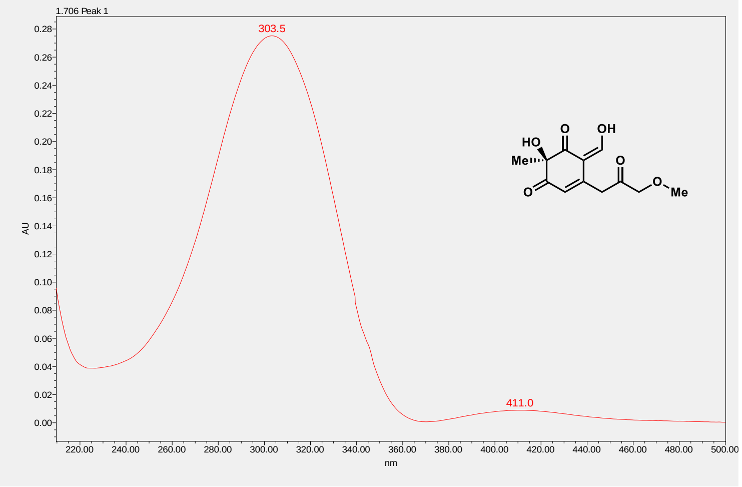

	Retention Time	Area	% Area
1	1.705	11056	100

Substrate **S4** – step 1 with AzaH

270 nm:

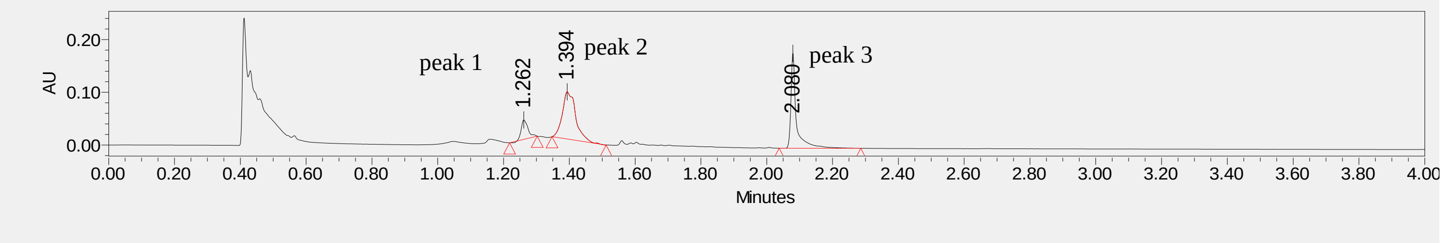

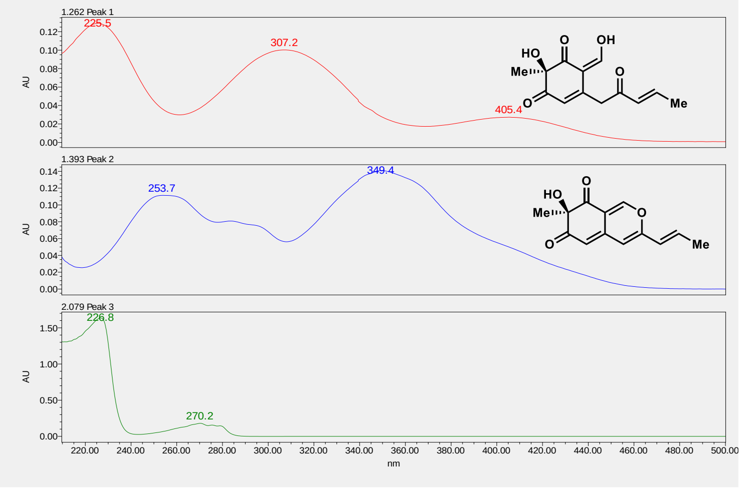

	Retention Time	Area	% Area
1	1.262	54919	10.6
2	1.394	256166	49.44
3	2.08	207026	39.96

Substrate **S4** – step 1 no enzyme control

270 nm:

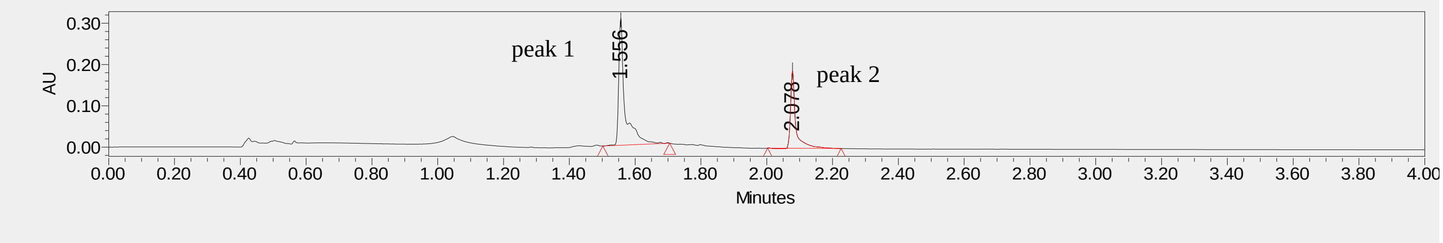

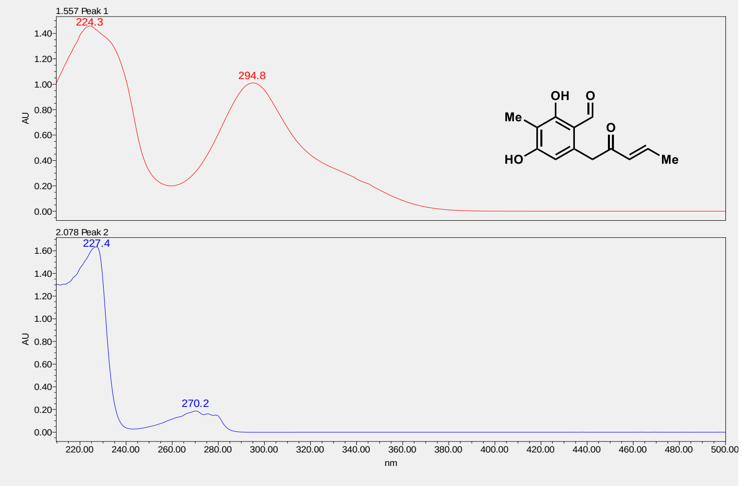

	Retention Time	Area	% Area
1	1.556	411776	66.4
2	2.078	208401	33.6

Substrate **S4** – step 2 with C5 thioester and MrPigD

270 nm

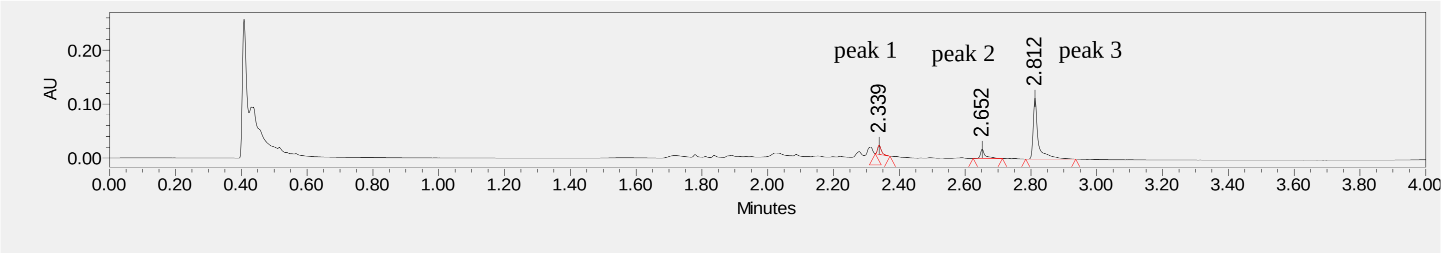

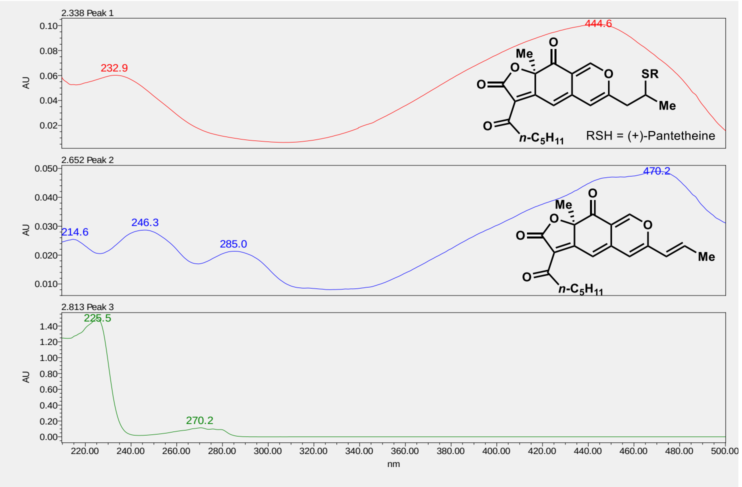

	Retention Time	Area	% Area
1	2.339	13377	8.83
2	2.652	17475	11.53
3	2.812	120658	79.64

440 nm

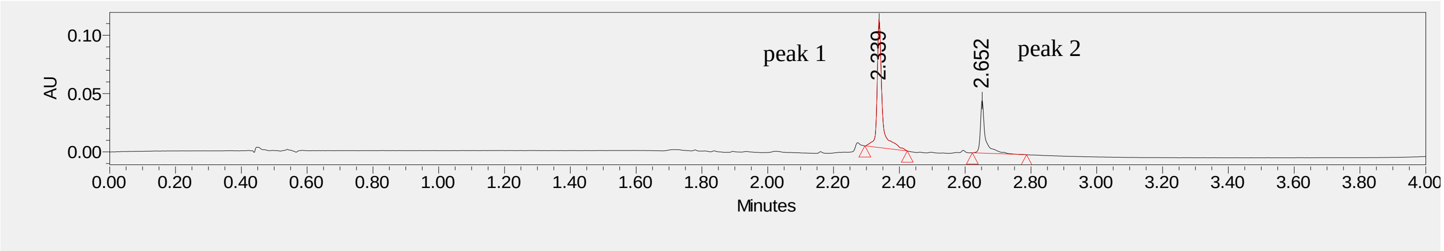

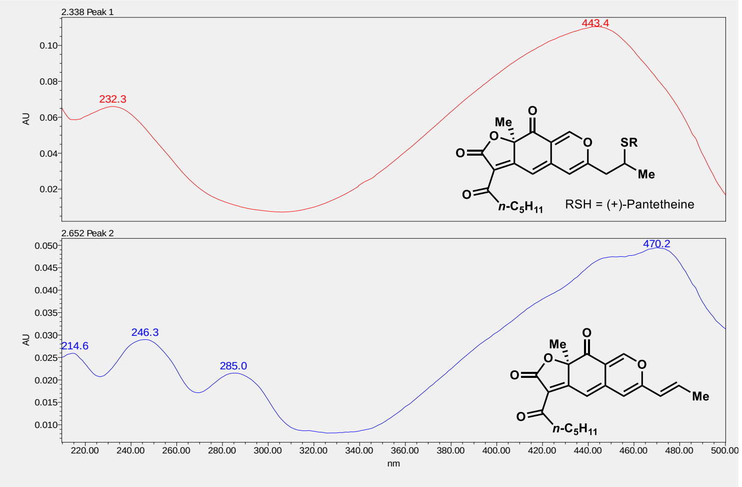

	Retention Time	Area	% Area
1	2.339	118622	71.34
2	2.652	47663	28.66

Substrate **S4** – step 2 with C5 thioester and no enzyme control

270 nm

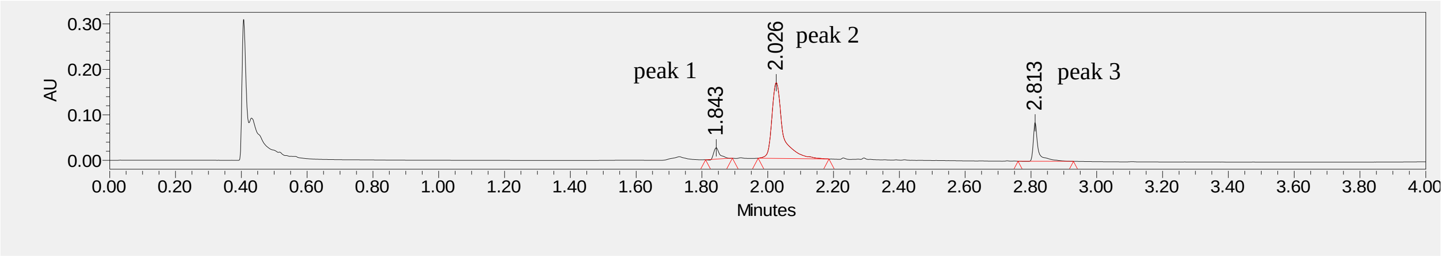

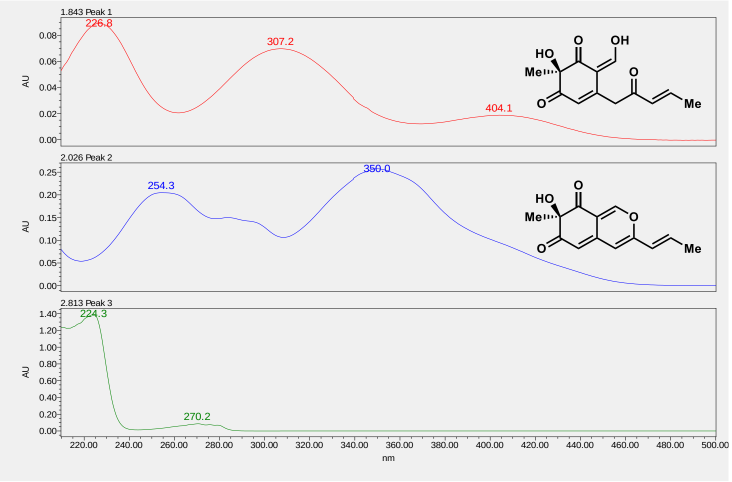

	Retention Time	Area	% Area
1	1.843	32119	6.29
2	2.026	387566	75.95
3	2.813	90609	17.76

440 nm

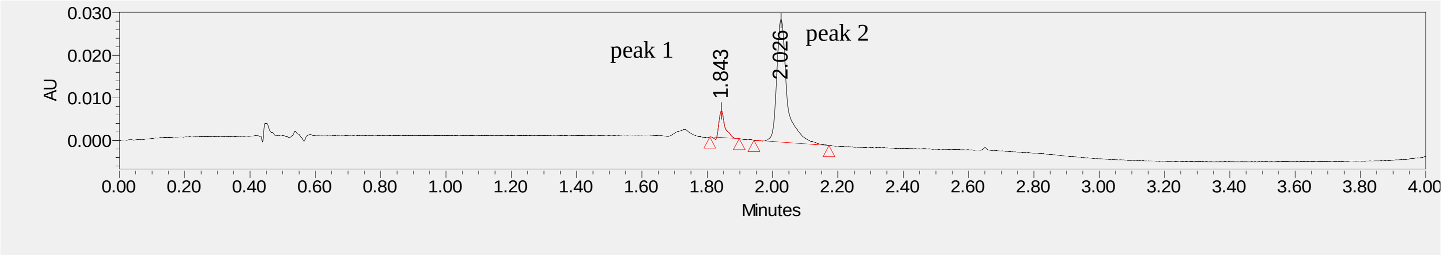

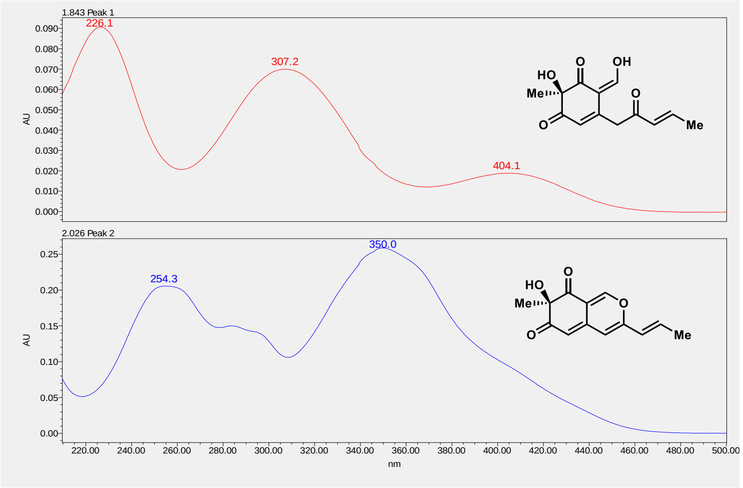

	Retention Time	Area	% Area
1	1.843	8206	11.26
2	2.026	64688	88.74

XI. Preparative-scale biocatalytic reactions of Substrate S4 with C5(18) and C7 (S11) thioester.

2.5 mM orcinaldehyde substrate (400 µL of a 50 mM stock solution in DMSO), 5 mM G6P (80 µL, 500 mM), 1 mM NADP+ (80 µL, 100 mM), 1 U/mL G6P-DH (80 µL, 100 U/mL), 10 µM AzaH (535 µL of a 150 µM stock solution) was added to 6.51 mL of 50 mM potassium phosphate buffer, pH 8.0. The reaction was placed in an incubator at 30 °C with 100 rpm shaking for 1 h. Once the starting material had disappeared via UPLC, 2.75 mM thioester **18** or **S11** (220 µL of a 100 mM stock solution in DMSO), 5 mM maleimide (thiol scavenger, 40 µL 1 M stock solution in DMSO) 20 µM MrPigD (95 µL of a 1.7 mM stock solution) were then added, and the reaction was returned to the 30 °C for 20 min. The reaction was then diluted with ethyl acetate (~3x reaction volume) and extracted 3 times with ~10 mL EtOAc. The combined organic layers were washed with ~30 mL of brine, then dried with sodium sulfate. The sodium sulfate and precipitated biomolecules were removed through gravity filtration and the flow-through was passed through a 0.22 µm filter before the solvent was removed using a rotary evaporator. The resulting crude oil was dissolved in 1 mL µL of HPLC grade MeOH and subjected to purification by preparative HPLC using a Phenomenex Kinetex 5 µm C18, 150 x 21.2 mm column under the following conditions: mobile phase A = deionized water and B = acetonitrile; method = 5% B hold 1 min,5% to 20% B over 2 min, 20% to 50% B over 5 min, 50% to 90% B over 9 min, 90% to 100% B over 5 min, 100% B for 3 min; flow rate, 10 mL/min. Fractions containing the purified compound were pooled and the solvent was removed using a rotary evaporator.

Standard curve for conversion calculation:

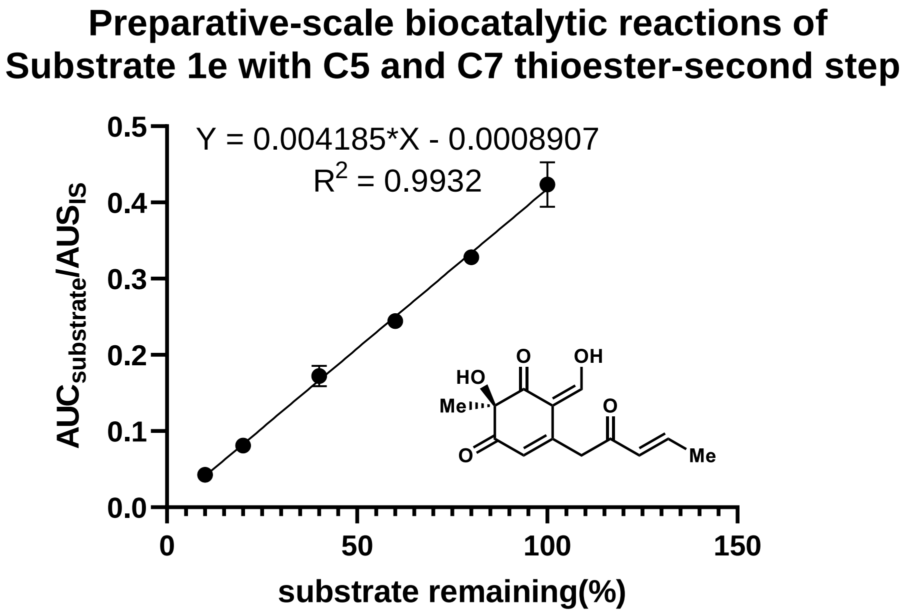

Reaction trace:

Substrate **S4** with **C5** thioester (**18**):

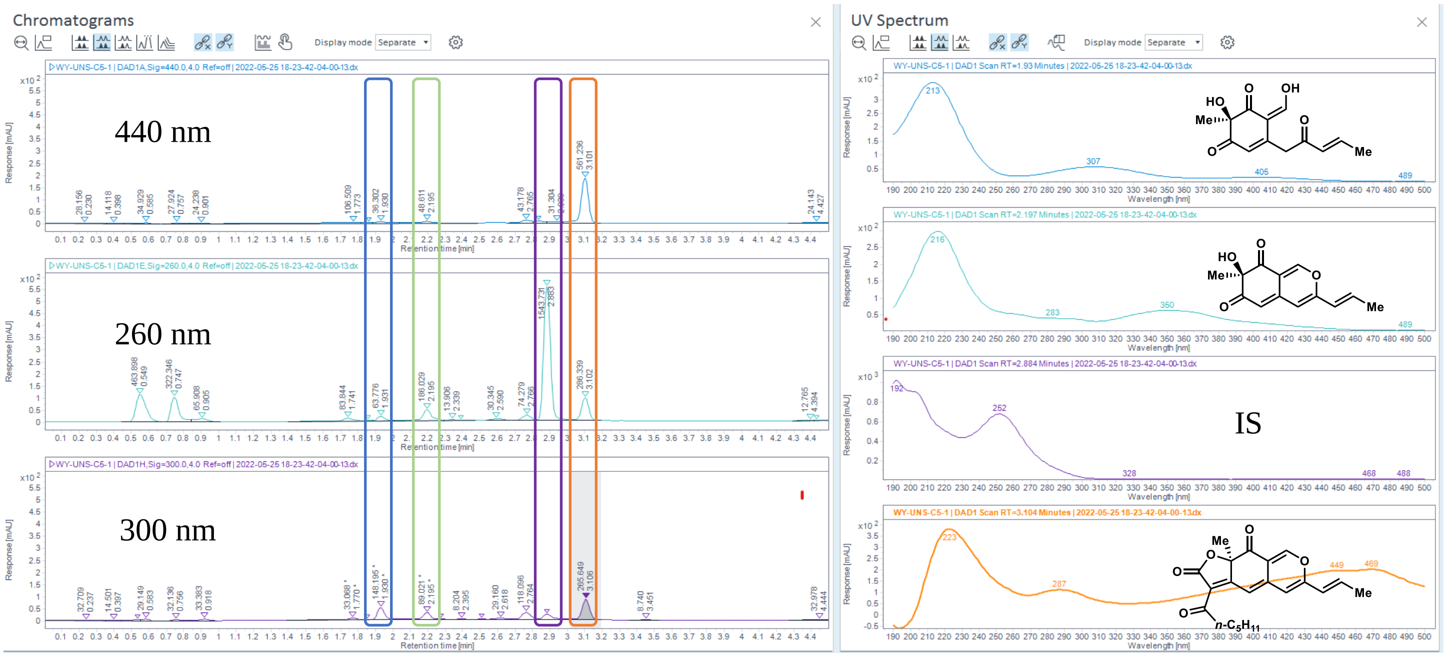

Substrate **S4** with **C7** thioester (**S11**):

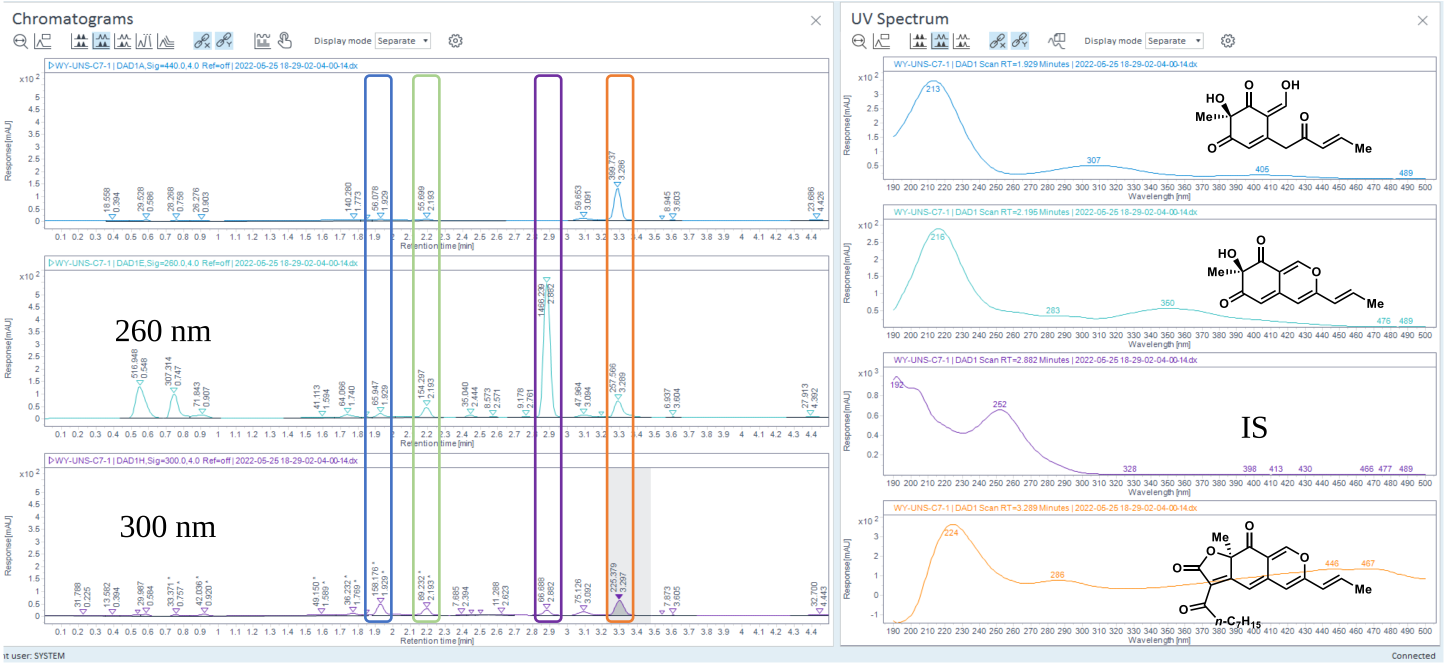

XII. Natural product NMR comparison and assignment of absolute configurations

i. Rubropunctatin NMR comparison table

Rubropunctatin ^{1}H NMR comparison table:

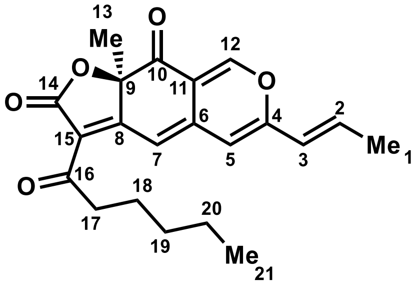

Rubropunctatin

rubropunctatin-^{1}H NMR					
Reported data[6]			My data		Difference
no.	peak detail	Signal	peak detail	Signal	
1	1.95 (d, *J* = 8.4 Hz, 3H)	1.95	1.95 (dd, *J* = 6.6, 1.2 Hz, 3H)	1.95	0.00
2	6.59 (m, 1H)	6.59	6.59 (dq, *J* = 15.6, 6.6 Hz, 1H)	6.59	0.00
3	6.04 (d, *J* = 15.6 Hz, 1H)	6.04	6.04 (dd, *J* = 15.6, 1.2 Hz, 1H)	6.04	0.00
5	6.14 (s, 1H)	6.14	6.14 (s, 1H)	6.14	0.00
7	6.89 (s, 1H)	6.89	6.89 (s, 1H)	6.89	0.00
12	7.86 (s, 1H)	7.86	7.86 (s, 1H)	7.86	0.00
13	1.71 (s, 3H)	1.71	1.71 (s, 3H)	1.71	0.00
17	2.99-2.89 (m, 2H)	2.94	2.99-2.89 (m, 2H)	2.94	0.00
18	1.62-1.58 (m, 2H)	1.60	1.64-1.59 (m, 2H)	1.61	0.01
19 and 20	1.33-1.31 (m, 4H)	1.32	1.33-1.31 (m, 4H)	1.32	0.00
21	0.88 (t, *J* = 6.6 Hz, 3H)	0.88	0.88 (t, *J* = 6.9 Hz, 3H)	0.88	0.00

Rubropunctatin ^{13}C NMR comparison table:

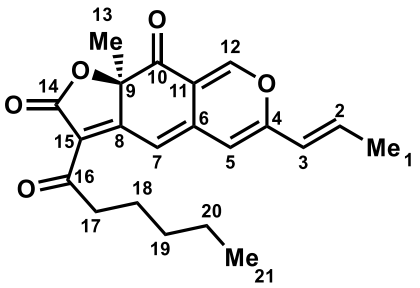

Rubropunctatin

rubropunctatin-^{13}C NMR			
reported data[7]		My data	Difference
no.	peak detail	peak detail	
1	18.7	18.9	0.2
2	136.4	136.5	0.1
3	122.4	122.5	0.1
4	156.4	156.5	0.1
5	109.5	109.7	0.2
6	141.6	141.7	0.1
7	104.2	104.3	0.1
8	113.2	113.4	0.2
9	85.8	85.9	0.1
10	190.8	190.9	0.1
11	116.3	116.4	0.1
12	152.8	152.9	0.1
13	28.3	28.5	0.2
14	171.6	171.8	0.2
15	169.2	169.4	0.2
16	197.4	197.6	0.2
17	41.6	41.8	0.2
18	23.4	23.5	0.1
19	31.4	31.5	0.1
20	22.5	22.7	0.2
21	13.9	14.1	0.2

ii. ***Monascorubrin NMR comparison table:***

Monascorubrin ^{1}H NMR comparison table:

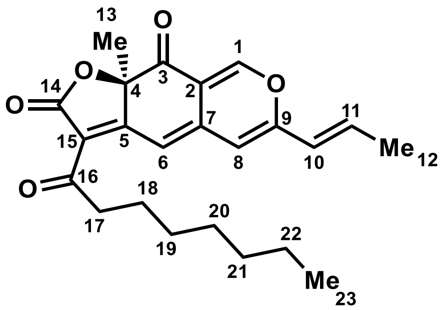

Monascorubrin

monascorubrin-^{1}H NMR					
**Reported data**[8]			**My data**		**Difference**
no.	**peak detail**	**Signal**	**peak detail**	**Signal**	
1	7.86 (s, 1H)	7.86	7.86 (s, 1H)	7.86	0.00
6	6.88 (s, 1H)	6.88	6.89 (s, 1H)	6.89	0.01
8	6.14 (s, 1H)	6.14	6.14 (s, 1H)	6.14	0.00
10	6.04 (d, *J* = 14.3, 1H)	6.04	6.04 (m, 1H)	6.04	0.00
11	6.57 (m, 1H)	6.57	6.59 (dd, J = 15.5, 7.0 Hz, 1H)	6.59	0.02
12	1.94 (dd, *J* = 7.0, 1.7 Hz, 3H)	1.94	1.95 (dd, *J* = 7.0, 1.2 Hz, 3H)	1.95	0.01
13	1.70 (s, 3H)	1.70	1.71 (s, 3H)	1.71	0.01
17	2.92 (m, 2H)	2.92	2.94 (m, 2H)	2.94	0.02
18	1.59 (m, 2H)	1.59	1.61 (m, 2H)	1.61	0.02
19,20,21,22	1.26 (m, 8H)	1.26	1.28 (m, 8H)	1.28	0.02
23	0.86 (t, J = 7.1 Hz, 3H)	0.86	0.87 (t, *J* = 6.8 Hz, 3H)	0.87	0.01

Monascorubrin ^{13}C NMR comparison table:

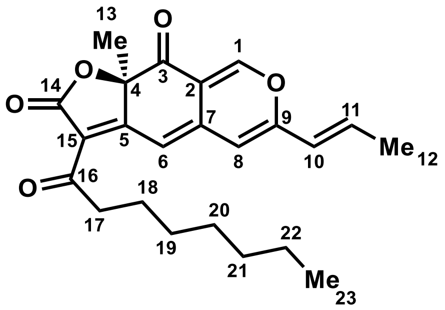

Monascorubrin

^{13}C NMR			
**reported data**[8]		**My data**	**Difference**
no.	**peak detail**	**peak detail**	
1	153.0	152.9	0.1
2	109.8	109.7	0.1
3	191.0	191.0	0.0
4	86.0	85.9	0.1
5	169.4	169.4	0.0
6	113.4	113.4	0.0
7	141.8	141.7	0.1
8	104.4	104.3	0.0
9	156.6	156.5	0.1
10	116.5	116.4	0.1
11	136.6	136.5	0.1
12	19.0	18.9	0.1
13	28.5	28.5	0.0
14	171.8	171.8	0.0
15	122.5	122.5	0.0
16	197.6	197.6	0.0
17	41.9	41.8	0.1
18	23.9	23.9	0.0
19	29.4	29.3	0.1
20	29.4	29.3	0.1
21	31.9	31.9	0.0
22	22.8	22.8	0.0
23	14.3	14.2	0.1

iii. ***Monaphilol B NMR comparison table:***

Monaphilol B ^{1}H NMR comparison table

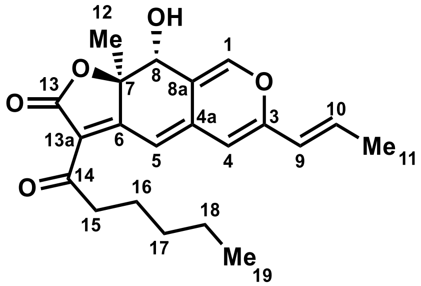

Monaphilol B

Monaphilol B-^{1}H NMR					
Reported data[9]			My data		Difference
no.	peak detail	Signal	peak detail	Signal	
1	7.52 (d, *J* = 2.0, 1H)	7.52	7.52 (s, 1H)	7.52	0
4	6.41 (s, 1H)	6.41	6.42 (s, 1H)	6.42	0.01
5	6.67 (s, 1H)	6.67	6.67 (s, 1H)	6.67	0
8	4.72 (dd, *J* = 5.2, 2.0, 1H)	4.72	4.72 (dd, J = 4.9, 2.2 Hz, 1H)	4.72	0
8-OH	5.52 (d, *J* = 5.2, 1H)	5.52	5.50 (d, J = 5.0 Hz, 1H)	5.50	0.02
9	6.23 (d, *J* = 16.0, 1H)	6.23	6.23 (d, J = 15.6 Hz, 1H)	6.23	0
10	6.57 (dq, *J* = 16.0, 6.8, 1H)	6.57	6.59 (dq, 15.6, 7.0 Hz, 1H)	6.59	0.02
11	1.90 (d, *J* = 6.8, 3H)	1.90	1.91 (d, *J* = 6.9 Hz, 3H)	1.91	0.01
12	1.32 (s)	1.32	1.32 (s, 3H)	1.32	0
15	2.83 (t, *J* = 7.2, 2H)	2.83	2.83 (m, 2H, overlap with water peak)	2.83	0
16	1.56 (m, 2H)	1.56	1.57 (m, 2H)	1.57	0.01
17 and 18	1.29 (m, 4H)	1.29	1.29 (m, 4H)	1.29	0
19	0.87 (t, J = 6.8, 3H)	0.87	0.88 (t, *J* = 6.7 Hz, 3H)	0.88	0.01

Monaphilol B ^{13}C NMR comparison table:

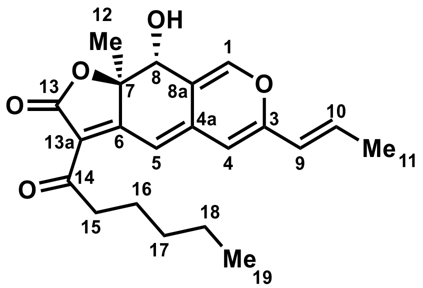

Monaphilol B

Monaphilol B-^{13}C NMR			
**Reported data[9]**		**My data**	**Difference**
no.	**peak detail**	**peak detail**	
1	147.0	147.1	0.1
3	158.1	158.2	0.1
4	109.1	109.1	0
4a	145.9	145.9	0
5	103.5	103.5	0
6	173.9	174.0	0.1
7	83.6	83.7	0.1
8	71.9	72.0	0.1
8a	122.6	122.7	0.1
9	124.2	124.2	0
10	135.3	135.4	0.1
11	18.5	18.5	0
12	19.7	19.7	0
13	111.6	111.7	0.1
13a	171.2	171.3	0.1
14	197.0	197.0	0
15	41.6	41.7	0.1
16	24.3	24.4	0.1
17	32.3	32.3	0
18	23.2	23.2	0
19	14.2	14.3	0.1

iv. Rubropunctamine NMR comparison table:

rubropunctamine ^{1}H NMR comparison table:

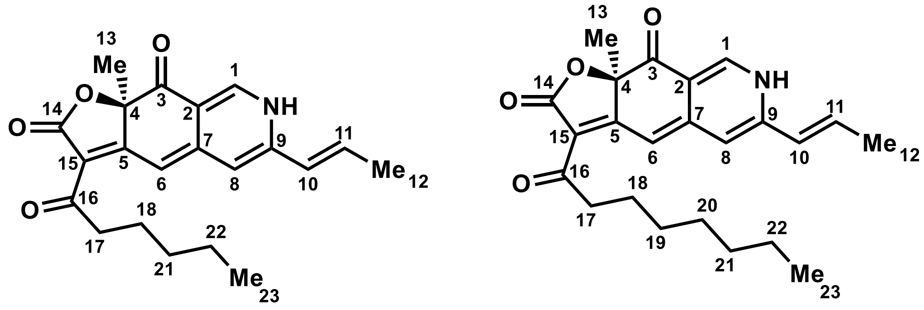

rubropunctamine-^{1}H NMR					
**Reported rubropunctamine data (C5 side chain)[7]**			**My data (C5 side chain)**		**Difference**
no.	**peak detail**	**Signal**	**peak detail**	**Signal**	
1	9.25 (s, 1H)	9.25	9.27 (s, 1H)	9.27	0.02
6	6.77 (s, 1H)	6.77	6.78 (s, 1H)	6.78	0.01
8	6.75 (s, 1H)	6.75	6.73 (s, 1H)	6.73	0.02
10	6.39 (d, 1H)	6.39	6.35 (d, *J* = 15.9 Hz 1H)	6.35	0.04
11	7.05 (dq, 1H)	7.05	7.02 (m, 1H)	7.02	0.03
12	2.05 (d, 3H)	2.05	2.05 (d, *J* = 6.5 Hz, 3H)	2.05	0.00
13	1.81 (s, 3H)	1.81	1.81 (s, 3H)	1.81	0.00
17	2.86 (m, 2H)	2.86	2.87 (m, 2H)	2.87	0.01
18	1.70-1.64 (m, 2H)	1.67	1.67 (m, 2H)	1.67	0.00
21,22	1.37 (m, 4H)	1.37	1.36 (m, 4H)	1.36	0.01
23	0.90 (t, 3H)	0.9	0.90 (t, *J* = 6.8 Hz, 3H)	0.9	0.00
NH	not founded	-	12.62 (s, 1H)	12.62	-

rubropunctamine-^{1}H NMR					
**Reported monascorubramine data (C7 side chain)[8]**			**My data (C5 side chain)**		**Difference**
no.	**peak detail**	**Signal**	**peak detail**	**Signal**	
1	9.27 (s, 1H)	9.27	9.27 (s, 1H)	9.27	0.00
6	6.77 (s, 1H)	6.77	6.78 (s, 1H)	6.78	0.01
8	6.76 (s, 1H)	6.76	6.73 (s, 1H)	6.73	0.03
10	6.36 (d, *J* = 15.9, 1H)	6.36	6.35 (d, *J* = 15.9 Hz 1H)	6.35	0.01
11	7.04 (m, 1H)	7.04	7.02 (m, 1H)	7.02	0.02
12	2.04 (d, *J* = 6.0 Hz, 3H)	2.04	2.05 (d, *J* = 6.5 Hz, 3H)	2.05	0.01
13	1.80 (s, 3H)	1.80	1.81 (s, 3H)	1.81	0.01
17	2.87 (m, 2H)	2.87	2.87 (m, 2H)	2.87	0.00
18	1.65 (m, 2H)	1.65	1.67 (m, 2H)	1.67	0.02
19,20,21,22 or 21, 22	1.36 (m, 8H)	1.36	1.36 (m, 4H)	1.36	0.00
23	0.85 (t, J = 7.1 Hz, 3H)	0.85	0.90 (t, *J* = 6.8 Hz, 3H)	0.9	0.05
NH	not founded	-	12.62 (s, 1H)	12.62	-

rubropunctamine ^{13}C NMR comparison table:

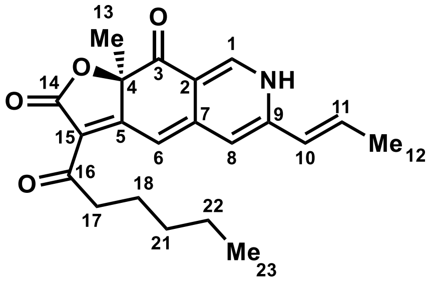

rubropunctamine

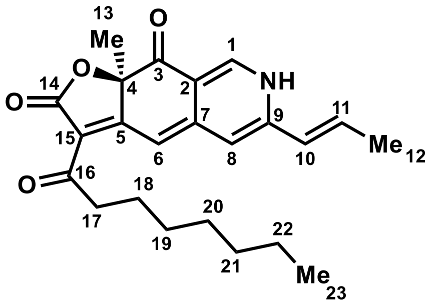

Monascorubramine

^{13}C NMR			
monascorubrin-reported data[8]		My rubropunctatin data	Difference
no.	peak detail	peak detail	
1	147.5	147.3	0.2
2	101.0	101.1	0.1
3	195.3	195.4	0.1
4	86.8	86.8	0.0
5	153.6	153.7	0.1
6	116.2	116.1	0.1
7	140.8	140.9	0.1
8	98.5	98.7	0.2
9	173.0 (buried)	173.0	0.0
10	117.5	117.5	0.0
11	138.4	138.1	0.3
12	19.3	19.2	0.1
13	22.6	22.5	0.1
14	174.2	174.4	0.2
15	123.3	123.3	0.0
16	196.3	196.6	0.3
17	40.5	40.6	0.1
18	29.6	29.6	0.1
19	29.7	-	
20	29.6	-	
21	31.8	31.9	0.1
22	24.9	24.5	0.4
23	14.1	14.1	0.1

v. Rubropunctain alanine NMR compariosn table:

rubropunctain alanine ^{1}H NMR comparison table:

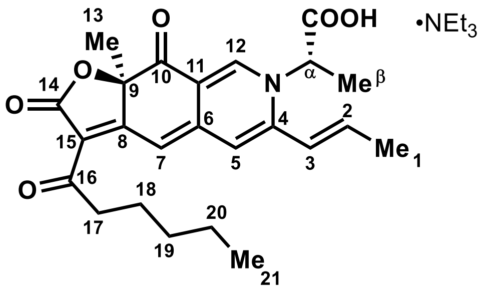

rubropunctain alanine

rubropunctain alanine-^{1}H NMR					
Reported data (with L-Ala)[10]			My data (with L-Ala)		Difference
no.	peak detail	Signal	peak detail	Signal	
1	1.98 (d, *J* = 6.6 Hz, 3H)	1.98	1.99 (dd, *J* = 6.6, 1.6 Hz, 3H)	1.99	0.01
2	6.59 (dq, J = 15.3, 6.6 Hz, 1H)	6.59	6.61 (dq, *J* = 15.4, 6.6 Hz, 1H)	6.61	0.02
3	6.51 (brd, J = 15.3 Hz, 1H)	6.51	6.51 (dd, *J* = 15.4, 1.8 Hz, 1H)	6.51	0
5	7.08 (s, 1H)	7.08	7.09 (s, 1H)	7.09	0.01
7	6.72 (s, 1H)	6.72	6.73 (s, 1H)	6.73	0.01
12	8.28 (s, 1H)	8.28	8.29 (s, 1H)	8.29	0.01
13	1.67 (s, 3H)	1.67	1.65 (s, 3H)	1.65	0.02
17	2.81 (m, 2H)	2.81	2.83 – 2.80 (m, 2H)	2.82	0.01
18	1.60 (p, J = 7.2 Hz, 2H)	1.60	1.60 (p, *J* = 7.4 Hz, 2H)	1.60	0
19,20	1.34-1.30 (m, 4H)	1.32	1.36 – 1.33 (m, 4H)	1.34	0.02
21	0.89 (t, J = 6.9 Hz, 3H)	0.89	0.91 (t, *J* = 6.9 Hz, 3H)	0.91	0.02
alpha	4.95 (q, J = 7.2 Hz, 1H)	4.95	4.95 (q, *J* = 7.2 Hz, 1H)	4.95	0
beta	1.73 (d, J = 7.2 Hz, 3H)	1.73	1.74 (d, J = 7.2 Hz, 3H)	1.75	0.02

Plus two peaks for TEA
3.21 (q, J = 7.3 Hz, 6H)
1.31 (t, J = 7.2 Hz, 9H)

rubropunctain alanine-^{1}H NMR					
Reported data (with D-Ala)[10]		My data (with L-Ala)		My data (with D-Ala)	
no.	**Signal**	**Signal**	**Difference**	**Signal**	**Difference**
1	1.98	1.99	0.01	1.99	0.01
2	6.59	6.61	0.02	6.59	0
3	6.52	6.51	0.01	6.53	0.01
5	7.06	7.09	0.03	7.08	0.02
7	6.71	6.73	0.02	6.71	0
12	8.23	8.29	0.06	8.25	0.02
13	1.67	1.65	0.02	1.68	0.01
17	2.81	2.82	0.01	2.81	0
18	1.60	1.60	0.00	1.6	0
19,20	1.32	1.34	0.02	1.31	0.01
21	0.89	0.91	0.02	0.91	0.02
alpha	4.95	4.95	0.00	overlap with water	
beta	1.74	1.75	0.01	1.76	0.02

rubropunctain alanine ^{13}C NMR comparison table:

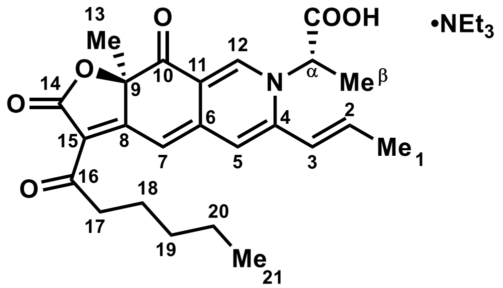

rubropunctain alanine

rubropunctain L-alanine-^{13}C NMR

Reported data[10]		My data	Difference
**Carbon**	**Signal**	**Signal**	
1	19.16	19.15	0.0
2	141.26	141.29	0.0
3	123.71	123.64	0.1
4	152.98	152.93	0.0
5	119.42	119.41	0.0
6	153.51	153.52	0.0
7	98.84	98.85	0.0
8	173.68	173.68	0.0
9	86.39	86.89	0.5
10	196.35	196.17	0.2
11	119.61	119.66	0.0
12	141.59	141.53	0.1
13	30.73	30.67	0.1
14	173.89	173.9	0.0
15	102.05	101.98	0.1
16	198.73	198.74	0.0
17	41.27	41.16	0.1
18	26.45	26.16	0.3
19	32.97	32.92	0.0
20	23.71	23.61	0.1
21	14.44	14.34	0.1
alpha	65.48	65.49	0.0
carboxylic acid	174.66	not dectected due to the TEA salt fom	
beta	18.71	18.6	0.1

**Plus two peaks for TEA**
47.94
9.22

vi. *Natural product optical rotation and our assignment of configurations:*[10-12]

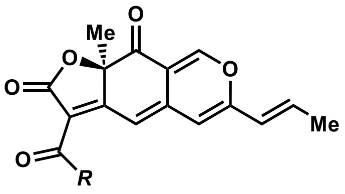

R = *n*-C_5H_{11}, (–)-rubropunctatin
reported $[\alpha]_D$ = -3481, c = 1.07, in ethanol
ours $[\alpha]_D$ = -998, c = 0.002, in enhanol
R = *n*-C_7H_{15}, (–)-monascorubrin
reported $[\alpha]_D$ = -1500, c = 0.10, in ethanol
ours $[\alpha]_D$ = -1780, c = 0.002, in ethanol

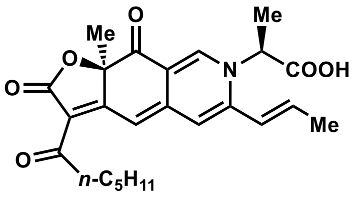

(–)-rubropunctatin L-alanine
reported $[\alpha]_D$ = -2500 with L-Ala, c = 0.0025, in methanol
$[\alpha]_D$ = -2800 with D-Ala, c = 0.0028, in methanol
ours $[\alpha]_D$ = -1425, c = 0.002, in methanol

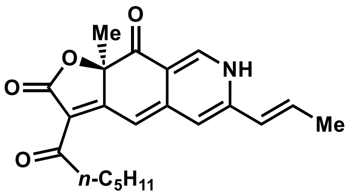

(–)-rubropunctamine
$[\alpha]_D$ = -1183, c = 0.0002, in cholorform

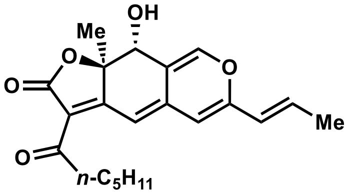

(–)-monophilol B
reported $[\alpha]_D$ = -2649, c = 0.33, in acetone
ours $[\alpha]_D$ = -1694, c = 0.15, in acetone

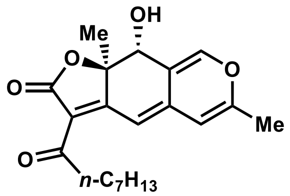

(–)-pitholide D's distereoisomer
$[\alpha]_D$ = -1532, c = 0.11, in methanol

reported:

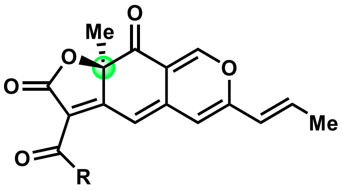

R = *n*-C_5H_{11}, (–)-rubropunctatin
R = *n*-C_7H_{15}, (–)-monascorubrin

our assignment:

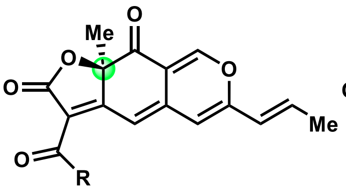

R = *n*-C_5H_{11}, (–)-rubropunctatin
R = *n*-C_7H_{15}, (–)-monascorubrin

reported:

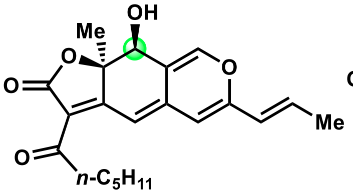

(–)-monophilol B

our assignment:

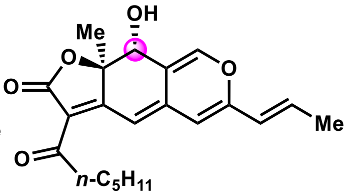

(–)-monophilol B

XIII. ^{1}H NMR and ^{13}C NMR spectra of compounds

S1: ^{1}H NMR (600 MHz, $CDCl_3$)

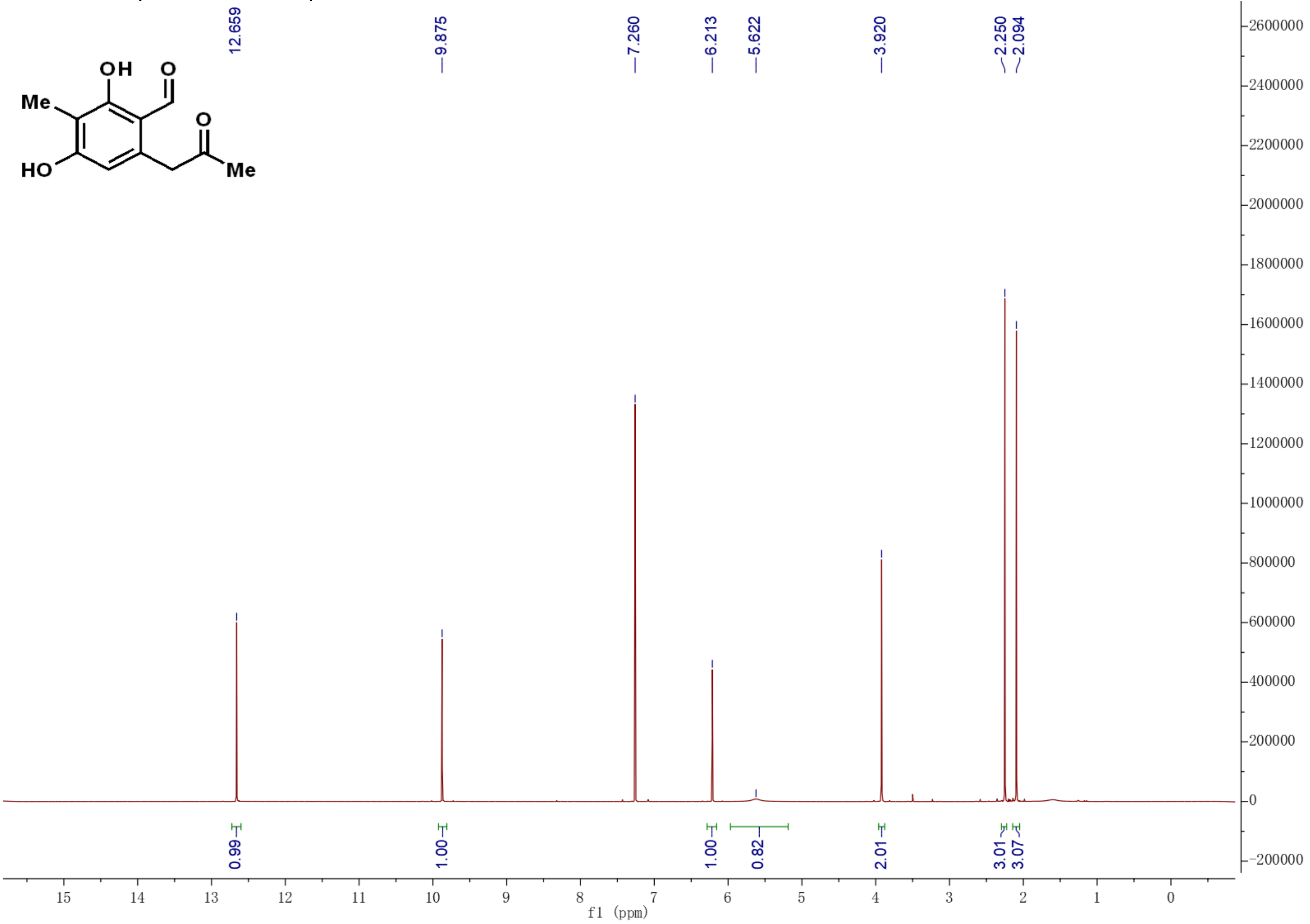

17: ^{13}C NMR (600 MHz, $CDCl_3$)

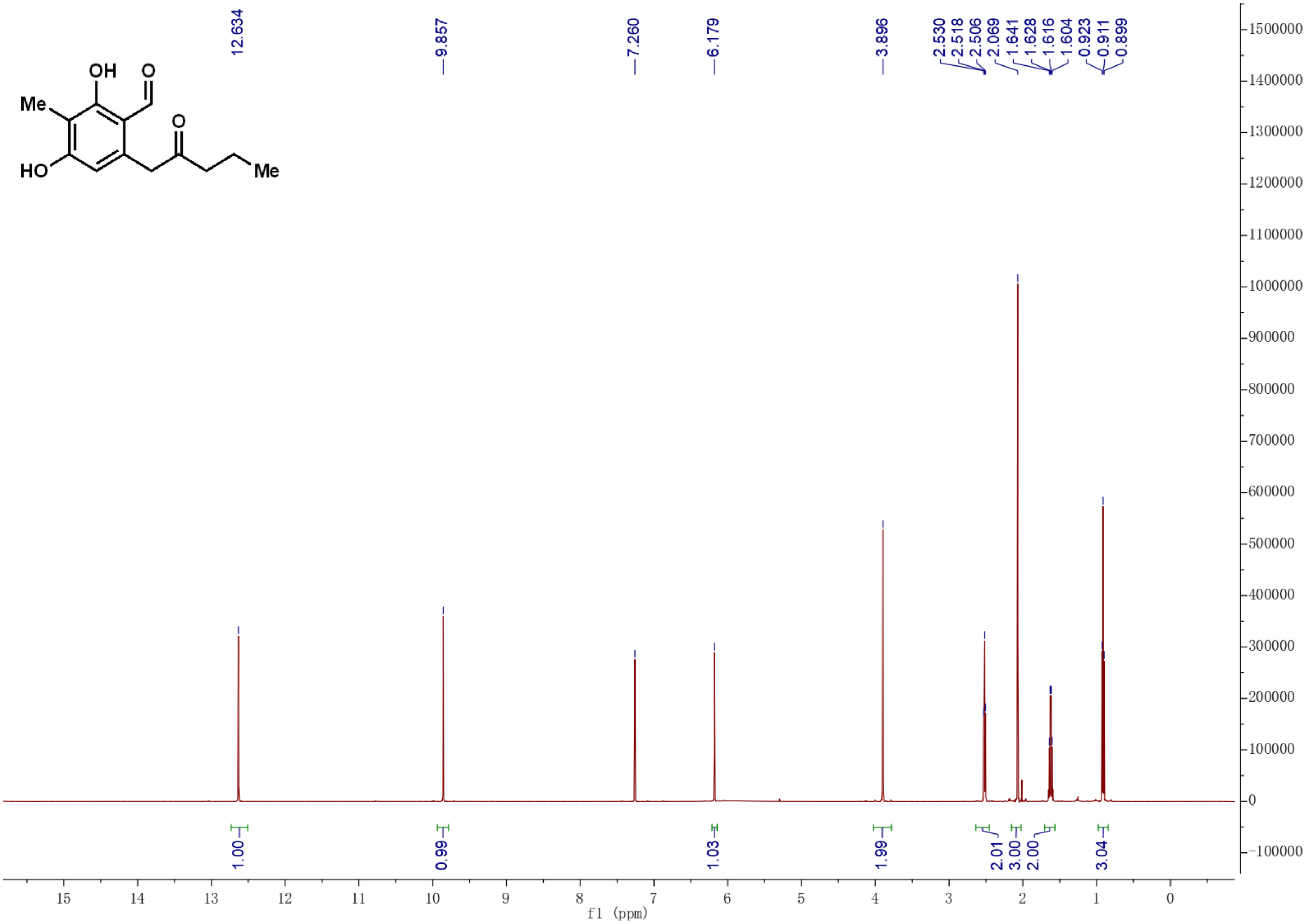

S2: ^{1}H NMR (600 MHz, CD_3OD)

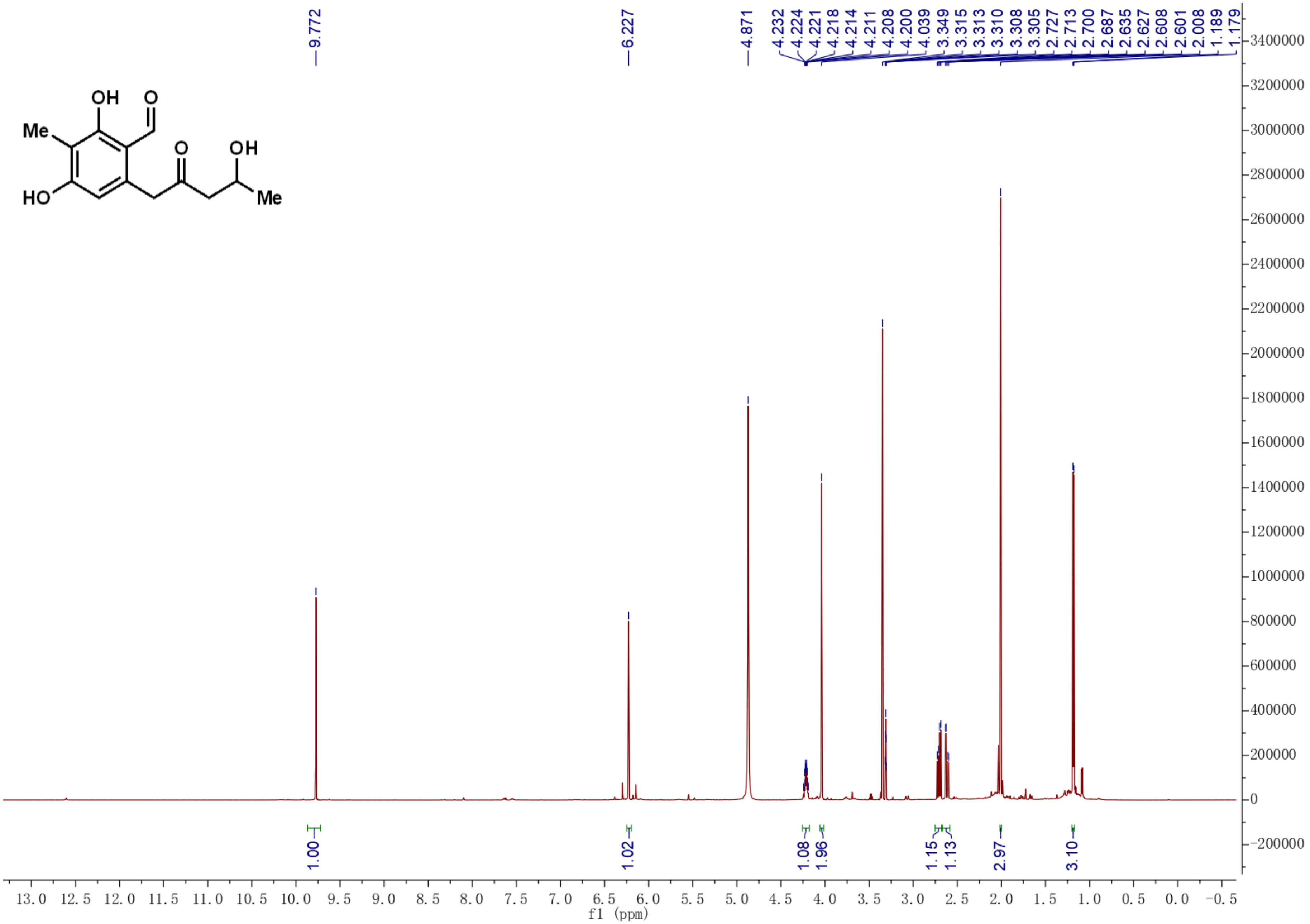

S3: ^{1}H NMR (600 MHz, CD_3OD)

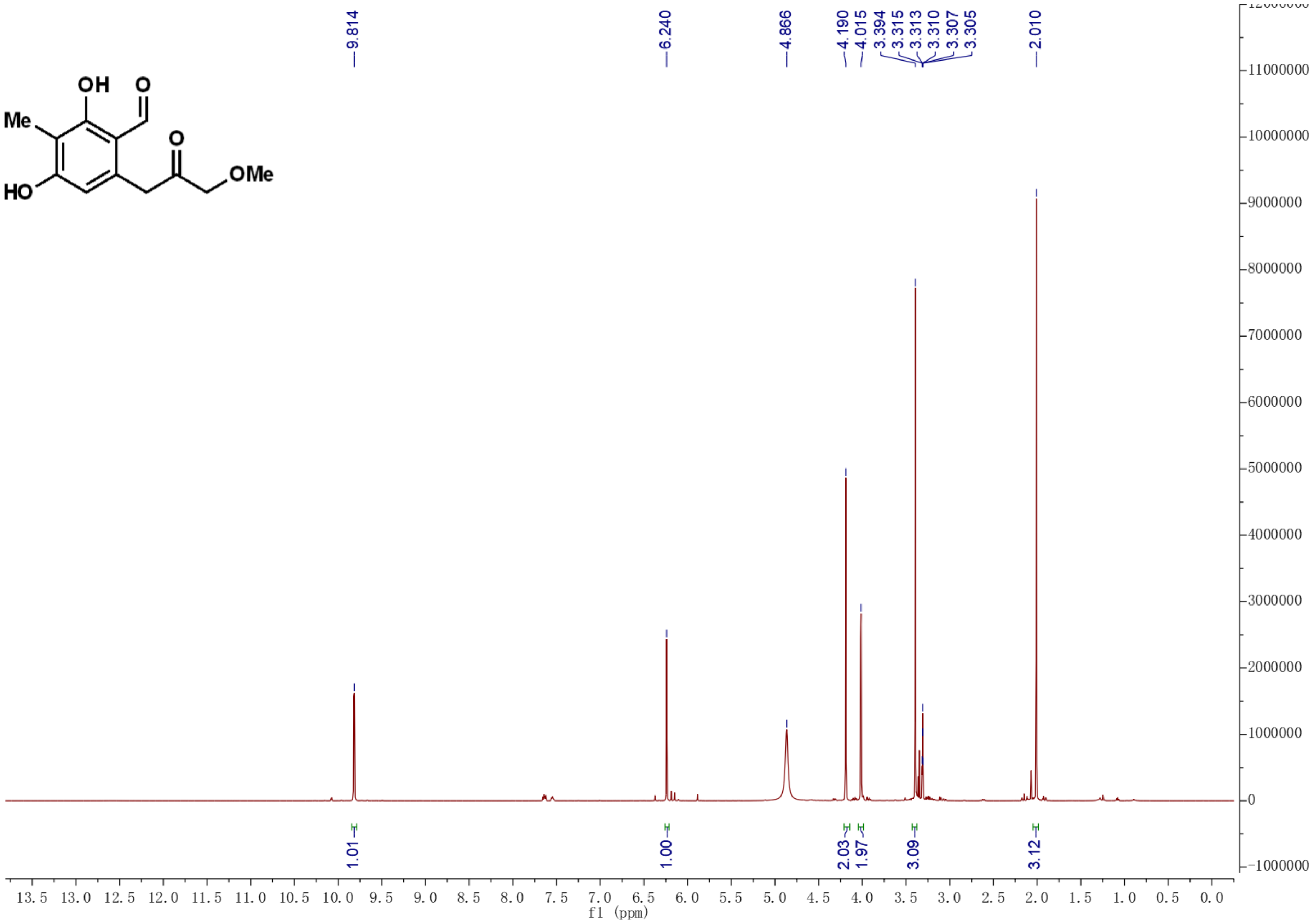

S4: ^{1}H NMR (400 MHz, CD_3OD)

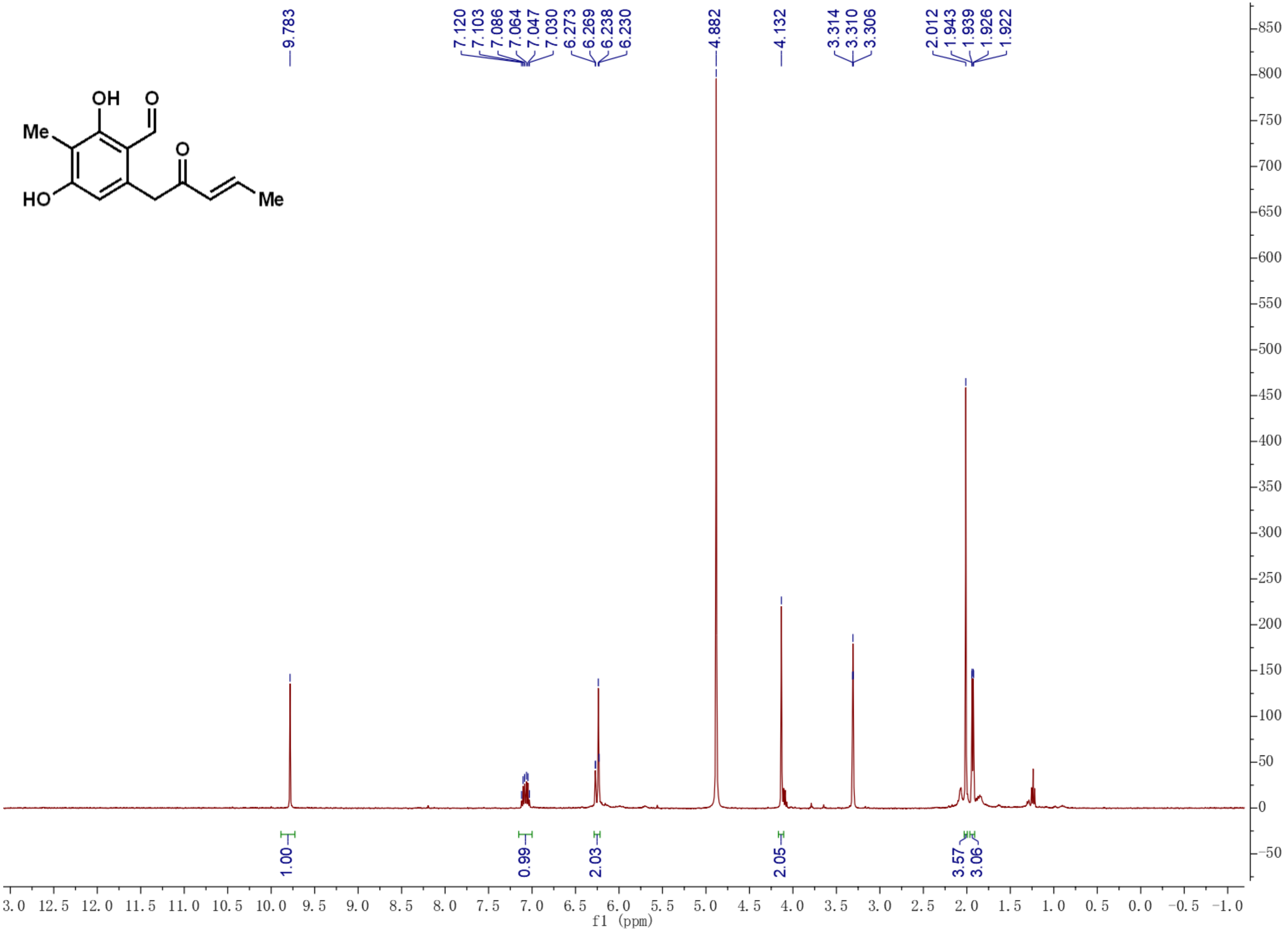

18: ^1H NMR (600 MHz, $CDCl_3$)

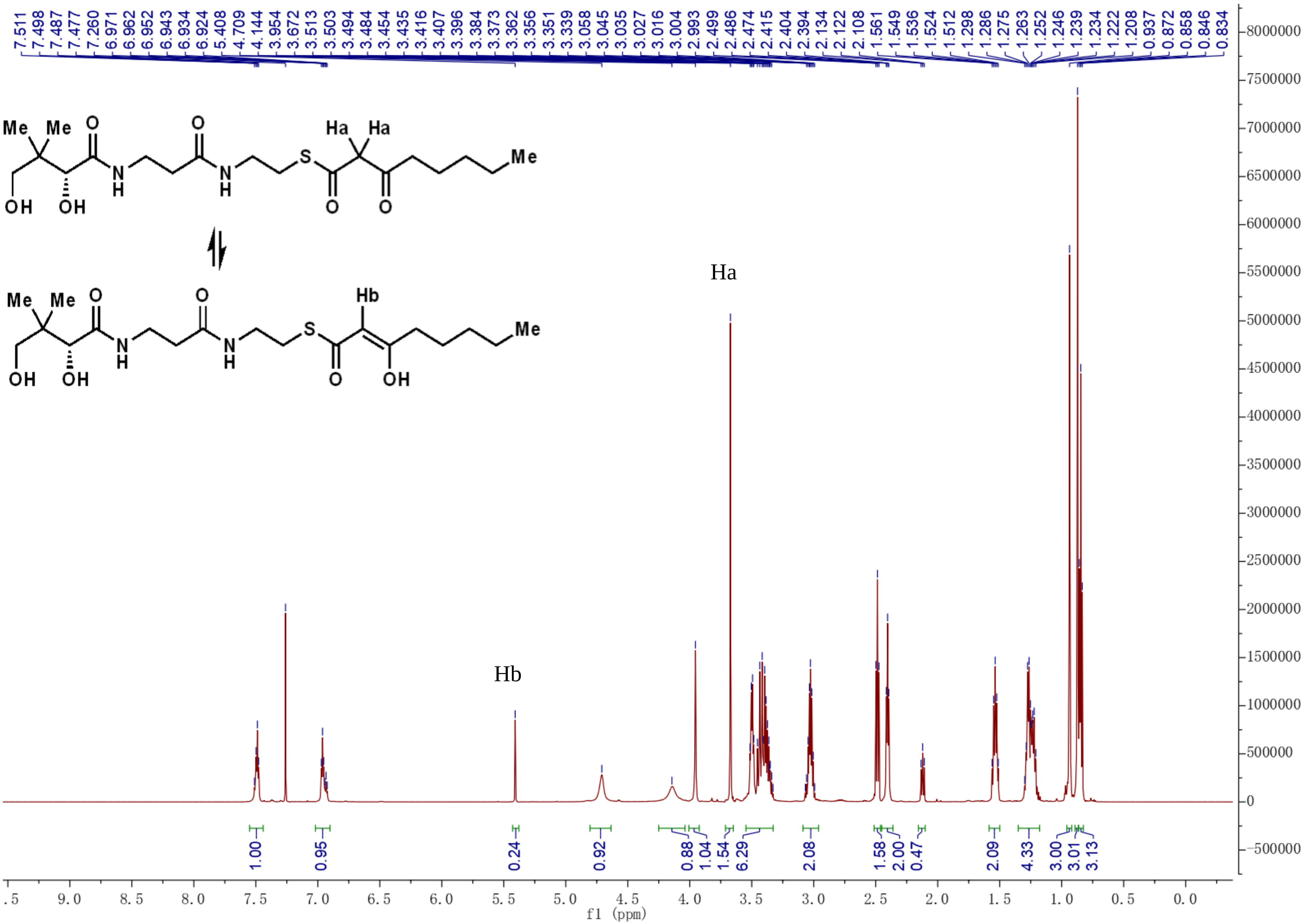

S8: ^{1}H NMR (600 MHz, CD_3OD)

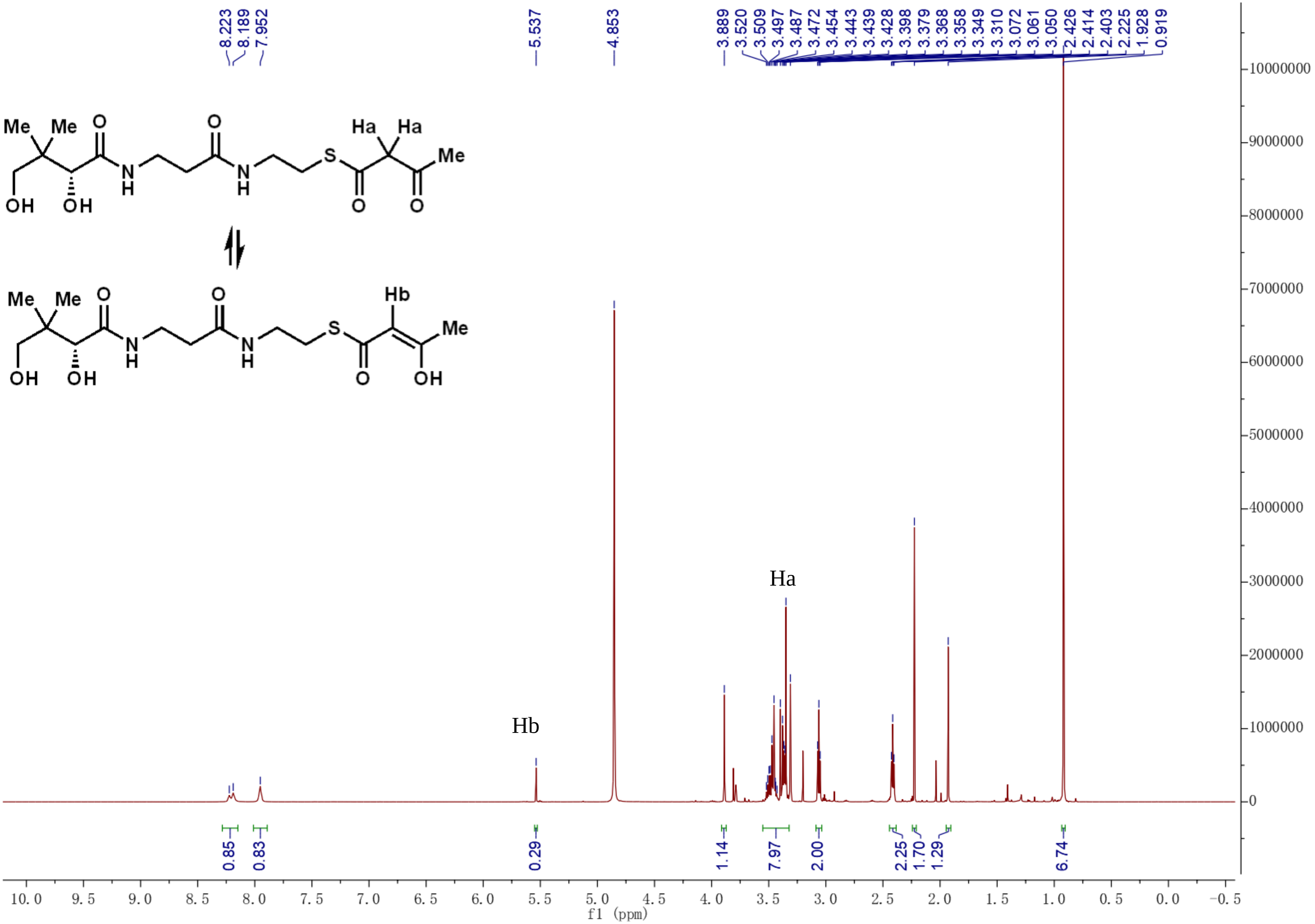

S9: ^{1}H NMR (600 MHz, $CDCl_3$)

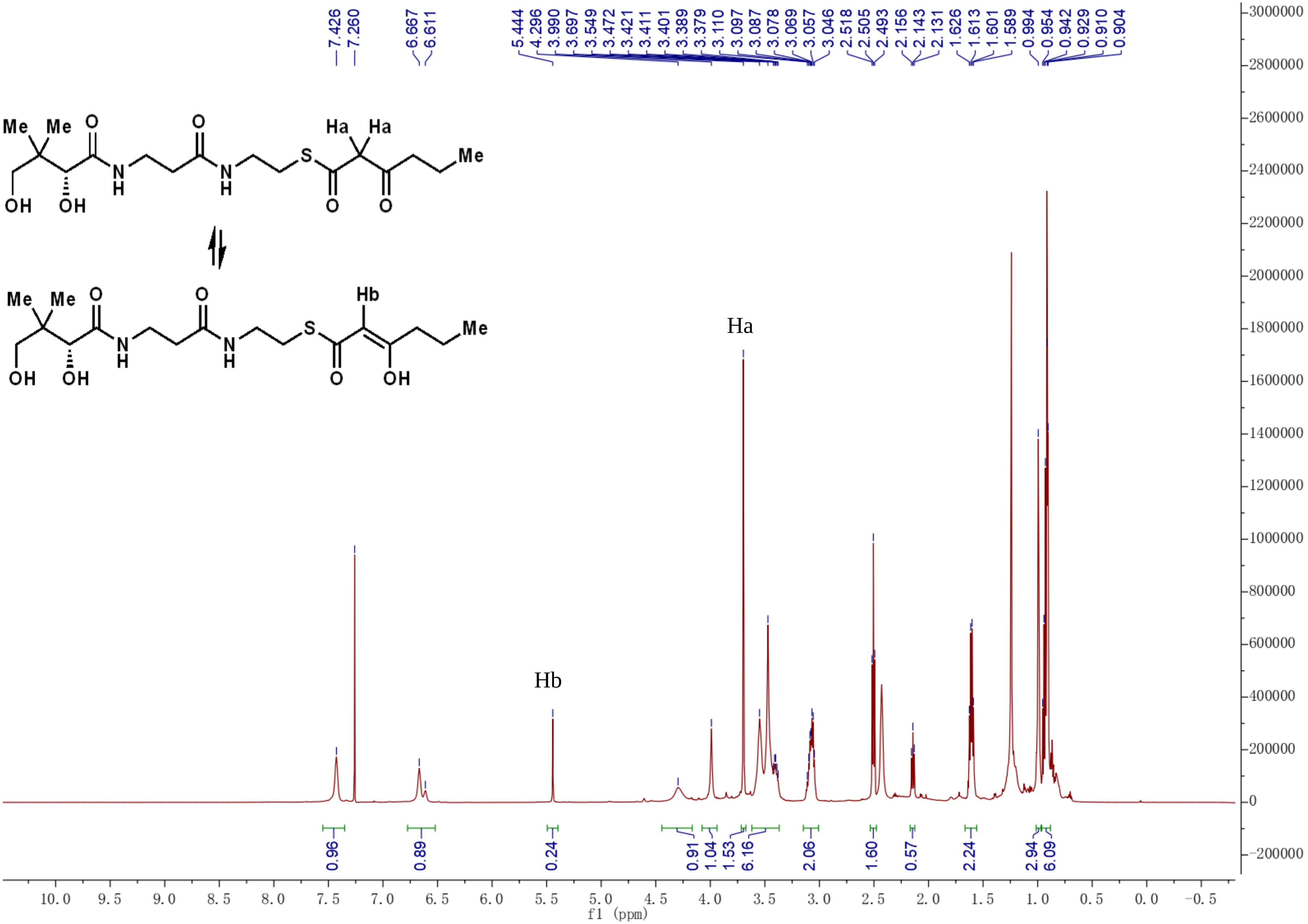

S9: ^{13}C NMR (150 MHz, $CDCl_3$)

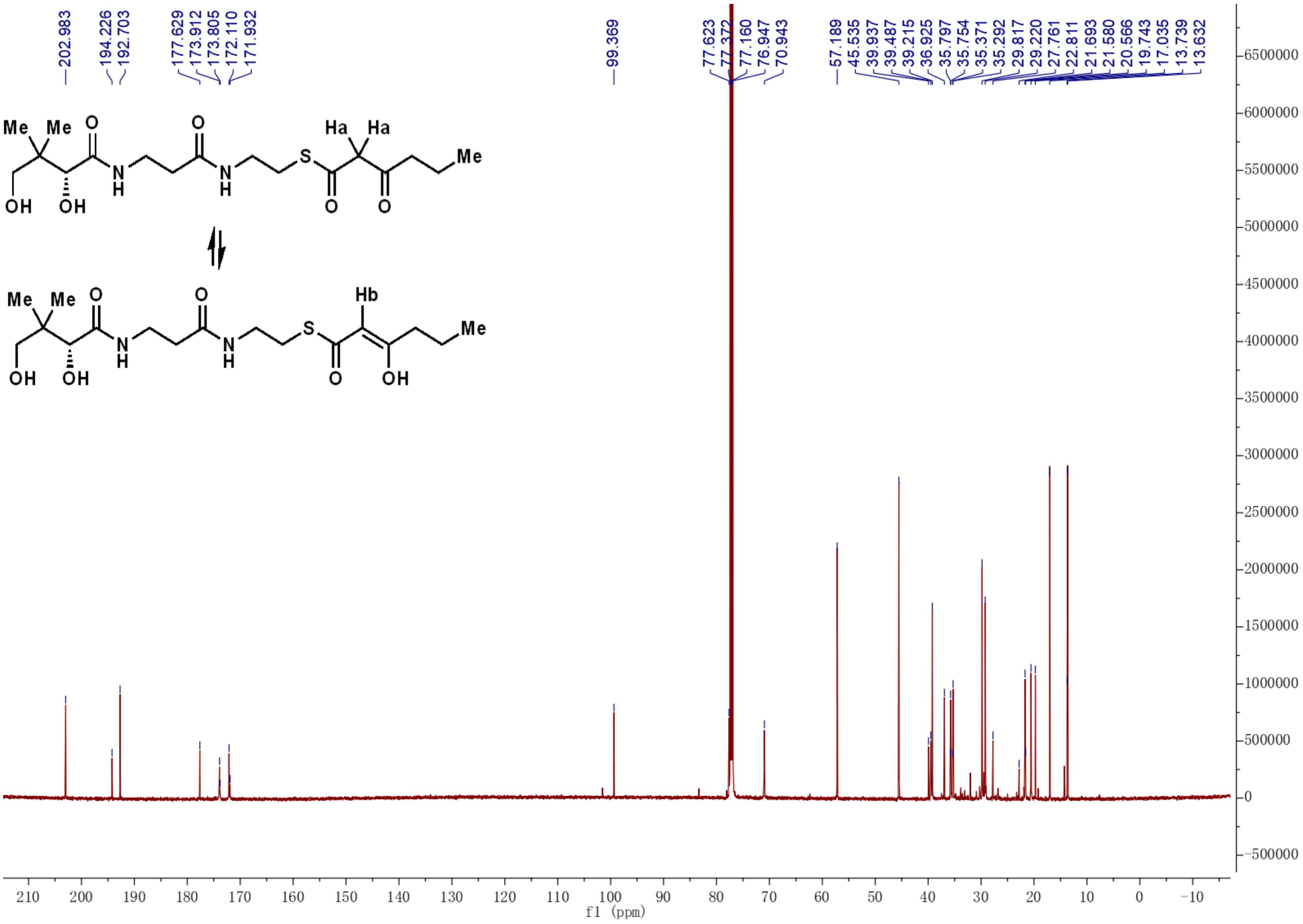

S10: ^1H NMR (600 MHz, $CDCl_3$)

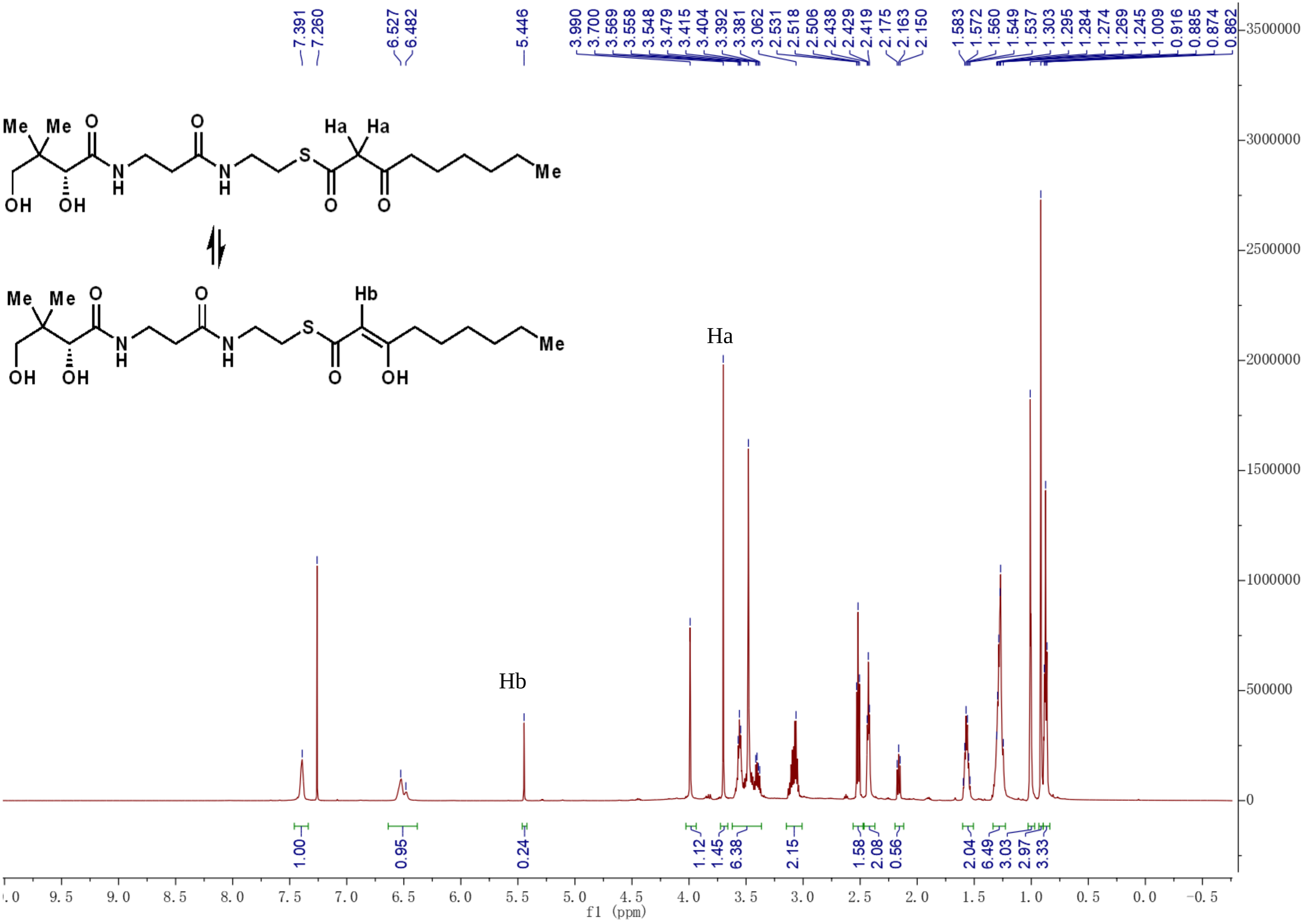

S10: ^{13}C NMR (150 MHz, $CDCl_3$)

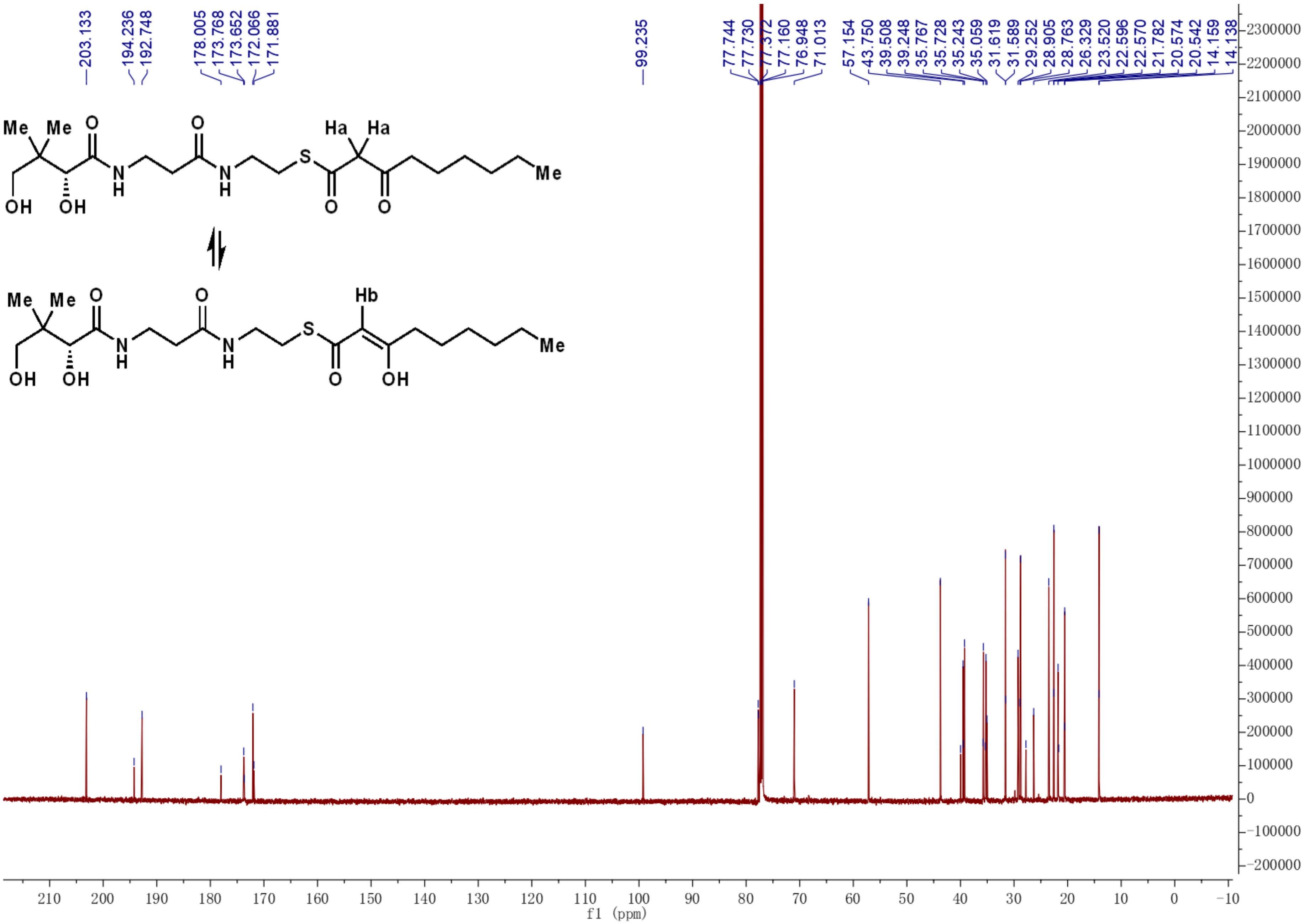

S11: ^{1}H NMR (600 MHz, $CDCl_3$)

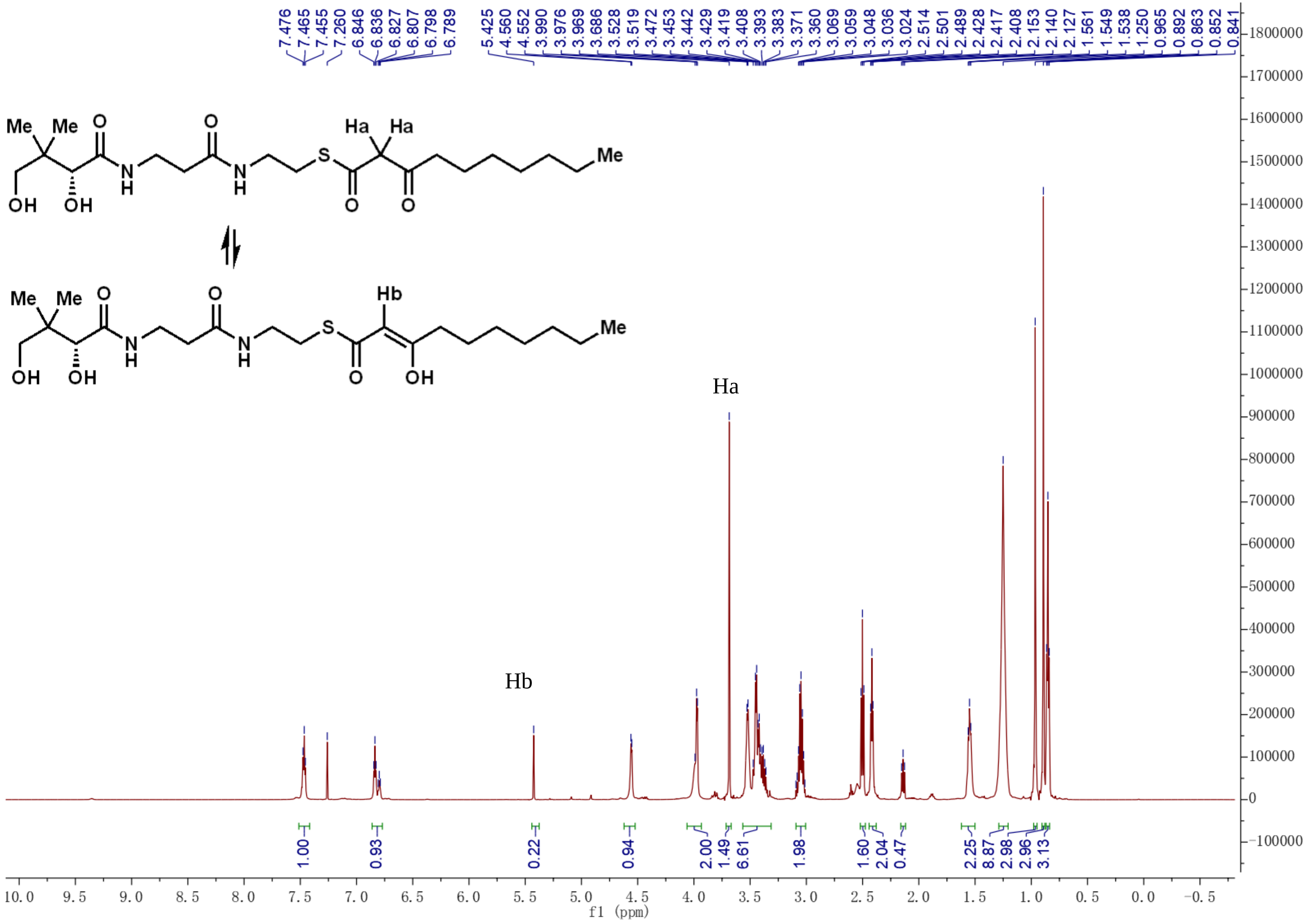

S11: ^{13}C NMR (150 MHz, $CDCl_3$)

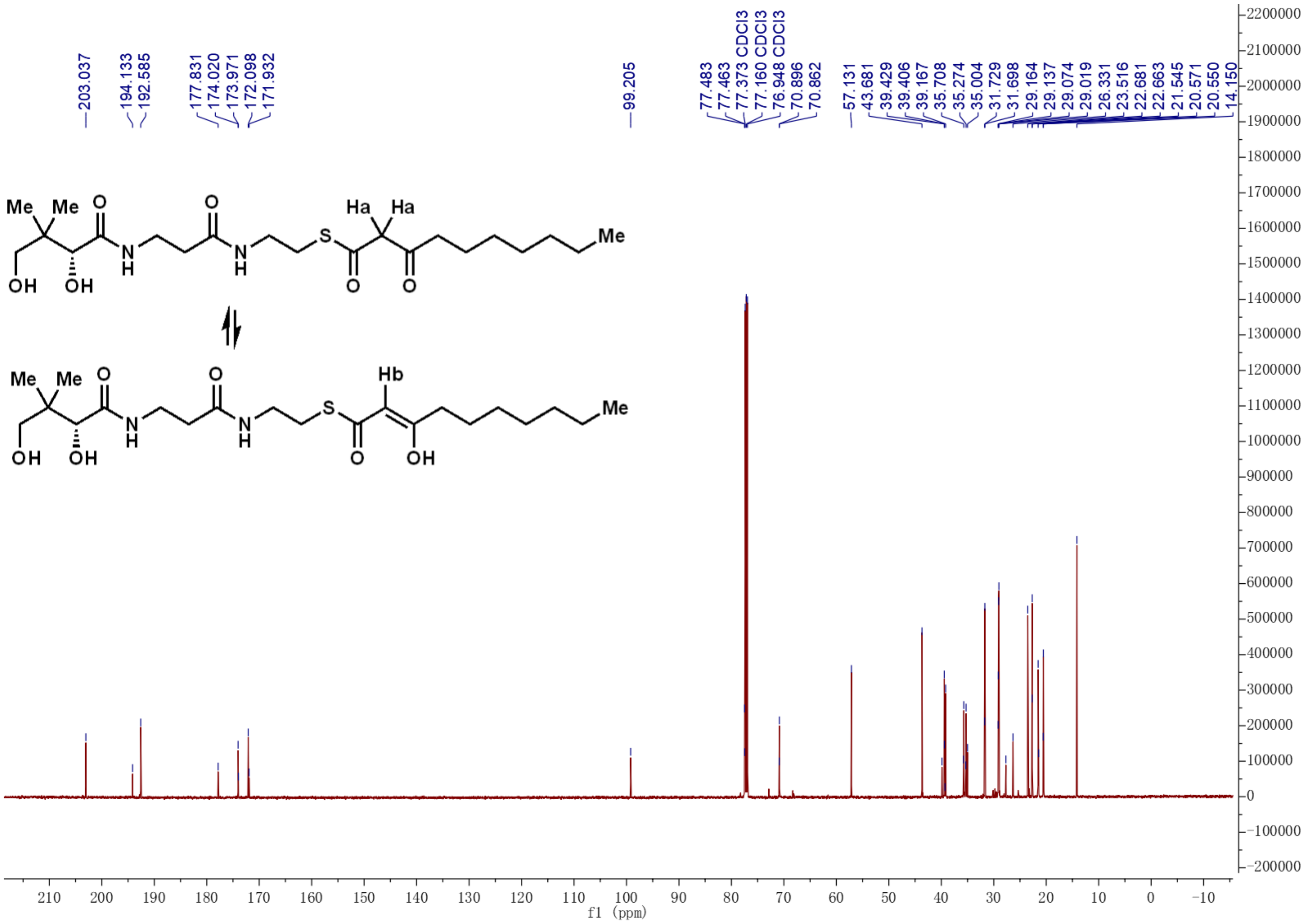

S12: ^{1}H NMR (600 MHz, $CDCl_3$)

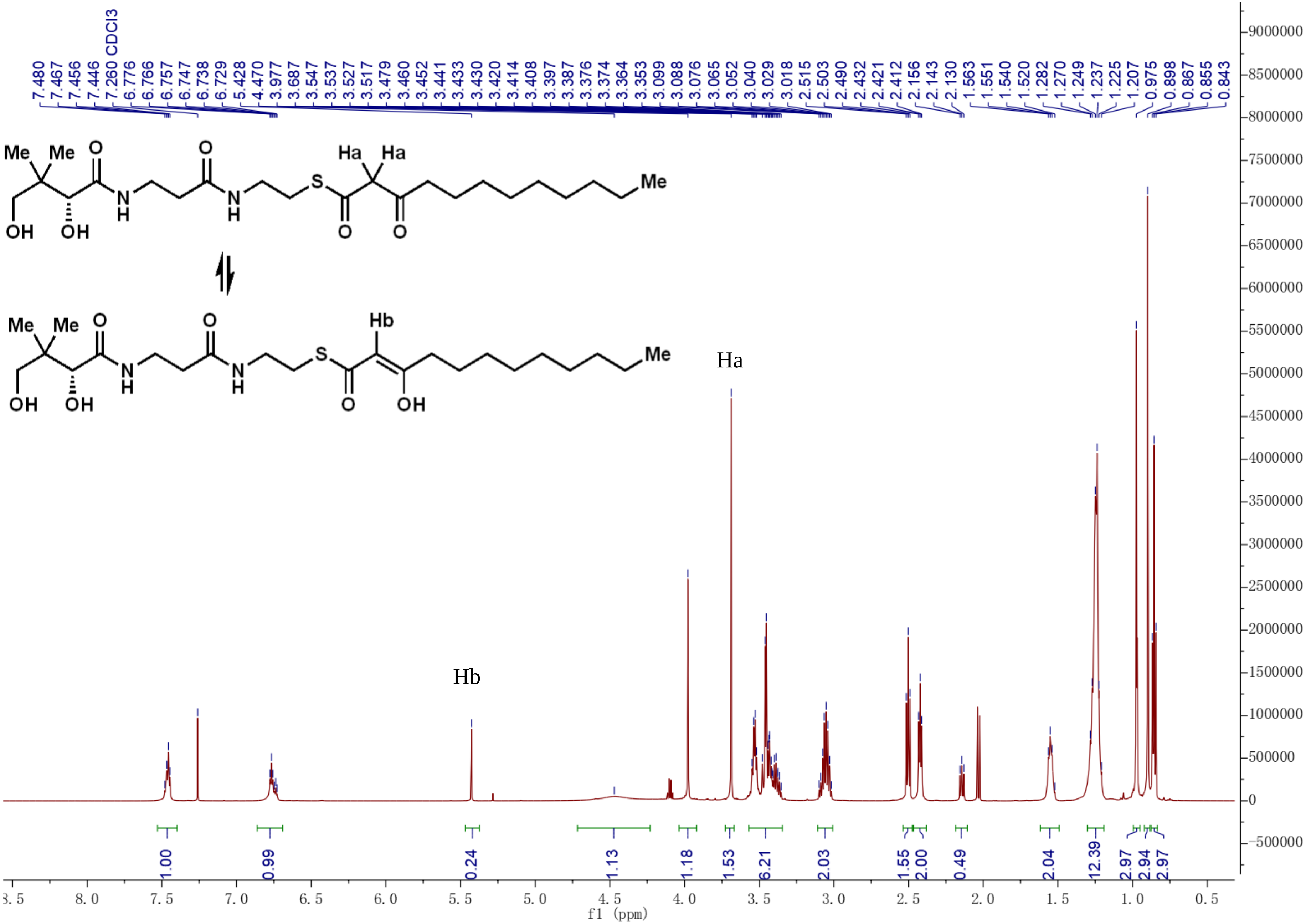

S12: ^{13}C NMR (150 MHz, $CDCl_3$)

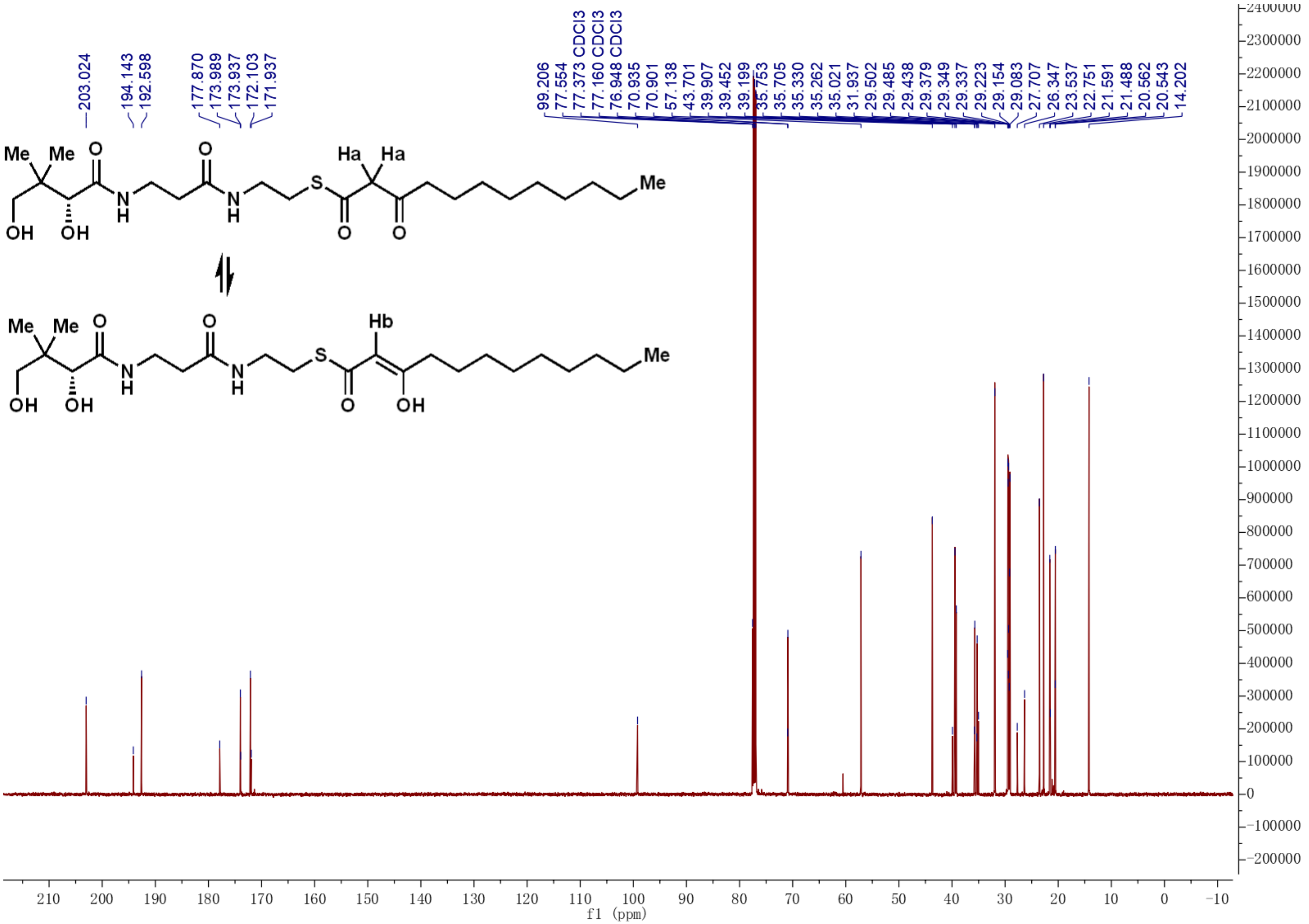

S13: ^{1}H NMR (600 MHz, $CDCl_3$)

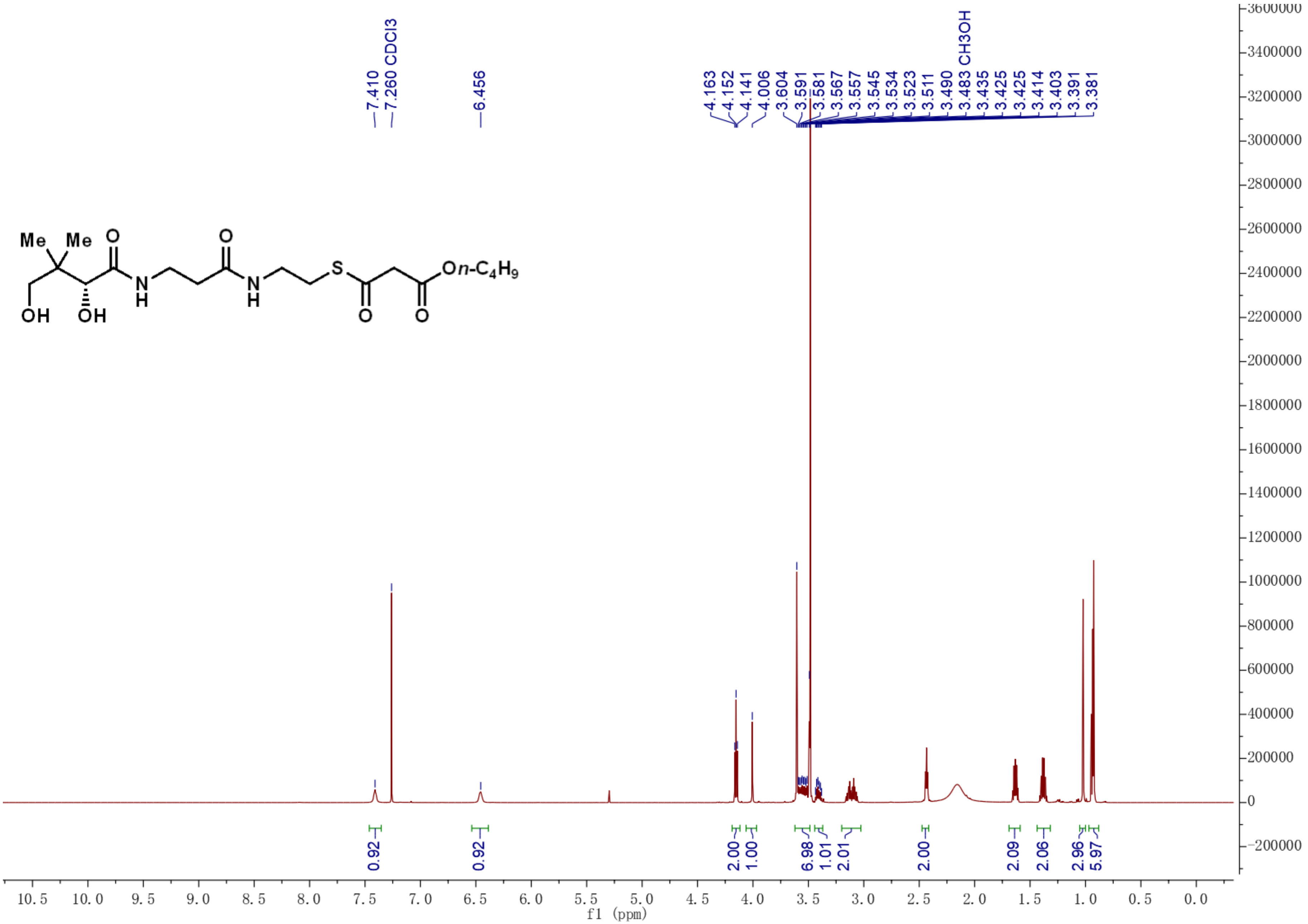

S13: ^{13}C NMR (150 MHz, $CDCl_3$)

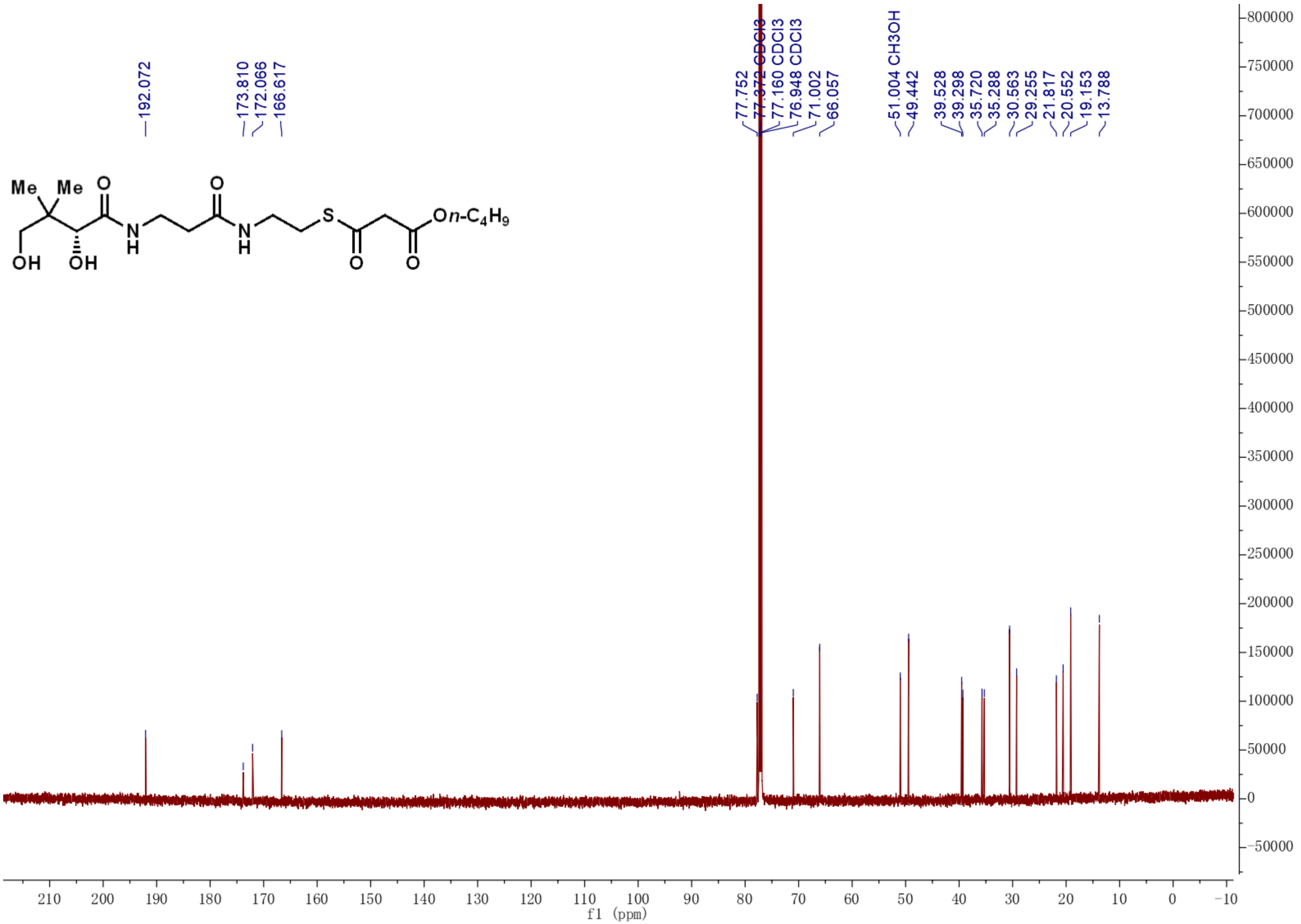

32: ^{1}H NMR (600 MHz, $CDCl_3$)

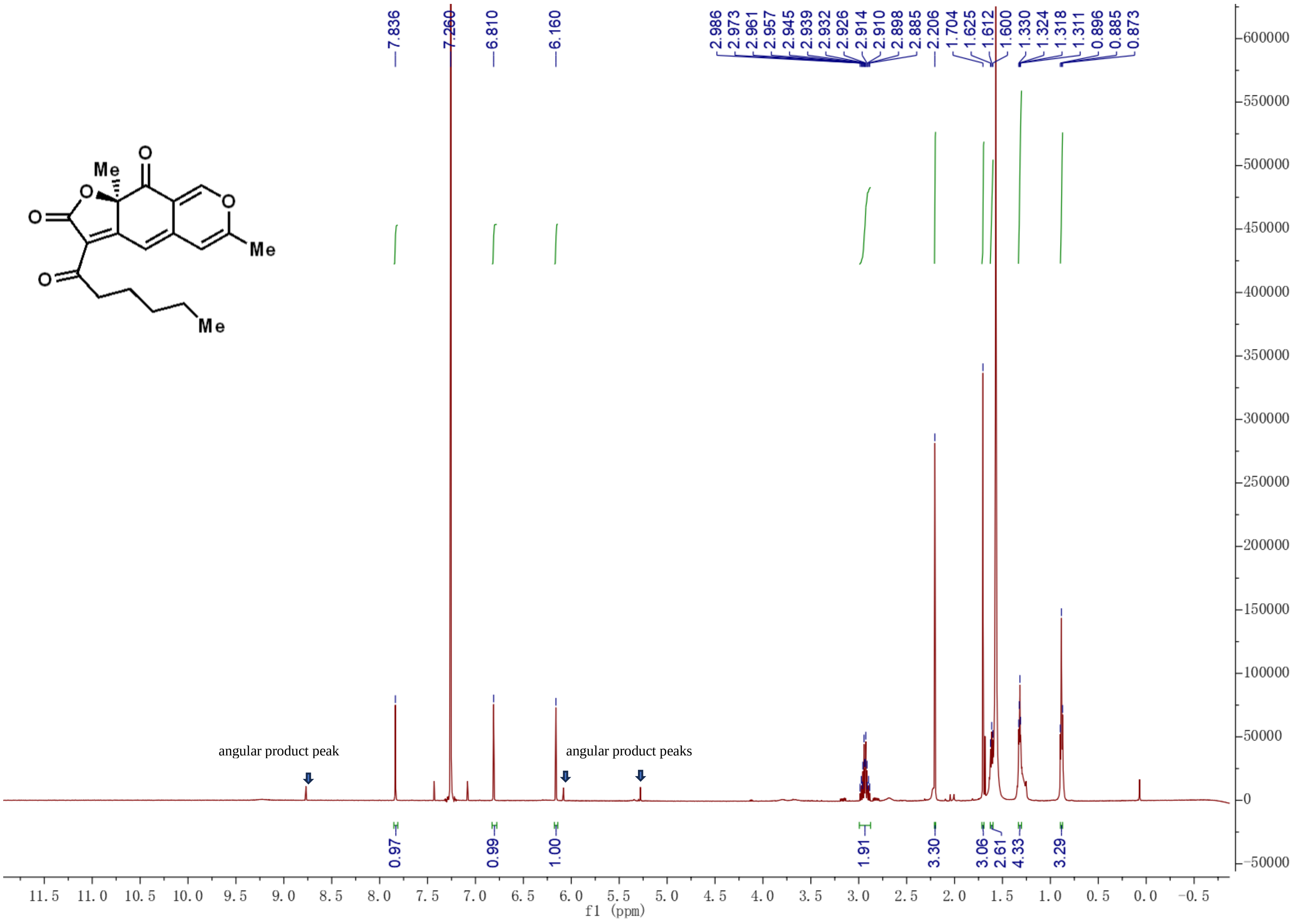

32: ^{13}C NMR (150 MHz, $CDCl_3$)

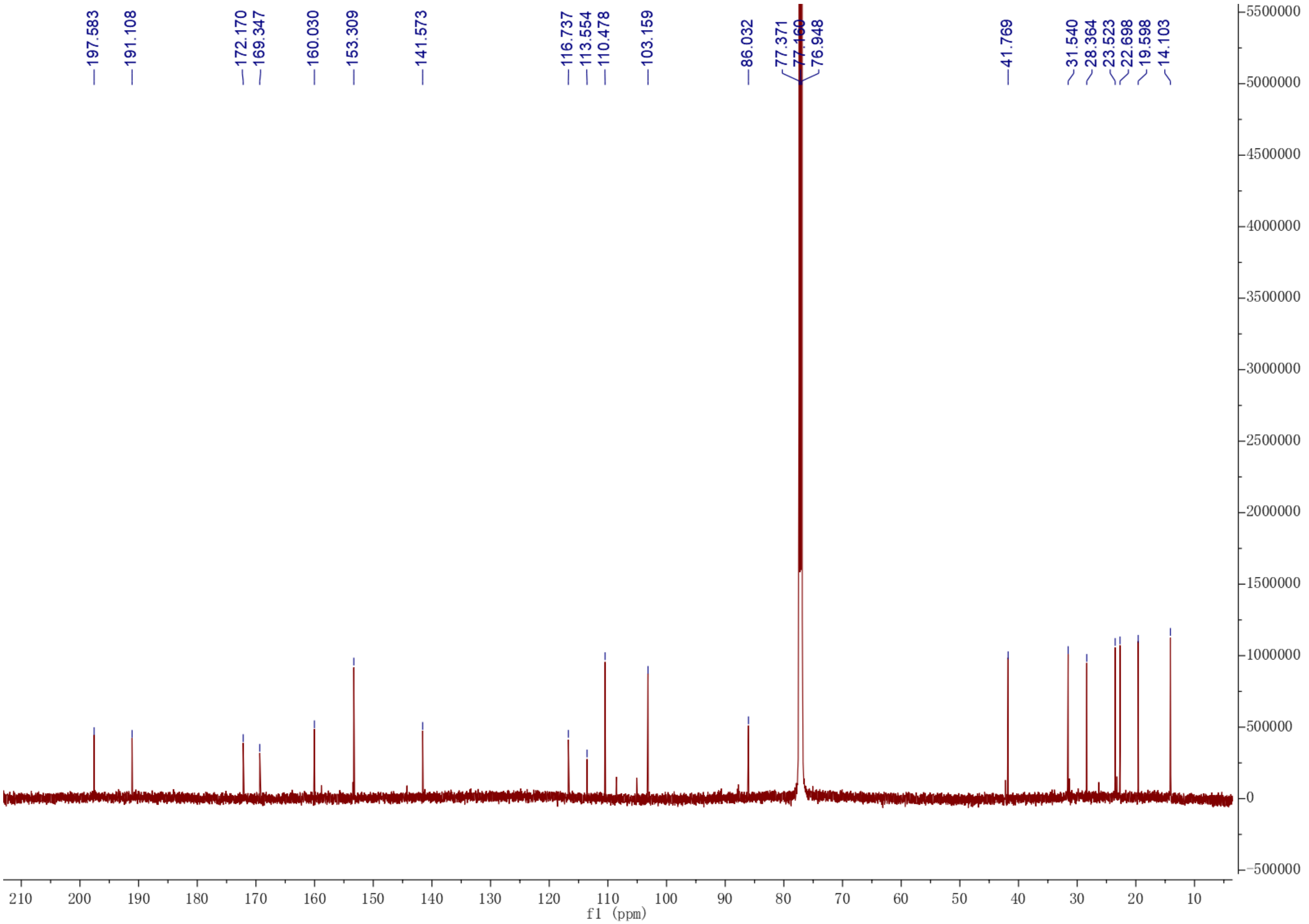

19: ^{1}H NMR (600 MHz, $CDCl_3$)

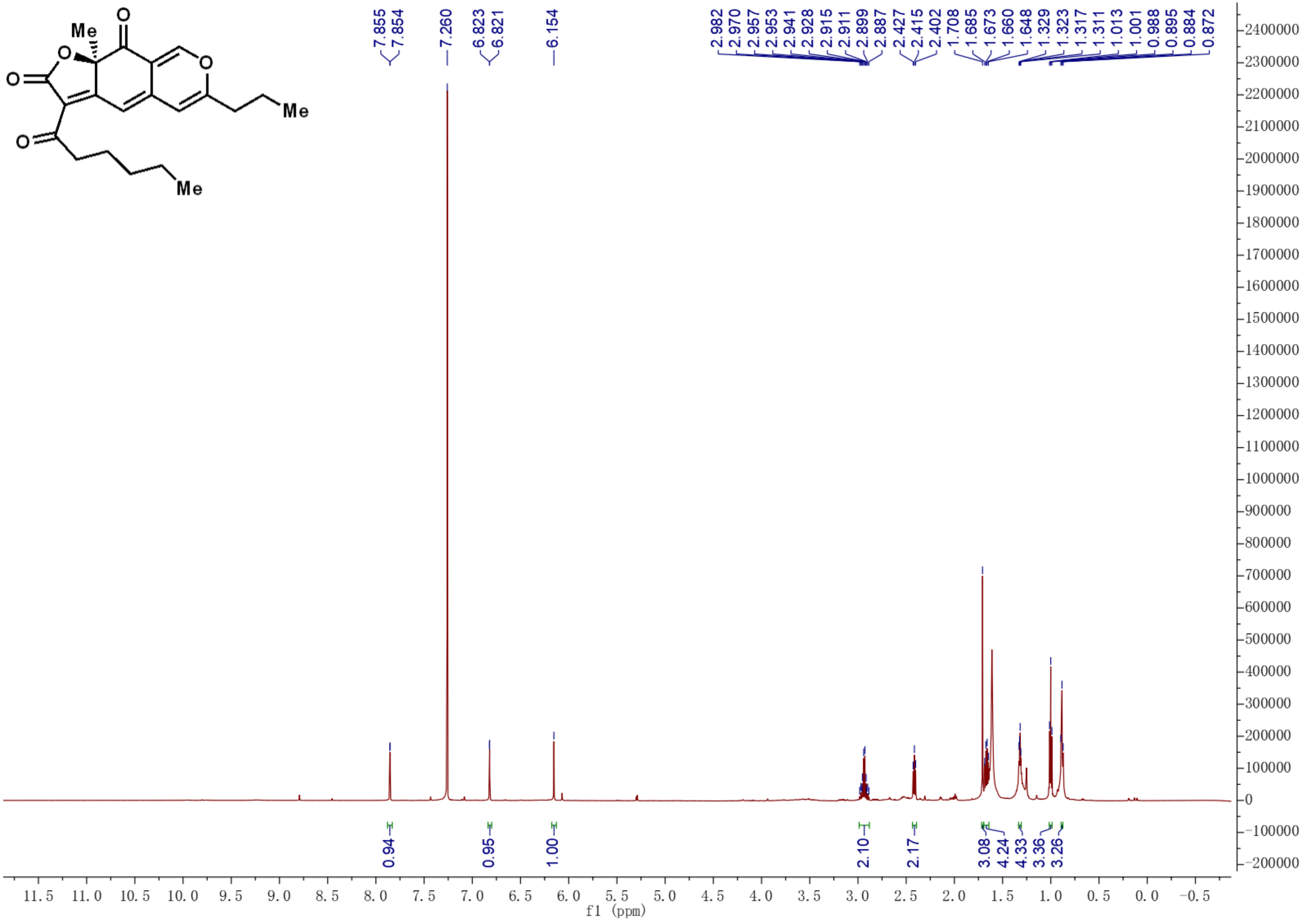

19: ^{13}C NMR (150 MHz, $CDCl_3$)

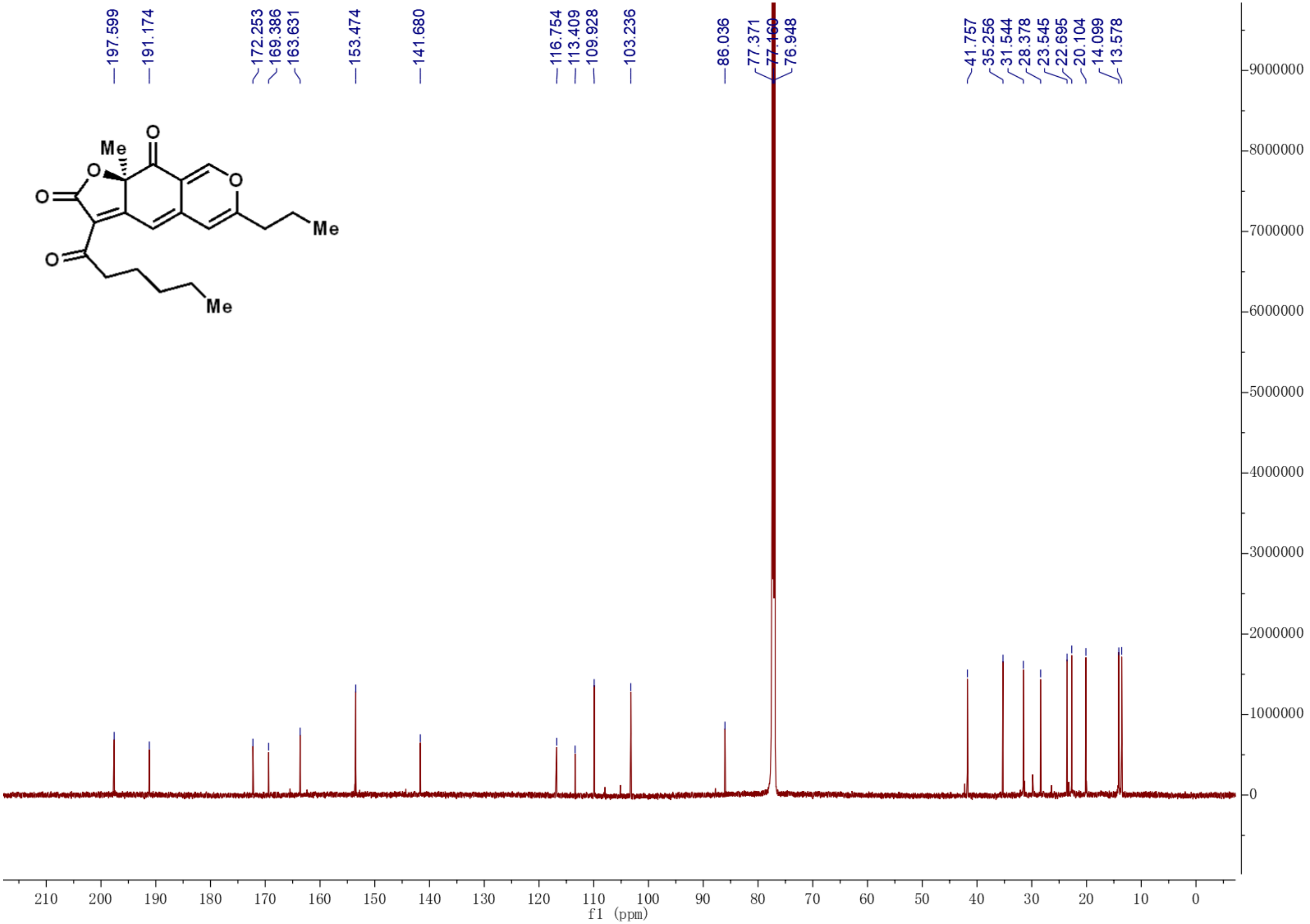

33: ^{1}H NMR (600 MHz, $CDCl_3$)

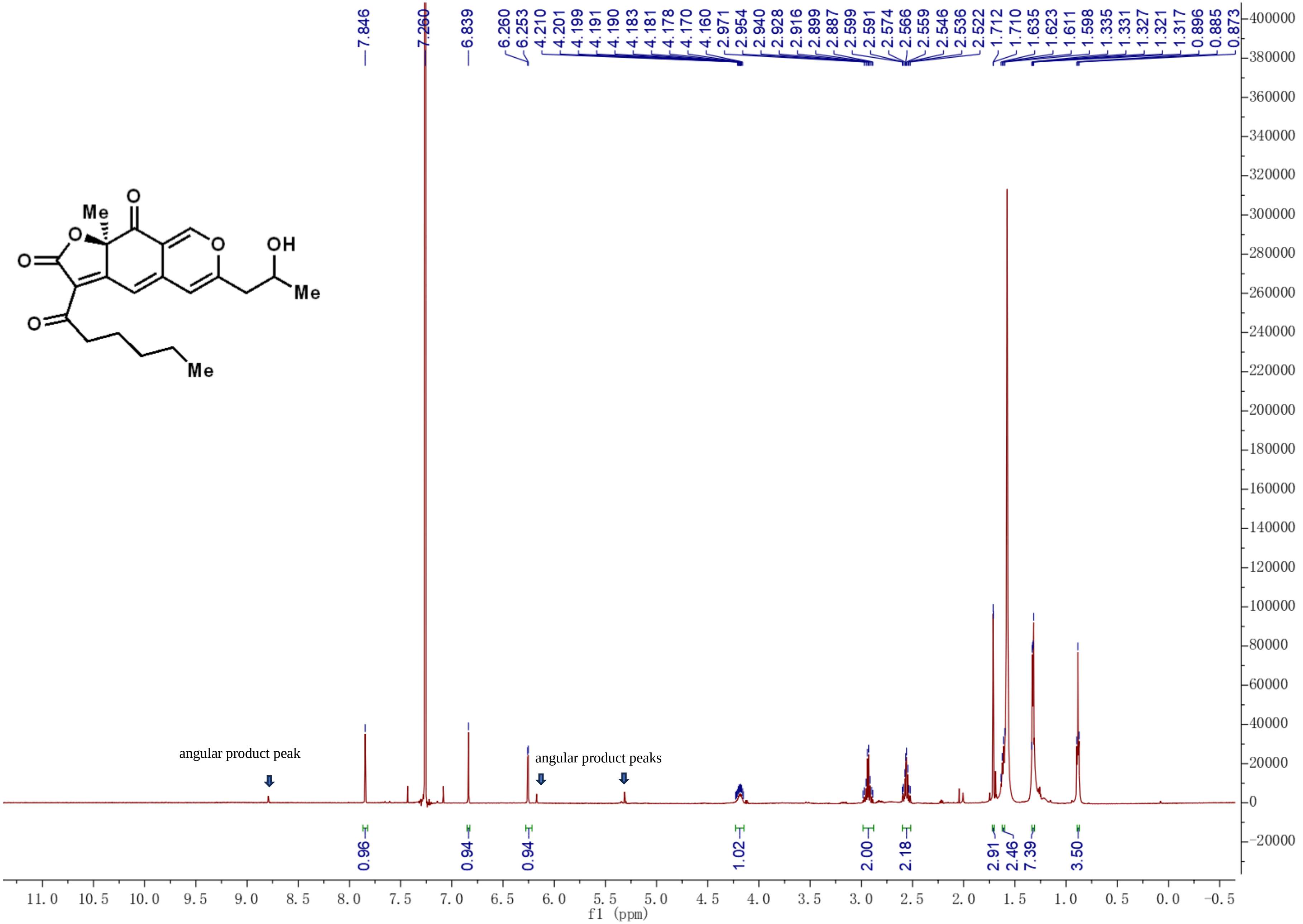

33: ^{13}C NMR (150 MHz, $CDCl_3$)

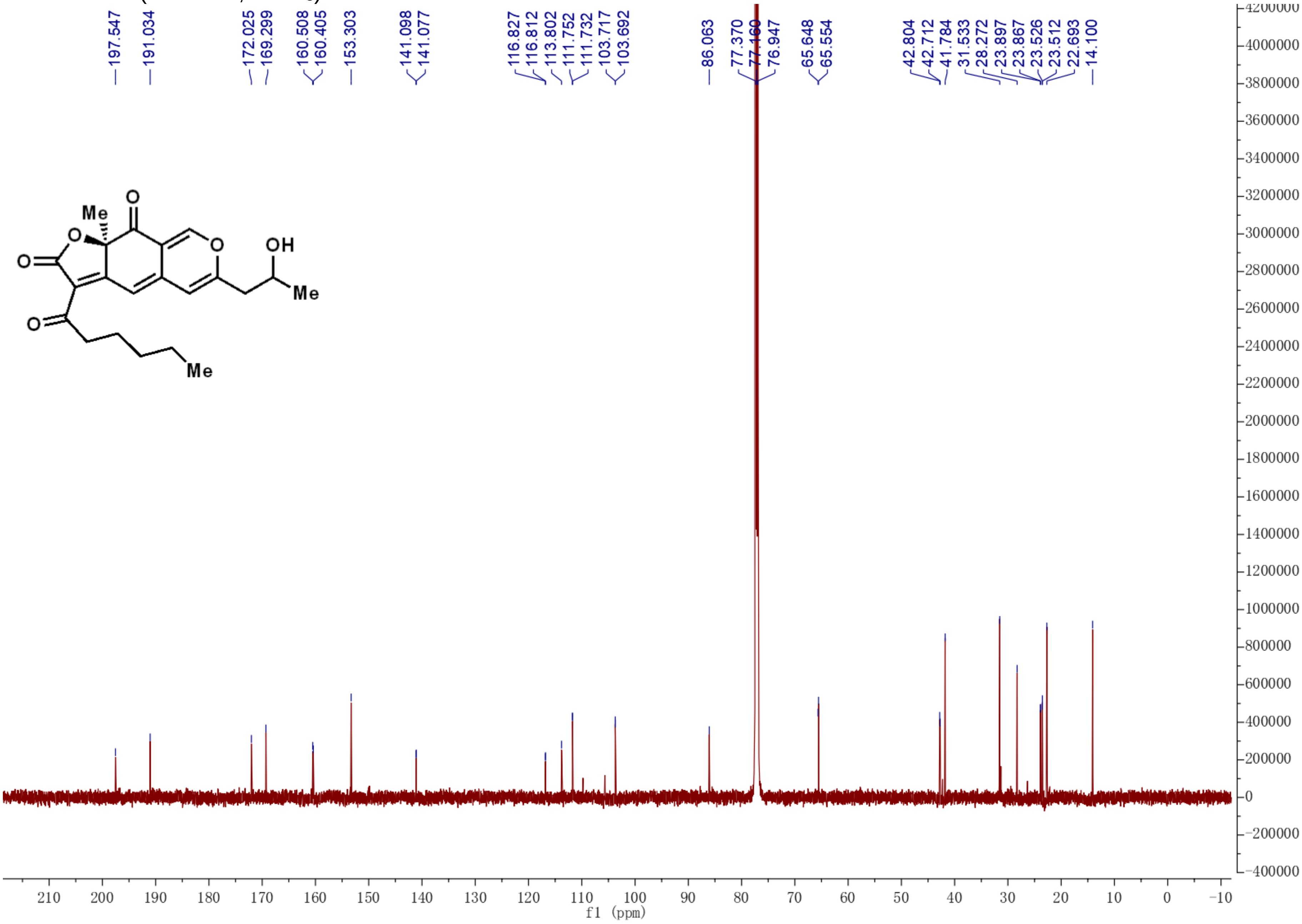

34: ^{1}H NMR (600 MHz, $CDCl_3$)

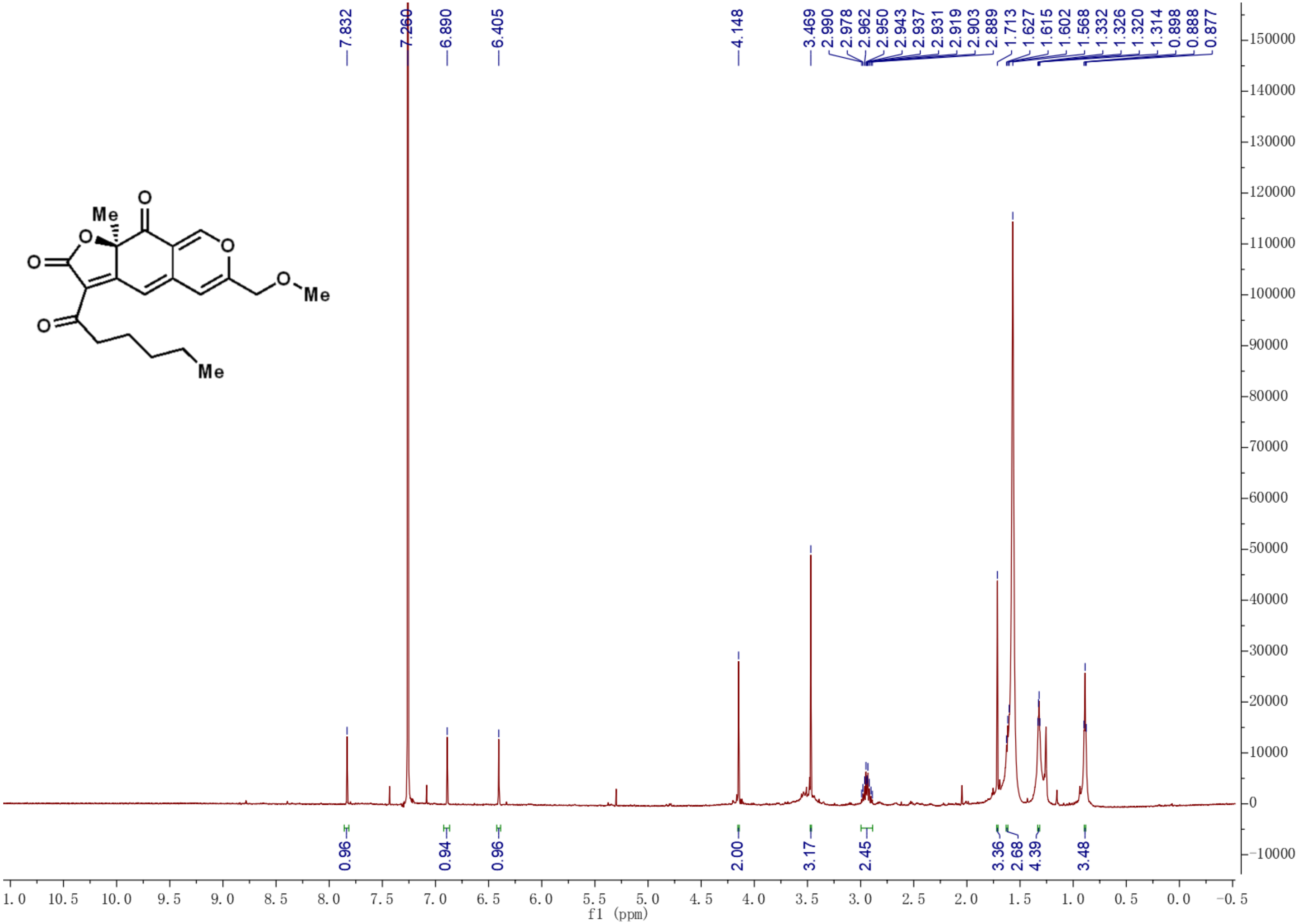

34: ^{13}C NMR (150 MHz, $CDCl_3$)

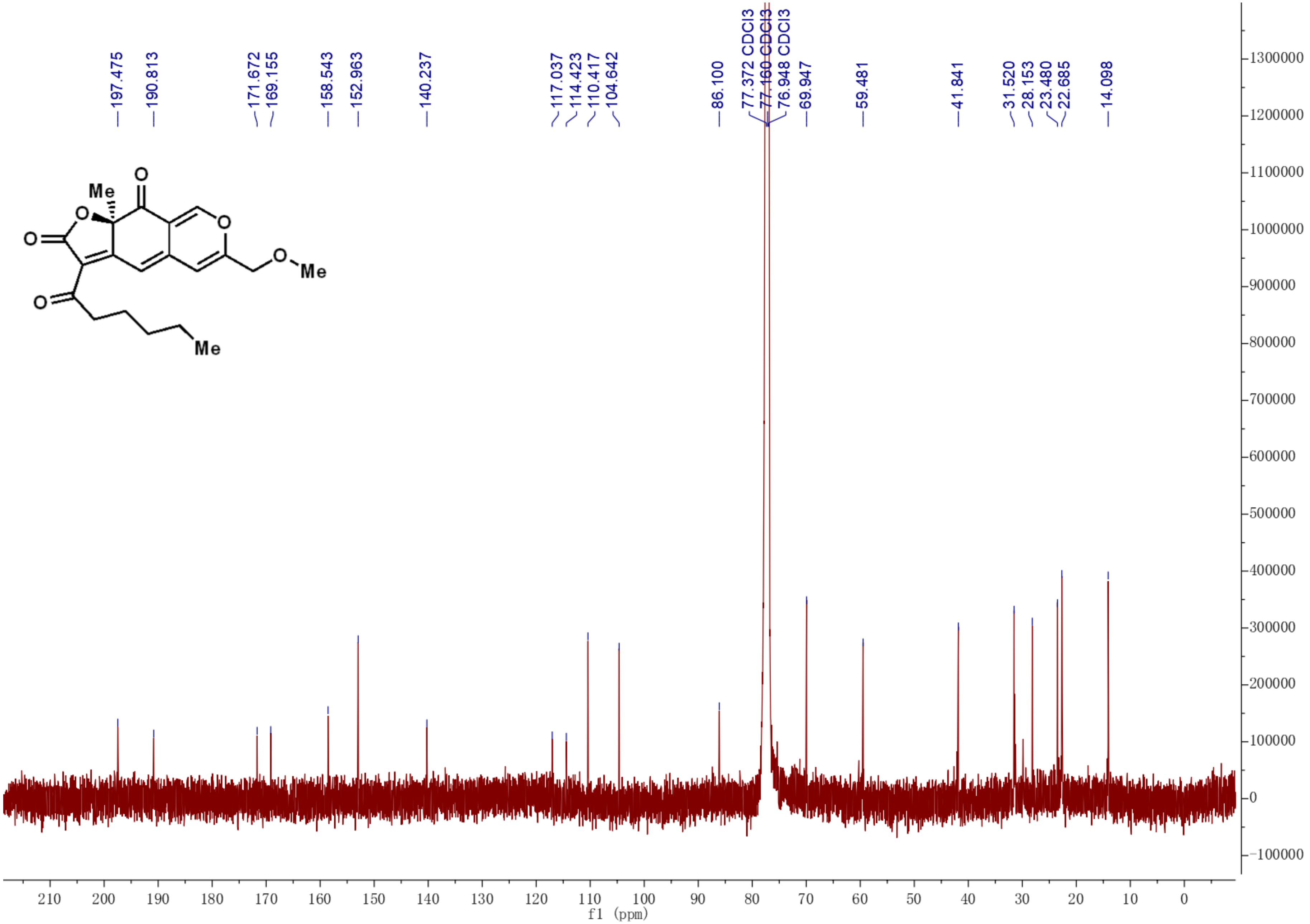

8: ^{1}H NMR (600 MHz, $CDCl_3$)

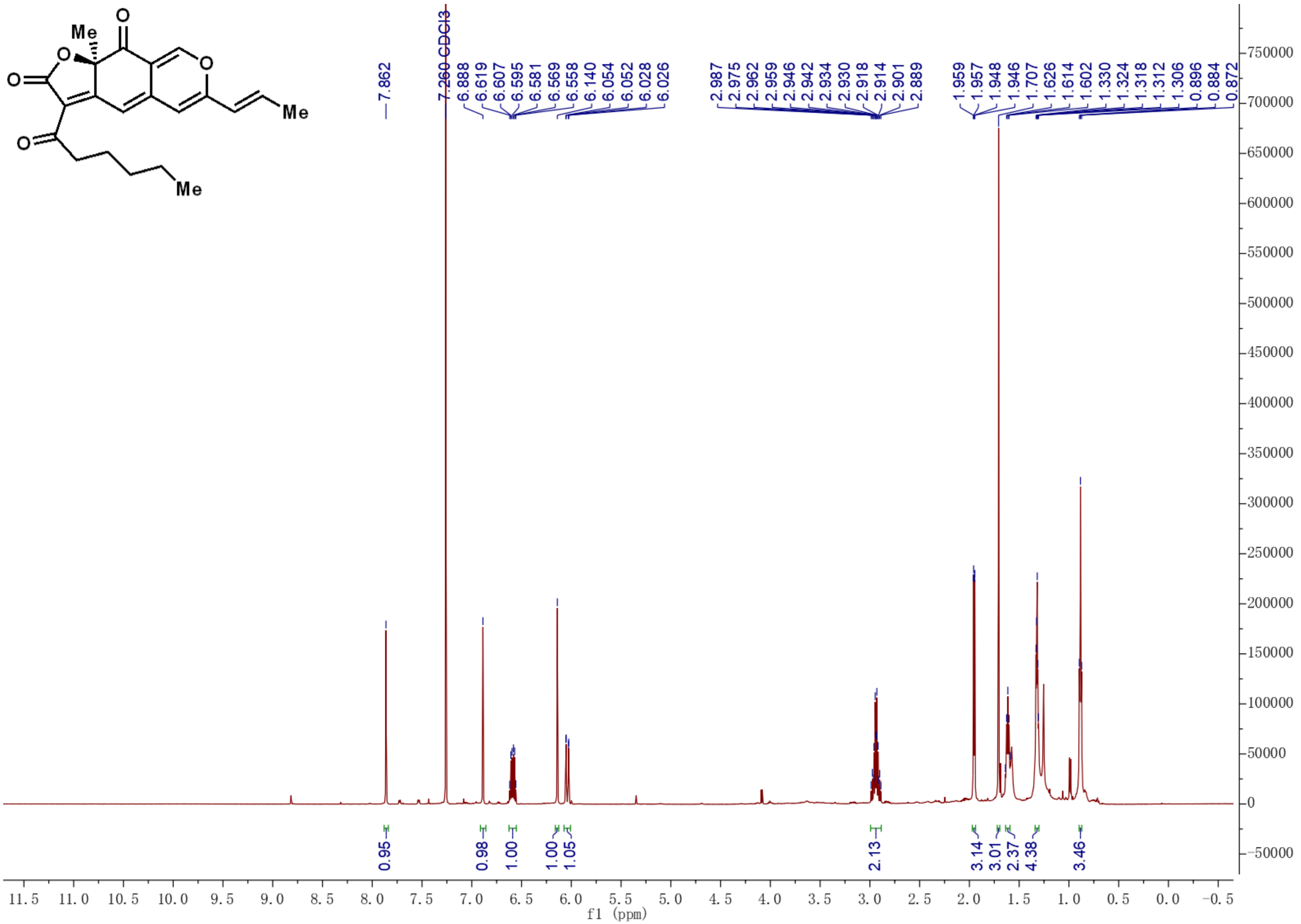

8: ^{13}C NMR (150 MHz, $CDCl_3$)

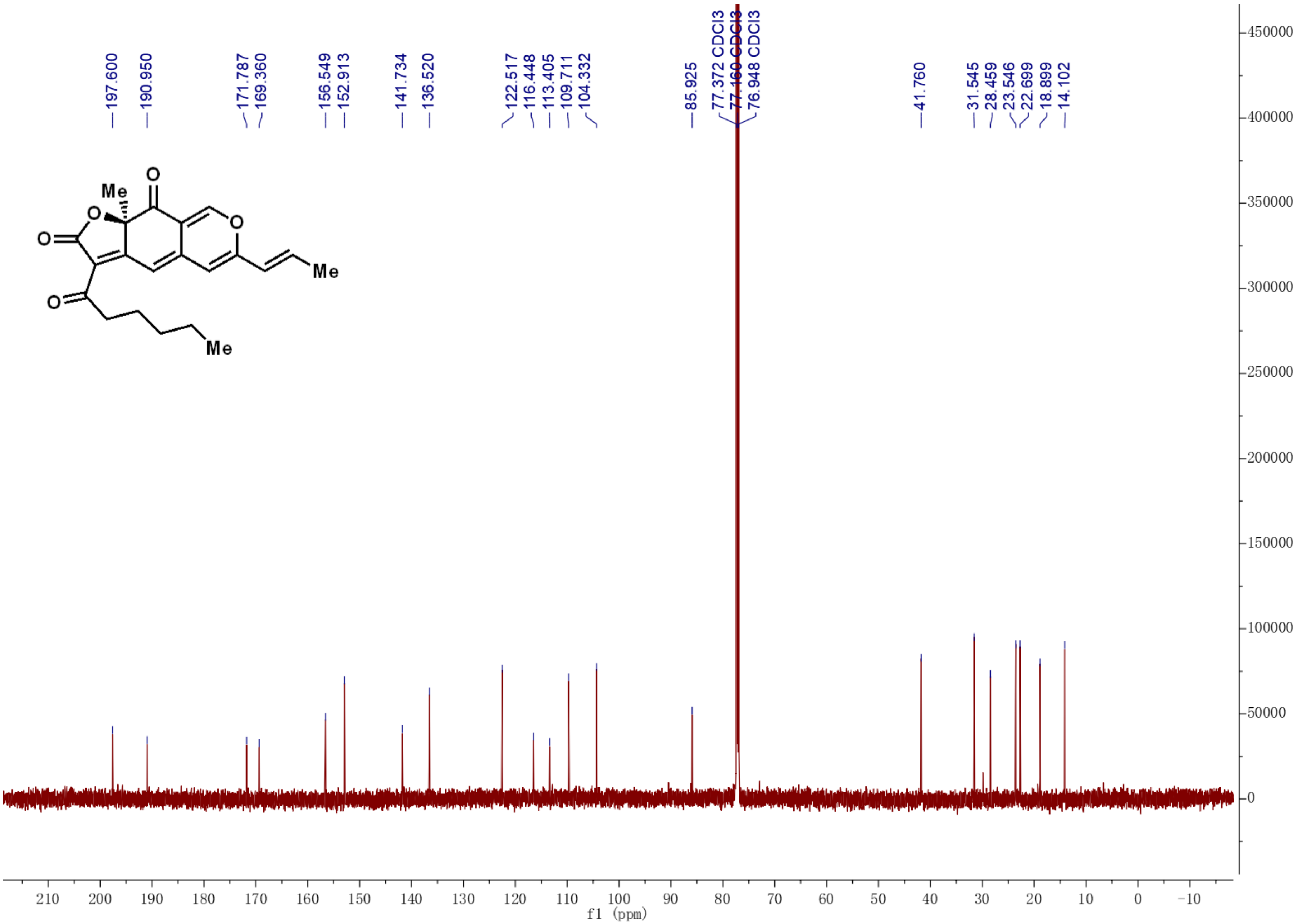

37: ^{1}H NMR (600 MHz, $CDCl_3$)

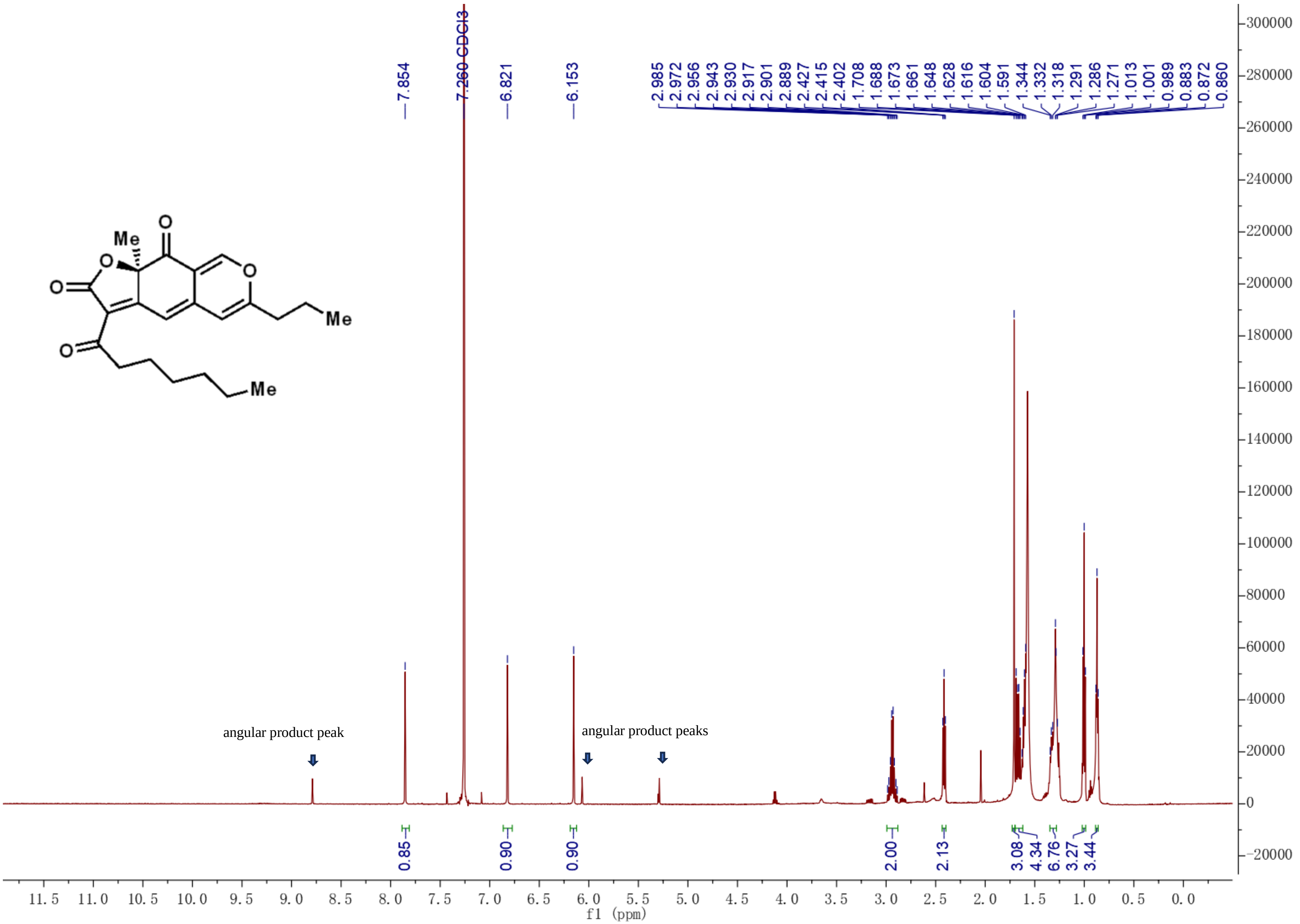

37: ^{13}C NMR (150 MHz, $CDCl_3$)

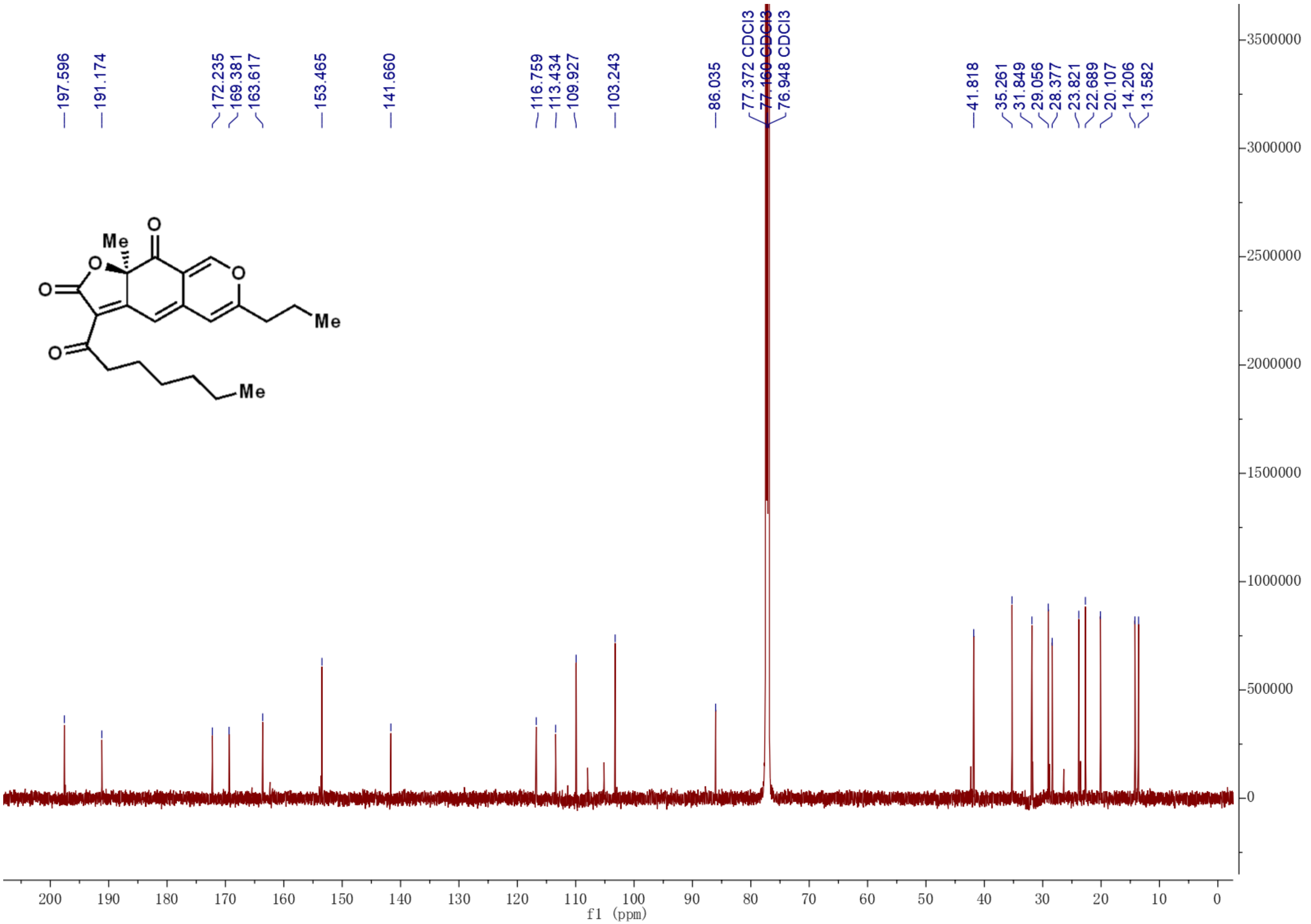

38: ^{1}H NMR (600 MHz, $CDCl_3$)

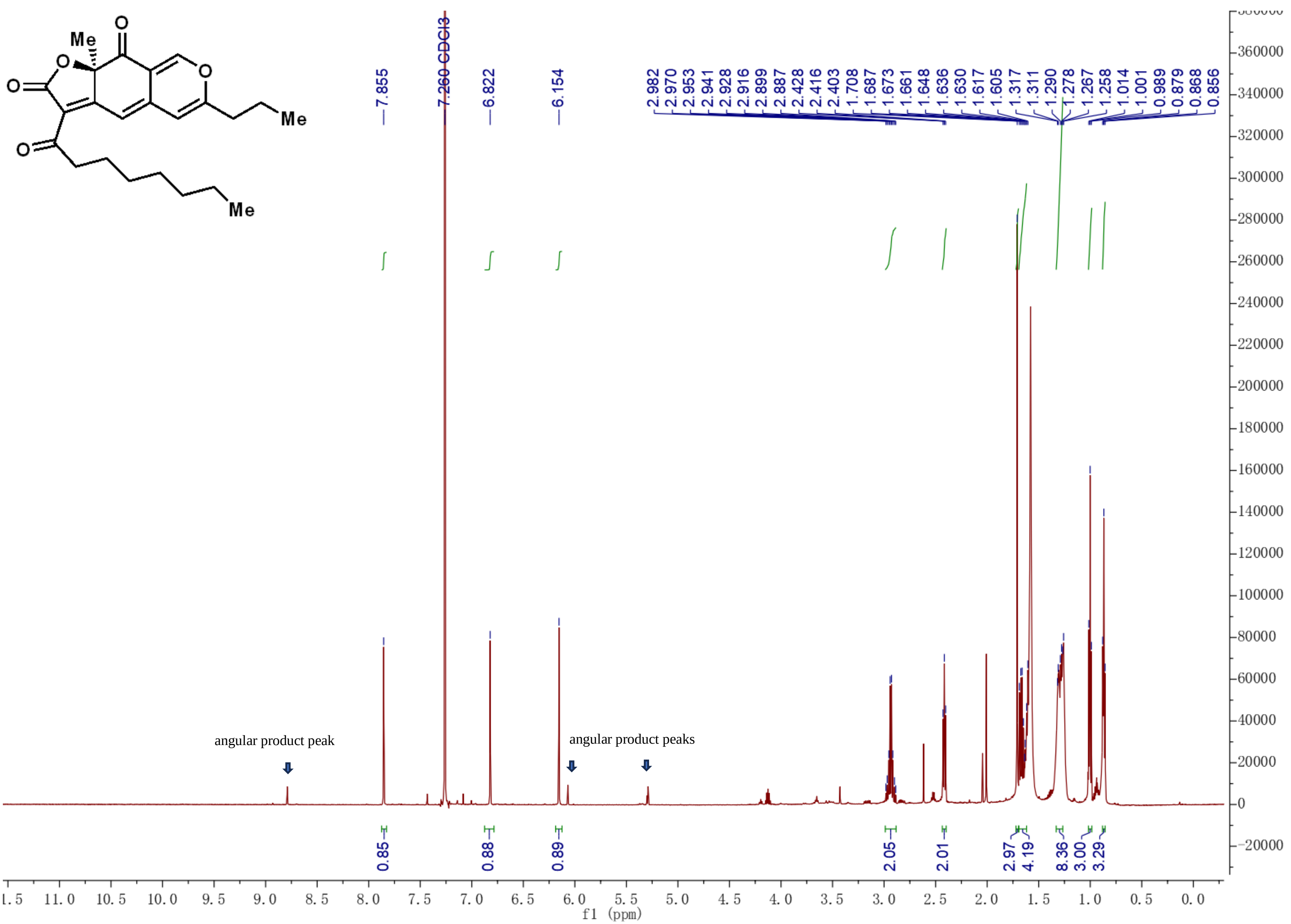

38: ^{13}C NMR (150 MHz, $CDCl_3$)

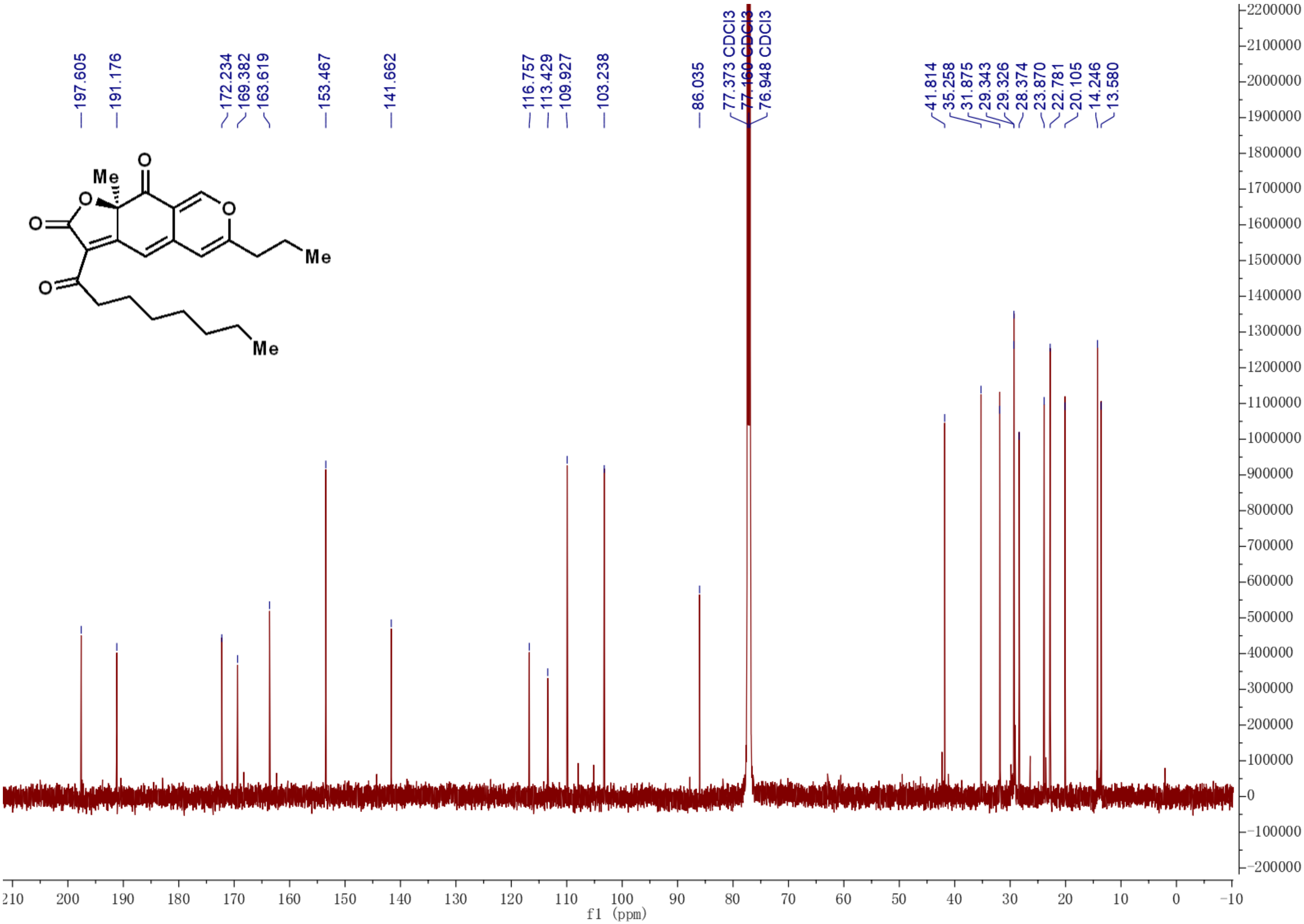

44: ^{1}H NMR (600 MHz, $CDCl_3$)

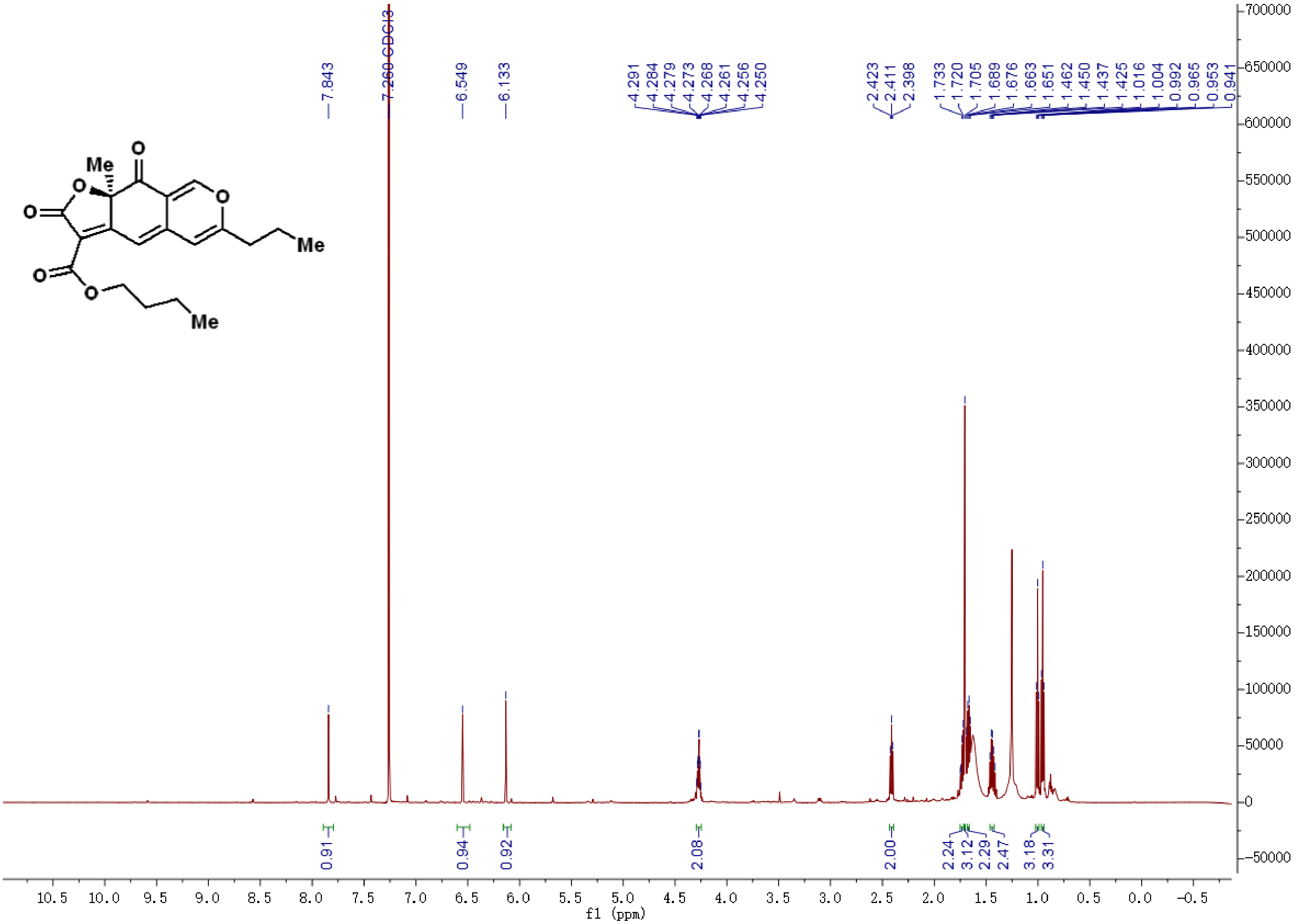

44: ^{13}C NMR (150 MHz, $CDCl_3$)

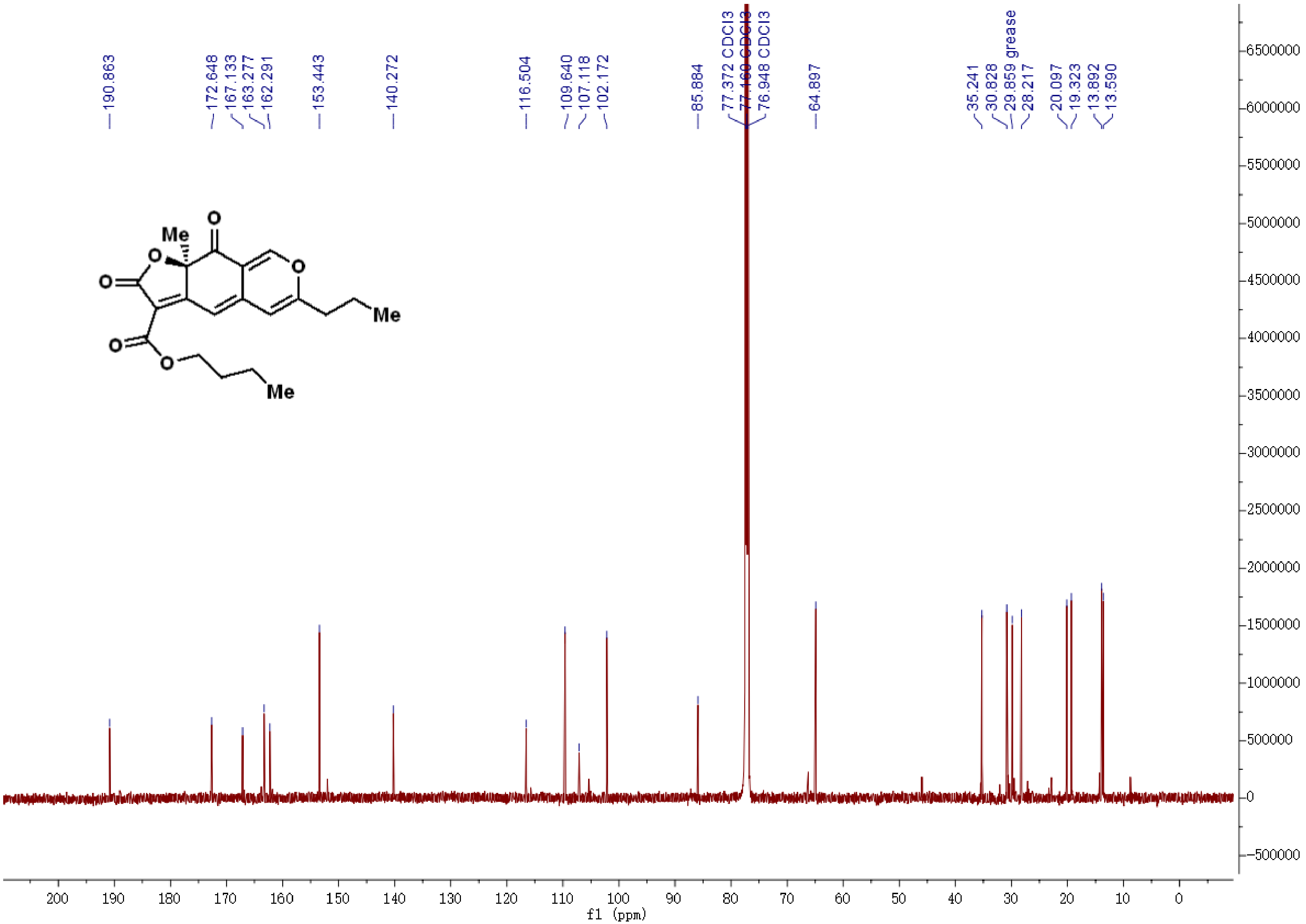

46: ^{1}H NMR (600 MHz, $CDCl_3$)

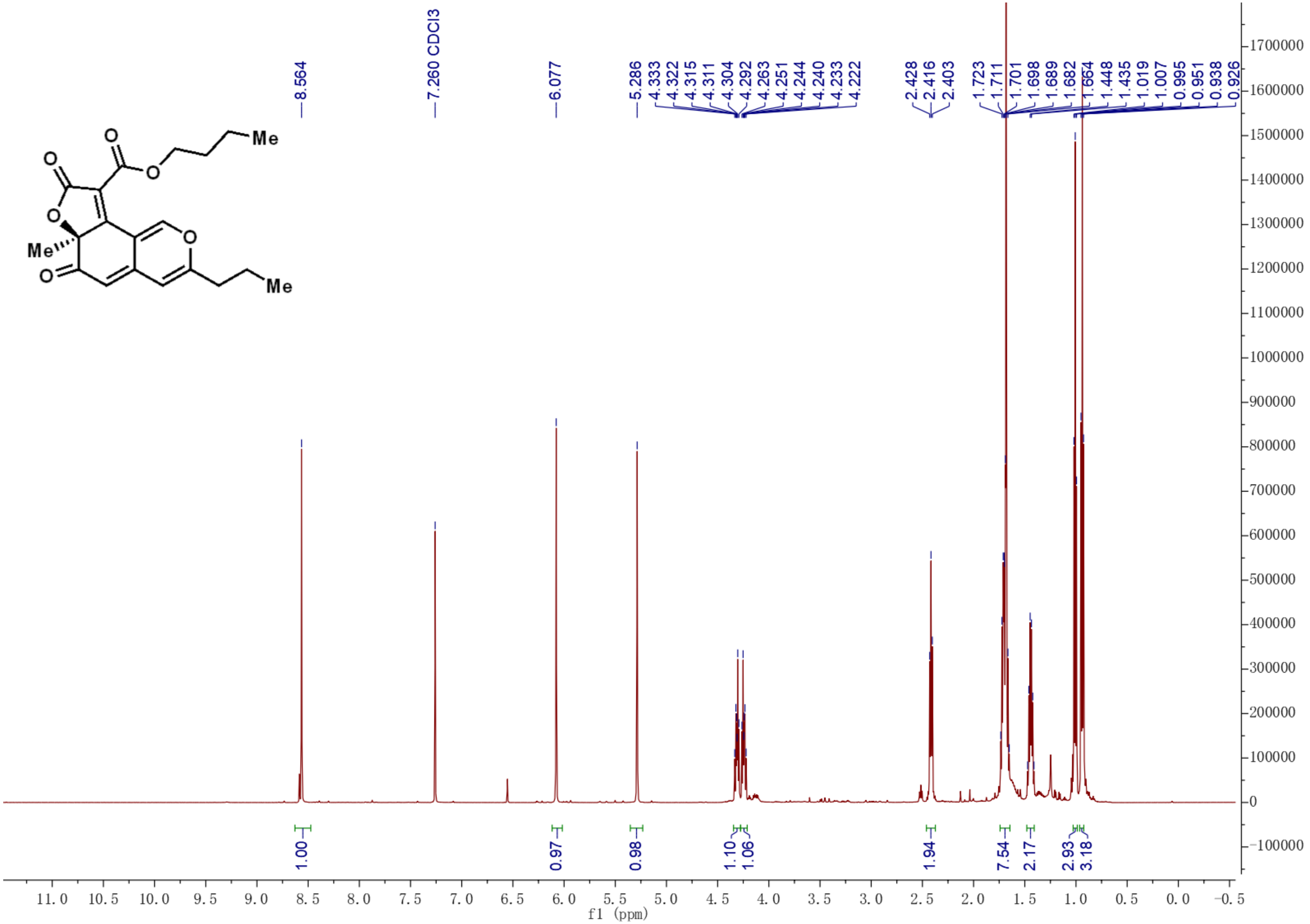

46: ^{13}C NMR (150 MHz, $CDCl_3$)

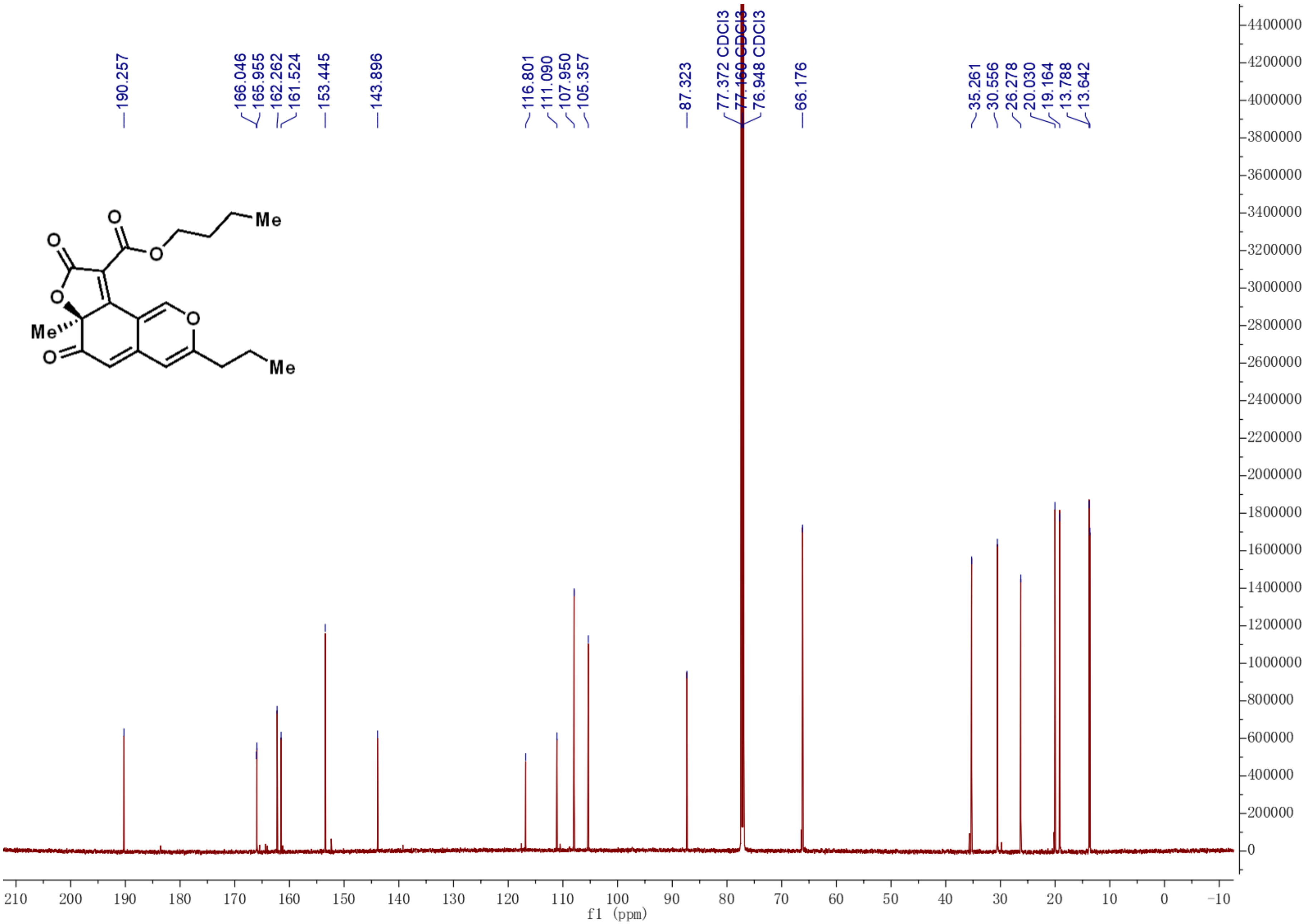

9: ^{1}H NMR (600 MHz, $CDCl_3$)

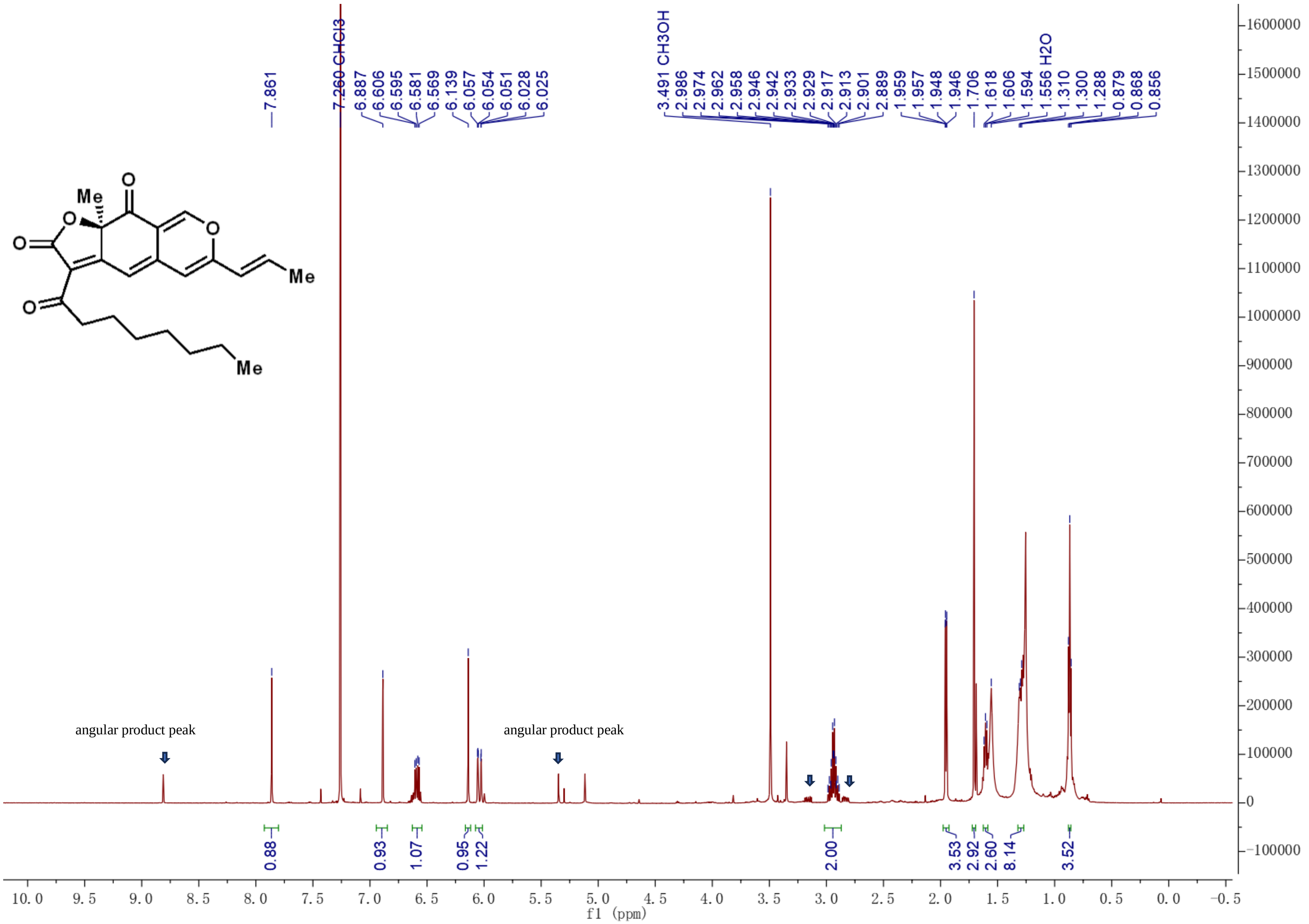

9: ^{13}C NMR (150 MHz, $CDCl_3$)

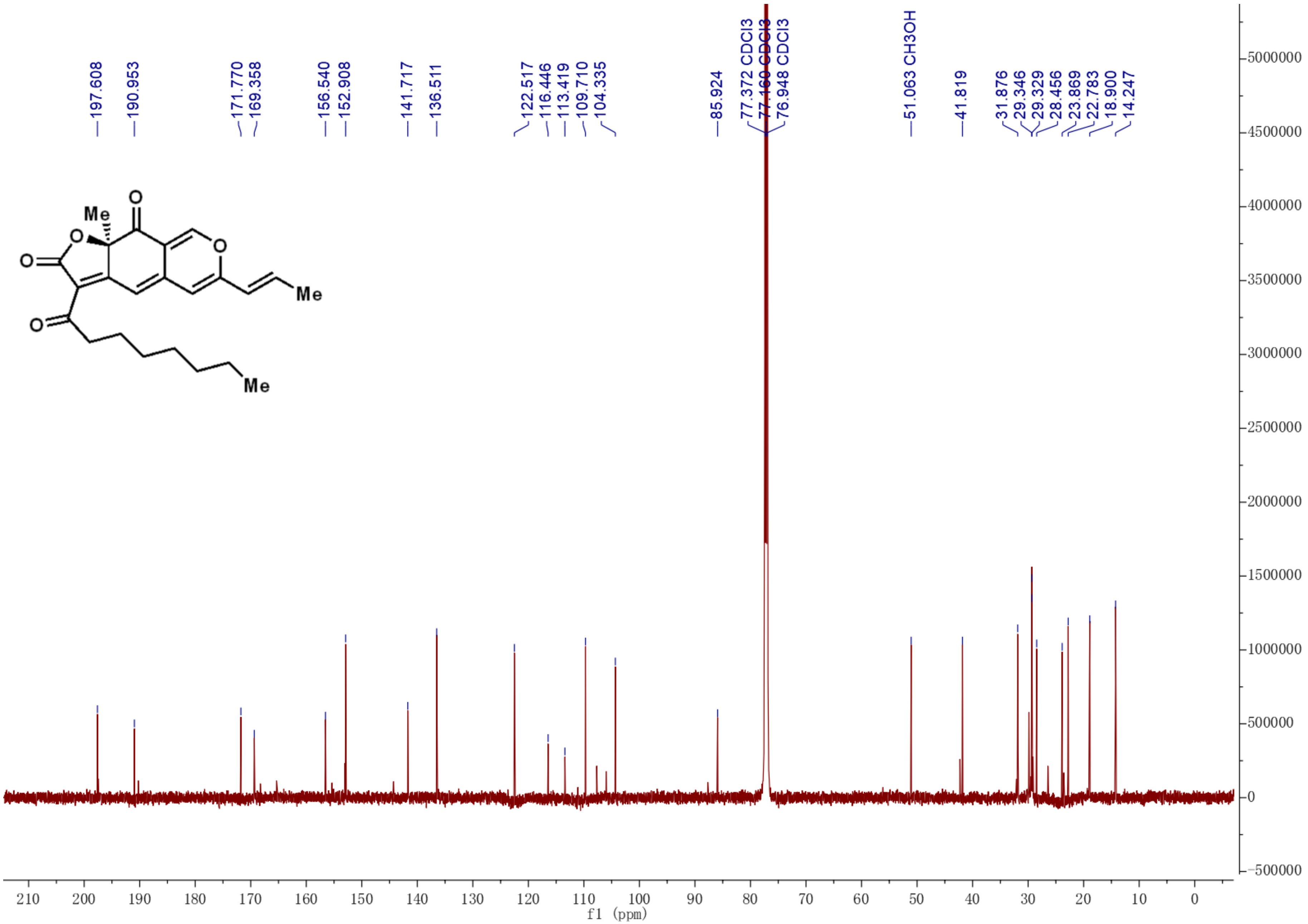

41: ^{1}H NMR (600 MHz, $CDCl_3$)

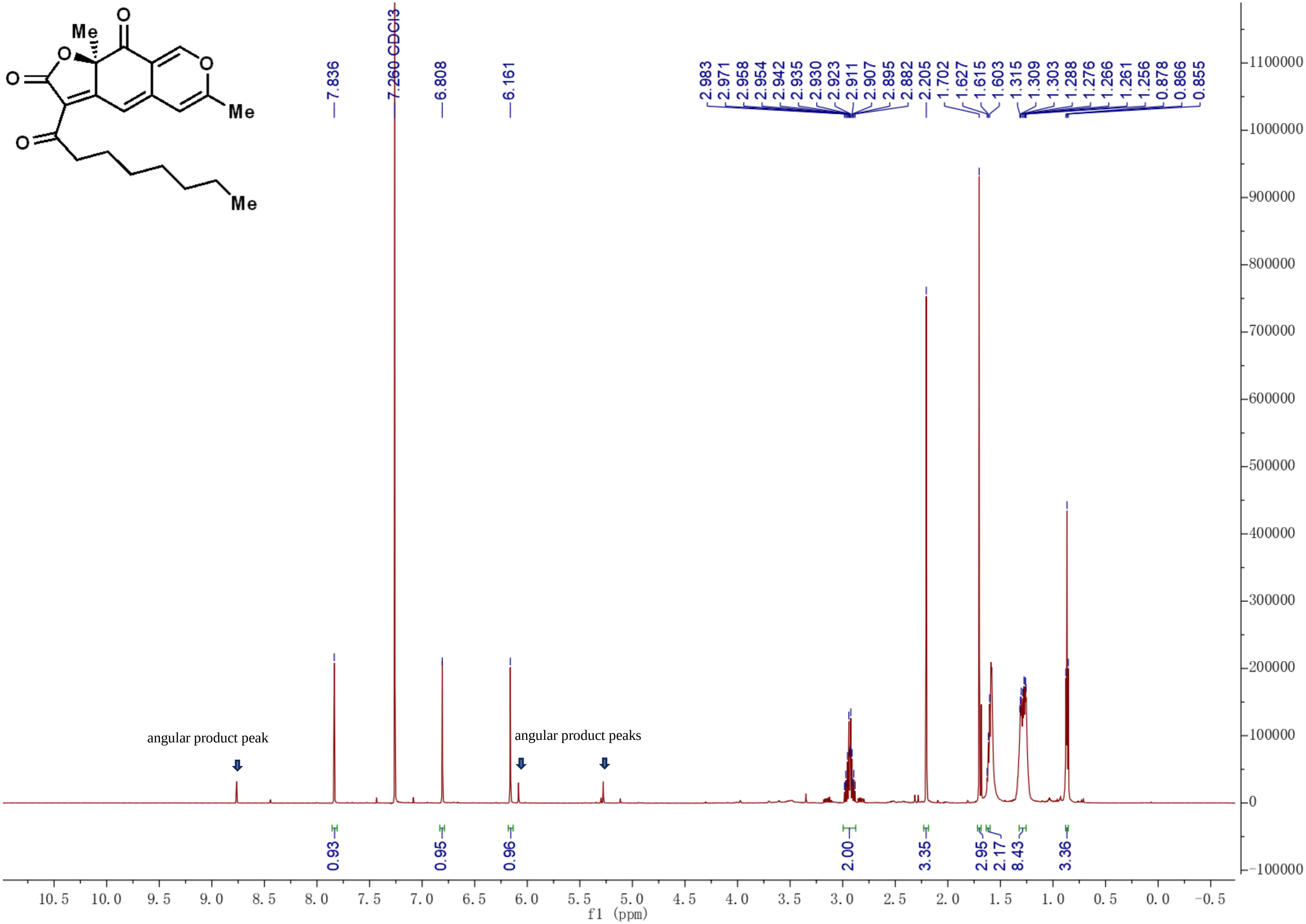

41: ^{13}C NMR (150 MHz, $CDCl_3$)

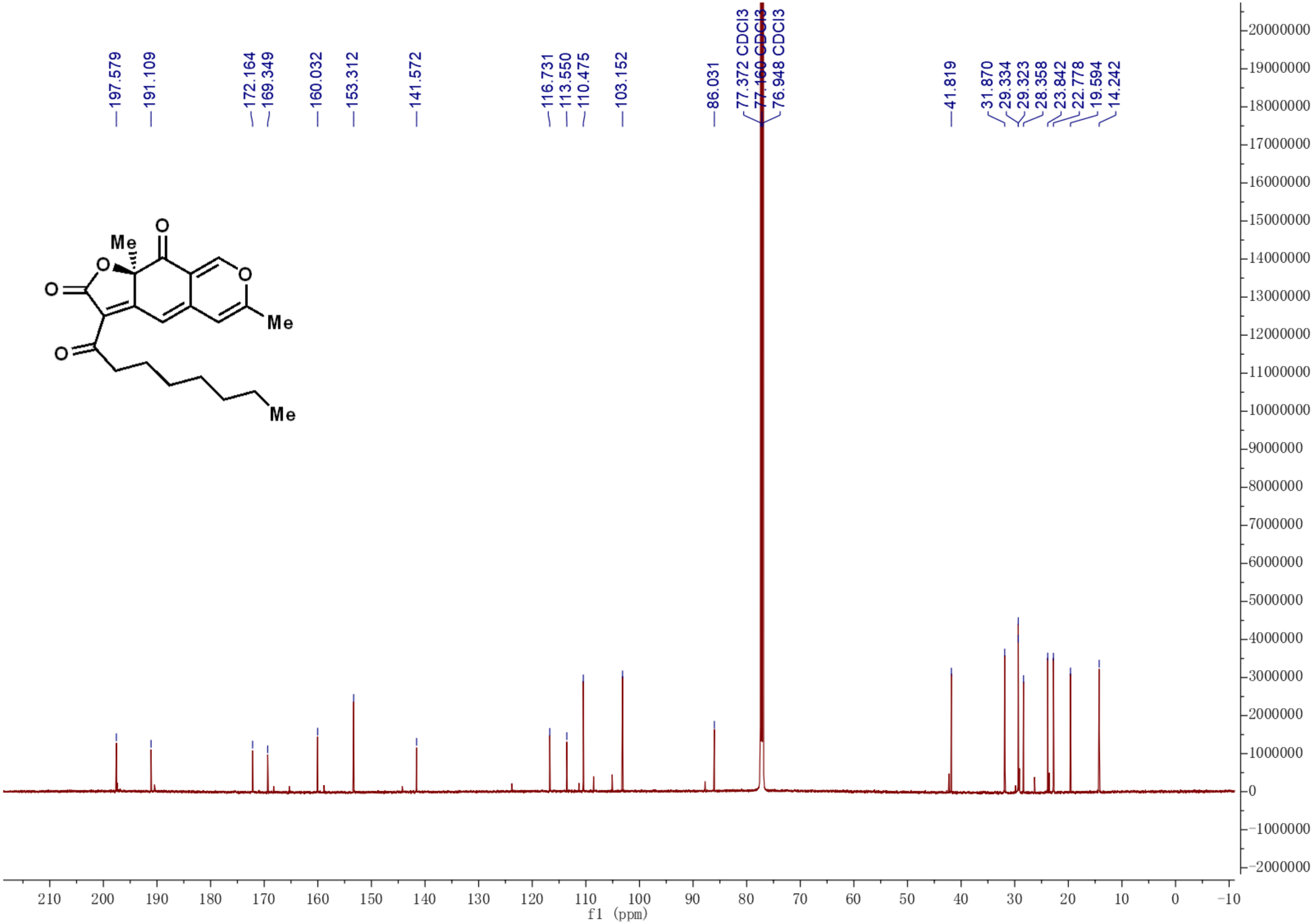

40: ^{1}H NMR (600 MHz, $CDCl_3$)

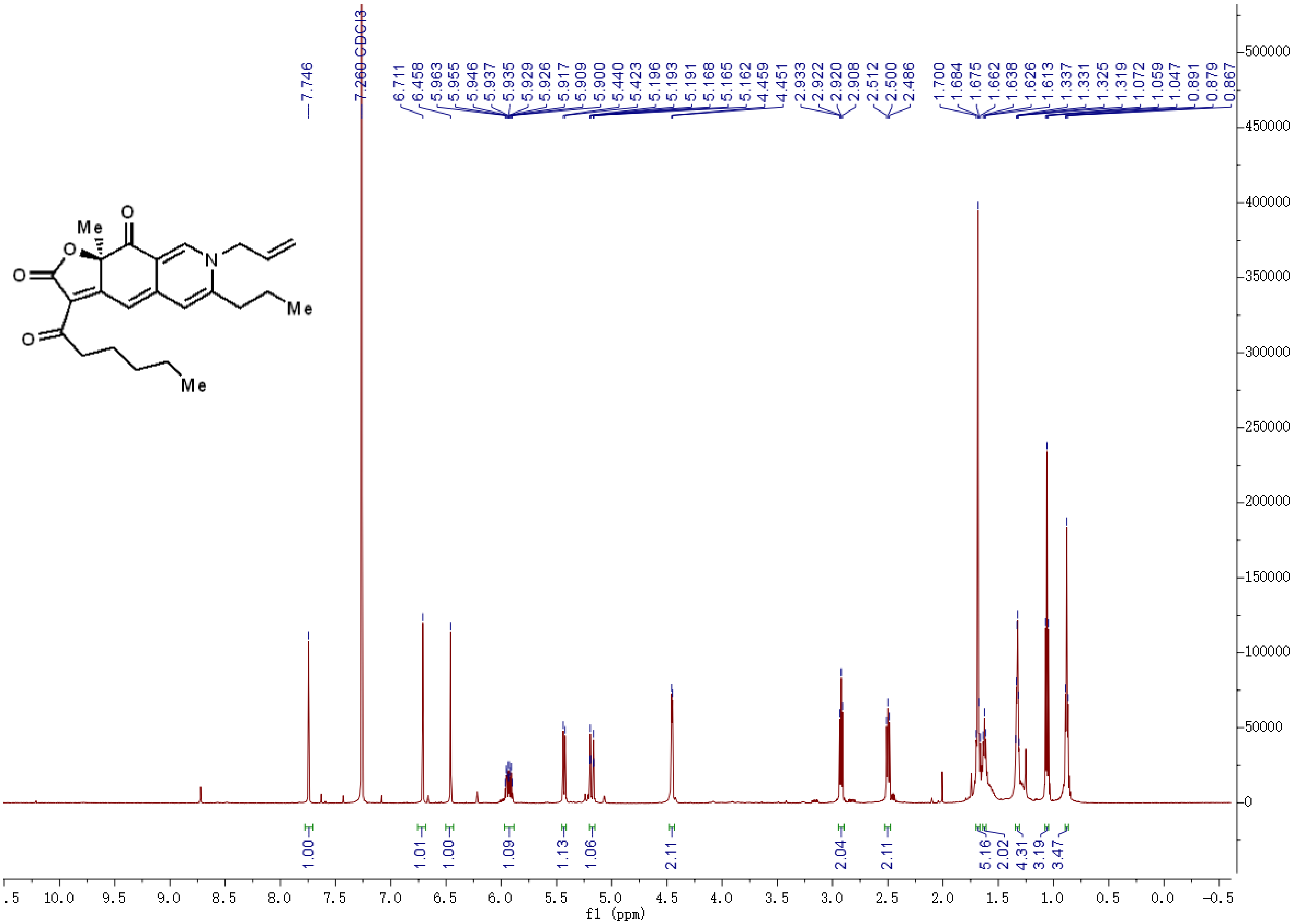

40: ^{13}C NMR (150 MHz, $CDCl_3$)

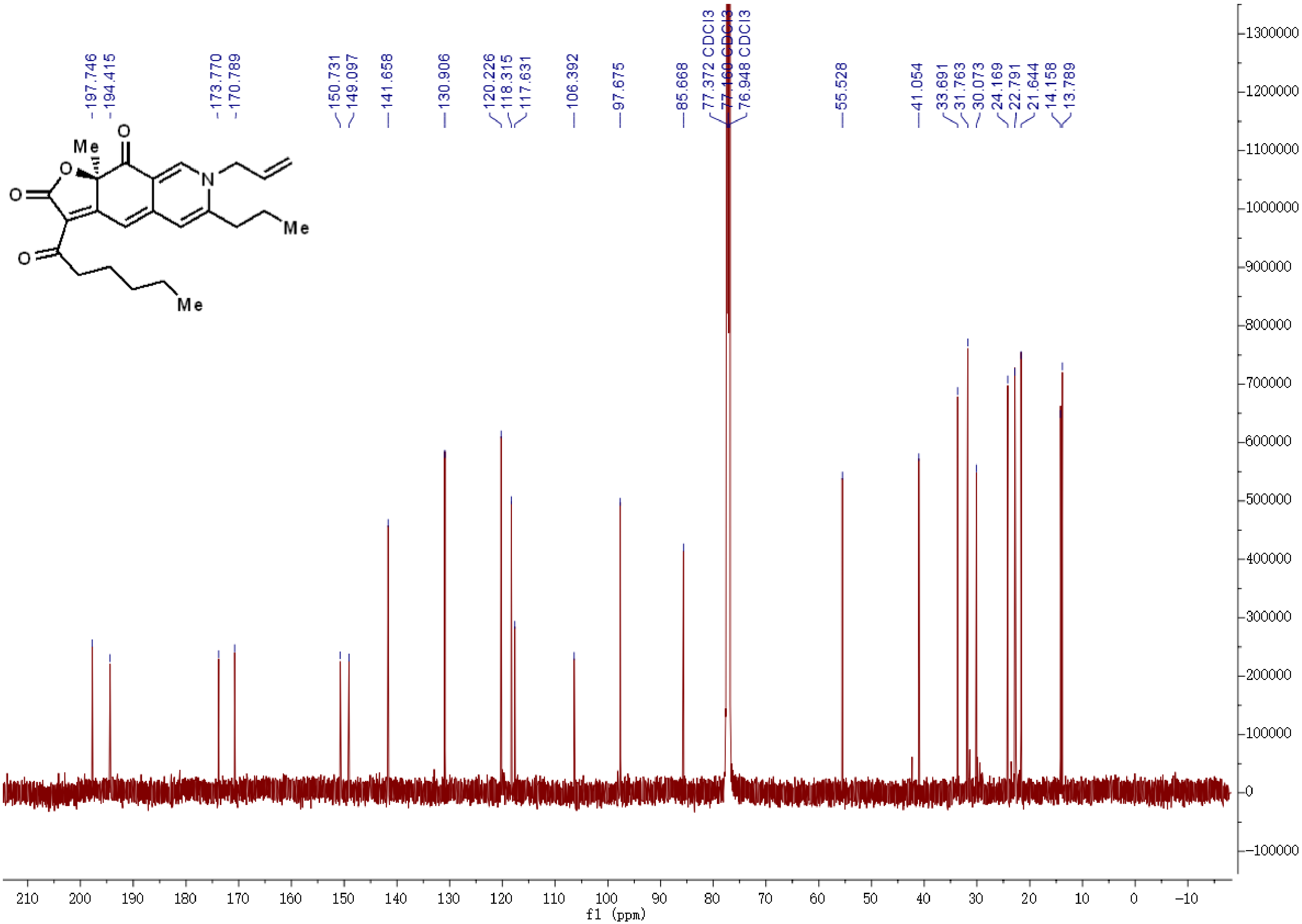

29: ^{1}H NMR (600 MHz, $CDCl_3$)

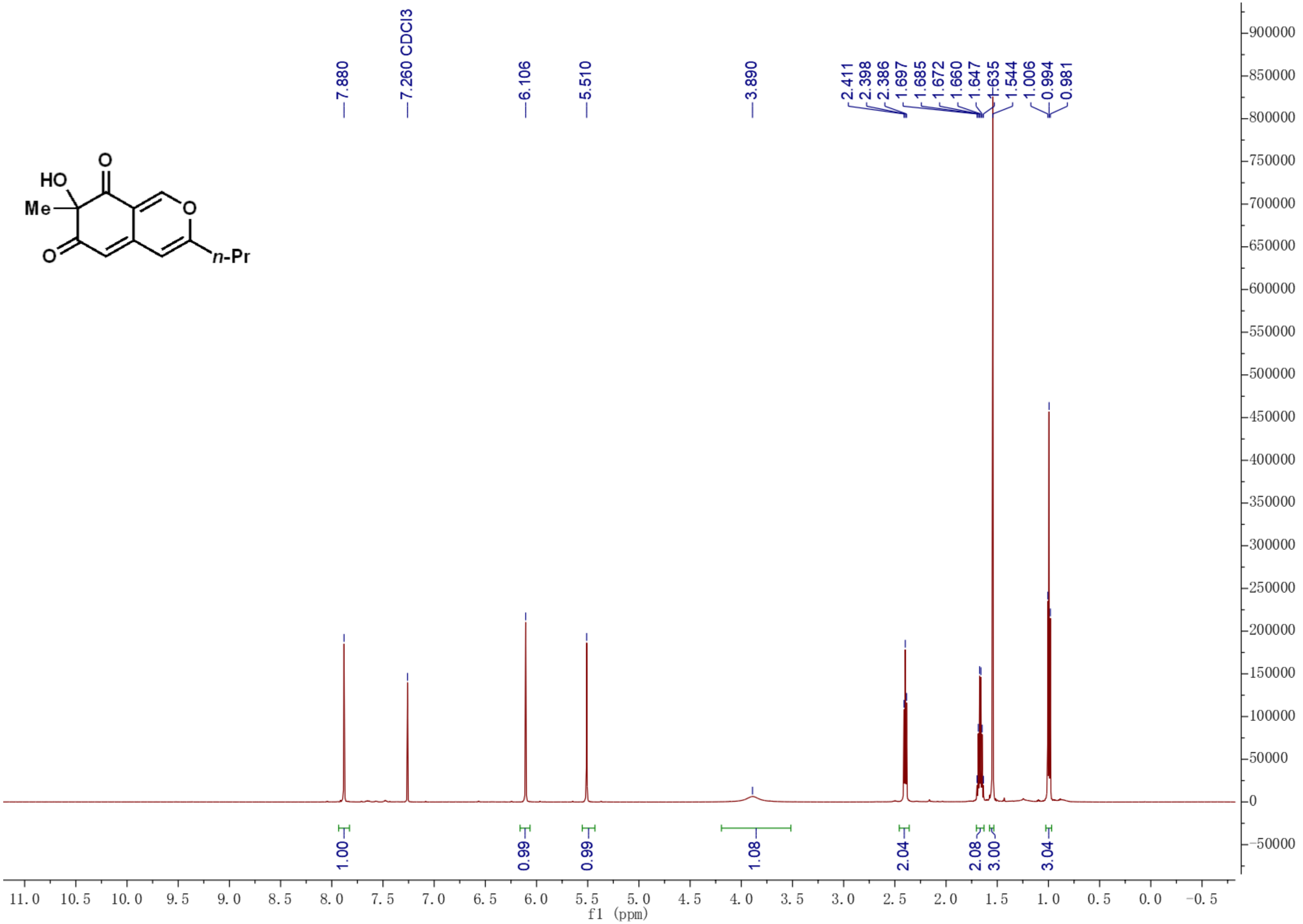

21: ^{1}H NMR (600 MHz, $CDCl_3$)

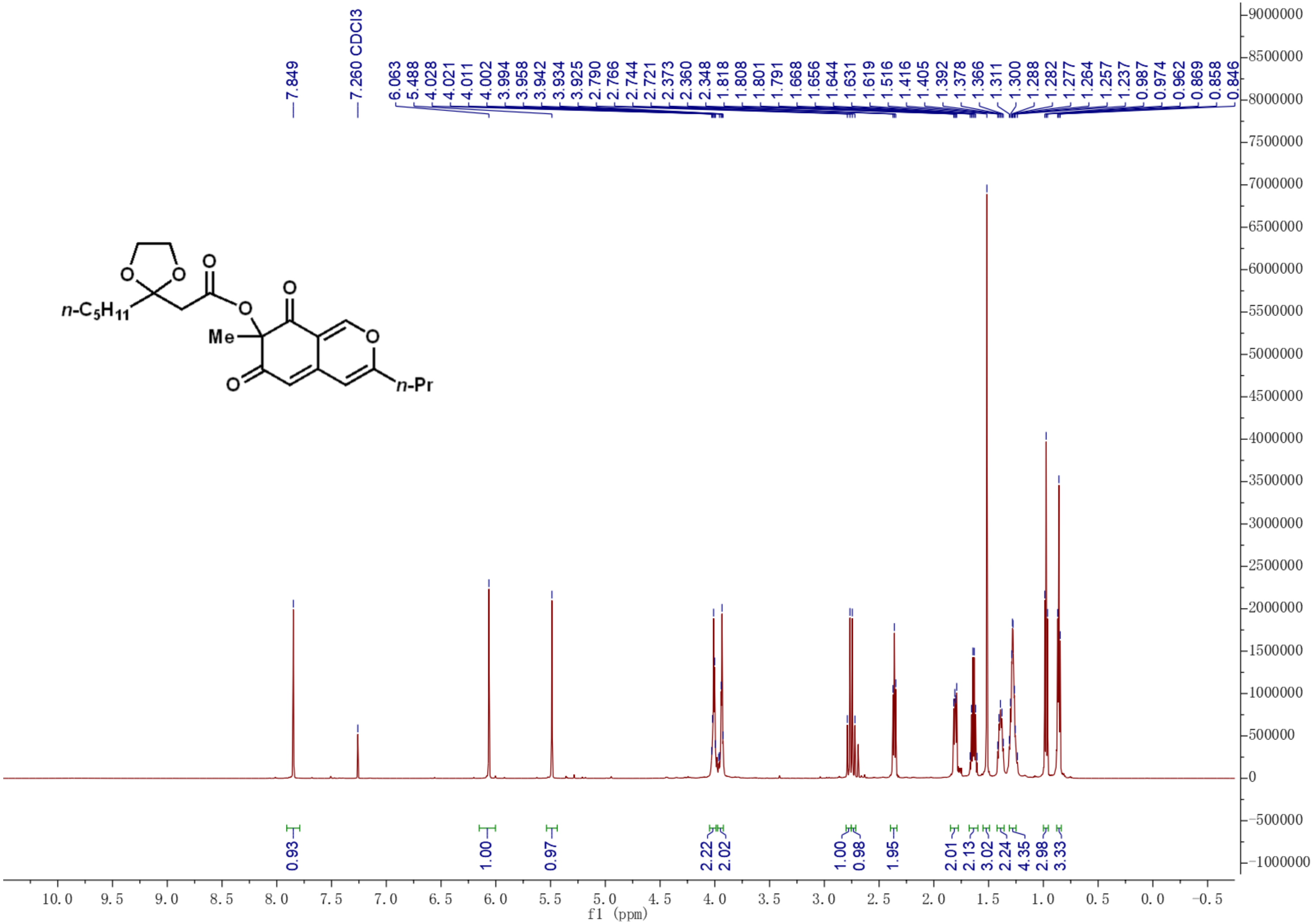

21: ^{13}C NMR (150 MHz, $CDCl_3$)

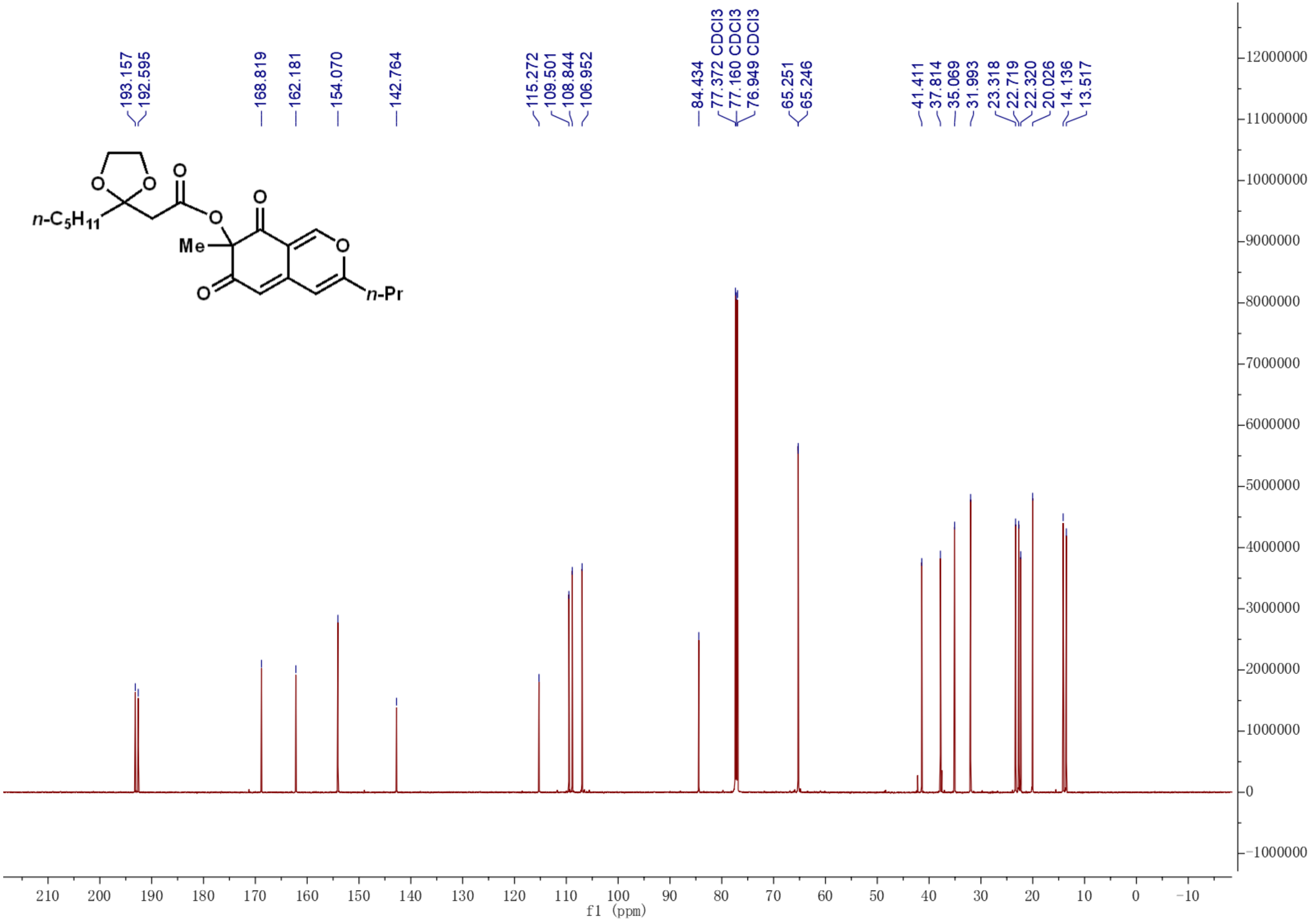

22: ^{1}H NMR (600 MHz, $CDCl_3$)

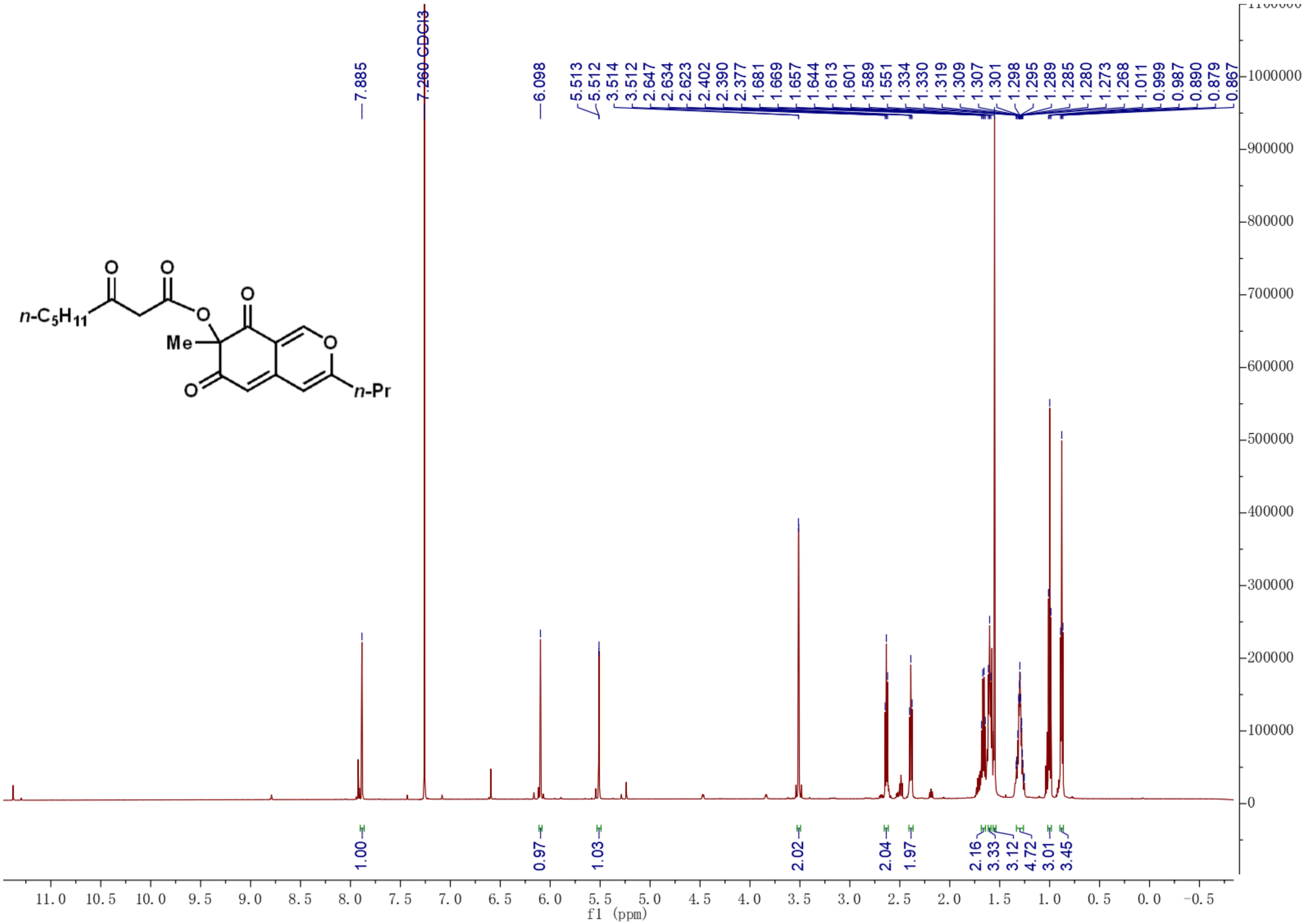

22: ^{13}C NMR (150 MHz, $CDCl_3$)

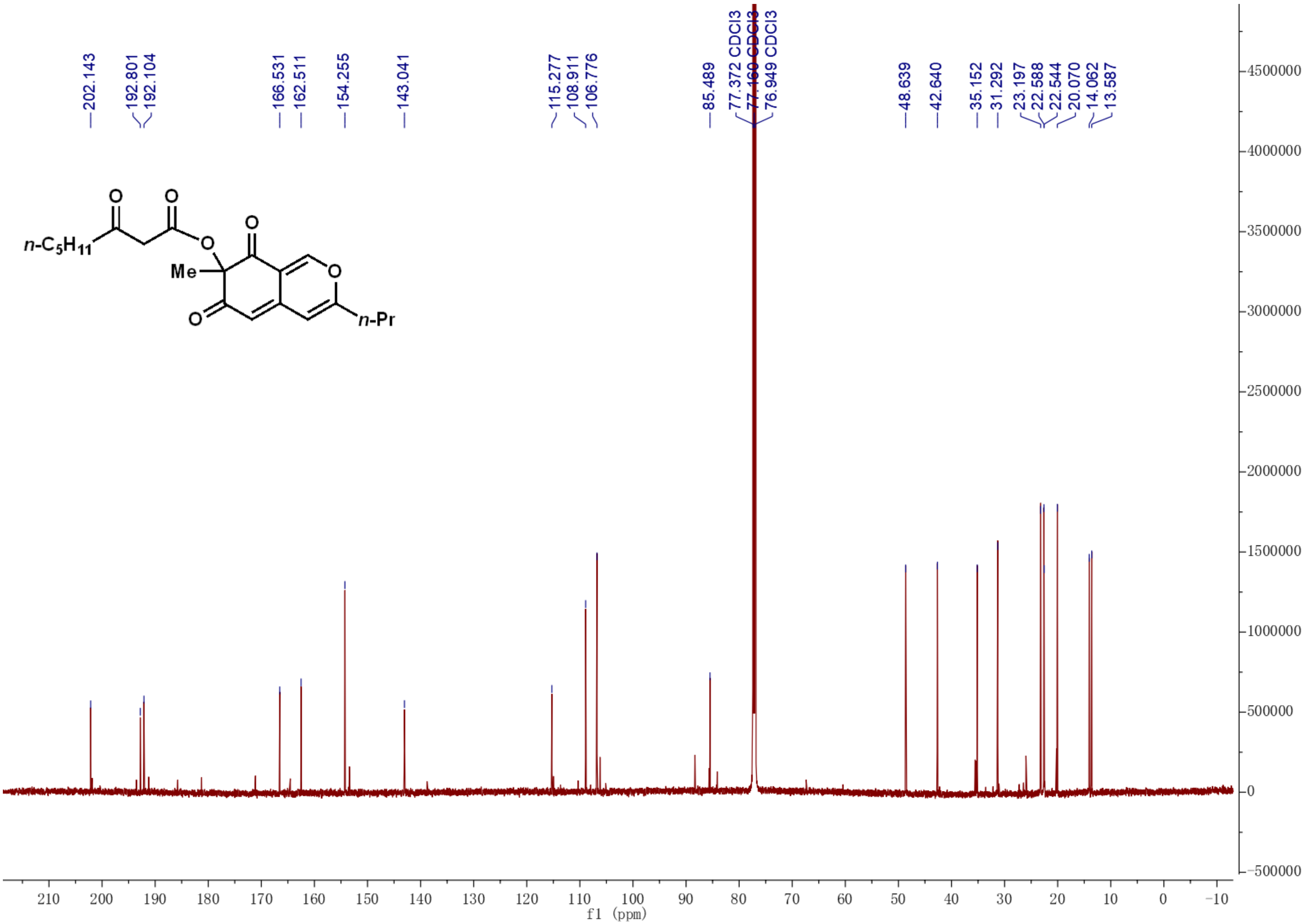

23: ^{1}H NMR (600 MHz, $CDCl_3$)

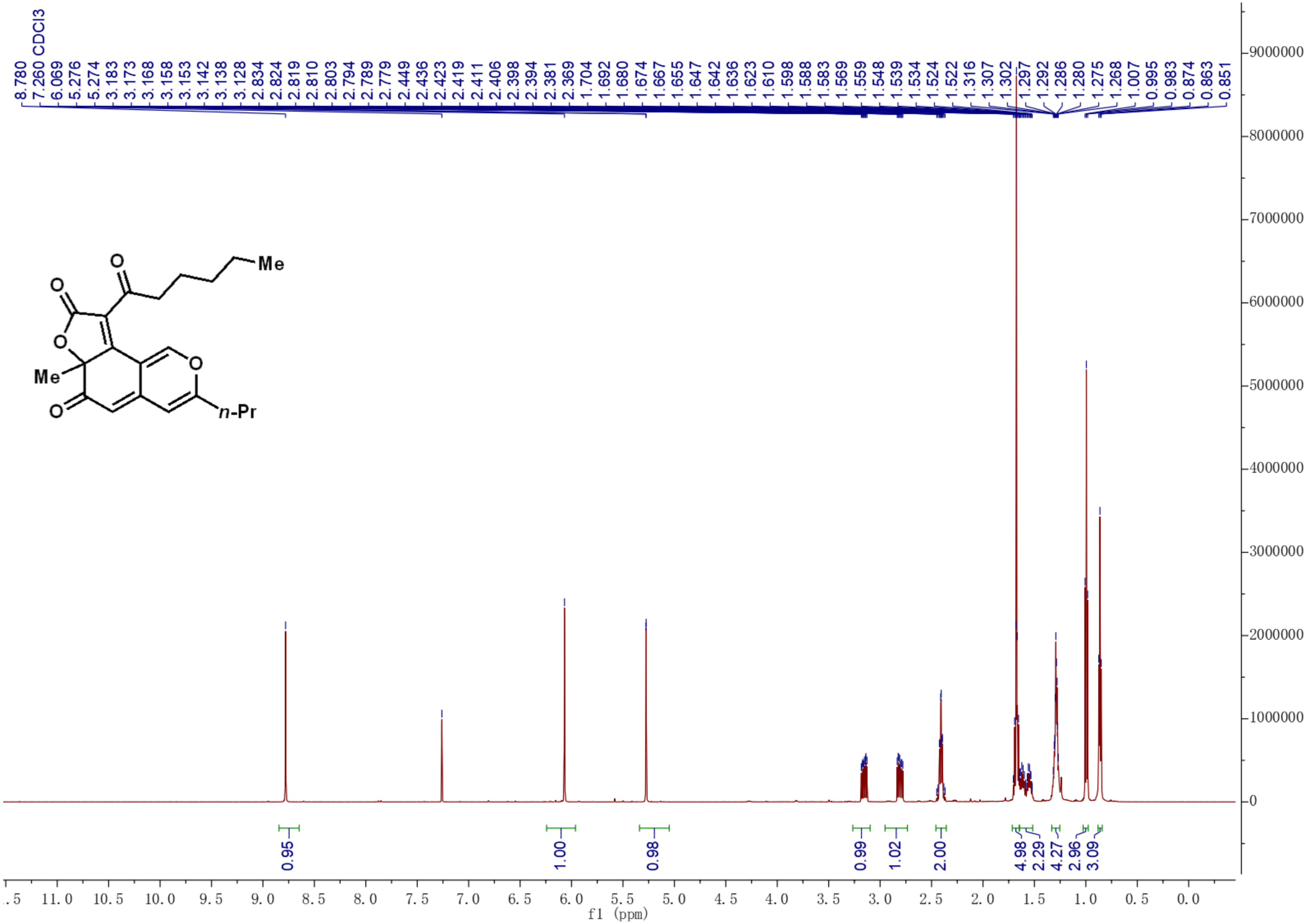

23: ^{13}C NMR (150 MHz, $CDCl_3$)

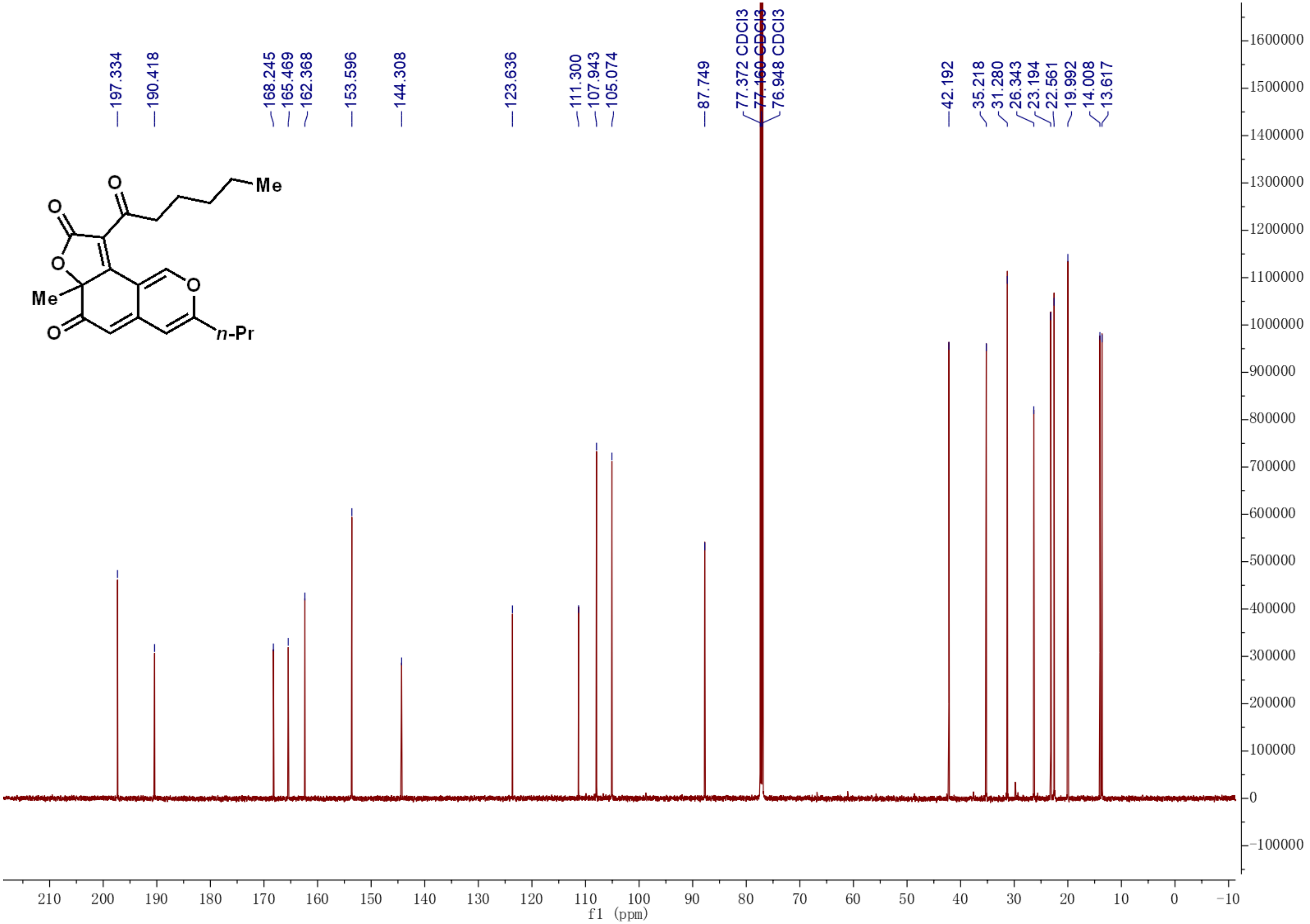

24: ^{1}H NMR (600 MHz, $CDCl_3$)

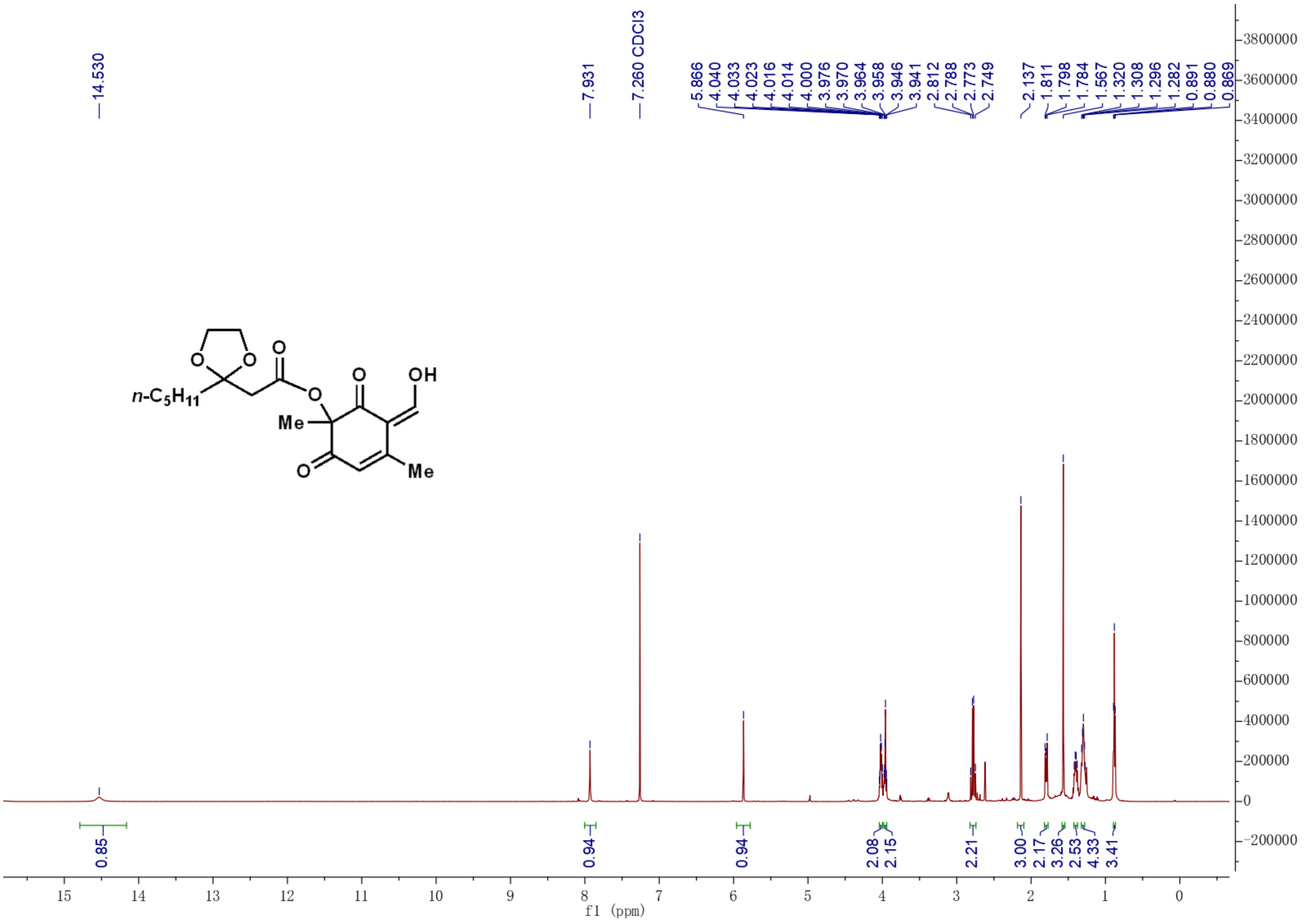

24: ^{13}C NMR (150 MHz, $CDCl_3$)

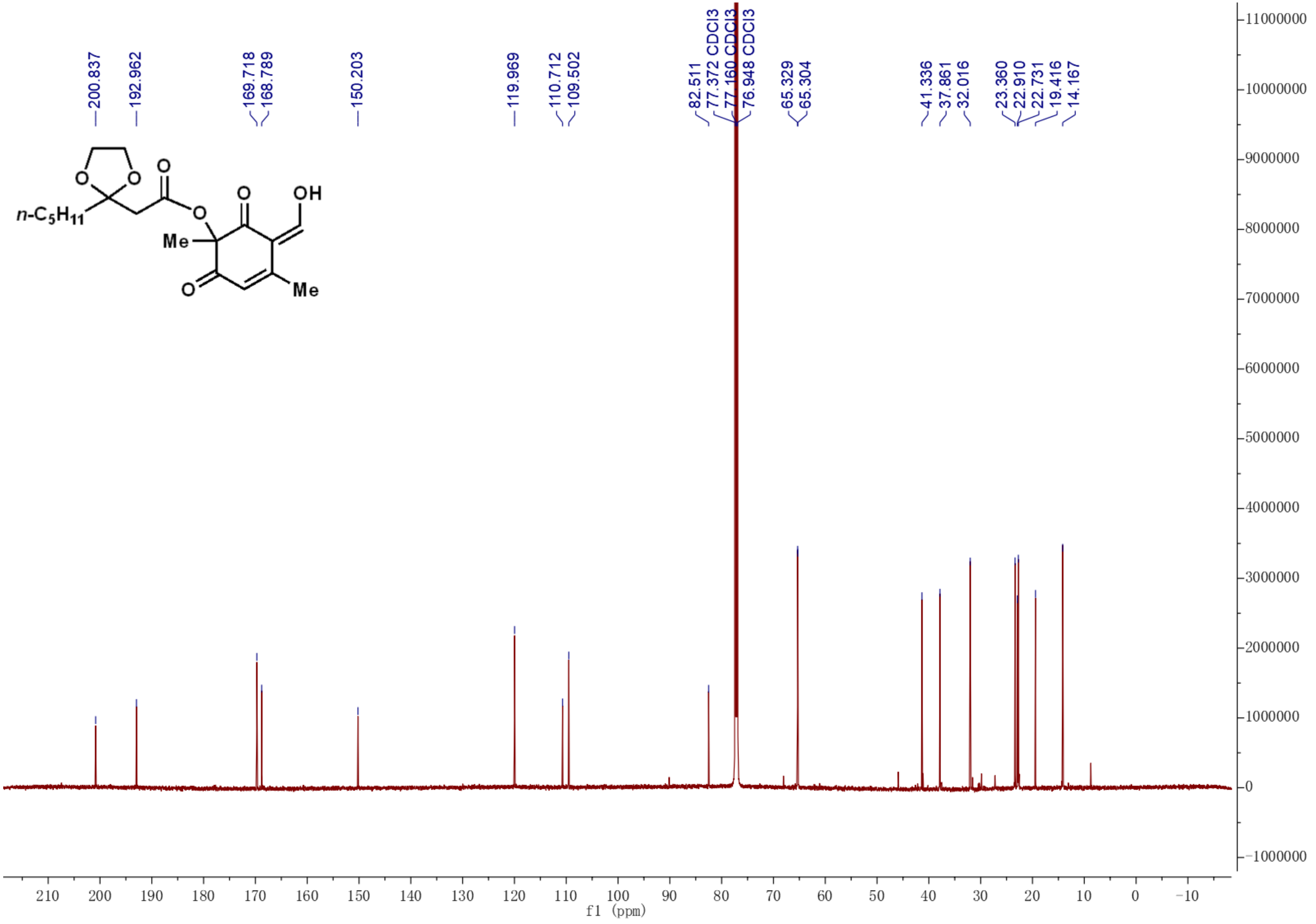

26: ^{1}H NMR (600 MHz, CD_3OD)

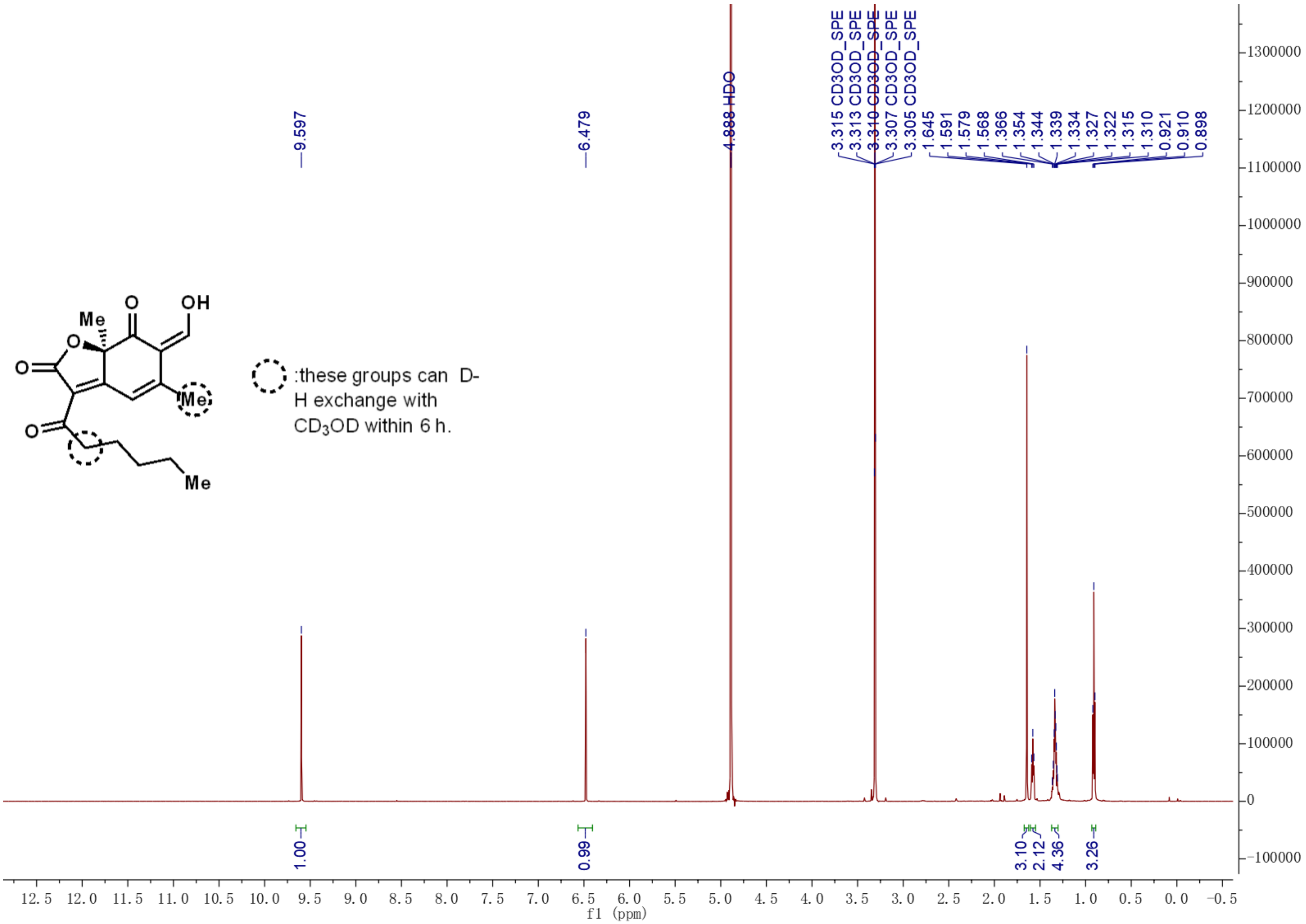

26: ^{13}C NMR (150 MHz, CD_3OD)

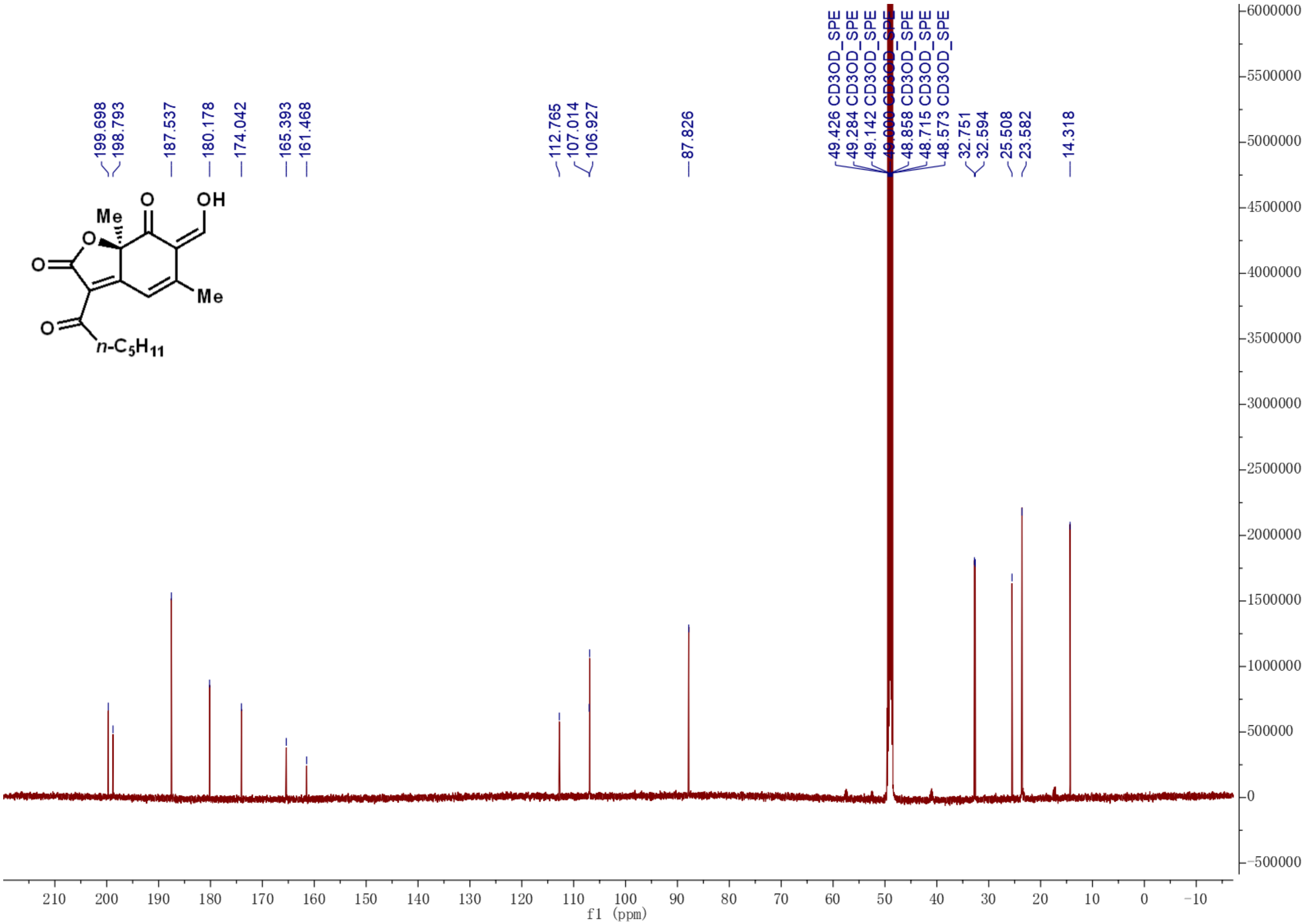

26: ^{1}H NMR (600 MHz, CD_3CN)

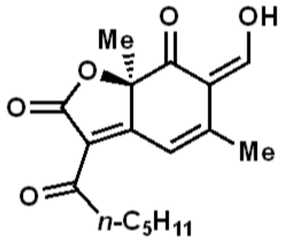

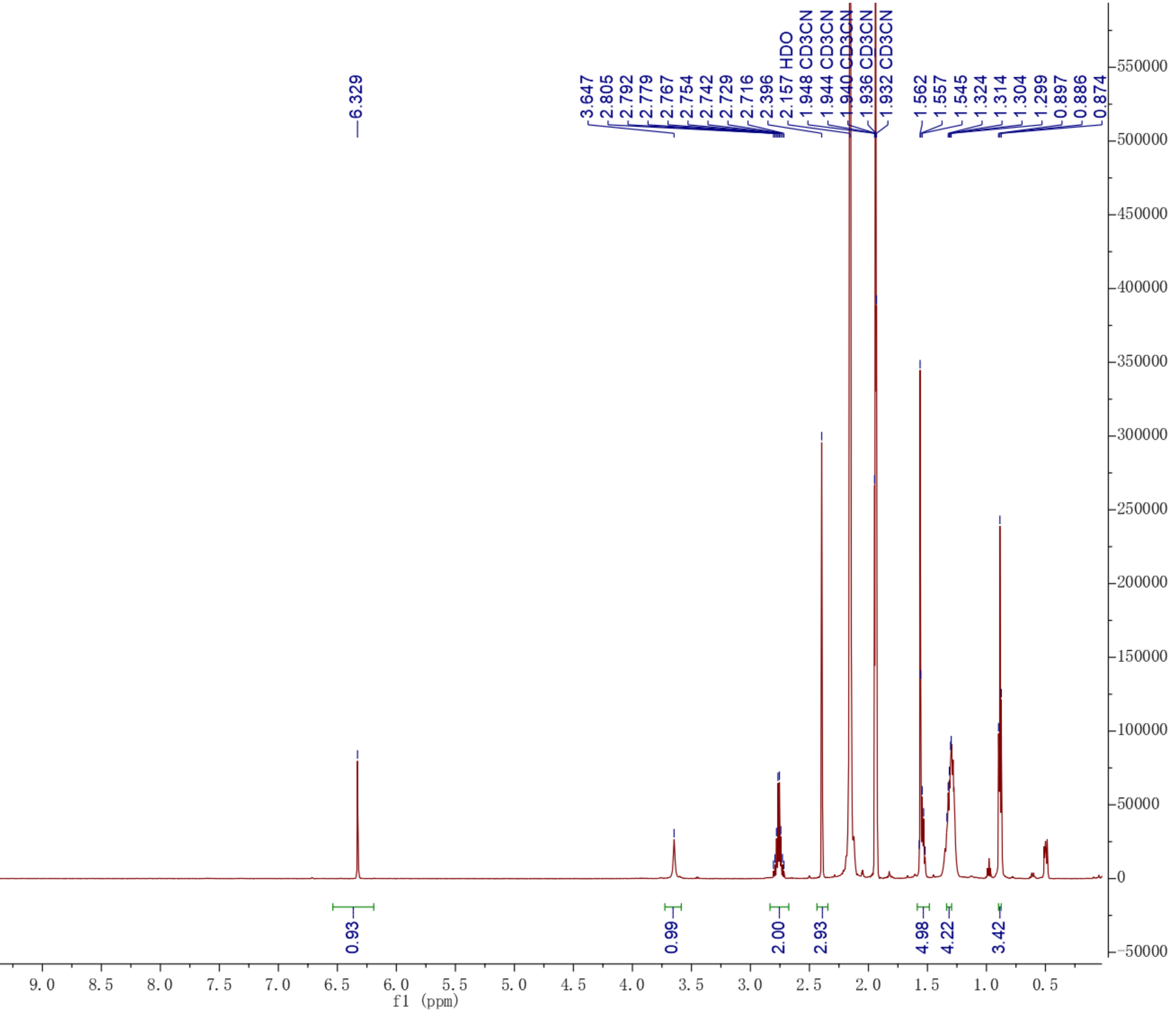

47: ^{1}H NMR (600 MHz, $(CD_3)_2CO$)

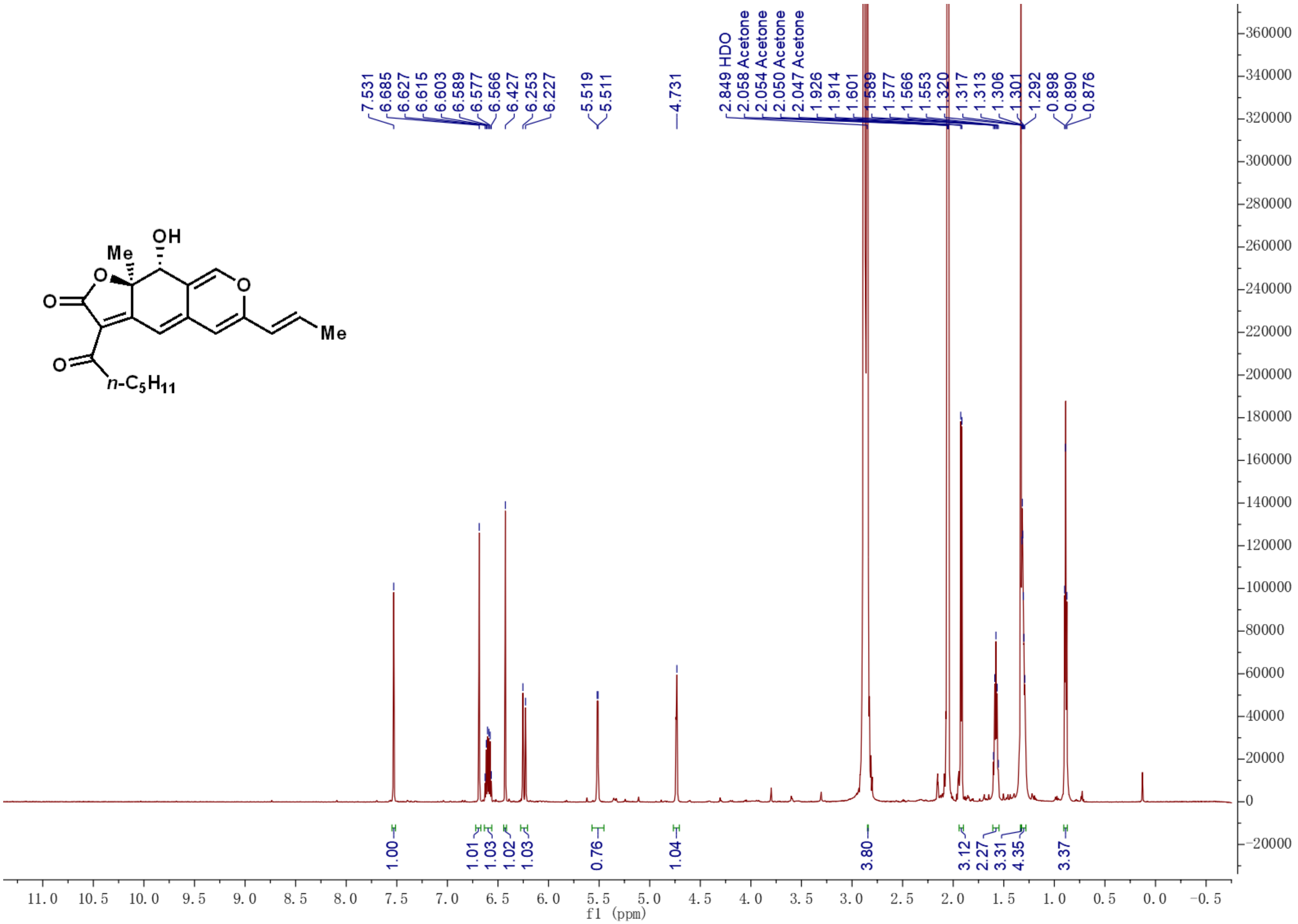

47: ^{13}C NMR (150 MHz, $(CD_3)_2CO$)

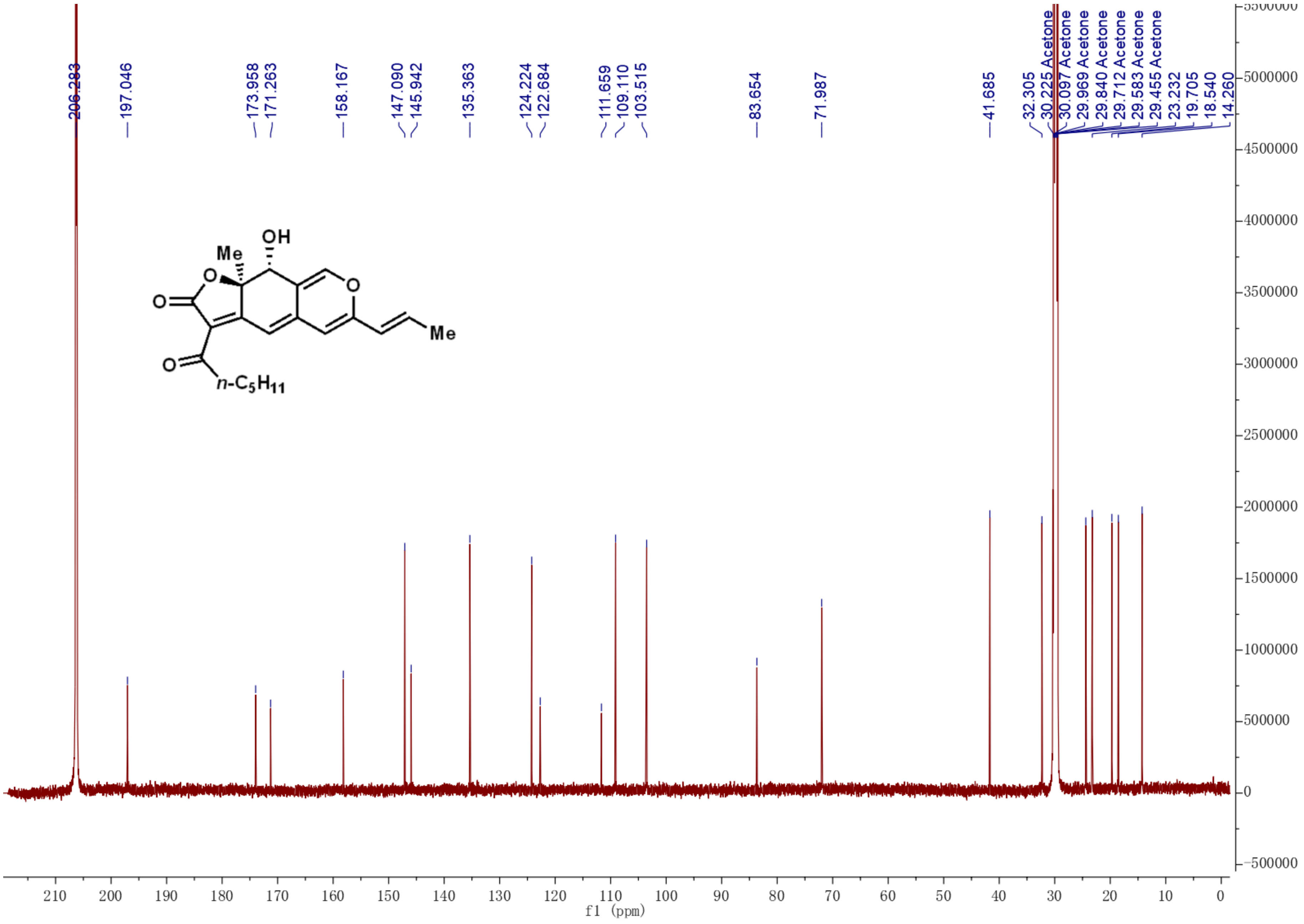

10: ^{1}H NMR (600 MHz, CD_3OD)

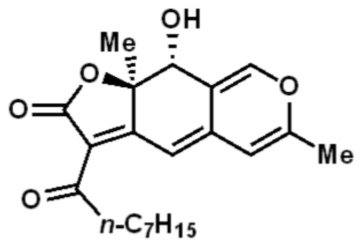

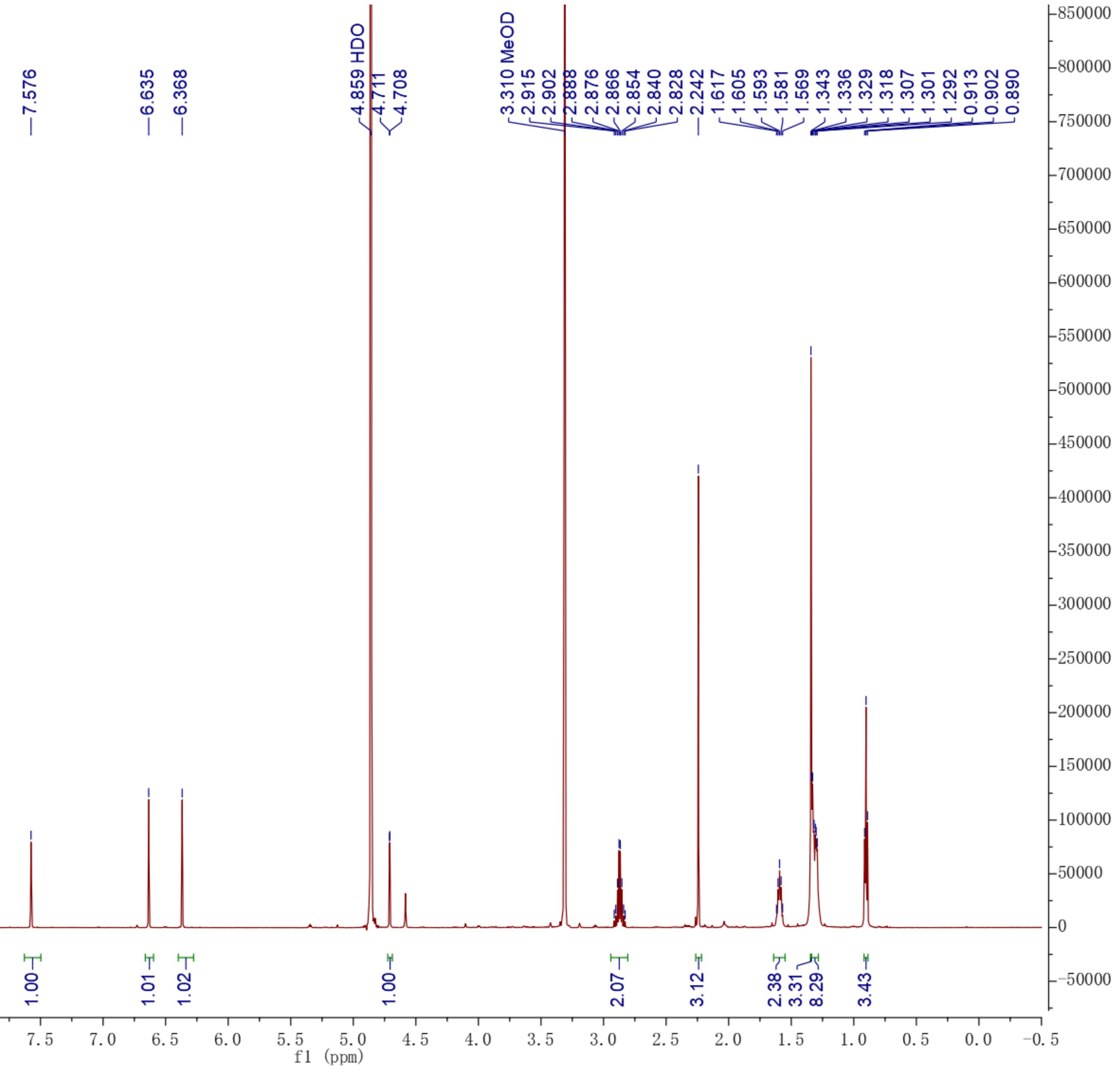

10: ^{13}C NMR (150 MHz, CD_3OD)

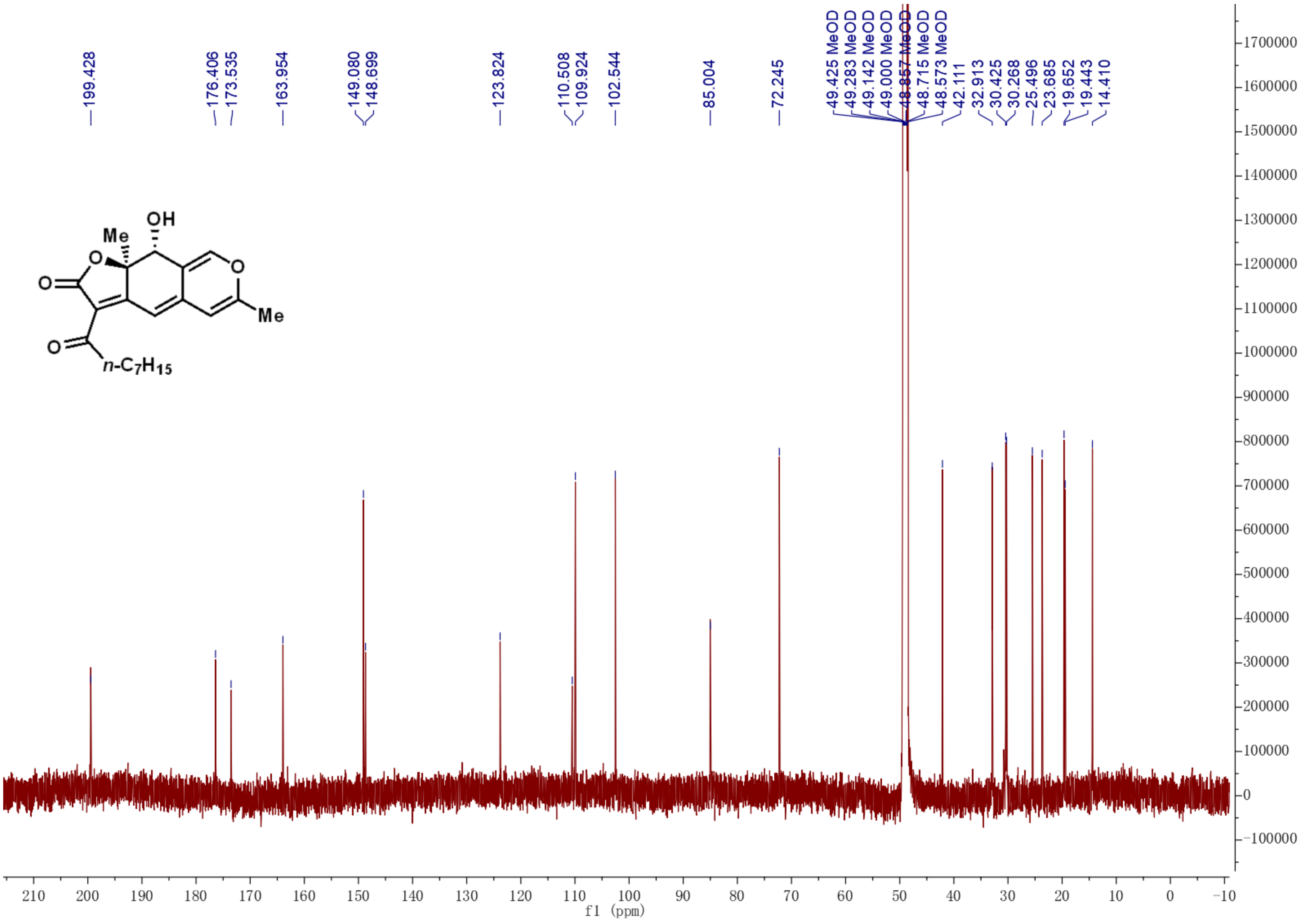

S20: ^1H NMR (600 MHz, $CDCl_3$)

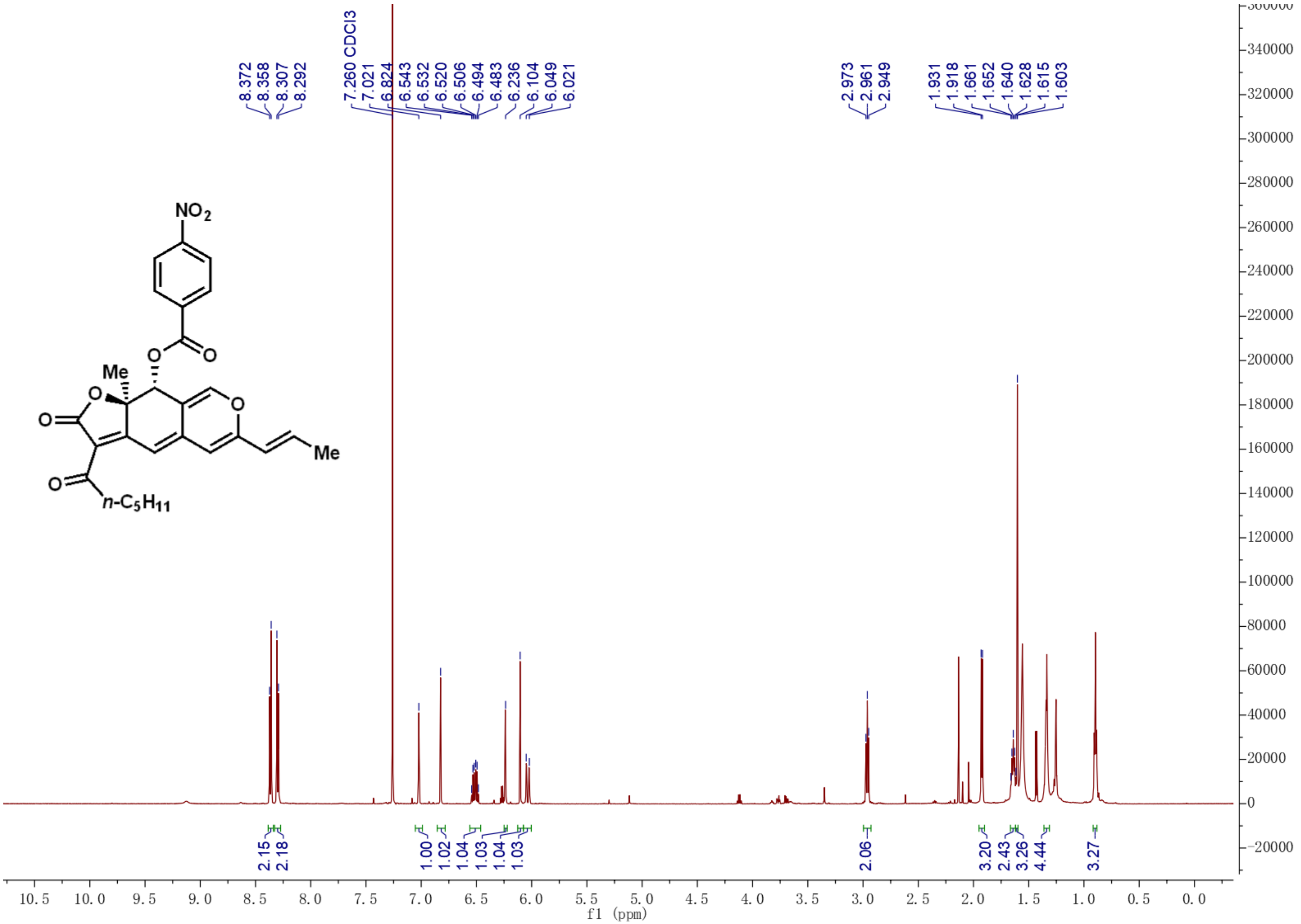

S20: ^{13}C NMR (150 MHz, $CDCl_3$)

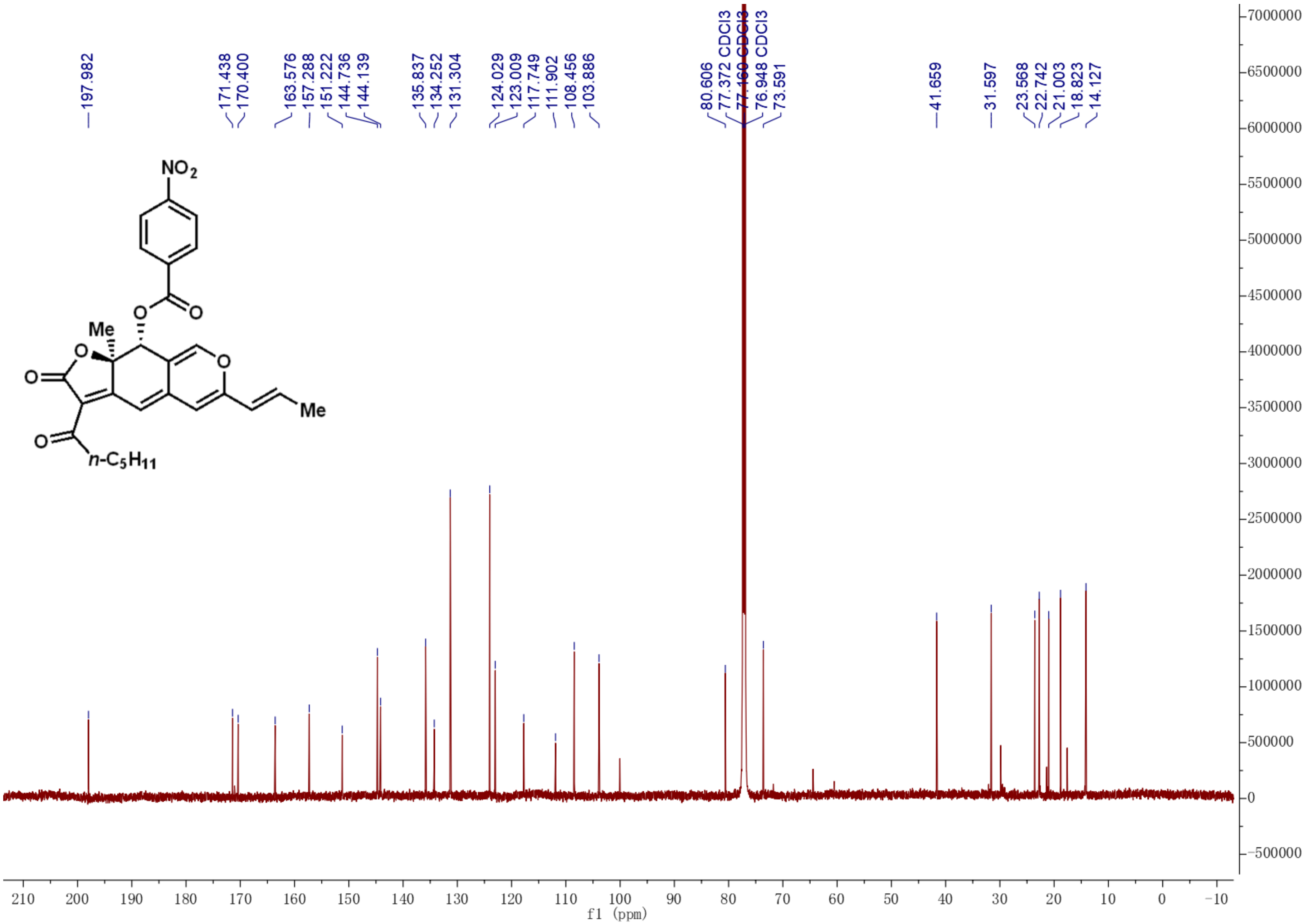

S20: NOE-full spectrum

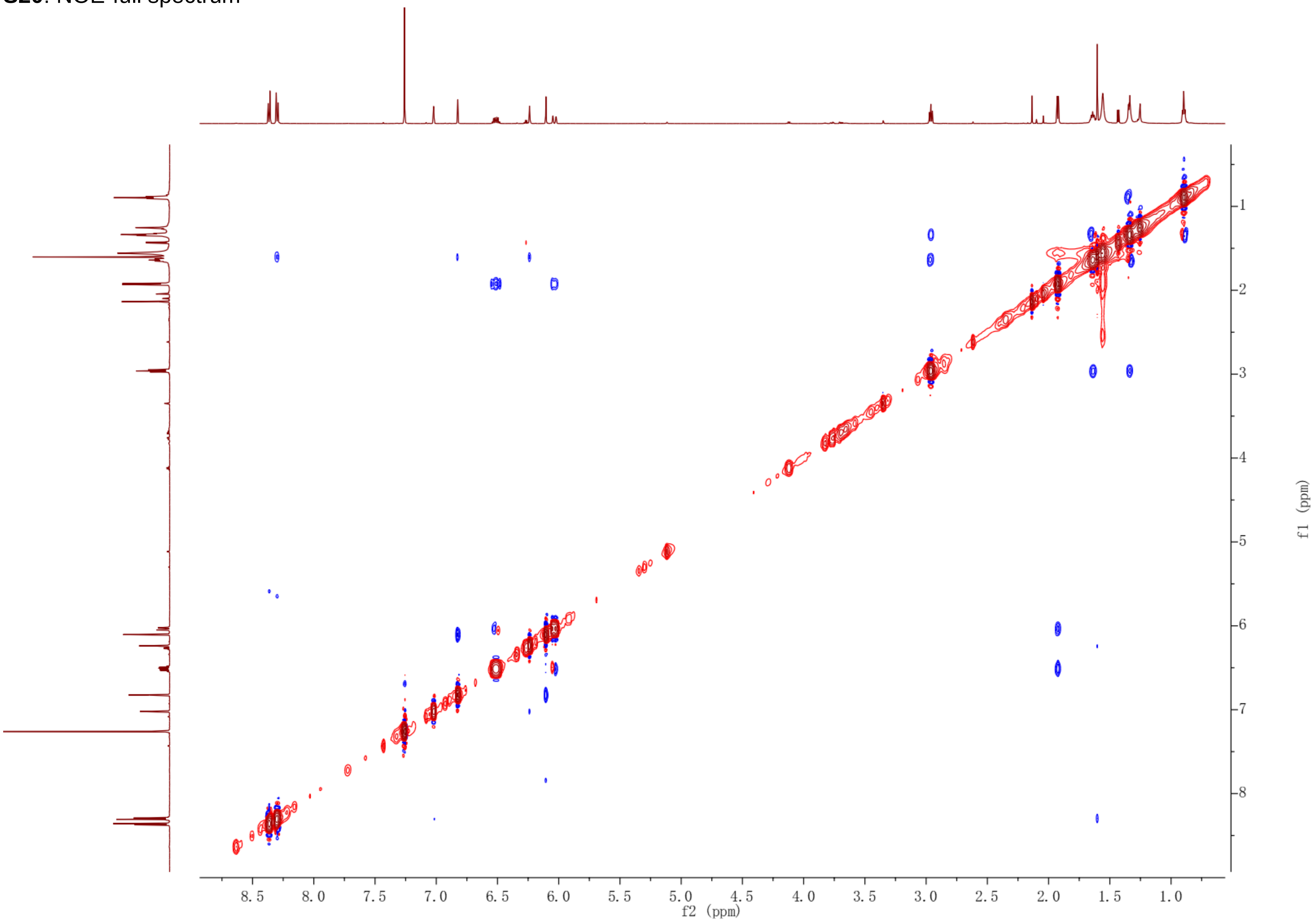

S20: NOE-detail to determine the configuration:

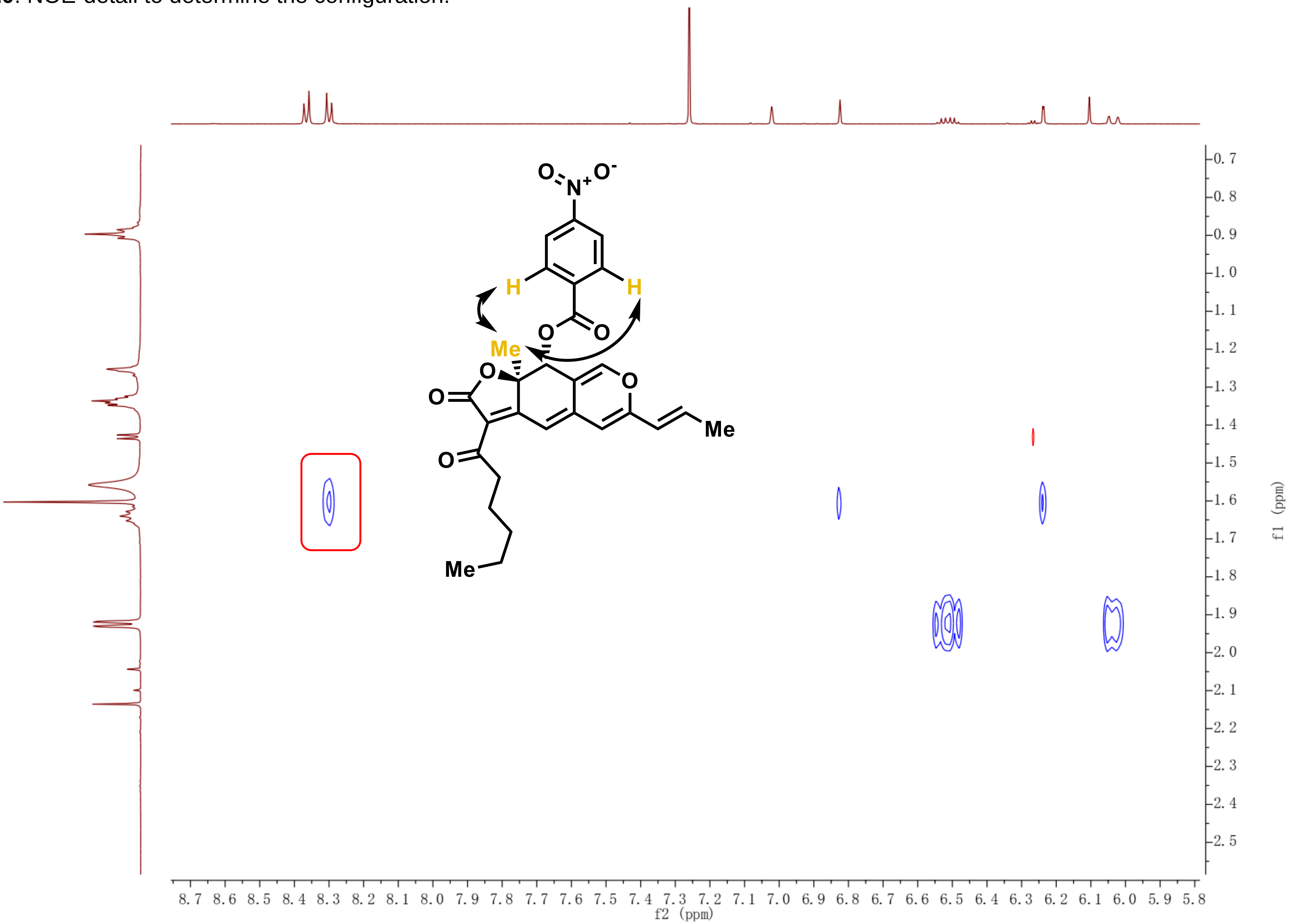

49 ^{1}H NMR (600 MHz, $CDCl_3$)

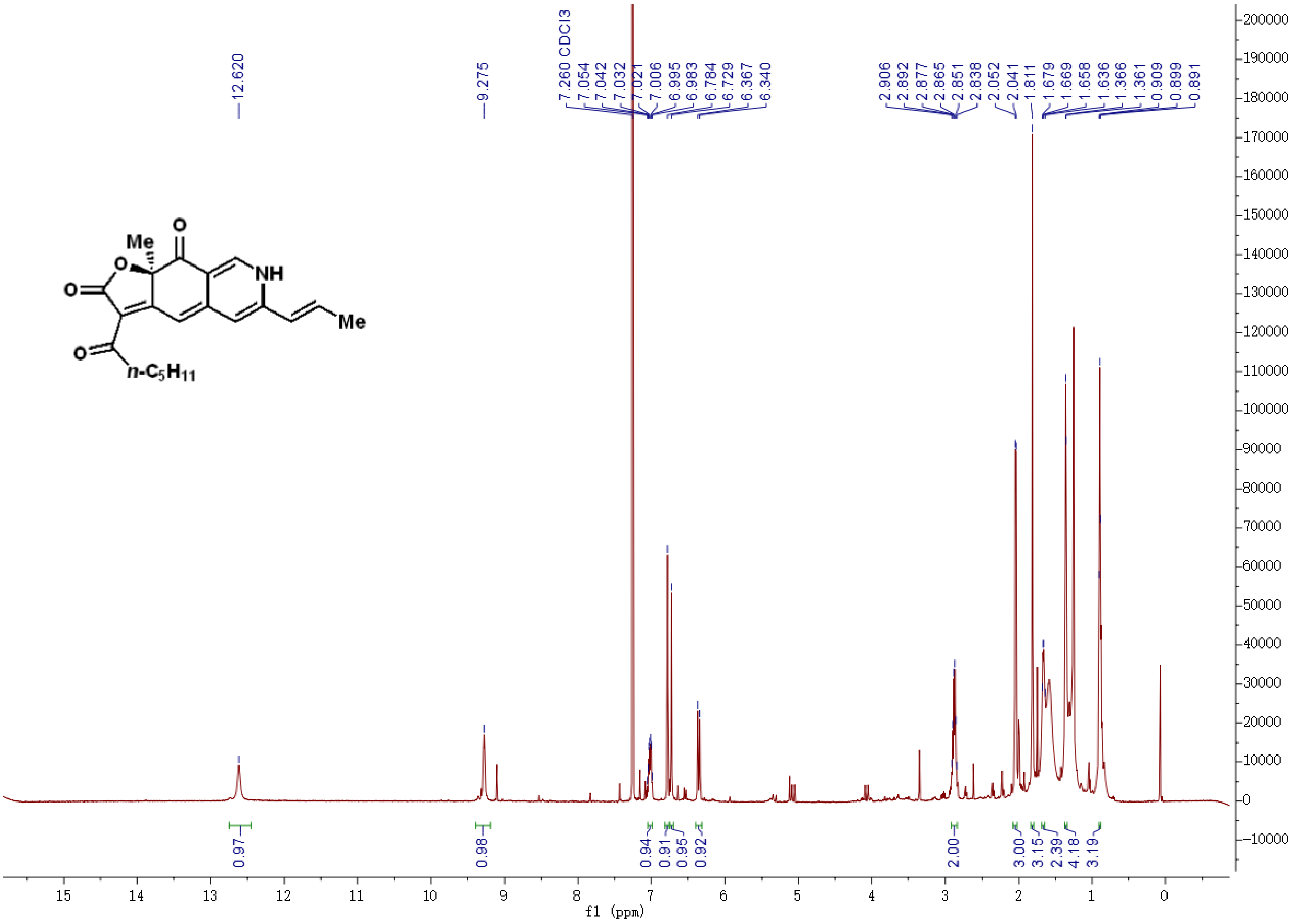

49: ^{13}C NMR (150 MHz, $CDCl_3$)

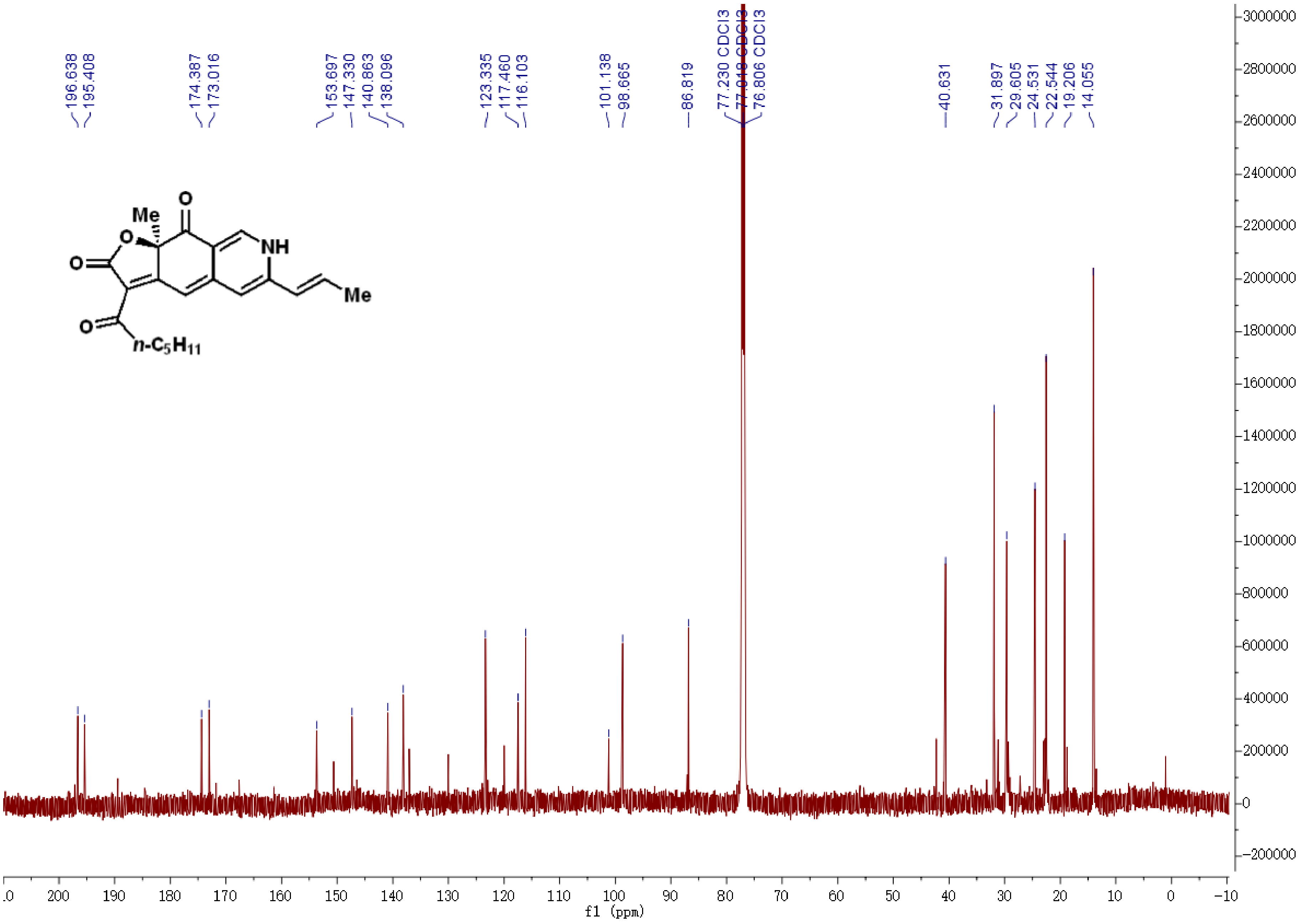

50 ^{1}H NMR (600 MHz, CD_3OD)

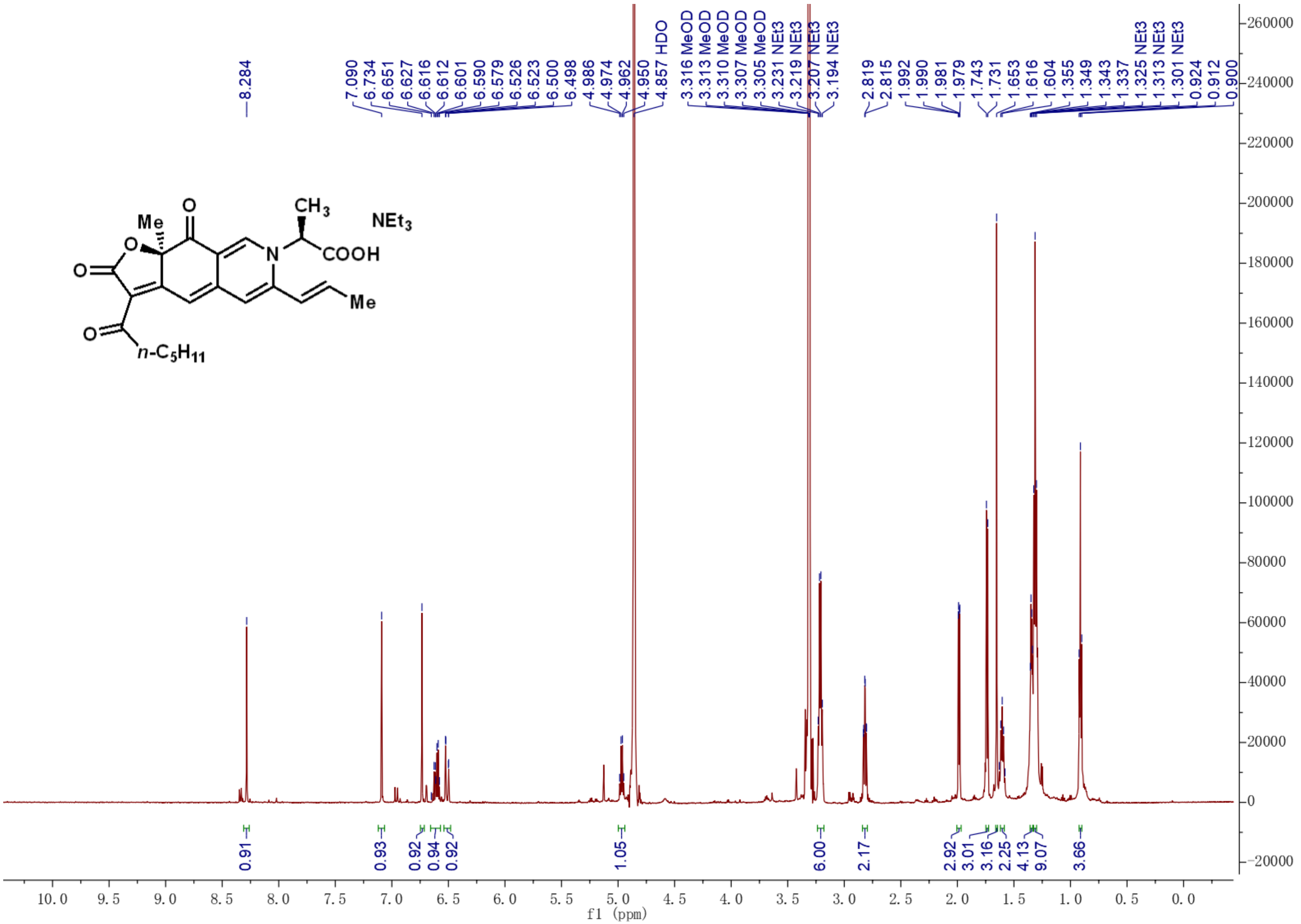

50: ^{13}C NMR (150 MHz, CD_3OD)

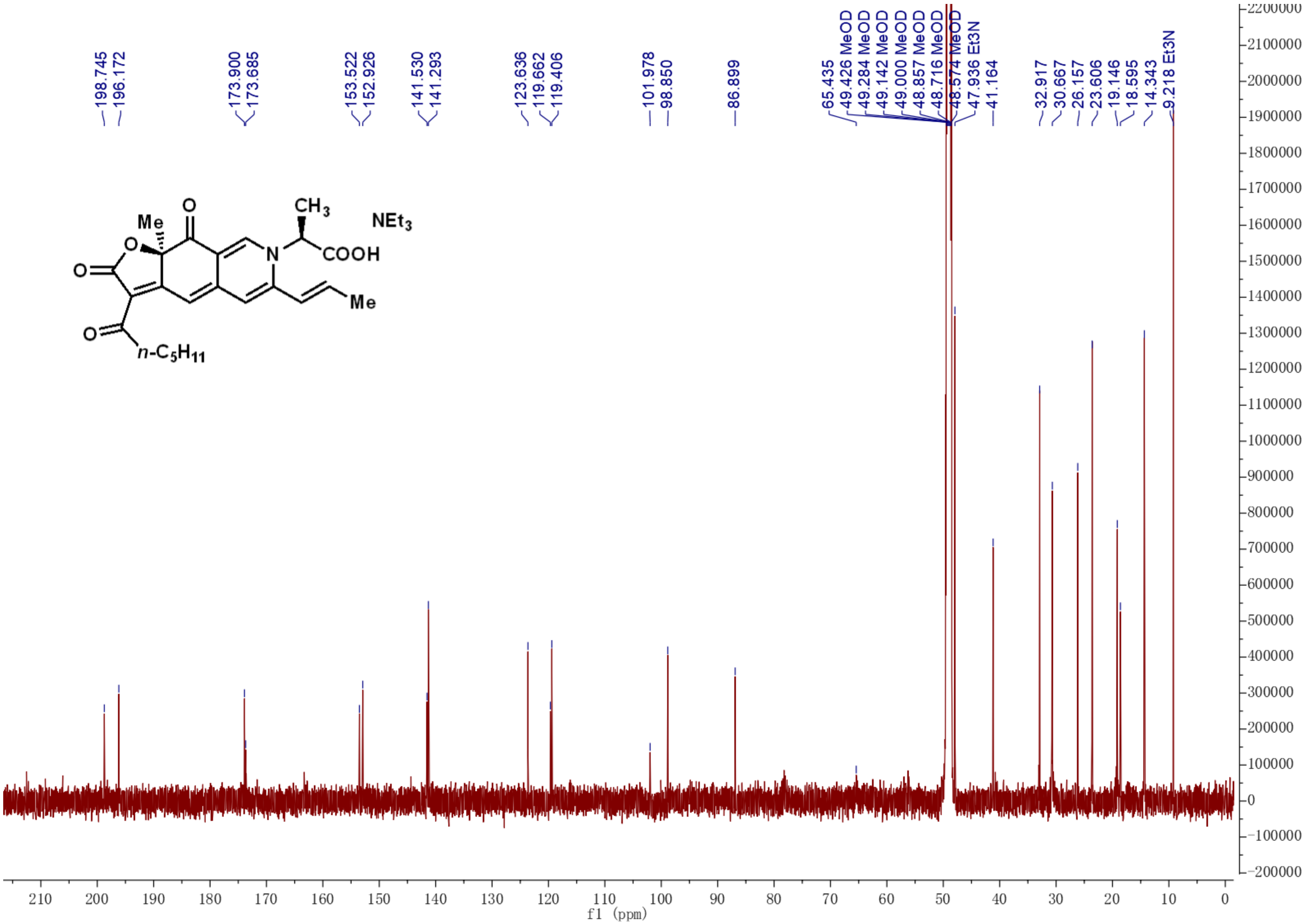

XIV. References

1. Baker Dockrey, S. A.; Lukowski, A. L.; Becker, M. R.; Narayan, A. R. H., Biocatalytic site- and enantioselective oxidative dearomatization of phenols. *Nat. Chem.* **2018,** *10,* 119-125.

2. Pyser, J. B.; Baker Dockrey, S. A.; Benítez, A. R.; Joyce, L. A.; Wiscons, R. A.; Smith, J. L.; Narayan, A. R. H., Stereodivergent, Chemoenzymatic Synthesis of Azaphilone Natural Products. *J. Am. Chem. Soc.* **2019,** *141,* 18551-18559.

3. Agarwal, V.; Diethelm, S.; Ray, L.; Garg, N.; Awakawa, T.; Dorrestein, P. C.; Moore, B. S., Chemoenzymatic Synthesis of Acyl Coenzyme A Substrates Enables in Situ Labeling of Small Molecules and Proteins. *Org. Lett.* **2015,** *17,* 4452-4455.

4. Chen, W.; Chen, R.; Liu, Q.; He, Y.; He, K.; Ding, X.; Kang, L.; Guo, X.; Xie, N.; Zhou, Y.; Lu, Y.; Cox, R. J.; Molnár, I.; Li, M.; Shao, Y.; Chen, F., Orange, red, yellow: biosynthesis of azaphilone pigments in Monascus fungi. *Chem. Sci.* **2017,** *8,* 4917-4925.

5. Zabala, Angelica O.; Xu, W.; Chooi, Y.-H.; Tang, Y., Characterization of a Silent Azaphilone Gene Cluster from Aspergillus niger ATCC 1015 Reveals a Hydroxylation-Mediated Pyran-Ring Formation. *Chem. Biol.* **2012,** *19,* 1049-1059.

6. Huang, Z.; Zhang, S.; Xu, Y.; Li, L.; Li, Y., Structural characterization of two new orange pigments with strong yellow fluorescence. *Phytochem. Lett.* **2014,** *10,* 140-144.

7. Zheng, Y.; Xin, Y.; Shi, X.; Guo, Y., Cytotoxicity of Monascus Pigments and Their Derivatives to Human Cancer Cells. *J. Agric. Food. Chem.* **2010,** *58,* 9523-9528.

8. Liu, L.; Wu, S.; Wang, W.; Zhang, X.; Wang, Z., Sulfonation of Monascus pigments to produce water-soluble yellow pigments. *Dyes Pigm.* **2020,** *173,* 107965.

9. Hsu, Y.-W.; Hsu, L.-C.; Liang, Y.-H.; Kuo, Y.-H.; Pan, T.-M., New Bioactive Orange Pigments with Yellow Fluorescence from Monascus-Fermented Dioscorea. *J. Agric. Food. Chem.* **2011,** *59,* 4512-4518.

10. Sato, K.; Goda, Y.; Sakamoto Sasaki, S.; Shibata, H.; Maitani, T.; Yamada, T., Identification of Major Pigments Containing D-Amino Acid Units in Commercial Monascus Pigments. *Chem. Pharm. Bull.* **1997,** *45,* 227-229.

11. Haws, E. J.; Holker, J. S. E.; Kelly, A.; Powell, A. D. G.; Robertson, A., 722. The chemistry of fungi. Part XXXVII. The structure of rubropunctatin. *J. Chem. Soc.* **1959,** 3598-3610.

12. Kumasaki, S.; Nakanishi, K.; Nishikawa, E.; Ohashi, M., Structure of monascorubrin. *Tetrahedron* **1962,** *18,* 1171-1184.
